# Chiral cyclopropenimine-catalyzed enantioselective Michael reactions of phenol and benzofuran-derived α,β-unsaturated pyrazolamides with benzophenone-imine of glycine esters

**DOI:** 10.3762/bjoc.22.69

**Published:** 2026-06-08

**Authors:** Ya Bai, Xue-Ying Wang, Si-Kai Zhu, Yan-Ting Shen, Sheng-Yong Zhang, Ping-An Wang

**Affiliations:** 1 Department of Medicinal Chemistry and Pharmaceutical Analysis, School of Pharmacy, Fourth Military Medical University, Xi’an, 710032, China; 2 Department of Pharmacy, Xijing Hospital, Fourth Military Medical University, Xi’an, 710032, China; 3 School of Pharmacy, Lanzhou University, Lanzhou, 730000, China

**Keywords:** α,β-unsaturated pyrazolamide, chiral cyclopropenimine, glutamic acid, lactamization, Michael addition

## Abstract

The enantioselective Michael reactions of benzophenone-imine of glycine esters with phenol- and benzofuran-derived α,β-unsaturated pyrazolamides have been realized by using a chiral cyclopropenimine (Lambert catalyst, **CSB-1**) as an organocatalyst. In the presence of 20 mol % **CSB-1**, the Michael adducts were obtained in up to 85% yield and 98% ee under mild conditions. The configurations of these Michael products were deduced by X-ray single crystal diffraction of a pyroglutamic acid ester containing two adjacent stereocenters, which was obtained from in-situ acidic hydrolysis and lactamization of the corresponding Michael product.

## Introduction

**Figure 1:**
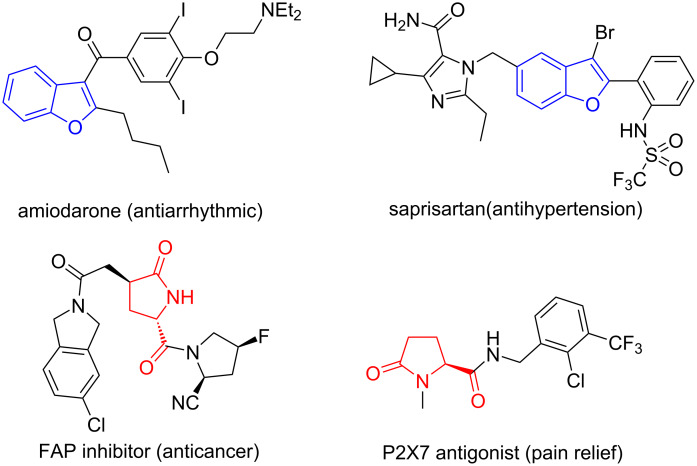
Bioactive molecules with benzofuran and pyroglutamic acid motif.

**Figure 2:**
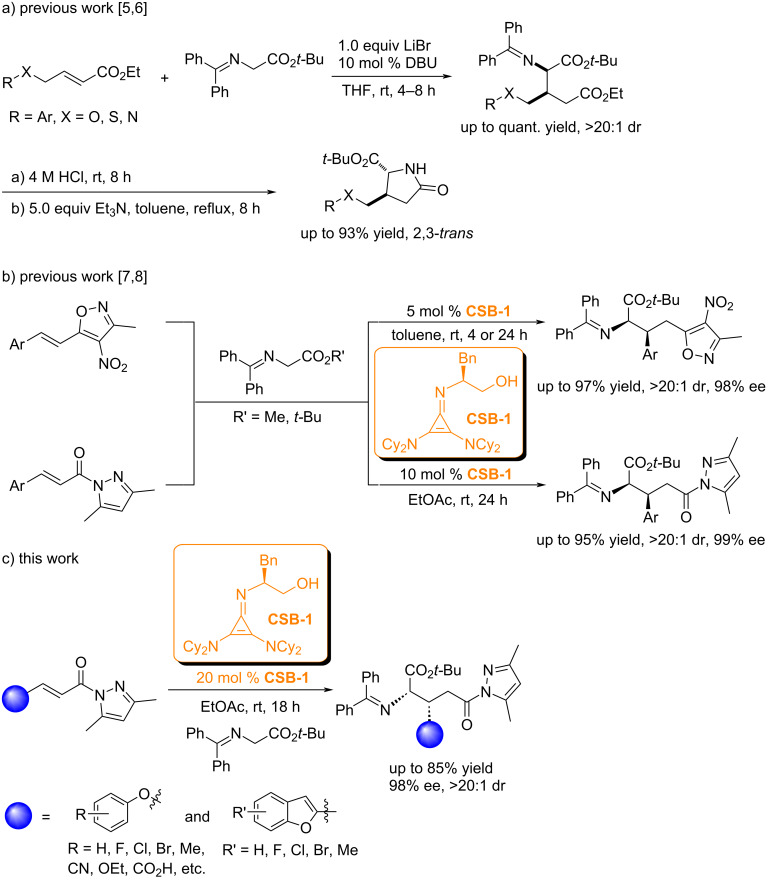
**CSB-1**-catalyzed Michael reactions of α,β-unsaturated compouds with glycine benzophenone-imine esters.

Phenols and benzofurans are feedstock chemicals in organic synthesis. Many medicines and intermediates contain these two motifs and show diverse functions and bioactivities [1–2]. The esterification and etherification of phenols are some of their common modifications. 2-Substituted benzofurans are backbones for many medicines such as amiodarone, dronedarone, saprisartan and so on [3]. Therefore, the introduction of benzofuran to organic molecules plays an important role in drug research and development. Compounds containing glutamic and pyroglutamic acid frameworks (Figure 1) indicate many pharmacologic properties including influence on protein synthesis, neurotransmitter function, and regulation of acid-base balance, metabolic intermediates, and promotion of nutrient absorption [4]. Therefore, the synthesis of substituted glutamic and pyroglutamic acid derivatives is vital to medicinal chemistry. In our previous reports, we have synthesized a series of 3-substitued glutamic acid esters in high yields and diastereoselectivities through DBU-catalyzed Michael additions of benzophenone-imine of glycine ester and α,β-unsaturated esters which derived from phenols and benzofurans under mild conditions [5–6]. The chiral 3-aryl-substituted glutamic and pyroglutamic acid esters were also produced by enantioselective Michael additions of β-aryl-substituted α,β-unsaturated pyrazolamides with benzophenone-imine of glycine ester in excellent ee and de values by using a chiral cyclopropenimine (Lambert catalyst, Figure 2b) as an organosuperbase catalyst [7–8]. Due to the importance of unnatural amino acids in the development of new medicines [9], we want to introduce phenol and benzofuran motifs to glutamic acids. For our continuous interest on the synthesis of chiral 3-substituted glutamic and pyroglutamic acids, herein, we present Michael reactions of phenol- and benzofuran-derived α,β-unsaturated pyrazolamides with benzophenone-imine of glycine esters by a chiral cyclopropenimine (**CSB-1**) to give 3-substituted glutamic and pyroglutamic acid esters in up to 98% ee and 20:1 diastereomeric ratio. In this reaction, a phenoxymethyl or benzofuryl group was introduced to the 3-position of glutamic and pyroglutamic acid esters, respectively (Figure 2c).

## Results and Discussion

**Scheme 1:**
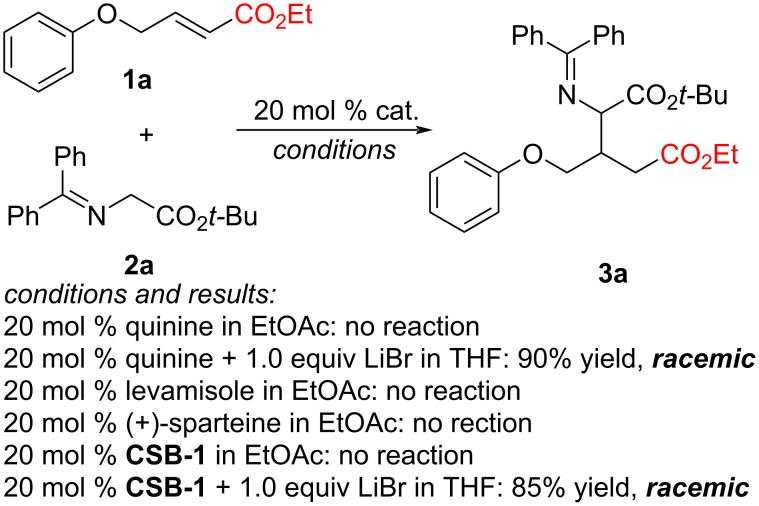
Chiral base-catalyzed Michael reaction of **1a** and **2a**.

In fact, some simple α,β-unsaturated esters can be used as substrates to give Michael adducts in good yields and stereoselectivties [10–11]. Firstly, we tried to explore asymmetric Michael reactions between β-substituted α,β-unsaturated ester **1a** and benzophenone-imine of glycine *tert*-butyl ester **2a** in the presence of 20 mol % of **CSB-1** and other chiral tertiary amine catalysts including quinine, levamisole, (+)-sparteine (Scheme 1). To our disappointment, the results showed that the chiral catalysts quinine and **CSB-1** can give the corresponding product **3a** in high yields but in its racemic form in the presence of 1 equiv LiBr.

**Scheme 2:**
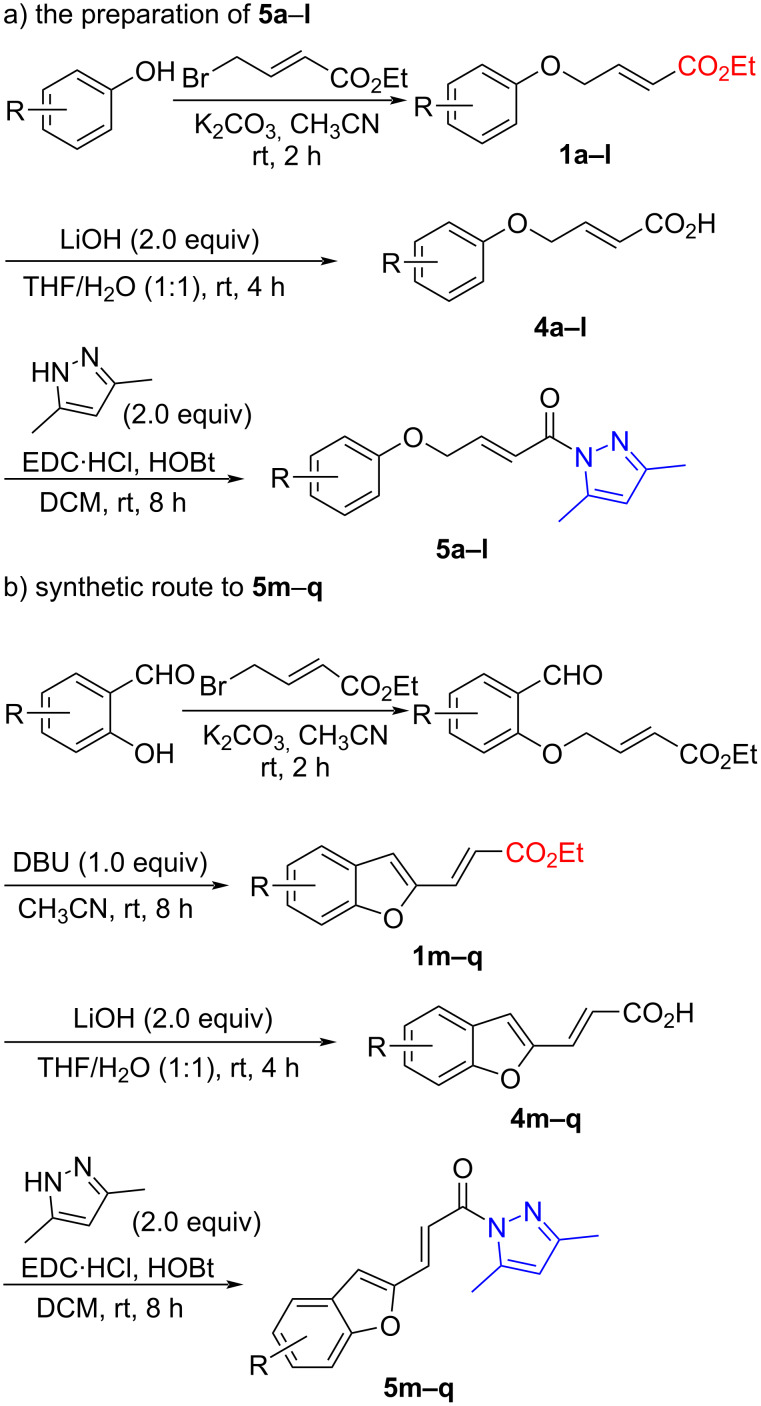
The preparation of β-substituted α,β-unsaturated pyrazolamides **5**.

Based on our previous research [7–8], β-aryl-substituted α,β-unsaturated isoxazoles and pyrazolamide can be used as Michael acceptors to produce Michael adducts in high yields and enantioselectivities in the presence of Lambert catalyst **CSB-1**. Therefore, the phenol-derived pyrazolamides **5a**–**l** were prepared from phenols, ethyl 4-bromocrotonate and 3,5-dimethyl-1*H*-pyrazole in three steps including alkylation, hydrolysis and amidation (Scheme 2a). The benzofuran-derived pyrazolamides **5m**–**q** were synthesized through DBU-mediated cyclocondensation of 2-hydroxybenzaldehydes with 4-bromocrotonates to afford (*E*)-2-benzofuranyl-3-acrylates followed hydrolysis and coupling with 3,5-dimethyl-1*H*-pyrazole (Scheme 2b) [12].

**Figure 3:**
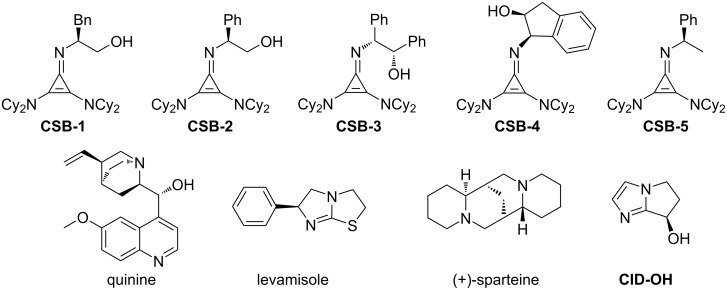
The catalysts used in the screening of Michael reaction conditions.

Initially, the Michael reaction between α,β-unsaturated pyrazolamide **5a** and benzophenone-imine of glycine ester **2a** was performed as a model reaction for reaction conditions screening with nine chiral organocatalysts (Figure 3) including five Lambert catalysts (**CSB-1**–**CSB-5**), quinine, levamisole, (+)-sparteine, and chiral imidazole (**CID-OH**). Among these nine catalysts, it was found that **CSB-1** derived from ʟ-phenylalaninol demonstrates good chiral induction among five chiral cyclopropenimines to afford **6a** in high yield (85%) and excellent stereoselectivities (98% ee, dr > 20:1, Table 1, entry 1). To our surprise, **CSB-2** derived from ʟ-phenylglycinol only provided trace product **6a** (Table 1, entry 2), although its structure is very similar to **CSB-1**. **CSB-3** based on vicinal amino-alcohol backbone also afforded trace product. The other catalysts resulted in no reaction of **2a** and **5a** (Table 1, entries 3–9). These results show the unique catalytic and stereospecific ability of **CSB-1**. This may be due to a more flexible hydrogen-bonding donor group and a smaller steric hinderance in **CSB-1** than in other cyclopropenimines to provide good hydrogen-bonding interaction in the transition state [13]. With decreasing amount of **CSB-1** from 20 mol % to 10 mol %, the yield of **6a** dropped to 76% but with an excellent ee value (Table 1, entry 10 vs entry 1). The yields and enantioselectivities of **6a** are both decreased in dichloromethane (DCM), toluene, and acetone (Table 1, entries 11–13). When THF was used as a solvent, the yield of **6a** was slightly lower than in ethyl acetate (EA) as a solvent (Table 1, entry 14 vs entry 1). The reaction carried out in acetonitrile provided racemic **6a** (Table 1, entry 15). When MeOH was used as a solvent, it led to no reaction (Table 1, entry 16), this may due to the large amount of hydrogen-bonding interactions in MeOH to inactivate the catalytic effect of **CSB-1**. Based on these results, the optimal reaction conditions are listed in Table 1, entry 1. The reactions between **2** and **5** were carried out in EtOAc by using 20 mol % of **CSB-1** as a catalyst at room temperature.

**Table 1:** The screening of reaction conditions^a^.

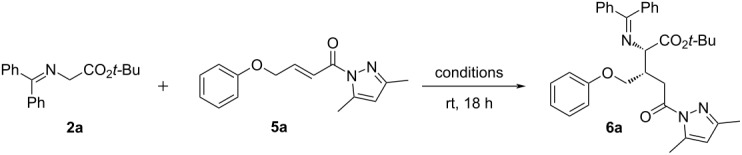				

Entry^a^	Catalyst	Solvent	Yield (%)^b^	ee (%)^c^

**1**	**CSB-1 (0.2 equiv)**	**EA**	**85**	**98**
2	**CSB-2** (0.2 equiv)	EA	trace	–
3	**CSB-3** (0.2 equiv)	EA	trace	–
4	**CSB-4** (0.2 equiv)	EA	n.r.	–
5	**CSB-5** (0.2 equiv)	EA	n.r.	–
6	quinine (0.2 equiv)	EA	n.r.	–
7	levamisole (0.2 equiv)	EA	n.r.	–
8	(+)-sparteine (0.2 equiv)	EA	n.r.	–
9	**CID-OH** (0.2 equiv)	EA	n.r.	–
10^d^	**CSB-1** (0.1 equiv)	EA	76	97
11	**CSB-1** (0.2 equiv)	DCM	37	47
12	**CSB-1** (0.2 equiv)	toluene	52	89
13	**CSB-1** (0.2 equiv)	acetone	76	45
14	**CSB-1** (0.2 equiv)	THF	78	96
15	**CSB-1** (0.2 equiv)	CH_3_CN	79	racemic
16	**CSB-1** (0.2 equiv)	CH_3_OH	n.r.	–

^a^**2a** (0.1 mmol), **5a** (0.1 mmol) and 0.5 mL solvent were used. ^b^Isolated yield based on **5a**. ^c^Enantiomeric excess (ee) was measured by HPLC analysis using a chiralcel OD-H column. ^d^Reaction time was 36 h.

**Scheme 3:**
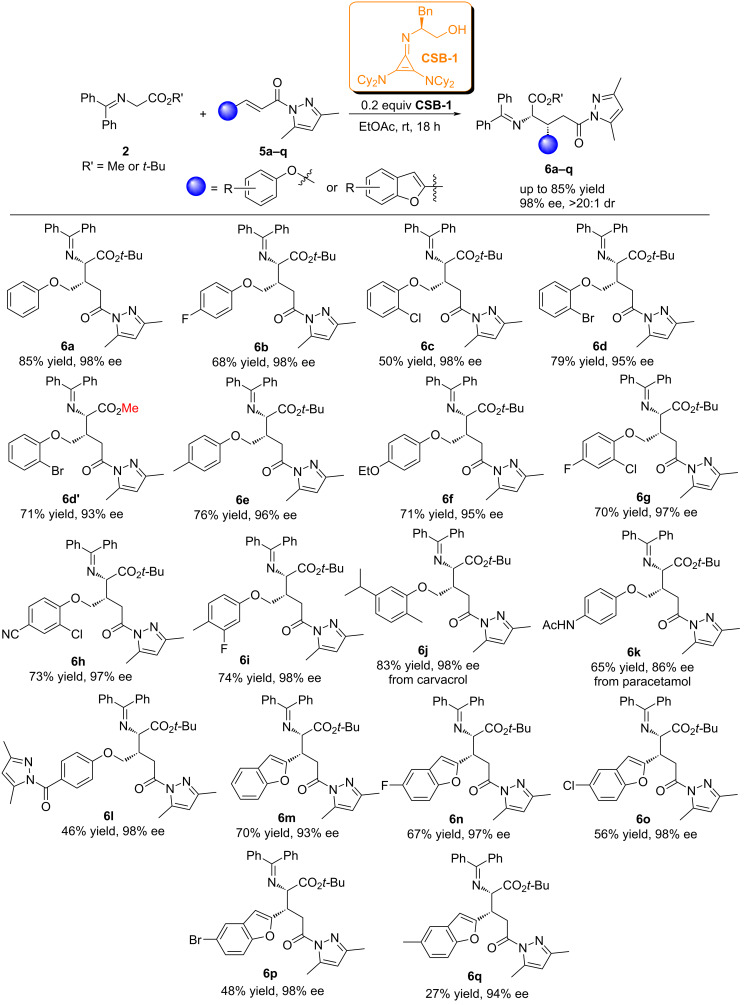
**CSB-1**-catalyzed Michael additions between compounds compounds **2** and **5**.

With the optimal conditions in hand, the asymmetric Michael reactions of α,β-unsaturated pyrazolamides **5a–q** with benzophenone-imine of glycine esters **2** (Scheme 3) were performed to provide the corresponding products **6a–q** in moderate to good yields (up to 85%) with excellent ee values (up to 98% ee). The substrates from phenols **5a–l** containing electron-withdrawing or electron-donating groups have afforded chiral Michael adducts in good yields and enantioselectivities, however, substrates from benzofurans **(5m–p)** have produced the corresponding Michael adducts in moderate yields except **5q**, which gave **6q** in 27% yield but with 94% ee. Carvacrol-derived substrate **5j** and paracetamol-derived substrate **5k** provided **6j** and **6k** in good yields and enantioselectivities. These results demonstrated that some natural molecules and medicines containing a phenol group can be modified by this protocol to introduce a glutamic acid motif to their molecular structures.

**Figure 4:**
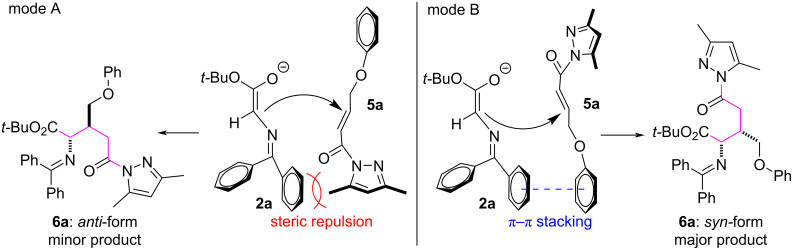
The proposed attack modes of Michael addition of **2a** and **5a**.

A possible explanation for the high diastereoselectivity for this Michael reaction is shown in Figure 4. By attack according to mode A, the *anti*-form of **6a** should be obtained, however, the steric repulsion between benzophenone-imine **2a** and the 3,5-dimethylpyrazolyl group in **5a** may inhibit this attack pathway to afford the *anti*-form of **6a**. With mode B, the π–π stacking between benzophenone-imine **2a** and the phenoxylmethyl group in **5a** may enhance the ratio of this attack to produce the *syn*-form of **6a** as major product.

**Figure 5:**
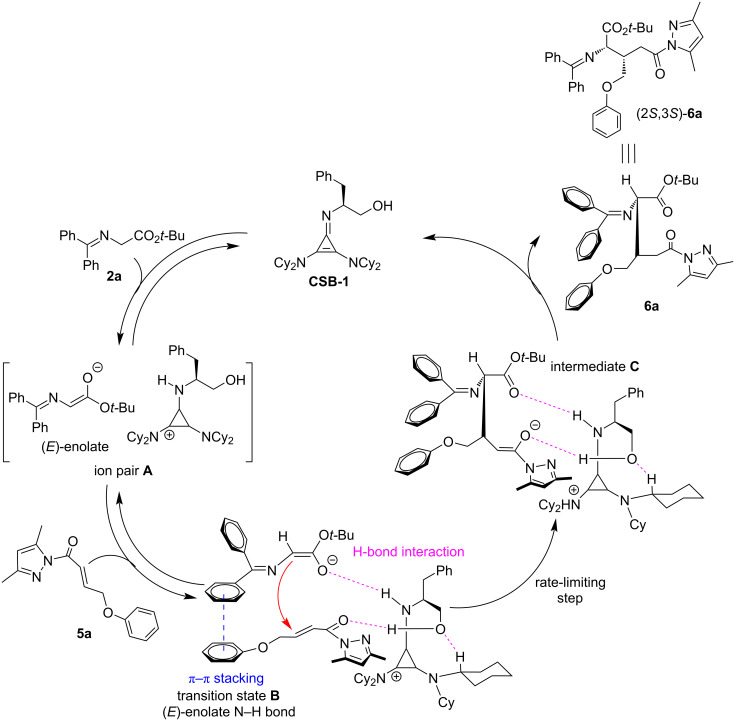
A proposed reaction mechanism of **CSB-1** catalyzed Michael reaction between **2a** and **5a**.

A mechanism for this highly enantioselective Michael addition between **2a** and **5a** was proposed based on experimental facts and the studies of the Lambert group [13]. This proposed reaction mechanism is demonstrated in Figure 5. At the beginning of the reaction, the chiral organosuperbase catalyst **CSB-1** can deprotonate benzophenone-imine of glycine ester **2a** to form the (*E*)-enolate of **2a** and a cyclopropenium as ion pair **A**, which attacks β-substituted α,β-unsaturated pyrazolamide **5a** to provide transition state **B**. Transition state **B** may be stabilized through an H-bonding interaction network between the two substrates and **CSB-1**. Additionally, π–π stacking between the two phenyl groups of **2a** and **5a** may also enhance the formation of transition state **B**. The highly diastereomic ratio of **6a** in *syn*-form may be generated from transition state **B**. The formation of intermediate **C** is the rate-limiting step. When intermediate **C** is formed, the protonation happened rapidly to afford **6a** in high enantioselectivity. The catalyst **CSB-1** was released and entered the next catalytic cycle.

**Scheme 4:**
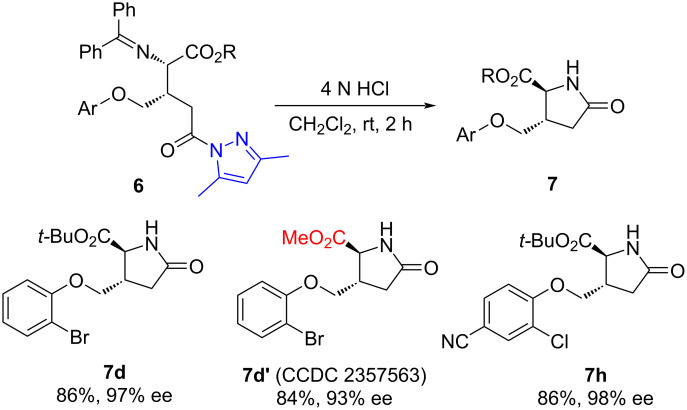
In situ acidic hydrolysis and lactamization of **6**.

The pyroglutamic acid esters **7** were obtained through in-situ acidic hydrolysis and lactamization of **6** [14]. Three Michael products **6d**, **6d’** and **6h** were treated under 4 N HCl in DCM to give 3-substituted pyroglutamic acid esters **7d**, **7d’** and **7h** in high yields and excellent ee values (Scheme 4)

**Figure 6:**
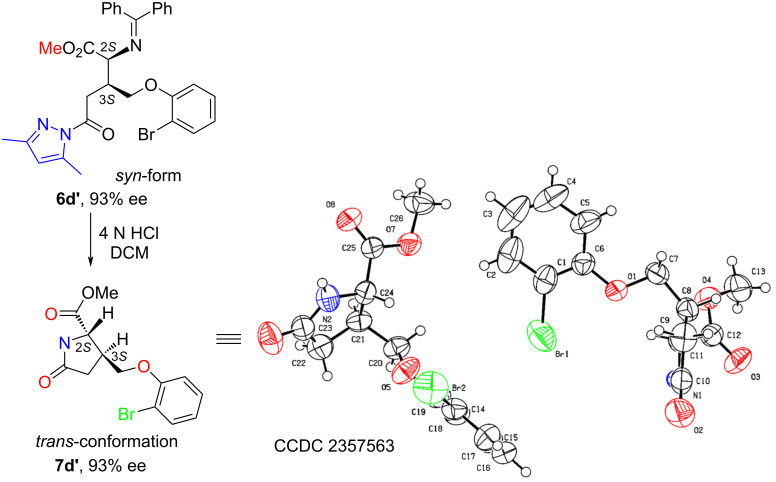
Configuration of **7d’** determined by X-ray diffraction analysis.

To our delight, 3-substituted pyroglutamic acid ester **7d’** was obtained as single crystals for X-ray diffraction analysis [15]. The ester group and the bromophenoxymethyl group were arranged on the other side of the pyrrolidinone ring to be in *trans*-conformation and the absolute configurations of **7d’** was unambiguously assigned as 2*S* and 3*S*, this means that the benzophenone-imine group and bromophenoxymethyl group should be arranged on the same side of the longest carbon chain to be *syn* before lactamization, therefore, the absolute configuration of **6d’** was deduced to be 2*S* and 3*S* either (Figure 6).

## Conclusion

In conclusion, we have developed facile routes to chiral 3-substituted glutamic and pyroglutamic acid esters from phenols and benzofuran-derived α,β-unsaturated pyrazolamides in the presence of Lambert catalyst **CSB-**1 under mild conditions in good yields and enantioselectivities. The absolute configuration of chiral 3-substituted pyroglutamic acid ester were determined by X-ray single crystal diffraction. This protocol can be used for the late-stage modification of bioactive molecules containing a phenol group. The synthesis of chiral 3,4-disubstituted glutamic and pyroglutamic acid esters by this protocol are underway.

## Experimental

### General procedure for the **CSB-1**-catalyzed Michael addition of compounds **2** and **5**

To **2a** (1.0 mmol) and **5a** (1.0 mmol) in EtOAc (10.0 mL) was added **CSB-1** (0.2 mmol) and the mixture was stirred at rt for 18 h. After the reaction was completed (detected by TLC), the solvent was removed by a rotary evaporator under reduced pressure. The residue was purified by flash column chromatography (petroether/EtOAc/Et_3_N 40:1:0.01–20:1:0.01, v/v) to afford pure **6a** as a colorless sticky oil.

### Typical procedure for in situ acidic hydrolysis and lactamization to compound **7d**

To **6d** (1.0 mmol) in DCM (5.0 mL) was added 4 N HCl (5.0 mL) and the mixture was stirred at rt for 2–3 h. After the reaction was completed (detected by TLC), it was quenched by H_2_O (15.0 mL) and extracted with DCM (2 × 25.0 mL). The combined organic layers were dried over Na_2_SO_4_ and the solvent was evaporated under vacuum. The residue was purified by flash column chromatography to afford pure **7d** as a white solid.

## Supporting Information

File 1 Detailed experimental procedures, characterization data of all new compounds with NMR, HRMS, HPLC charts, and X-ray single crystal diffraction data.

## Data Availability

All data that supports the findings of this study is available in the published article and/or the supporting information of this article.
