# Supplementary material for: Chiral cyclopropenimine-catalyzed enantioselective Michael reactions of phenol and benzofuran-derived α,β-unsaturated pyrazolamides with benzophenone-imine of glycine esters
Source: Beilstein J Org Chem. 2026 Jun 8;22:888–96. doi: 10.3762/bjoc.22.69 (PMC13267487; doi:10.3762/bjoc.22.69)
Supplement: File 1 — Detailed experimental procedures, characterization data of all new compounds with NMR, HRMS, HPLC charts, and X-ray single crystal diffraction data. [file Beilstein_J_Org_Chem-22-888-s001.pdf]

BEILSTEIN JOURNAL OF ORGANIC CHEMISTRY

Supporting Information

for

Chiral cyclopropenimine-catalyzed enantioselective Michael reactions of phenol and benzofuran-derived α,β-unsaturated pyrazolamides with benzophenone-imine of glycine esters

Ya Bai, Xue-Ying Wang, Si-Kai Zhu, Yan-Ting Shen, Sheng-Yong Zhang and Ping-An Wang

Beilstein J. Org. Chem. **2026,** *22,* 888–896. doi:10.3762/bjoc.22.69

Detailed experimental procedures, characterization data of all new compounds with NMR, HRMS, HPLC charts, and X-ray single crystal diffraction data

License and Terms: This is a supporting information file under the terms of the Creative Commons Attribution License (https://creativecommons.org/licenses/by/4.0). Please note that the reuse, redistribution and reproduction in particular requires that the author(s) and source are credited and that individual graphics may be subject to special legal provisions.
The license is subject to the *Beilstein Journal of Organic Chemistry* terms and conditions: (https://www.beilstein-journals.org/bjoc/terms)

Table of Contents

1. General..........S3
2. Experimental sections..........S3
3. X-ray diffraction data..........S17
4. NMR scanning copies..........S19
5. HRMS scanning copies..........S69
6. HPLC charts..........S85

1. General

The ^{1}H NMR, ^{13}C NMR, ^{19}F NMR spectra were measured in acetone-*d*$_6$, $CDCl_3$, or DMSO-*d*$_6$ solution on a Bruker AV-400 spectrometer using TMS as an internal reference. Coupling constant (*J*) values are given in Hz. Multiplicities are designated by the following abbreviations: s, singlet; d, doublet; t, triplet; q, quartet; br, broad; m, multiplet. High-resolution mass spectromrtry (HRMS) was performed on a Bruker microTOF-Q II Mass Spectrometer with ES ionization (ESI). All commercially available reagents were used as received. Thin-layer chromatography on silica (with GF254) was used to monitor all reactions. Products were purified by flash column chromatography on silica gel purchased from Qingdao Haiyang Chemical Co., Ltd. The configurations of the products have been assigned by single crystal X-ray diffraction analysis. All solvents, organic and inorganic reagents were from commercial sources and used without purification unless otherwise noted. Glycine esters, β-substituted α,β-unsaturated pyrazolamides were prepared according to literatures reported methods.[1,2] Chiral cyclopropenimine organosuperbsaes (CSBs) **CSB-1-5** were prepared following literature reported procedure, and their characterization data are consistent with reference report.[3] The characterization data of all new compounds are listed in this document.

2. Experimental sections

2.1 The synthesis of substrates

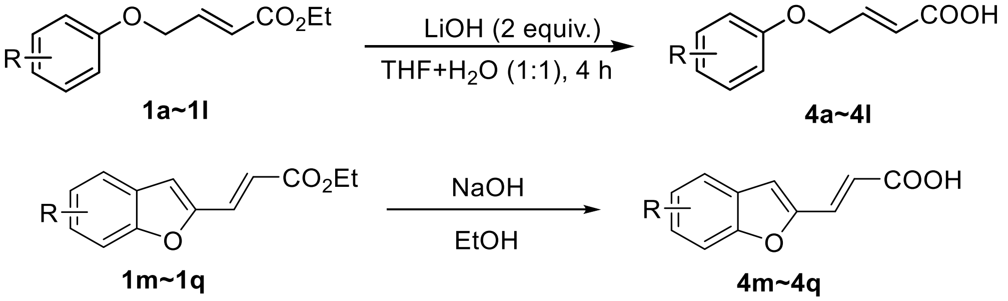

To α,β-unsaturated ester **1** (1 equiv) in THF+H_2O (1:1) added LiOH·H_2O (2 equiv) at 0 °C and then the reaction mixture was stirred at room temperature for 4 h. After the substrate was detected by TLC plate, adjusted the pH to 3-4 with 2 *N* hydrochloric acid at 0 °C. The reaction was quenched by saturated saline, then extracted with DCM. The combined organic layers were dried over Na_2SO_4 and the solvents were evaporated under vacuum to afford α,β-unsaturated acid **4**.

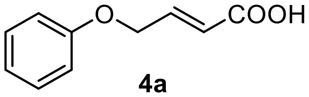

4a

(*E*)-4-Phenoxybut-2-enoic acid (**4a**)[4]: white solid, quant. yield, m.p. 115.4-117.2 °C. ^{1}H NMR (400 MHz, DMSO-*d*$_6$) δ 12.38 (s, 1H), 7.31 (dtd, *J* = 11.6, 8.0, 3.4 Hz, 2H), 7.08 – 6.89 (m, 5H), 6.04 (d, *J* = 15.0 Hz, 1H), 4.77 (d, *J* = 4.4 Hz, 2H).

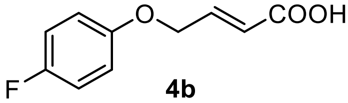

4b

(*E*)-4-(4-Fluorophenoxy)but-2-enoic acid (**4b**): white solid, 97% yield. ^{1}H NMR (400 MHz, DMSO-*d*$_6$) δ 12.43 (s, 1H), 7.19 – 7.09 (m, 2H), 7.04 – 6.90 (m, 3H), 6.04 (dt, *J* = 15.7, 2.0 Hz, 1H), 4.76 (dd, *J* = 4.2, 2.1 Hz, 2H).

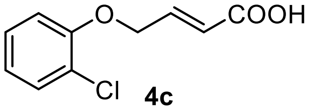

4c

(*E*)-4-(2-Chlorophenoxy)but-2-enoic acid (**4c**): white solid, quant. yield, m.p. 171.9-173.0 °C. ^{1}H NMR (400 MHz, DMSO-*d*$_6$) δ 12.51 (s, 1H), 7.49 – 7.42 (m, 1H), 7.34 – 7.28 (m, 1H), 7.15 (d, *J* = 8.3 Hz, 1H), 7.03 – 6.92 (m, 2H), 6.16 – 6.04 (m, 1H), 4.95 – 4.80 (m, 2H).

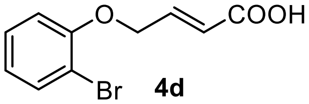

4d

(*E*)-4-(2-Bromophenoxy)but-2-enoic acid (**4d**): white solid, 99% yield, m.p. 177.2-178.5 °C. ^{1}H NMR (400 MHz,

DMSO-d_6) δ 12.5 (s, 1H), δ 7.61 (dd, J = 7.9, 1.6 Hz, 1H), 7.40 – 7.32 (m, 1H), 7.12 (dd, J = 8.3, 1.4 Hz, 1H), 7.01 – 6.89 (m, 2H), 6.12 (dt, J = 15.7, 2.0 Hz, 1H), 4.87 (dd, J = 4.0, 2.1 Hz, 2H).

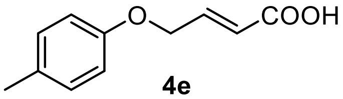

4e

(E)-4-(p-Tolyloxy)but-2-enoic acid (**4e**): white solid, quant. yield, m.p. 158.3-159.2 °C. ^{1}H NMR (400 MHz, DMSO-d_6) δ 7.09 (d, J = 8.3 Hz, 2H), 6.93 (dt, J = 15.8, 4.1 Hz, 1H), 6.88 – 6.81 (m, 2H), 6.01 (dt, J = 15.8, 2.1 Hz, 1H), 4.72 (dd, J = 4.3, 2.0 Hz, 2H), 2.22 (s, 3H).

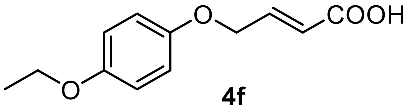

4f

(E)-4-(4-Ethoxyphenoxy)but-2-enoic acid (**4f**): white solid, 97% yield, m.p. 147.1-147.6 °C. ^{1}H NMR (400 MHz, DMSO-d_6) δ 7.00 – 6.81 (m, 5H), 6.03 (dt, J = 15.7, 2.0 Hz, 1H), 4.70 (dd, J = 4.2, 2.1 Hz, 2H), 3.94 (q, J = 6.9 Hz, 2H), 1.29 (t, J = 7.0 Hz, 3H).

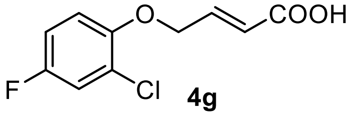

4g

(E)-4-(2-Chloro-4-fluorophenoxy)but-2-enoic acid (**4g**): white solid, quant. yield, m.p. 124.2-125.6 °C. ^{1}H NMR (400 MHz, DMSO-d_6) δ 7.46 (dd, J = 8.3, 2.5 Hz, 1H), 7.22 – 7.15 (m, 2H), 6.95 (dt, J = 15.8, 4.0 Hz, 1H), 6.08 (dt, J = 15.7, 2.1 Hz, 1H), 4.86 (dd, J = 4.1, 2.1 Hz, 2H).

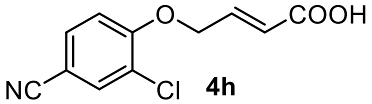

4h

(E)-4-(2-Chloro-4-cyanophenoxy)but-2-enoic acid (**4h**): white solid, 94% yield, m.p. 142.4-143.2 °C. ^{1}H NMR (400 MHz, DMSO-d_6) δ 8.05 (d, J = 2.0 Hz, 1H), 7.87 – 7.84 (m, 1H), 7.32 (d, J = 8.7 Hz, 1H), 6.93 – 6.90 (m, 1H), 6.08 (dt, J = 15.7, 2.0 Hz, 1H), 5.00 (dd, J = 4.1, 2.1 Hz, 2H).

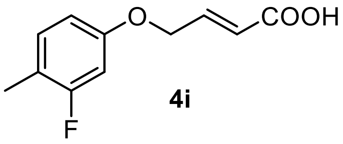

4i

(E)-4-(3-Fluoro-4-methylphenoxy)but-2-enoic acid (**4i**): white solid, 97% yield, m.p.165.7-166.6 °C. ^{1}H NMR (400 MHz, DMSO-d_6) δ 12.44 (s, 1H), 7.17 (t, J = 8.8 Hz, 1H), 6.91 (dt, J = 15.7, 4.2 Hz, 1H), 6.81 (dd, J = 11.8, 2.6 Hz, 1H), 6.72 (dd, J = 8.4, 2.6 Hz, 1H), 6.01 (dt, J = 15.7, 2.0 Hz, 1H), 4.75 (dd, J = 4.3, 2.0 Hz, 2H), 2.14 (d, J = 1.8 Hz, 3H).

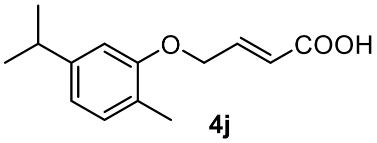

4j

(E)-4-(5-Isopropyl-2-methylphenoxy)but-2-enoic acid (**4j**): white solid, 95%. ^{1}H NMR (400 MHz, Chloroform-d) δ 7.00 (dd, J = 7.5, 0.9 Hz, 1H), 6.71 (dd, J = 7.6, 1.7 Hz, 1H), 6.63 (d, J = 1.7 Hz, 1H), 5.94 (d, J = 1.1 Hz, 1H), 5.75 (s, 1H), 4.39 – 4.24 (m, 2H), 2.89 – 2.75 (m, 1H), 2.38 (s, 3H), 1.21 (d, J = 7.0 Hz, 6H).

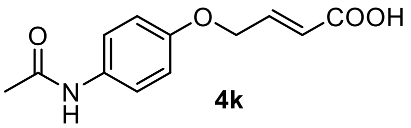

4k

(E)-4-(4-Acetamidophenoxy)but-2-enoic acid (**4k**): white solid, 87% yield，m.p. 210.3-211.4 °C. ^{1}H NMR (400 MHz, DMSO-d_6) δ 12.43 (s, 1H), 9.84 (s, 1H), 7.49 (d, J = 9.0 Hz, 2H), 7.01 – 6.82 (m, 3H), 6.03 (dt, J = 15.8, 2.0 Hz, 1H), 4.73 (dd, J = 4.3, 2.0 Hz, 2H), 2.01 (s, 3H).

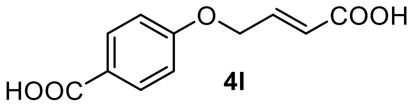

4l

(*E*)-4-((3-Carboxyallyl)oxy)benzoic acid (**4l**): white solid, quant. yield, m.p. 328.3-330.2 °C. ^{1}H NMR (400 MHz, DMSO-d_6) δ 7.90 (d, *J* = 8.7 Hz, 2H), 7.06 (d, *J* = 8.9 Hz, 2H), 6.95 (dt, *J* = 15.7, 4.3 Hz, 1H), 6.11 – 5.97 (m, 1H), 4.87 (dd, *J* = 4.3, 2.0 Hz, 2H);

To intermediate **1m** (1 equiv) in EtOH added dropwise of sodium hydroxide solution (2 N, 3 equiv) at room temperature. The reaction mixture was stirred at room temperature for 2 h. After the substrate was detected by TLC plate, it was concentrated under reduced pressure. Then added a little water and adjusted the pH to 3-4 with 2 *N* HCl at 0 °C. Filter and remove solids and place in oven. These solids are intermediate **4m**.

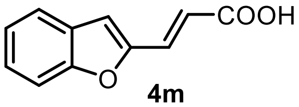

4m

(*E*)-3-(Benzofuran-2-yl)acrylic acid (**4m**)[5]: white solid, 98% yield, ^{1}H NMR (400 MHz, DMSO-d_6) δ 7.70 (d, *J* = 7.0 Hz, 1H), 7.59 (dd, *J* = 19.4, 12.0 Hz, 2H), 7.48 – 7.37 (m, 1H), 7.37 – 7.23 (m, 2H), 6.44 (d, *J* = 15.7 Hz, 1H).

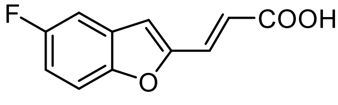

4n

(*E*)-3-(5-Fluorobenzofuran-2-yl)acrylic acid (**4n**): white solid, 74% yield, ^{1}H NMR (400 MHz, DMSO-d_6) δ 12.68 (s, 1H), 7.75 – 7.62 (m, 1H), 7.62 – 7.48 (m, 2H), 7.35 (s, 1H), 7.25 (t, *J* = 7.8 Hz, 1H), 6.44 (d, *J* = 15.8 Hz, 1H).

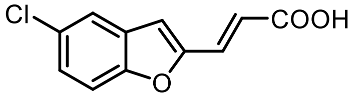

4o

(*E*)-3-(5-Chlorobenzofuran-2-yl)acrylic acid (**4o**): white solid, 97% yield, ^{1}H NMR (400 MHz, DMSO-d_6) δ 7.80 (d, *J* = 2.0 Hz, 1H), 7.72 – 7.55 (m, 2H), 7.43 (dd, *J* = 8.8, 2.2 Hz, 1H), 7.34 (s, 1H), 6.45 (d, *J* = 15.8 Hz, 1H).

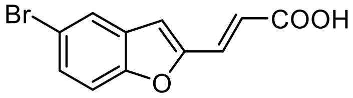

4p

(*E*)-3-(5-Bromobenzofuran-2-yl)acrylic acid (**4p**): white solid, 77% yield, ^{1}H NMR (400 MHz, DMSO-d_6) δ 12.71 (s, 1H), 7.94 (s, 1H), 7.69 – 7.47 (m, 3H), 7.33 (s, 1H), 6.45 (d, *J* = 15.8 Hz, 1H).

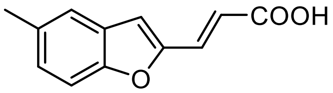

4q

(*E*)-3-(5-Methylbenzofuran-2-yl)acrylic acid (**4q**): white solid, quant. yield, ^{1}H NMR (400 MHz, DMSO-d_6) δ 12.60 (s, 1H), 7.65 – 7.42 (m, 3H), 7.35 – 7.16 (m, 2H), 6.40 (d, *J* = 15.7 Hz, 1H), 2.40 (s, 3H).

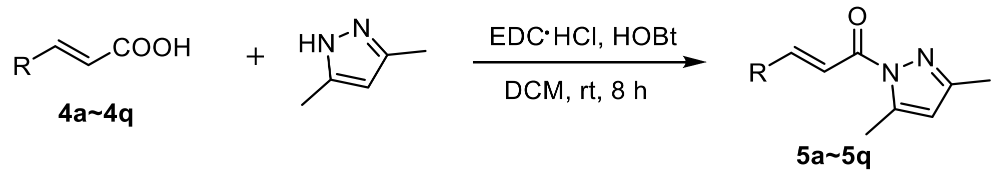

To **4** (5.6 mol), EDC·HCl (6.7 mol, 1.2 equiv) and HOBt (5.6 mol, 1 equiv) in dry DCM (35.0 mL) added 3,5-dimethylpyrazole (11.2 mol, 2 equiv) and the mixture was stirred at rt for 8 h. After the reaction completed (detected by TLC), and it was quenched by H_2O (15 mL), $NaHCO_3$ (5%) and saturated saline, then extracted with DCM. The combined

organic layers were dried over Na_2SO_4 and the solvents were evaporated under vacuum. The residue was purified by a flash column chromatography to afford pure substrates **5** as white solid.

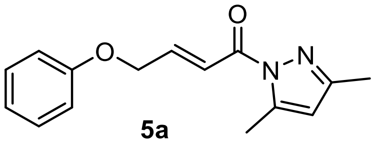
5a

(*E*)-1-(3,5-Dimethyl-1*H*-pyrazol-1-yl)-4-phenoxybut-2-en-1-one (**5a**): white solid, 51% yield, m.p. 82.9-83.6 °C. ^{1}H NMR (400 MHz, Chloroform-*d*) δ 7.65 (d, *J* = 15.8 Hz, 1H), 7.36 – 7.26 (m, 3H), 7.02 – 6.91 (m, 3H), 5.99 (s, 1H), 4.80 (s, 2H), 2.58 (s, 3H), 2.25 (s, 3H); ^{13}C NMR (101 MHz, Chloroform-*d*) δ 164.7, 158.3, 152.1, 144.4, 144.2, 129.5, 122.1, 121.3, 114.9, 111.6, 67.1, 14.5, 13.8. HRMS-ESI (m/z): [M+Na]$^+$ calcd. for $C_{15}H_{16}N_2O_2Na^+$: 279.1109, found: 279.1110.

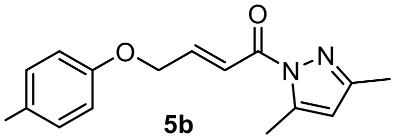
5b

(*E*)-1-(3,5-Dimethyl-1*H*-pyrazol-1-yl)-4-(4-fluorophenoxy)but-2-en-1-one (**5b**): white solid, 48% yield, m.p. 104.5-105.7 °C. ^{1}H NMR (400 MHz, Chloroform-*d*) δ 7.60 (dt, *J* = 15.8, 2.0 Hz, 1H), 7.26 – 7.19 (m, 1H), 6.98 – 6.91 (m, 2H), 6.89 – 6.82 (m, 2H), 5.96 (s, 1H), 4.72 (dd, *J* = 4.4, 2.0 Hz, 2H), 2.55 (d, *J* = 1.0 Hz, 3H), 2.22 (s, 3H); ^{13}C NMR (101 MHz, Chloroform-*d*) δ 164.6, 157.6 (d, *J* = 239.1 Hz), 154.3 (d, *J* = 2.2 Hz), 152.2, 144.5, 143.9, 122.2, 115.9 (d, *J* = 8.0 Hz), 115.9 (d, *J* = 23.0 Hz), 111.6, 67.8, 14.6, 13.8; ^{19}F NMR (376 MHz, Chloroform-*d*) δ -123.2. HRMS-ESI (m/z): [M+H]$^+$ calcd. for $C_{15}H_{15}FN_2O_2H^+$: 275.1190, found: 275.1188.

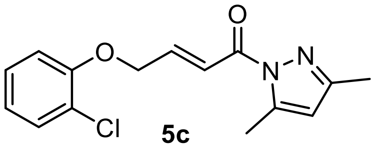
5c

(*E*)-4-(2-Chlorophenoxy)-1-(3,5-dimethyl-1*H*-pyrazol-1-yl)but-2-en-1-one (**5c**): white solid, 41% yield, m.p. 85.1-86.3°C. ^{1}H NMR (400 MHz, Chloroform-*d*) δ 7.65 (dt, *J* = 15.8, 1.9 Hz, 1H), 7.32 (dd, *J* = 8.1, 1.6 Hz, 1H), 7.26 – 7.18 (m, 1H), 7.16 – 7.10 (m, 1H), 6.90 – 6.79 (m, 2H), 5.92 (s, 1H), 4.80 (dd, *J* = 4.4, 2.0 Hz, 2H), 2.51 (d, *J* = 1.0 Hz, 3H), 2.18 (s, 3H); ^{13}C NMR (101 MHz, Chloroform-*d*) δ 164.6, 153.8, 152.2, 144.4, 143.3, 130.5, 127.7, 123.4, 122.7, 122.1, 114.0, 111.6, 68.3, 14.6, 13.9. HRMS-ESI (m/z): [M+H]$^+$ calcd. for $C_{15}H_{15}ClN_2O_2H^+$: 291.0895, found: 291.0890.

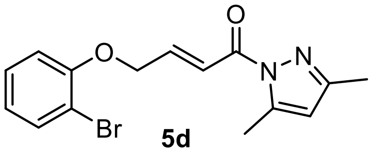
5d

(*E*)-4-(2-Bromophenoxy)-1-(3,5-dimethyl-1*H*-pyrazol-1-yl)but-2-en-1-one (**5d**): white solid, 57% yield, m.p. 75.8-76.9°C. ^{1}H NMR (400 MHz, Chloroform-*d*) δ 7.68 (dt, *J* = 15.8, 2.0 Hz, 1H), 7.49 (dd, *J* = 7.8, 1.6 Hz, 1H), 7.28 – 7.14 (m, 2H), 6.87 – 6.75 (m, 2H), 5.92 (s, 1H), 4.79 (dd, *J* = 4.4, 2.0 Hz, 2H), 2.51 (d, *J* = 1.0 Hz, 3H), 2.18 (s, 3H); ^{13}C NMR (101 MHz, Chloroform-*d*) δ 164.6, 154.7, 152.2, 144.4, 143.2, 133.6, 128.5, 122.7, 122.6, 113.8, 112.6, 111.6, 68.3, 14.6, 13.9. HRMS-ESI (m/z): [M+H]$^+$ calcd. for $C_{15}H_{15}BrN_2O_2H^+$: 335.0390, found: 335.0385.

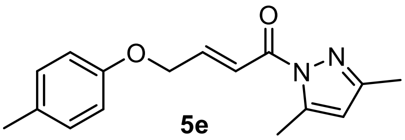
5e

(*E*)-1-(3,5-Dimethyl-1*H*-pyrazol-1-yl)-4-(*p*-tolyloxy)but-2-en-1-one (**5e**): white solid, 23% yield, m.p. 65.1-66.4 °C. ^{1}H NMR (400 MHz, Chloroform-*d*) δ 7.57 (dt, *J* = 15.8, 2.0 Hz, 1H), 7.24 – 7.16 (m, 1H), 7.07 – 6.97 (m, 2H), 6.83 – 6.74 (m, 2H), 5.92 (s, 1H), 4.70 (dd, *J* = 4.4, 2.0 Hz, 2H), 2.51 (s, 3H), 2.22 (s, 3H), 2.19 (s, 3H); ^{13}C NMR (101 MHz, Chloroform-*d*) δ 164.7, 156.1, 152.1, 144.5, 144.4, 130.6, 130.0, 121.9, 114.7, 111.6, 67.3, 20.5, 14.6, 13.8. HRMS-ESI (m/z): [M+H]$^+$ calcd. for $C_{16}H_{18}N_2O_2H^+$: 271.1441, found: 271.1440.

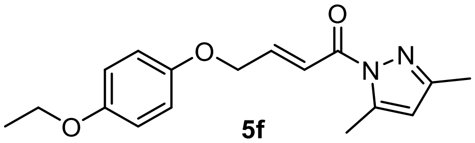

5f

(*E*)-1-(3,5-Dimethyl-1*H*-pyrazol-1-yl)-4-(4-ethoxyphenoxy)but-2-en-1-one (**5f**): white solid, 59% yield, m.p. 77.4-78.5 °C. ^{1}H NMR (400 MHz, Chloroform-*d*) δ 7.66 (dt, *J* = 15.8, 2.0 Hz, 1H), 7.31 (dt, *J* = 15.8, 4.3 Hz, 1H), 6.96 – 6.82 (m, 4H), 6.01 (s, 1H), 4.77 (dd, *J* = 4.4, 2.0 Hz, 2H), 4.00 (q, *J* = 7.0 Hz, 2H), 2.61 (d, *J* = 1.0 Hz, 3H), 2.28 (s, 3H), 1.41 (t, *J* = 7.0 Hz, 3H); ^{13}C NMR (101 MHz, Acetone-d_6) δ 165.1, 154.6, 153.3, 152.5, 145.9, 144.8, 122.0, 116.7, 116.2, 112.3, 68.5, 64.4, 15.2, 14.5, 13.8. HRMS-ESI (m/z): $[M+H]^+$ calcd. for $C_{17}H_{20}N_2O_3H^+$: 301.1547, found: 301.1543.

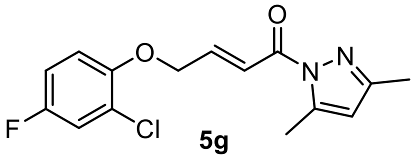

5g

(*E*)-4-(2-Chloro-4-fluorophenoxy)-1-(3,5-dimethyl-1*H*-pyrazol-1-yl)but-2-en-1-one (**5g**): white solid, 40% yield, m.p. 91.6-92.4°C. ^{1}H NMR (400 MHz, Chloroform-*d*) δ 7.63 (dt, *J* = 15.8, 1.9 Hz, 1H), 7.25 – 7.15 (m, 1H), 7.08 (dd, *J* = 8.0, 2.8 Hz, 1H), 6.90 – 6.77 (m, 2H), 5.93 (s, 1H), 4.76 (dd, *J* = 4.5, 2.0 Hz, 2H), 2.51 (d, *J* = 1.0 Hz, 3H), 2.18 (s, 3H); ^{13}C NMR (101 MHz, Chloroform-*d*) δ 164.5, 157.0 (d, *J* = 242.9 Hz), 152.3, 150.4 (d, *J* = 2.9 Hz), 144.4, 143.0, 124.3 (d, *J* = 10.7 Hz), 122.7, 117.8 (d, *J* = 25.8 Hz), 115.1 (d, *J* = 8.8 Hz), 114.1 (d, *J* = 22.6 Hz), 111.6, 69.1, 14.5, 13.8; ^{19}F NMR (376 MHz, Chloroform-*d*) δ -120.5. HRMS-ESI (m/z): $[M+H]^+$ calcd. for $C_{15}H_{14}ClFN_2O_2H^+$: 309.0801, found: 309.0798.

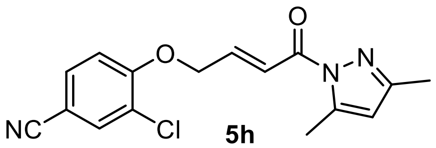

5h

(*E*)-3-Chloro-4-((4-(3,5-dimethyl-1*H*-pyrazol-1-yl)-4-oxobut-2-en-1-yl)oxy)benzonitrile (**5h**): white solid, 24% yield, m.p. 145.2-146.9°C. ^{1}H NMR (400 MHz, Acetone-d_6) δ 7.90 (s, 1H), 7.80 – 7.67 (m, 2H), 7.39 (d, *J* = 8.6 Hz, 1H), 7.29 (dt, *J* = 15.9, 4.3 Hz, 1H), 6.15 (s, 1H), 5.23 – 5.10 (m, 2H), 2.55 (s, 3H), 2.20 (s, 3H); ^{13}C NMR (101 MHz, Acetone-d_6) δ 164.9, 158.2, 152.7, 144.9, 143.3, 134.5, 133.8, 124.2, 123.1, 118.3, 115.3, 112.4, 106.2, 69.2, 14.5, 13.8. HRMS-ESI (m/z): $[M+H]^+$ calcd. for $C_{16}H_{14}ClN_3O_2H^+$: 316.0847, found: 316.0845.

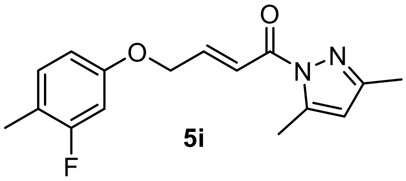

5i

(*E*)-1-(3,5-Dimethyl-1*H*-pyrazol-1-yl)-4-(3-fluoro-4-methylphenoxy)but-2-en-1-one (**5i**): white solid, 38% yield, m.p. 91.7-93.3 °C. ^{1}H NMR (400 MHz, Chloroform-*d*) δ 7.56 (dt, *J* = 15.8, 2.0 Hz, 1H), 7.24 – 7.12 (m, 1H), 7.05 – 6.90 (m, 1H), 6.62 – 6.49 (m, 2H), 5.92 (s, 1H), 4.68 (dd, *J* = 4.4, 2.0 Hz, 2H), 2.51 (d, *J* = 1.0 Hz, 3H), 2.18 (s, 3H), 2.12 (d, *J* = 1.8 Hz, 3H); ^{13}C NMR (101 MHz, Chloroform-*d*) δ 164.6, 161.6 (d, *J* = 244.9 Hz), 157.4 (d, *J* = 10.4 Hz), 152.2, 144.5, 143.7, 131.5 (d, *J* = 7.2 Hz), 122.2, 117.3 (d, *J* = 17.6 Hz), 111.6, 110.2 (d, *J* = 3.1 Hz), 102.5 (d, *J* = 25.8 Hz), 67.4, 14.6, 13.8, 13.8 (d, *J* = 3.1 Hz); ^{19}F NMR (376 MHz, Chloroform-*d*) δ -115.0. HRMS-ESI (m/z): $[M+H]^+$ calcd. for $C_{16}H_{17}FN_2O_2H^+$: 289.1347, found: 289.1348.

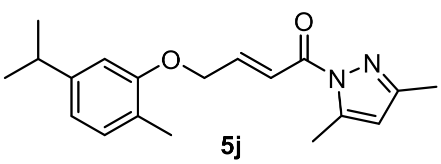

5j

(*E*)-1-(3,5-Dimethyl-1*H*-pyrazol-1-yl)-4-(5-isopropyl-2-methylphenoxy)but-2-en-1-one (**5j**): white solid, 38% yield, m.p. 50.9-52.0 °C. ^{1}H NMR (400 MHz, Chloroform-*d*) δ 7.70 (dt, *J* = 15.8, 2.0 Hz, 1H), 7.32 (dt, *J* = 15.7, 4.4 Hz, 1H), 7.09 (dd, *J* = 7.6, 0.8 Hz, 1H), 6.77 (dd, *J* = 7.5, 1.6 Hz, 1H), 6.69 (d, *J* = 1.6 Hz, 1H), 6.00 (s, 1H), 4.81 (dd, *J* = 4.5, 2.0 Hz, 2H),

2.59 (d, *J* = 1.0 Hz, 3H), 2.26 (d, *J* = 5.2 Hz, 6H), 1.24 (d, *J* = 6.9 Hz, 6H); ^{13}C NMR (101 MHz, Acetone-*d*$_6$) δ 165.2, 157.3, 152.5, 148.8, 145.7, 144.8, 131.4, 124.7, 122.1, 119.5, 112.2, 111.0, 68.0, 34.8, 24.4, 16.0, 14.5, 13.8. HRMS-ESI (m/z): $[M+H]^+$ calcd. for $C_{19}H_{24}FN_2O_2H^+$: 313.1911, found: 313.1902.

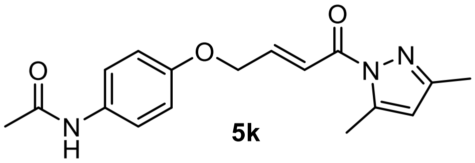

5k

(*E*)-N-(4-((4-(3,5-Dimethyl-1*H*-pyrazol-1-yl)-4-oxobut-2-en-1-yl)oxy) phenyl)acetamide (**5k**): white solid, 24% yield, m.p. 143.5-144.2°C. ^{1}H NMR (400 MHz, Chloroform-*d*) δ 7.56 (dt, *J* = 15.7, 2.0 Hz, 1H), 7.38 – 7.30 (m, 2H), 7.25 – 7.16 (m, 2H), 7.11 (s, 1H), 6.88 – 6.80 (m, 2H), 5.93 (s, 1H), 4.70 (dd, *J* = 4.3, 2.0 Hz, 2H), 2.51 (d, *J* = 1.0 Hz, 3H), 2.19 (s, 3H), 2.08 (s, 3H); ^{13}C NMR (101 MHz, Chloroform-*d*) δ 168.1, 164.6, 155.0, 152.2, 144.5, 144.1, 131.6, 122.1, 121.8, 115.2, 111.6, 67.4, 24.4, 14.6, 13.8. HRMS-ESI (m/z): $[M+H]^+$ calcd. for $C_{17}H_{19}N_3O_3H^+$: 314.1499, found: 314.1497.

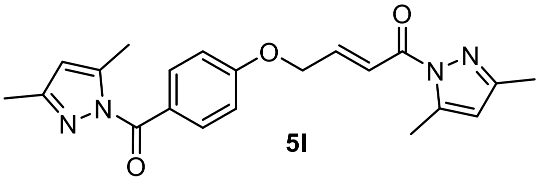

5l

(*E*)-1-(3,5-Dimethyl-1*H*-pyrazol-1-yl)-4-(4-(3,5-dimethyl-1*H*-pyrazole-1-carbonyl)phenoxy)but-2-en-1-one (**5l**): white solid, 38% yield, m.p. 131.3-132.2°C. ^{1}H NMR (400 MHz, Chloroform-*d*) δ 8.06 – 7.89 (m, 2H), 7.58 (dt, *J* = 15.8, 2.0 Hz, 1H), 7.25 – 7.17 (m, 1H), 6.94 (d, *J* = 8.9 Hz, 2H), 5.95 (dd, *J* = 16.7, 1.1 Hz, 2H), 4.80 (dd, *J* = 4.3, 2.0 Hz, 2H), 2.52 (d, *J* = 8.6 Hz, 6H), 2.19 (d, *J* = 2.5 Hz, 6H); ^{13}C NMR (101 MHz, Acetone-*d*$_6$) δ 167.8, 165.0, 162.6, 152.7, 152.1, 145.6, 144.9, 144.7, 134.8, 127.1, 122.5, 114.7, 112.3, 111.4, 68.1, 14.5, 14.2, 13.8, 13.7. HRMS-ESI (m/z): $[M+H]^+$ calcd. for $C_{21}H_{22}N_4O_3H^+$: 379.1765, found: 379.1759.

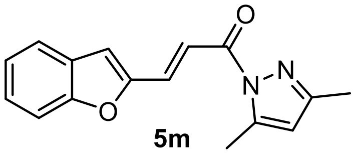

5m

(*E*)-3-(Benzofuran-2-yl)-1-(3,5-dimethyl-1*H*-pyrazol-1-yl)prop-2-en-1-one (**5m**): white solid, 71% yield, m.p. 152.3-153.1°C. ^{1}H NMR (400 MHz, Chloroform-*d*) δ 8.03 (d, *J* = 15.6 Hz, 1H), 7.75 (d, *J* = 15.6 Hz, 1H), 7.60 (d, *J* = 7.5 Hz, 1H), 7.52 (d, *J* = 8.1 Hz, 1H), 7.37 (t, *J* = 7.4 Hz, 1H), 7.24 (d, *J* = 7.1 Hz, 1H), 7.03 (s, 1H), 6.02 (s, 1H), 2.62 (s, 3H), 2.31 (s, 3H); ^{13}C NMR (101 MHz, Chloroform-*d*) δ 165.1, 155.8, 153.0, 152.1, 144.4, 132.2, 128.5, 126.7, 123.3, 121.8, 118.7, 112.0, 111.6, 14.6, 13.9. HRMS-ESI (m/z): $[M+Na]^+$ calcd. for $C_{16}H_{14}N_2O_2Na^+$: 289.0953, found: 289.0954.

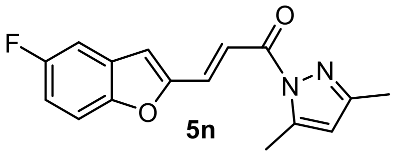

5n

(*E*)-1-(3,5-Dimethyl-1*H*-pyrazol-1-yl)-3-(5-fluorobenzofuran-2-yl)prop-2-en-1-one (**5n**): white solid, 69% yield, m.p. 158.3-159.1°C. ^{1}H NMR (400 MHz, Chloroform-*d*) δ 8.03 (d, *J* = 15.6 Hz, 1H), 7.72 (d, *J* = 15.7 Hz, 1H), 7.52 – 7.40 (m, 1H), 7.24 (s, 1H), 7.10 (t, *J* = 7.7 Hz, 1H), 7.00 (s, 1H), 6.03 (s, 1H), 2.63 (s, 3H), 2.31 (s, 3H); ^{13}C NMR (101 MHz, Chloroform-*d*) δ 164.9, 159.4 (d, *J* = 238.9 Hz), 154.6, 144.4, 131.8, 129.2, 129.1, 119.5, 114.5 (d, *J* = 26.6 Hz), 112.3 (d, *J* = 9.4 Hz), 111.7, 111.6, 107.2, 106.9, 14.6, 13.9; ^{19}F NMR (376 MHz, Chloroform-*d*) δ -120.0. HRMS-ESI (m/z): $[M+Na]^+$ calcd. for $C_{16}H_{13}N_2O_2FNa^+$: 307.0859, found: 307.0859.

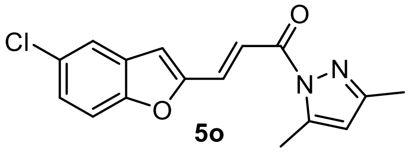

5o

(*E*)-3-(5-Chlorobenzofuran-2-yl)-1-(3,5-dimethyl-1*H*-pyrazol-1-yl)prop-2-en-1-one (**5o**): white solid, 59% yield, m.p. 171.3-172.9°C. ^{1}H NMR (400 MHz, Chloroform-*d*) δ 8.04 (d, *J* = 15.7 Hz, 1H), 7.71 (d, *J* = 15.7 Hz, 1H), 7.57 (d, *J* = 1.7 Hz, 1H), 7.45 (d, *J* = 8.7 Hz, 1H), 7.33 (dd, *J* = 8.8, 1.8 Hz, 1H), 6.03 (s, 1H), 2.63 (s, 3H), 2.31 (s, 3H); ^{13}C NMR (101 MHz, Acetone-*d*$_6$) δ 165.1, 155.3, 155.0, 152.8, 144.9, 132.2, 131.0, 129.5, 127.7, 122.4, 120.4, 113.7, 112.7, 112.5, 14.5, 13.8. HRMS-ESI (m/z): $[M+Na]^+$ calcd. for $C_{16}H_{13}N_2O_2ClNa^+$: 323.0563, found: 323.0562.

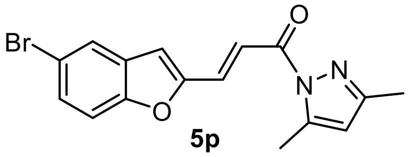

5p

(*E*)-3-(5-Bromobenzofuran-2-yl)-1-(3,5-dimethyl-1*H*-pyrazol-1-yl)prop-2-en-1-one (**5p**): white solid, 82% yield, m.p. 181.2-182.1°C. ^{1}H NMR (400 MHz, Chloroform-*d*) δ 8.04 (d, *J* = 15.7 Hz, 1H), 7.71 (d, *J* = 15.2 Hz, 2H), 7.54 – 7.35 (m, 2H), 6.96 (s, 1H), 6.03 (s, 1H), 2.62 (s, 3H), 2.31 (s, 3H); ^{13}C NMR (101 MHz, Chloroform-*d*) δ 164.9, 154.4, 154.1, 152.3, 144.4, 131.6, 130.4, 129.5, 124.3, 119.8, 116.4, 113.0, 111.7, 110.8, 14.6, 13.9. HRMS-ESI (m/z): $[M+Na]^+$ calcd. for $C_{16}H_{13}N_2O_2BrNa^+$: 367.0058, found: 367.0052.

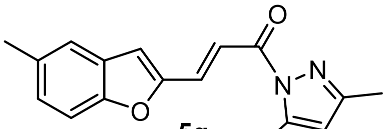

5q

(*E*)-1-(3,5-Dimethyl-1*H*-pyrazol-1-yl)-3-(5-methylbenzofuran-2-yl)prop-2-en-1-one (**5q**): white solid, 63% yield, m.p. 125.5-126.2°C. ^{1}H NMR (400 MHz, Chloroform-*d*) δ 8.00 (d, *J* = 15.6 Hz, 1H), 7.73 (d, *J* = 15.6 Hz, 1H), 7.46 – 7.33 (m, 2H), 7.18 (d, *J* = 7.8 Hz, 1H), 6.96 (s, 1H), 6.02 (s, 1H), 2.62 (s, 3H), 2.44 (s, 3H), 2.31 (s, 3H); ^{13}C NMR (101 MHz, Chloroform-*d*) δ 165.2, 154.3, 153.1, 152.1, 144.3, 132.9, 132.4, 128.6, 128.1, 121.5, 118.3, 111.8, 111.5, 111.1, 21.3, 14.6, 13.9. HRMS-ESI (m/z): $[M+Na]^+$ calcd. for $C_{17}H_{16}N_2O_2Na^+$: 303.1109, found: 303.1111.

2.2 General procedure for CSB-1 catalyzed Michael additions of 2 and 5.

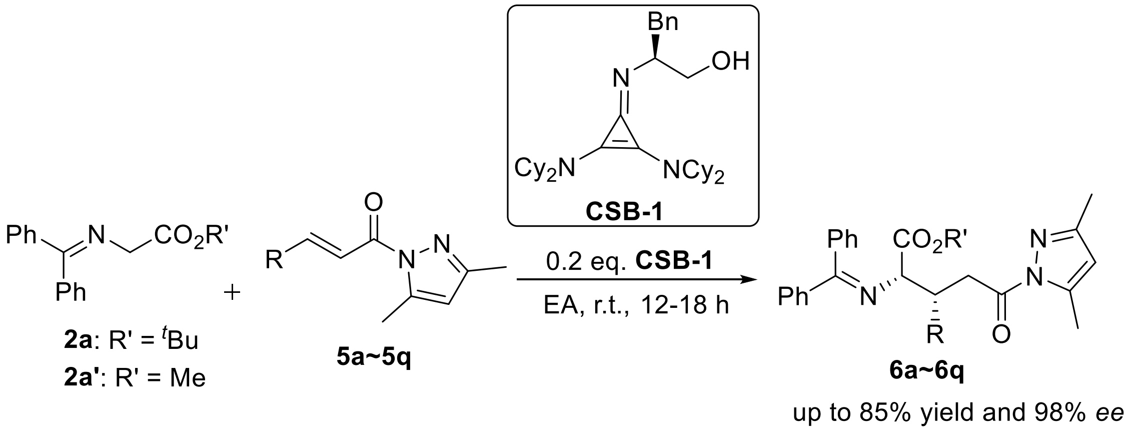

To **2** (1 mmol, 1 equiv) and **5** (1.0 mmol) in EA (10.0 mL) added **CSB-1** (0.2 mmol) and the mixture was stirred at rt for 12–18 h. After the reaction completed (detected by TLC), the solvent was removed by a rotary evaporator under reduced pressure. The residue was purified by a flash column chromatography (petroether/EtOAc/Et_3N 40:1:0.01~20:1:0.01, v/v) to afford pure **6** as a colorless sticky oil.

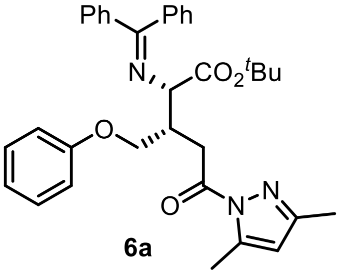

6a

tert-Butyl (2*S*,3*S*)-5-(3,5-dimethyl-1*H*-pyrazol-1-yl)-2-((diphenylmethylene)amino)-5-oxo-3-(phenoxymethyl)pentanoate

(**6a**): colorless sticky oil, 85% yield, 98% *ee*, $[\alpha]_D^{20}$ = +4.9 (c = 0.10, CH_2Cl_2). ^{1}H NMR (400 MHz, Acetone-*d*6) δ 7.61 (d, *J* = 7.1 Hz, 2H), 7.52 – 7.30 (m, 6H), 7.24 (t, *J* = 8.0 Hz, 2H), 7.18 – 7.11 (m, 2H), 6.94 – 6.81 (m, 3H), 6.03 (s, 1H), 4.39 (d, *J* = 4.1 Hz, 1H), 4.13 (dd, *J* = 9.4, 5.1 Hz, 1H), 3.99 (t, *J* = 8.8 Hz, 1H), 3.64 (dd, *J* = 17.1, 4.6 Hz, 1H), 3.50 – 3.30 (m, 2H), 2.49 (s, 3H), 2.17 (s, 3H), 1.41 (s, 9H); ^{13}C NMR (101 MHz, Acetone-*d*6) δ 173.5, 172.3, 170.8, 159.7, 152.2, 144.5, 140.4, 137.4, 131.2, 130.2, 129.5, 129.4, 129.3, 128.8, 128.6, 121.5, 115.3, 111.7, 81.8, 68.6, 66.3, 39.3, 34.5, 28.2, 14.6, 13.8. HRMS-ESI (m/z): $[M+H]^+$ calcd. for $C_{34}H_{38}N_3O_4^+$: 552.2862, found: 552.2862. HPLC test condition: Chiralpak OD-H, Hexane/2-Propanol = 90:10, flow rate 0.8 mL/min, test wavelength 254 nm, t_R (major) = 5.286 min and 6.271 min, t_R (minor) = 6.873 min, dr > 20:1.

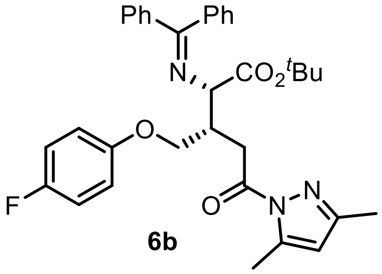

6b

tert-Butyl (2*S*,3*S*)-5-(3,5-dimethyl-1*H*-pyrazol-1-yl)-2-((diphenylmethylene)amino)-3-((4-fluorophenoxy)methyl)-5-oxo-pentanoate (**6b**): colorless sticky oil, 68% yield, 98% *ee*, $[\alpha]_D^{20}$ = -68.0 (c = 0.10, CH_2Cl_2). ^{1}H NMR (400 MHz, Acetone-*d*6) δ 7.63 – 7.56 (m, 2H), 7.52 – 7.45 (m, 3H), 7.45 – 7.39 (m, 1H), 7.39 – 7.31 (m, 2H), 7.19 – 7.10 (m, 2H), 7.05 – 6.97 (m, 2H), 6.88 – 6.81 (m, 2H), 6.03 (s, 1H), 4.36 (d, *J* = 4.2 Hz, 1H), 4.11 (dd, *J* = 9.5, 5.2 Hz, 1H), 4.00 – 3.94 (m, 1H), 3.63 (dd, *J* = 17.1, 4.7 Hz, 1H), 3.43 (dd, *J* = 17.1, 8.0 Hz, 1H), 3.38 – 3.29 (m, 1H), 2.49 (d, *J* = 1.0 Hz, 3H), 2.17 (s, 3H), 1.41 (s, 9H); ^{13}C NMR (101 MHz, Acetone-*d*6) δ 173.5, 172.3, 170.8, 158.0 (d, *J* = 236.2 Hz), 155.9 (d, *J* = 1.4 Hz), 152.2, 144.5, 140.4, 137.4, 131.3, 129.5, 129.3 (d, *J* = 8.8 Hz), 128.7 (d, *J* = 25.5 Hz), 116.5, 116.5, 116.4, 116.3, 111.8, 81.8, 69.3, 66.3, 39.3, 34.4, 28.2, 14.6, 13.8; ^{19}F NMR (376 MHz, Acetone-*d*6) δ 51.8. HRMS-ESI (m/z): $[M+H]^+$ calcd. for $C_{34}H_{37}FN_3O_4^+$: 570.2763, found: 570.2755. HPLC test condition: Chiralpak OD-H, Hexane/2-Propanol = 90:10, flow rate 0.8 mL/min, test wavelength 254 nm, t_R (major) = 5.514 min and 6.625 min, t_R (minor) = 9.637 min, dr > 20:1.

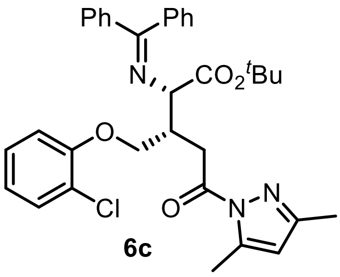

6c

tert-Butyl (2*S*,3*S*)-3-((2-chlorophenoxy)methyl)-5-(3,5-dimethyl-1*H*-pyrazol-1-yl)-2-((diphenylmethylene)amino)-5-oxo-pentanoate (**6c**): colorless sticky oil, 50% yield, 98% *ee*, $[\alpha]_D^{20}$ = -73.5 (c = 0.11, CH_2Cl_2). ^{1}H NMR (400 MHz, Acetone-*d*6) δ 7.64 – 7.56 (m, 2H), 7.49 – 7.39 (m, 4H), 7.38 – 7.29 (m, 3H), 7.27 – 7.16 (m, 3H), 7.15 – 7.09 (m, 1H), 6.95 – 6.86 (m, 1H), 6.00 (s, 1H), 4.52 – 4.47 (m, 1H), 4.23 – 4.13 (m, 2H), 3.70 – 3.59 (m, 1H), 3.49 – 3.36 (m, 2H), 2.48 (s, 3H), 2.16 (s, 3H), 1.39 (s, 9H); ^{13}C NMR (101 MHz, Acetone-*d*6) δ 173.4, 172.4, 170.8, 155.2, 152.2, 144.5, 140.5, 137.2, 131.3, 130.8, 129.4, 129.4, 129.3, 128.9, 128.8, 128.6, 123.2, 122.3, 114.4, 111.7, 81.8, 69.8, 66.3, 39.4, 34.5, 28.2, 14.7, 13.8. HRMS-ESI (m/z): $[M+H]^+$ calcd. for $C_{34}H_{37}ClN_3O_4^+$: 586.2467, found: 586.2457. HPLC test condition: Chiralpak OD-H, Hexane/2-Propanol = 90:10, flow rate 0.8 mL/min, test wavelength 254 nm, t_R (minor) = 12.714 min, t_R (major) = 14.896 min, dr > 20:1.

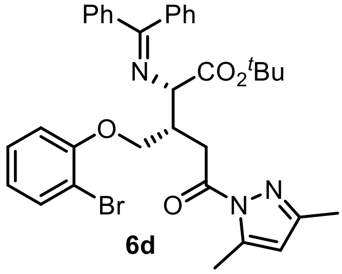

6d

tert-Butyl (2*S*,3S)-3-((2-bromophenoxy)methyl)-5-(3,5-dimethyl-1*H*-pyrazol-1-yl)-2-((diphenylmethylene)amino)-5-oxo-pentanoate (**6d**): colorless sticky oil, 79% yield, 95% *ee*, $[\alpha]_D^{20}$ = -80.0 (c = 0.10, CH_2Cl_2). ^{1}H NMR (400 MHz, Acetone-d_6) δ 7.63 – 7.55 (m, 2H), 7.53 – 7.38 (m, 5H), 7.37 – 7.25 (m, 3H), 7.24 – 7.17 (m, 2H), 7.09 (dd, *J* = 8.3, 1.5 Hz, 1H), 6.85 (td, *J* = 7.6, 1.4 Hz, 1H), 5.99 (s, 1H), 4.53 (d, *J* = 4.8 Hz, 1H), 4.19 (d, *J* = 5.4 Hz, 2H), 3.71 – 3.60 (m, 1H), 3.47 – 3.37 (m, 2H), 2.48 (d, *J* = 1.1 Hz, 3H), 2.16 (s, 3H), 1.38 (s, 9H); ^{13}C NMR (101 MHz, Acetone-d_6) δ 173.4, 172.4, 170.8, 156.0, 152.2, 144.5, 140.5, 137.3, 133.9, 131.3, 129.6, 129.4, 129.4, 128.8, 128.7, 122.8, 114.3, 112.6, 111.7, 81.8, 70.0, 66.4, 39.4, 34.6, 28.2, 14.7, 13.8. HRMS-ESI (m/z): $[M+H]^+$ calcd. for $C_{34}H_{37}BrN_3O_4^+$: 630.1962, found: 630.1954. HPLC test condition: Chiralpak OD-H, Hexane/2-Propanol = 90:10, flow rate 0.8 mL/min, test wavelength 254 nm, t_R (minor) = 5.199 min and 6.427 min, t_R (major) = 5.940 min and 6.726 min, dr > 20:1.

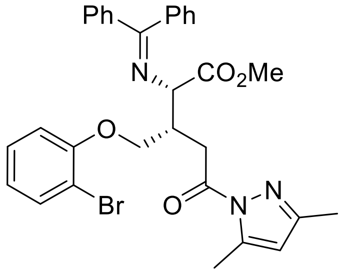

6d'

Methyl (2*S*,3*S*)-3-((2-bromophenoxy)methyl)-5-(3,5-dimethyl-1*H*-pyrazol-1-yl)-2-((diphenylmethylene)amino)-5-oxo-pentanoate (**6d'**): colorless sticky oil, 71% yield, 93% *ee*, $[\alpha]_D^{20}$ = -78.2 (c = 0.11, CH_2Cl_2). ^{1}H NMR (400 MHz, Acetone-d_6) δ 7.6 – 7.6 (m, 2H), 7.5 (dd, *J* = 7.9, 1.6 Hz, 1H), 7.5 – 7.4 (m, 4H), 7.4 – 7.3 (m, 2H), 7.3 – 7.2 (m, 1H), 7.2 – 7.1 (m, 2H), 7.1 (dd, *J* = 8.3, 1.4 Hz, 1H), 6.9 (td, *J* = 7.6, 1.4 Hz, 1H), 6.0 (s, 1H), 4.6 (d, *J* = 4.4 Hz, 1H), 4.2 (dd, *J* = 9.6, 4.3 Hz, 1H), 4.1 (dd, *J* = 9.6, 7.0 Hz, 1H), 3.7 – 3.7 (m, 1H), 3.6 (s, 3H), 3.5 – 3.4 (m, 2H), 2.5 (s, 3H), 2.2 (s, 3H); ^{13}C NMR (101 MHz, Acetone-d_6) δ 173.3, 172.8, 172.1, 155.9, 152.2, 144.5, 140.3, 137.0, 133.9, 131.4, 129.6, 129.5, 129.5, 128.8, 128.5, 122.7, 114.1, 112.5, 111.7, 69.6, 65.4, 52.4, 39.4, 34.4, 14.7, 13.8. HRMS-ESI (m/z): $[M+H]^+$ calcd. for $C_{31}H_{31}BrN_3O_4^+$:588.1492, found: 588.1486. HPLC test condition: Chiralpak OD-H, Hexane/2-Propanol = 92:8, flow rate 0.8 mL/min, test wavelength 254 nm, t_R (minor) = 18.219 min, t_R (major) = 17.155 min, dr > 20:1.

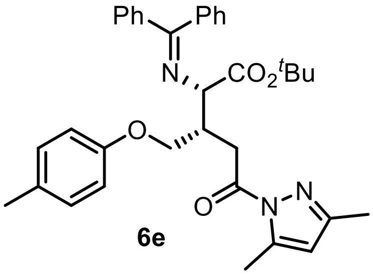

6e

tert-Butyl (2*S*,3*S*)-5-(3,5-dimethyl-1*H*-pyrazol-1-yl)-2-((diphenylmethylene)amino)-5-oxo-3-((p-tolyloxy)methyl) pentanoate (**6e**): colorless sticky oil, 76% yield, 96% *ee*, $[\alpha]_D^{20}$ = -44.8 (c = 0.11, CH_2Cl_2). ^{1}H NMR (400 MHz, Acetone-d_6) δ 7.65 – 7.58 (m, 2H), 7.52 – 7.38 (m, 4H), 7.38 – 7.31 (m, 2H), 7.19 – 7.11 (m, 2H), 7.08 – 7.00 (m, 2H), 6.78 – 6.70 (m, 2H), 6.03 (s, 1H), 4.38 (d, *J* = 4.2 Hz, 1H), 4.09 (dd, *J* = 9.5, 5.1 Hz, 1H), 3.99 – 3.91 (m, 1H), 3.64 (dd, *J* = 17.1, 4.7 Hz, 1H), 3.43 (dd, *J* = 17.1, 8.0 Hz, 1H), 3.39 – 3.30 (m, 1H), 2.49 (d, *J* = 1.0 Hz, 3H), 2.22 (s, 3H), 2.17 (s, 3H), 1.41 (s, 9H); ^{13}C NMR (101 MHz, Acetone-d_6) δ 173.5, 172.2, 170.8, 157.6, 152.2, 144.4, 140.4, 137.4, 131.2, 130.6, 130.4, 129.5, 129.4,

129.3, 128.8, 128.6, 115.1, 111.8, 81.7, 68.7, 66.3, 39.3, 34.5, 28.2, 20.5, 14.7, 13.8. HRMS-ESI (m/z): $[M+H]^+$ calcd. for $C_{35}H_{40}N_3O_4^+$: 566.3013, found: 566.3005. HPLC test condition: Chiralpak OD-H, Hexane/2-Propanol = 90:10, flow rate 0.8 mL/min, test wavelength 254 nm, t_R (major) = 5.089 min and 6.757 min, t_R (minor) = 6.221 min and 9.777 min, dr > 20:1.

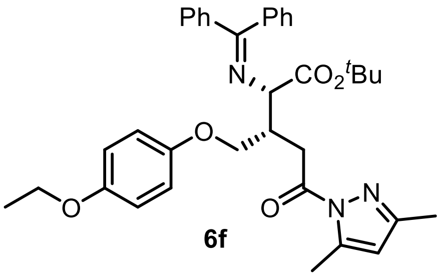

6f

tert-Butyl (2*S*,3*S*)-5-(3,5-dimethyl-1*H*-pyrazol-1-yl)-2-((diphenylmethylene)amino)-3-((4-ethoxyphenoxy)methyl)-5-oxo-pentanoate (**6f**): colorless sticky oil, 71% yield, 95% *ee*, $[\alpha]_D^{20}$ = -35.3° (c = 0.11, CH_2Cl_2). ^{1}H NMR (400 MHz, Acetone-d_6) δ 7.65 – 7.58 (m, 2H), 7.51 – 7.45 (m, 3H), 7.44 – 7.38 (m, 1H), 7.38 – 7.30 (m, 2H), 7.16 (dd, *J* = 6.6, 3.0 Hz, 2H), 6.84 – 6.73 (m, 4H), 6.02 (s, 1H), 4.39 (d, *J* = 4.2 Hz, 1H), 4.06 (dd, *J* = 9.4, 5.2 Hz, 1H), 3.98 – 3.89 (m, 3H), 3.64 (dd, *J* = 17.1, 4.7 Hz, 1H), 3.43 (dd, *J* = 17.1, 8.0 Hz, 1H), 3.39 – 3.30 (m, 1H), 2.49 (s, 3H), 2.18 (s, 3H), 1.41 (s, 9H), 1.31 (t, *J* = 7.0 Hz, 3H); ^{13}C NMR (101 MHz, Acetone-d_6) δ 173.5, 172.2, 170.9, 154.2, 153.6, 152.2, 144.4, 140.4, 137.4, 131.3, 129.5, 129.4, 129.3, 128.9, 128.6, 116.1, 116.0, 111.8, 81.7, 69.2, 66.3, 64.3, 39.4, 34.5, 28.2, 15.3, 14.7, 13.9. HRMS-ESI (m/z): $[M+H]^+$ calcd. for $C_{36}H_{42}N_3O_5^+$: 596.3119, found: 596.3112. HPLC test condition: Chiralpak OD-H, Hexane/2-Propanol = 80:20, flow rate 0.8 mL/min, test wavelength 254 nm, t_R (major) = 5.493 min and 8.428 min, t_R (minor) = 6.414 min and 8.428 min, dr > 20:1.

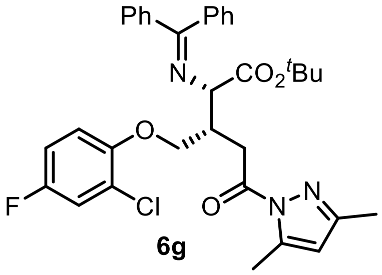

6g

tert-Butyl (2*S*,3*S*)-3-((2-chloro-4-fluorophenoxy)methyl)-5-(3,5-dimethyl-1*H*-pyrazol-1-yl)-2-((diphenylmethylene)amino)-5-oxopentanoate (**6g**): colorless sticky oil, 70% yield, 97% *ee*, $[\alpha]_D^{20}$ = -103.8 (c = 0.11, CH_2Cl_2). ^{1}H NMR (400 MHz, Acetone-d_6) δ 7.62 – 7.55 (m, 2H), 7.50 – 7.39 (m, 4H), 7.38 – 7.30 (m, 2H), 7.24 – 7.17 (m, 3H), 7.14 (dd, *J* = 9.1, 4.9 Hz, 1H), 7.07 – 7.00 (m, 1H), 6.00 (s, 1H), 4.47 (d, *J* = 4.7 Hz, 1H), 4.21 – 4.11 (m, 2H), 3.69 – 3.59 (m, 1H), 3.47 – 3.36 (m, 2H), 2.47 (s, 3H), 2.16 (s, 3H), 1.39 (s, 9H); ^{13}C NMR (101 MHz, Acetone-d_6) δ 173.4, 172.4, 170.7, 157.2 (d, *J* = 239.9 Hz), 152.2, 151.9 (d, *J* = 2.9 Hz), 144.5, 140.4, 137.2, 131.3, 129.5, 129.4, 129.3, 128.7 (d, *J* = 20.6 Hz), 123.8 (d, *J* = 10.8 Hz), 117.9 (d, *J* = 26.4 Hz), 115.2, 115.1 (d, *J* = 3.3 Hz), 114.9, 111.7, 81.8, 70.5, 66.3, 39.4, 34.5, 28.1, 14.6, 13.8; ^{19}F NMR (376 MHz, Acetone-d_6) δ 54.0. HRMS-ESI (m/z): $[M+H]^+$ calcd. for $C_{34}H_{35}ClFN_3O_4^+$: 604.2373, found: 604.2361. HPLC test condition: Chiralpak OD-H, Hexane/2-Propanol = 96:4, flow rate 0.8 mL/min, test wavelength 254 nm, t_R (minor) = 7.601 min, t_R (major) = 10.944 min, dr > 20:1.

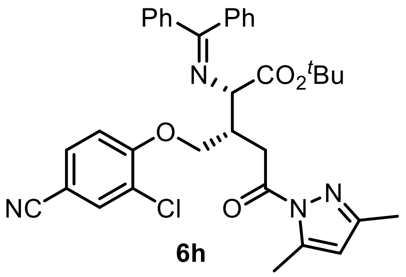

6h

tert-Butyl (2*S*,3*S*)-3-((2-chloro-4-cyanophenoxy)methyl)-5-(3,5-dimethyl-1*H*-pyrazol-1-yl)-2-((diphenylmethylene)amino) -5-oxopentanoate (**6h**): white solid, 73% yield, m.p. 62.0-62.9 °C, 97% *ee*, $[\alpha]_D^{20}$ = -73.6 (c = 0.11, CH_2Cl_2). ^{1}H NMR (400 MHz, Acetone-d_6) δ 7.79 (d, *J* = 2.1 Hz, 1H), 7.69 (dd, *J* = 8.6, 2.0 Hz, 1H), 7.63 – 7.55 (m, 2H), 7.50 – 7.38 (m, 4H), 7.34

(t, J = 8.5 Hz, 3H), 7.22 – 7.14 (m, 2H), 6.01 (d, J = 1.1 Hz, 1H), 4.47 (d, J = 4.5 Hz, 1H), 4.34 (dd, J = 9.7, 4.7 Hz, 1H), 4.27 (dd, J = 9.7, 6.9 Hz, 1H), 3.71 – 3.59 (m, 1H), 3.51 – 3.38 (m, 2H), 2.48 (d, J = 1.0 Hz, 3H), 2.16 (s, 3H), 1.40 (s, 9H); ^{13}C NMR (101 MHz, Acetone-d_6) δ 173.3, 172.6, 170.6, 158.7, 152.3, 144.6, 140.4, 137.1, 134.1, 133.7, 131.3, 129.5, 129.4, 129.4, 128.8, 128.6, 124.1, 118.4, 114.8, 111.8, 105.5, 82.0, 70.5, 66.2, 39.2, 34.4, 28.1, 14.6, 13.8. HRMS-ESI (m/z): $[M+H]^+$ calcd. for $C_{35}H_{36}ClN_4O_4^+$: 611.2420, found: 611.2417 HPLC test condition: Chiralpak AD-H, Hexane/2-Propanol = 90:10, flow rate 1.0 mL/min, test wavelength 254 nm, t_R (minor) = 6.476 min and 9.802 min, t_R (major) = 11.126 min and 20.032 min, dr > 20:1.

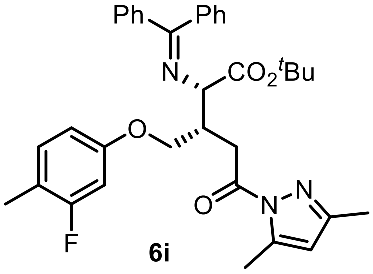

6i

tert-Buty (2*S*,3*S*)-5-(3,5-dimethyl-1*H*-pyrazol-1-yl)-2-((diphenylmethylene)amino)-3-((3-fluoro-4-methylphenoxy)methyl) -5-oxopentanoate (**6i**): colorless sticky oil, 74% yield, 98% *ee*, $[\alpha]_D^{20}$ = -64.0 (c = 0.10, CH_2Cl_2). ^{1}H NMR (400 MHz, Acetone-d_6) δ 7.65 – 7.57 (m, 2H), 7.52 – 7.38 (m, 4H), 7.38 – 7.30 (m, 2H), 7.18 – 7.05 (m, 3H), 6.59 (d, J = 10.3 Hz, 2H), 6.03 (s, 1H), 4.36 (d, J = 4.2 Hz, 1H), 4.13 (dd, J = 9.6, 5.1 Hz, 1H), 3.98 (dd, J = 9.6, 8.2 Hz, 1H), 3.64 (dd, J = 17.2, 4.6 Hz, 1H), 3.44 (dd, J = 17.1, 8.0 Hz, 1H), 3.39 – 3.30 (m, 1H), 2.49 (d, J = 1.0 Hz, 3H), 2.17 (s, 3H), 2.14 (d, J = 1.9 Hz, 3H), 1.41 (s, 9H); ^{13}C NMR (101 MHz, Acetone-d_6) δ 173.4, 172.3, 170.8, 162.3 (d, J = 242.8 Hz), 159.0 (d, J = 10.5 Hz), 152.2, 144.5, 140.4, 137.4, 132.5 (d, J = 6.7 Hz), 131.3, 129.5, 129.4, 129.3, 128.9, 128.6, 116.9 (d, J = 17.6 Hz), 111.8, 111.1 (d, J = 3.0 Hz), 102.6 (d, J = 25.8 Hz), 81.8, 69.1, 66.2, 39.1, 34.4, 28.2, 14.6, 13.8, 13.7 (d, J = 3.5 Hz); ^{19}F NMR (376 MHz, Acetone-d_6) δ 60.6. HRMS-ESI (m/z): $[M+H]^+$ calcd. for $C_{35}H_{39}FN_3O_4^+$: 584.2919, found: 584.2909. HPLC test condition: Chiralpak OD-H, Hexane/2-Propanol = 93:7, flow rate 1.0 mL/min, test wavelength 254 nm, t_R (major) = 5.014 min and 5.743 min, t_R (minor) = 6.777 min and 7.600 min, dr > 20:1.

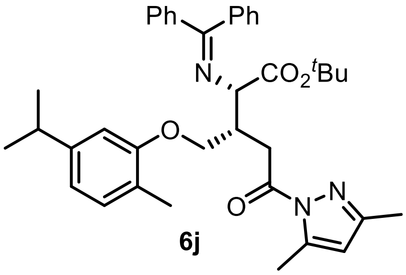

6j

tert-Butyl (2*S*,3*S*)-5-(3,5-dimethyl-1*H*-pyrazol-1-yl)-2-((diphenylmethylene)amino)-3-((5-isopropyl-2-methylphenoxy) methyl)-5-oxopentanoate (**6j**): colorless sticky oil, 83% yield, 98% *ee*, $[\alpha]_D^{20}$ = -55.8 (c = 0.11, CH_2Cl_2). ^{1}H NMR (400 MHz, Acetone-d_6) δ 7.63 – 7.57 (m, 2H), 7.48 – 7.39 (m, 4H), 7.38 – 7.31 (m, 2H), 7.19 – 7.12 (m, 2H), 6.96 (dd, J = 7.6, 0.9 Hz, 1H), 6.81 (d, J = 1.6 Hz, 1H), 6.67 (dd, J = 7.6, 1.7 Hz, 1H), 6.00 (d, J = 1.1 Hz, 1H), 4.45 (d, J = 4.8 Hz, 1H), 4.12 – 4.05 (m, 2H), 3.68 – 3.58 (m, 1H), 3.47 – 3.37 (m, 2H), 2.83 – 2.75 (m, 1H), 2.49 (d, J = 1.0 Hz, 3H), 2.16 (s, 3H), 1.87 (s, 3H), 1.39 (s, 9H), 1.16 (d, J = 6.9 Hz, 6H); ^{13}C NMR (101 MHz, Acetone-d_6) δ 173.6, 172.2, 171.0, 157.7, 152.2, 148.7, 144.5, 140.5, 137.4, 131.2, 131.0, 129.4, 129.3, 128.8, 128.6, 124.4, 118.8, 111.7, 110.1, 81.7, 68.6, 66.7, 39.6, 34.8, 34.6, 28.2, 24.4, 16.0, 14.6, 13.8. HRMS-ESI (m/z): $[M+H]^+$ calcd. for $C_{38}H_{46}N_3O_4^+$: 608.3483, found: 608.3477. HPLC test condition: Chiralpak OD-H, Hexane/2-Propanol = 97:3, flow rate 0.8 mL/min, test wavelength 254 nm,t_R (major) = 13.323 min and 15.850 min, t_R (minor) = 20.389 min, dr > 20:1.

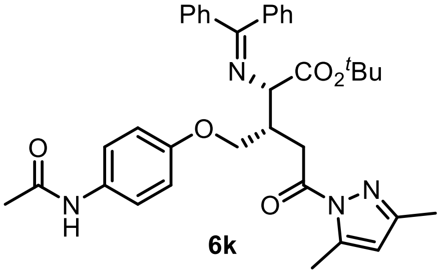

6k

tert-Butyl (2*S*,3*S*)-3-((4-acetamidophenoxy)methyl)-5-(3,5-dimethyl-1*H*-pyrazol-1-yl)-2-((diphenylmethylene)amino)-5-oxopentanoate (**6k**): white solid, 65% yield, m.p. 68.7-71.3 °C, 86% *ee*, $[\alpha]_D^{20}$ = -41.0 (c = 0.10, CH_2Cl_2). ^{1}H NMR (400 MHz, Acetone-*d*6) δ 7.64 – 7.57 (m, 2H), 7.56 – 7.45 (m, 5H), 7.44 – 7.39 (m, 1H), 7.38 – 7.30 (m, 2H), 7.18 – 7.10 (m, 2H), 6.82 – 6.74 (m, 2H), 6.03 (s, 1H), 4.38 (d, *J* = 4.1 Hz, 1H), 4.10 (dd, *J* = 9.5, 5.1 Hz, 1H), 4.01 – 3.91 (m, 1H), 3.69 – 3.59 (m, 1H), 3.44 (dd, *J* = 17.1, 7.9 Hz, 1H), 3.39 – 3.30 (m, 1H), 2.49 (s, 3H), 2.17 (s, 3H), 2.03 (s, 3H), 1.41 (s, 9H); ^{13}C NMR (101 MHz, Acetone-*d*6) δ 173.5, 172.3, 170.9, 168.4, 168.3, 155.5, 152.2, 144.5, 140.4, 137.4, 131.3, 129.5, 129.4, 129.3, 128.9, 128.6, 121.4, 121.3, 115.2, 111.8, 81.8, 68.9, 66.3, 39.3, 34.4, 28.2, 24.1, 14.7, 13.8. HRMS-ESI (m/z): $[M+H]^+$ calcd. for $C_{36}H_{41}N_4O_5^+$: 609.3071, found: 609.3066. HPLC test condition: Chiralpak OD-H, Hexane/2-Propanol = 80:20, flow rate 0.9 mL/min, test wavelength 254 nm, t_R (minor) = 6.174 min, t_R (major) = 7.695 min, dr > 20:1.

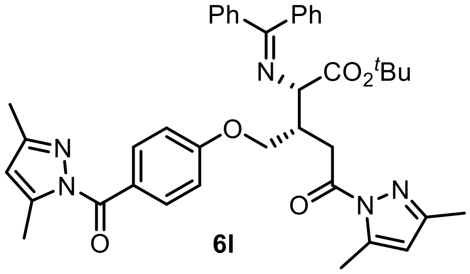

6l

tert-Butyl (2*S*,3*S*)-5-(3,5-dimethyl-1*H*-pyrazol-1-yl)-3-((4-(3,5-dimethyl-1*H*-pyrazole-1-carbonyl)phenoxy)methyl)-2-((diphenylmethylene)amino)-5-oxopentanoate (**6l**): white solid, 46% yield, m.p. 59.3-62.1 °C, 98% *ee*, $[\alpha]_D^{20}$ = -15.0 (c = 0.12, CH_2Cl_2). ^{1}H NMR (400 MHz, Acetone-*d*6) δ 8.06 – 7.97 (m, 2H), 7.67 – 7.58 (m, 2H), 7.53 – 7.46 (m, 3H), 7.46 – 7.40 (m, 1H), 7.39 – 7.32 (m, 2H), 7.20 – 7.12 (m, 2H), 6.99 – 6.91 (m, 2H), 6.16 (d, *J* = 1.1 Hz, 1H), 6.04 (d, *J* = 1.1 Hz, 1H), 4.39 (d, *J* = 4.0 Hz, 1H), 4.28 (dd, *J* = 9.6, 5.1 Hz, 1H), 4.12 (dd, *J* = 9.7, 8.2 Hz, 1H), 3.66 (dd, *J* = 17.2, 4.6 Hz, 1H), 3.48 (dd, *J* = 17.2, 8.0 Hz, 1H), 3.44 – 3.34 (m, 1H), 2.55 (d, *J* = 1.0 Hz, 3H), 2.50 (d, *J* = 1.0 Hz, 3H), 2.18 (d, *J* = 0.9 Hz, 6H), 1.43 (s, 9H); ^{13}C NMR (101 MHz, Acetone-*d*6) δ 173.4, 172.4, 170.7, 167.9, 163.0, 152.3, 152.1, 145.5, 144.5, 140.4, 137.3, 134.7, 131.3, 129.6, 129.4, 129.4, 128.9, 128.6, 126.5, 114.4, 111.8, 111.4, 81.9, 69.1, 66.2, 39.2, 34.4, 28.2, 14.7, 14.3, 13.8, 13.8. HRMS-ESI (m/z): $[M+H]^+$ calcd. for $C_{40}H_{44}N_5O_5^+$: 674.3337, found: 674.3336. HPLC test condition: Chiralpak OD-H, Hexane/2-Propanol = 95:5, flow rate 0.8 mL/min, test wavelength 254 nm, t_R (major) = 9.692 min, t_R (minor) = 11.523 min, dr > 20:1.

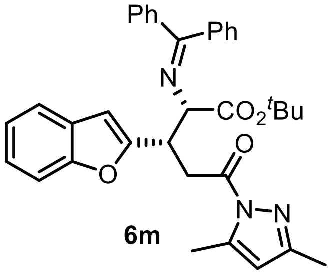

6m

tert-Butyl (2*S*,3*S*)-3-(benzofuran-2-yl)-5-(3,5-dimethyl-1*H*-pyrazol-1-yl)-2-((diphenylmethylene)amino)-5-oxopentanoate (**6m**): colorless sticky oil, 70% yield, 93% *ee*, $[\alpha]_D^{20}$ = -13.3 (c = 0.11, CH_2Cl_2). ^{1}H NMR (400 MHz, Acetone-*d*6) δ 7.63 – 7.53 (m, 2H), 7.51 – 7.28 (m, 8H), 7.26 – 7.20 (m, 1H), 7.19 – 7.12 (m, 1H), 6.93 (d, *J* = 7.1 Hz, 2H), 6.59 (s, 1H), 5.99 (s, 1H), 4.52 – 4.40 (m, 2H), 3.99 – 3.86 (m, 1H), 3.86 – 3.76 (m, 1H), 2.42 (s, 3H), 2.20 (s, 3H), 1.37 (s, 9H); ^{13}C NMR (101 MHz, Acetone-*d*6) δ 172.5, 172.3, 169.9, 159.2, 155.5, 152.5, 144.5, 140.2, 137.0, 131.4, 129.6, 129.5, 129.3, 128.9, 128.5, 124.6, 123.5, 121.5, 111.9, 111.6, 104.3, 82.0, 69.3, 40.1, 35.7, 28.1, 14.6, 13.9. HRMS-ESI (m/z): $[M+H]^+$ calcd. for

$C_{35}H_{36}N_3O_4^+$: 562.2706, found: 562.2701. HPLC test condition: Chiralpak AD-H, Hexane/2-Propanol = 90:10, flow rate 1.0 mL/min, test wavelength 254 nm, t_R (minor) = 5.852 min and 8.134 min, t_R (major) = 6.594 min and 14.373 min, dr = 96:4.

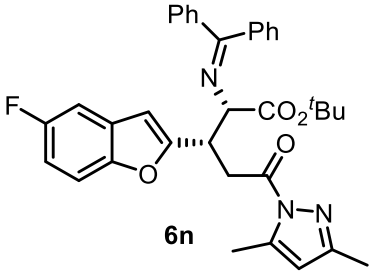

6n

tert-Butyl (2*S*,3*S*)-5-(3,5-dimethyl-1*H*-pyrazol-1-yl)-2-((diphenylmethylene)amino)-3-(5-fluorobenzofuran-2-yl)-5-oxo-pentanoate (**6n**): colorless sticky oil, 67% yield, 97% *ee*, $[\alpha]_D^{20}$ = +5.0 (c = 0.10, CH_2Cl_2). ^1H NMR (400 MHz, Acetone-d_6) δ 7.64 – 7.55 (m, 2H), 7.51 – 7.31 (m, 7H), 7.25 (dd, J = 8.8, 2.7 Hz, 1H), 7.03 (td, J = 9.2, 2.7 Hz, 1H), 6.96 (d, J = 7.3 Hz, 2H), 6.65 (s, 1H), 6.01 (s, 1H), 4.51 – 4.41 (m, 2H), 3.98 – 3.88 (m, 1H), 3.88 – 3.79 (m, 1H), 2.44 (s, 3H), 2.21 (s, 3H), 1.39 (s, 9H); ^{13}C NMR (101 MHz, Acetone-d_6) δ 172.5, 169.8, 161.4, 160.0 (d, J = 236.1 Hz), 152.5, 151.7, 144.6, 140.2, 137.0, 131.4, 130.5 (d, J = 11.0 Hz), 129.6, 129.5, 129.3, 128.9, 128.5, 112.5 (d, J = 9.6 Hz), 112.1, 111.9, 111.8, 107.0 (d, J = 25.2 Hz), 104.7 (d, J = 3.9 Hz), 82.1, 69.2, 40.2, 35.7, 28.1, 14.6, 13.9; ^{19}F NMR (376 MHz, Acetone-d_6) δ 55.1. HRMS-ESI (m/z): $[M+H]^+$ calcd. for $C_{35}H_{35}N_3O_4F^+$: 580.2612, found: 580.2607. HPLC test condition: Chiralpak AD-H, Hexane/2-Propanol = 90:10, flow rate 1.0 mL/min, test wavelength 254 nm, t_R (minor) = 5.875 min and 9.239 min, t_R (major) = 7.463 min and 19.360 min, dr = 98:2.

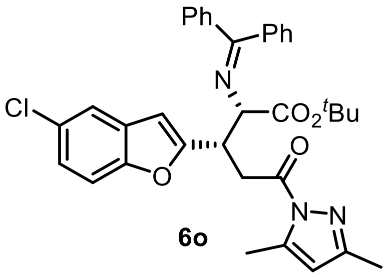

6o

tert-Butyl (2*S*,3*S*)-3-(5-chlorobenzofuran-2-yl)-5-(3,5-dimethyl-1*H*-pyrazol-1-yl)-2-((diphenylmethylene)amino)-5-oxo-pentanoate (**6o**): colorless sticky oil, 56% yield, 98% *ee*, $[\alpha]_D^{20}$ = +12.4 (c = 0.11, CH_2Cl_2). ^1H NMR (400 MHz, Acetone-d_6) δ 7.62 – 7.53 (m, 3H), 7.50 – 7.33 (m, 7H), 7.28 – 7.22 (m, 1H), 6.96 (d, J = 7.4 Hz, 2H), 6.64 (s, 1H), 6.02 (s, 1H), 4.50 – 4.40 (m, 2H), 3.98 – 3.78 (m, 2H), 2.44 (s, 3H), 2.21 (s, 3H), 1.39 (s, 9H); ^{13}C NMR (101 MHz, Acetone-d_6) δ 172.5, 172.4, 169.8, 161.2, 153.9, 152.6, 144.6, 140.2, 137.0, 131.4, 131.1, 129.6, 129.5, 129.3, 128.9, 128.5, 124.6, 121.1, 113.0, 111.9, 104.2, 82.1, 69.2, 40.1, 35.7, 28.1, 14.6, 13.9. HRMS-ESI (m/z): $[M+H]^+$ calcd. for $C_{35}H_{35}N_3O_4Cl^+$: 596.2316, found: 596.2311. HPLC test condition: Chiralpak AD-H, Hexane/2-Propanol = 90:10, flow rate 1.0 mL/min, test wavelength 254 nm, t_R (minor) = 5.819 min and 10.432 min, t_R (major) = 8.208 min and 13.349 min, dr = 97:3.

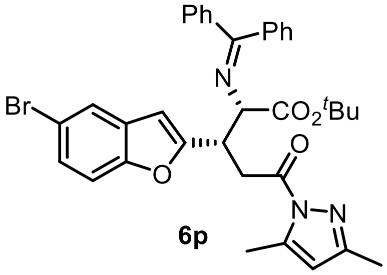

6p

tert-Butyl (2*S*,3*S*)-3-(5-bromobenzofuran-2-yl)-5-(3,5-dimethyl-1*H*-pyrazol-1-yl)-2-((diphenylmethylene)amino)-5-oxo-pentanoate (**6p**): colorless sticky oil, 48% yield, 98% *ee*, $[\alpha]_D^{20}$ = +23.3 (c = 0.11, CH_2Cl_2). ^1H NMR (400 MHz, Acetone-d_6) δ 7.70 – 7.66 (m, 1H), 7.61 – 7.54 (m, 2H), 7.47 – 7.38 (m, 4H), 7.37 – 7.31 (m, 4H), 6.98 – 6.90 (m, 2H), 6.62 (d, J = 0.8 Hz, 1H), 6.00 (d, J = 1.1 Hz, 1H), 4.48 – 4.39 (m, 2H), 3.94 – 3.77 (m, 2H), 2.42 (d, J = 1.0 Hz, 3H), 2.19 (s, 3H), 1.37 (s, 9H); ^{13}C NMR (101 MHz, Acetone-d_6) δ 172.5, 172.4, 169.8, 161.0, 154.2, 152.5, 144.6, 140.2, 137.0, 131.7, 131.4, 129.6, 129.5, 129.3, 128.9, 128.5, 127.3, 124.2, 116.2, 113.5, 111.9, 104.0, 82.1, 69.1, 40.1, 35.7, 28.1, 14.5, 13.9.

HRMS-ESI (m/z): $[M+H]^+$ calcd. for $C_{35}H_{35}N_3O_4Br^+$: 640.1811, found: 640.1804. HPLC test condition: Chiralpak AD-H, Hexane/2-Propanol = 90:10, flow rate 1.0 mL/min, test wavelength 254 nm, t_R (major) = 8.671 min and 12.616 min, t_R (minor) = 11.198 min and 21.486 min, dr = 92:8.

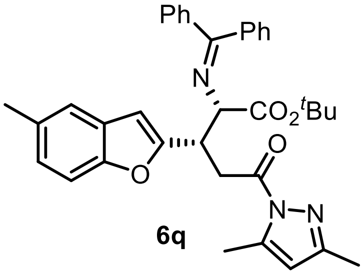

6q

tert-Butyl (2*S*,3*S*)-5-(3,5-dimethyl-1*H*-pyrazol-1-yl)-2-((diphenylmethylene)amino)-3-(5-methylbenzofuran-2-yl)-5-oxo-pentanoate (**6q**): colorless sticky oil, 27% yield, 93.5% *ee*, $[\alpha]_D^{20}$ = +4.7 (c = 0.11, CH_2Cl_2). ^1H NMR (400 MHz, Acetone-d_6) δ 7.57 (d, *J* = 7.9 Hz, 2H), 7.49 – 7.29 (m, 6H), 7.24 (d, *J* = 9.3 Hz, 2H), 7.04 (d, *J* = 8.3 Hz, 1H), 6.93 (d, *J* = 7.1 Hz, 2H), 6.50 (s, 1H), 5.99 (s, 1H), 4.50 – 4.39 (m, 2H), 3.91 (dd, *J* = 17.3, 7.9 Hz, 1H), 3.80 (dd, *J* = 17.3, 4.3 Hz, 1H), 2.42 (s, 3H), 2.34 (s, 3H), 2.20 (s, 3H), 1.38 (s, 9H); ^{13}C NMR (101 MHz, Acetone-d_6) δ 172.6, 172.3, 169.9, 159.3, 153.9, 152.4, 144.5, 140.2, 137.0, 132.7, 131.4, 129.6, 129.6, 129.5, 129.3, 128.9, 128.5, 125.7, 121.4, 111.9, 111.1, 104.1, 82.0, 69.3, 40.1, 35.7, 28.1, 21.4, 14.6, 13.9. HRMS-ESI (m/z): $[M+H]^+$ calcd. for $C_{36}H_{38}N_3O_4Br^+$: 576.2862, found: 576.2864. HPLC test condition: Chiralpak AD-H, Hexane/2-Propanol = 90:10, flow rate 1.0 mL/min, test wavelength 254 nm, t_R (minor) = 5.429 min and 8.190 min, t_R (major) = 7.242 min and 9.885 min, dr = 99:1.

2.3 Typical procedure for in-situ acidic hydrolysis and lactamization to 7d.

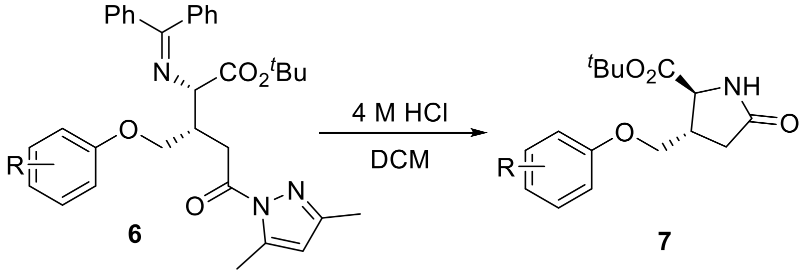

6 **7**

To **6d** in DCM (10.0 mL) added 4 M HCl and the mixture was stirred at r.t. for 2–3 h. After the reaction completed (detected by TLC), it was quenched by H_2O (15 mL) and extracted with DCM (2 × 25 mL). The combined organic layers were dried over Na_2SO_4 and the solvents were evaporated under vacuum. The residue was purified by a flash column chromatograph to afford pure **7d** as a white solid.

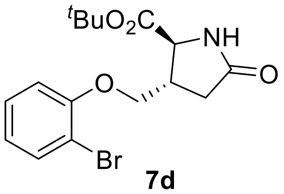

7d

tert-Butyl (2*S*,3*S*)-3-((2-bromophenoxy)methyl)-5-oxopyrrolidine-2-carboxylate (**7d**): white solid, 86% yield, m.p. 111.6-112.4 °C, 97% *ee*, $[\alpha]_D^{20}$ = +32.8 (c = 0.10, CH_2Cl_2). ^1H NMR (400 MHz, Chloroform-*d*) δ 7.56 (dd, *J* = 7.9, 1.6 Hz, 1H), 7.34 – 7.23 (m, 1H), 6.97 – 6.82 (m, 2H), 6.52 (s, 1H), 4.26 (d, *J* = 5.5 Hz, 1H), 4.22 – 4.09 (m, 2H), 3.01 (ddq, *J* = 10.4, 6.9, 5.2 Hz, 1H), 2.59 (qd, *J* = 17.1, 8.0 Hz, 2H), 1.50 (s, 9H); ^{13}C NMR (101 MHz, Chloroform-*d*) δ 176.3, 170.5, 154.7, 133.5, 128.5, 122.6, 113.5, 112.6, 82.8, 69.2, 57.7, 38.4, 32.8, 28.0. HRMS-ESI (m/z): $[M+H]^+$ calcd. for $C_{16}H_{21}BrNO_4{}^+$: 370.0648, found: 370.0655. HPLC test condition: Chiralpak AD-H, Hexane/2-Propanol = 80:20, flow rate 1.0 mL/min, test wavelength 230 nm, t_R (minor) = 16.597 min, t_R (major) = 11.036 min.

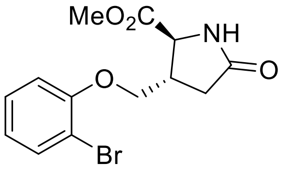

7d'

Methyl (*2S,3S*)-3-((2-bromophenoxy)methyl)-5-oxopyrrolidine-2-carboxylate (**7d'**): white solid, 84% yield, m.p. 93.6-94.9 °C, 93% *ee*, $[\alpha]_D^{20}$ = +32.0 (c = 0.10, CH_2Cl_2). ^1H NMR (400 MHz, Chloroform-*d*) δ 7.54 (dd, *J* = 7.8, 1.6 Hz, 1H), 7.28 (d, *J* = 7.5 Hz, 1H), 6.97 – 6.84 (m, 2H), 6.55 (s, 1H), 4.39 (d, *J* = 5.4 Hz, 1H), 4.23 – 4.08 (m, 2H), 3.80 (s, 3H), 3.13 – 3.00 (m, 1H), 2.64 (dd, *J* = 17.2, 9.2 Hz, 1H), 2.54 (dd, *J* = 17.2, 6.7 Hz, 1H); ^{13}C NMR (101 MHz, Chloroform-*d*) δ 176.2, 171.9, 154.6, 133.5, 128.5, 122.7, 113.5, 112.6, 69.1, 57.2, 52.8, 38.4, 32.5. HRMS-ESI (m/z): $[M+H]^+$ calcd. for $C_{13}H_{15}BrNO_4^+$:328.0179, found: 328.0172. HPLC test condition: Chiralpak AD-H, Hexane/2-Propanol = 80:20, flow rate 1.0 mL/min, test wavelength 230 nm, t_R (minor) = 18.966 min, t_R (major) = 15.792 min.

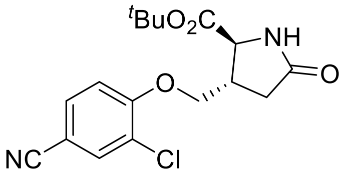

7h

tert-Butyl (*2S,3S*)-3-((2-chloro-4-cyanophenoxy)methyl)-5-oxopyrrolidine-2-carboxylate (**7h**): white solid, 86% yield, m.p. 121.6-122.8 °C, 98% *ee*, $[\alpha]_D^{20}$ = +43.8 (c = 0.11, CH_2Cl_2). ^1H NMR (400 MHz, Chloroform-*d*) δ 7.67 (d, *J* = 2.0 Hz, 1H), 7.55 (dd, *J* = 8.6, 2.0 Hz, 1H), 6.97 (d, *J* = 8.6 Hz, 1H), 6.28 (s, 1H), 4.27 – 4.14 (m, 3H), 3.11 – 2.96 (m, 1H), 2.63 (dd, *J* = 17.1, 9.3 Hz, 1H), 2.47 (dd, *J* = 17.2, 6.9 Hz, 1H), 1.48 (s, 9H); ^{13}C NMR (101 MHz, Chloroform-*d*) δ 175.6, 170.1, 157.4, 133.8, 132.4, 124.2, 117.7, 113.2, 105.6, 83.2, 69.4, 57.5, 38.2, 32.6, 28.0. HRMS-ESI (m/z): $[M+H]^+$ calcd. for $C_{17}H_{20}ClN_2O_4^+$: 351.1106, found: 351.1105. HPLC test condition: Chiralpak AD-H, Hexane/2-Propanol = 80:20, flow rate 1.0 mL/min, test wavelength 230 nm, t_R (minor) = 17.188 min, t_R (major) = 15.956 min.

3. X-ray single crystal diffraction data of 7d'[6]

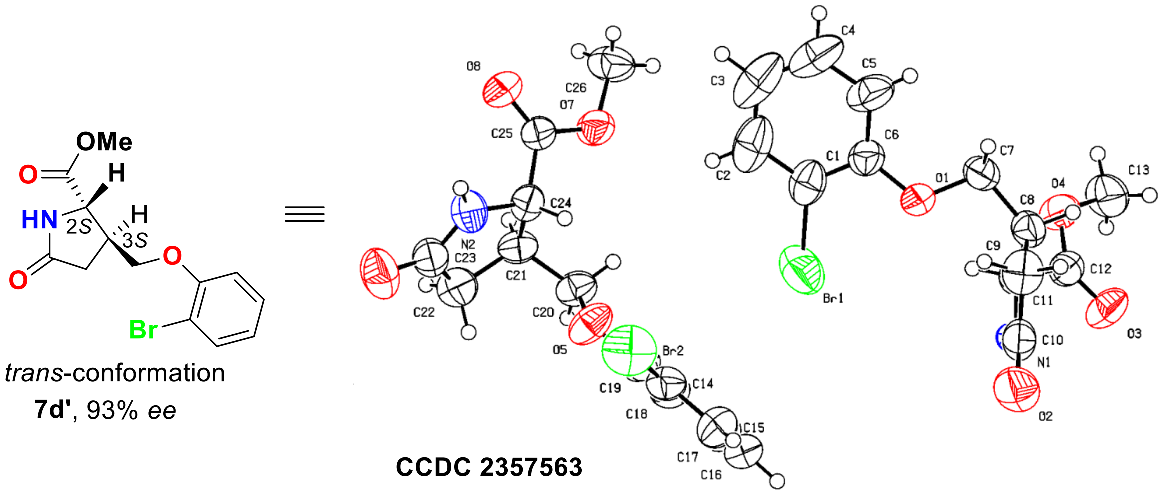

Table 1 Crystal data and structure refinement for 7d' (CCDC 2357563)

Identification code	ww
Empirical formula	$C_{13}H_{14}BrNO_4$
Formula weight	328.16
Temperature/K	303.00
Crystal system	monoclinic
Space group	$P2_1$

a/Å	8.978(3)
b/Å	6.518(2)
c/Å	24.261(7)
α/°	90
β/°	90.110(11)
γ/°	90
Volume/Å^3	1419.7(8)
Z	4
ρ_{calc}g/cm^3	1.535
μ/mm^{-1}	2.904
F(000)	664.0
Crystal size/mm^3	0.11 × 0.1 × 0.08
Radiation	MoKα (λ = 0.71073)
2Θ range for data collection/°	4.536 to 56.658
Index ranges	-11 ≤ h ≤ 11, -8 ≤ k ≤ 8, -32 ≤ l ≤ 32
Reflections collected	16828
Independent reflections	6753 [R_{int} = 0.0518, R_{sigma} = 0.0982]
Data/restraints/parameters	6753/1/346
Goodness-of-fit on F^2	1.010
Final R indexes [I>=2σ (I)]	R_1 = 0.0427, wR_2 = 0.1063
Final R indexes [all data]	R_1 = 0.0990, wR_2 = 0.1331
Largest diff. peak/hole / e Å^{-3}	0.27/-0.33
Flack parameter	0.054(12)

References

1. Sibi, M. P.; Itoh, K. *J. Am. Chem. Soc.*, **2007**, *129*, 8064-8065.
2. Biswas, R. G.; Ray, S. K.; Kannaujiya, V. K.; Unhale, R. A.; Singh, V. K. *Org. Biomol. Chem.*, **2021**, *19*, 4685-4690.
3. Bandar, J.S.; Lambert, T.H. *J. Am. Chem. Soc.*, **2012**, *134*, 5552-5555.
4. Wang, Z.; Hu, L.; Chekshin, N.; Zhuang, Z.; Qian, S.-Q.; Qiao, J.-X.; Yu, J.-Q. *Science*, **2021**, *374*, 1281-1285.
5. Abdel-Wahab, B. F.; Mohamed, H. A. *Phosphorus, Sulfur and Silicon and the Related Elements*, **2008**, *183*, 136-143.
6. The crystallographic data (CCDC 2357563) for **7d'**, can be obtained free of charge from the Cambridge crystallographic Data Centre via https://www.ccdc.cam.ac.uk/data_request/cif.

4. NMR scanning copies

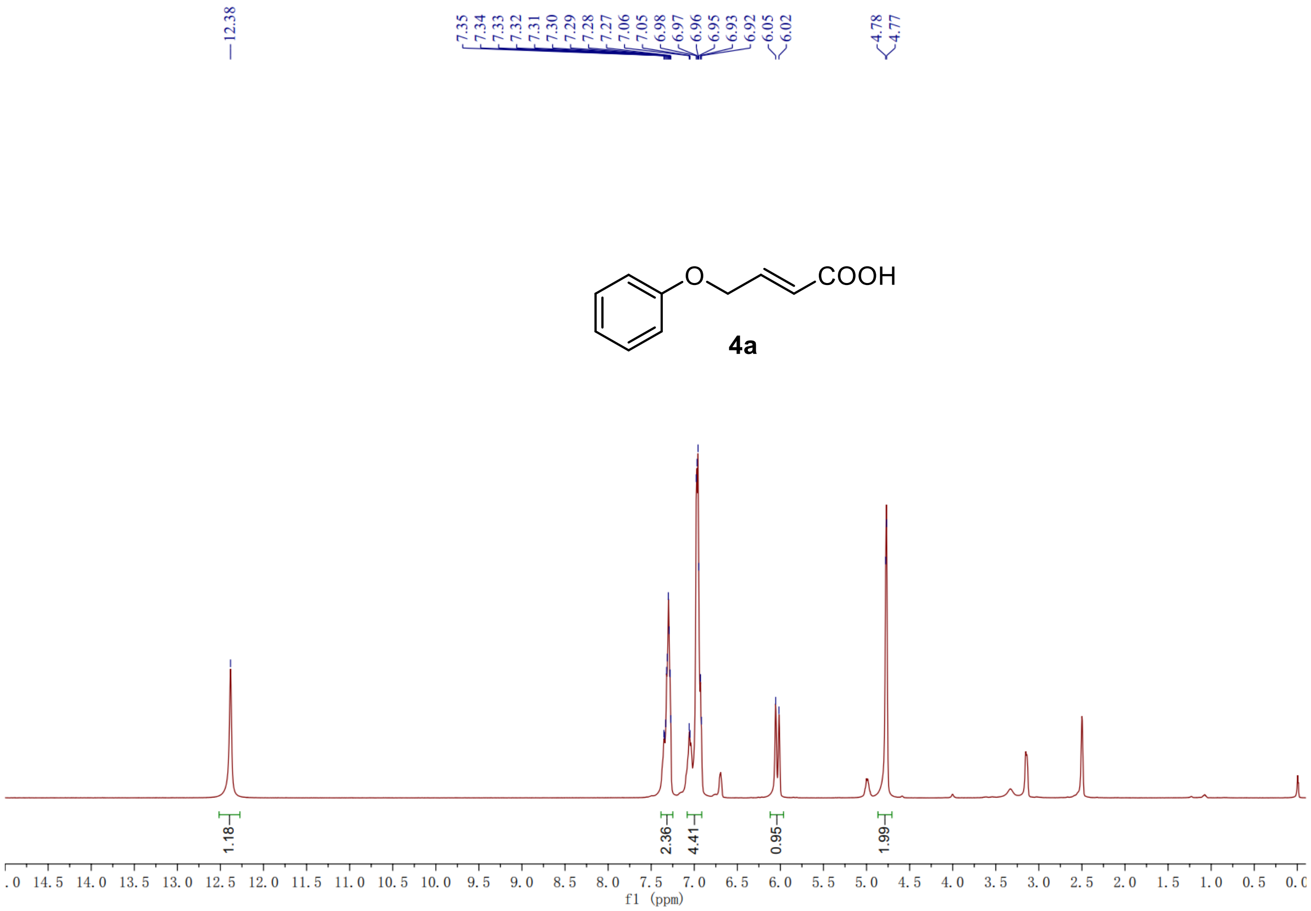

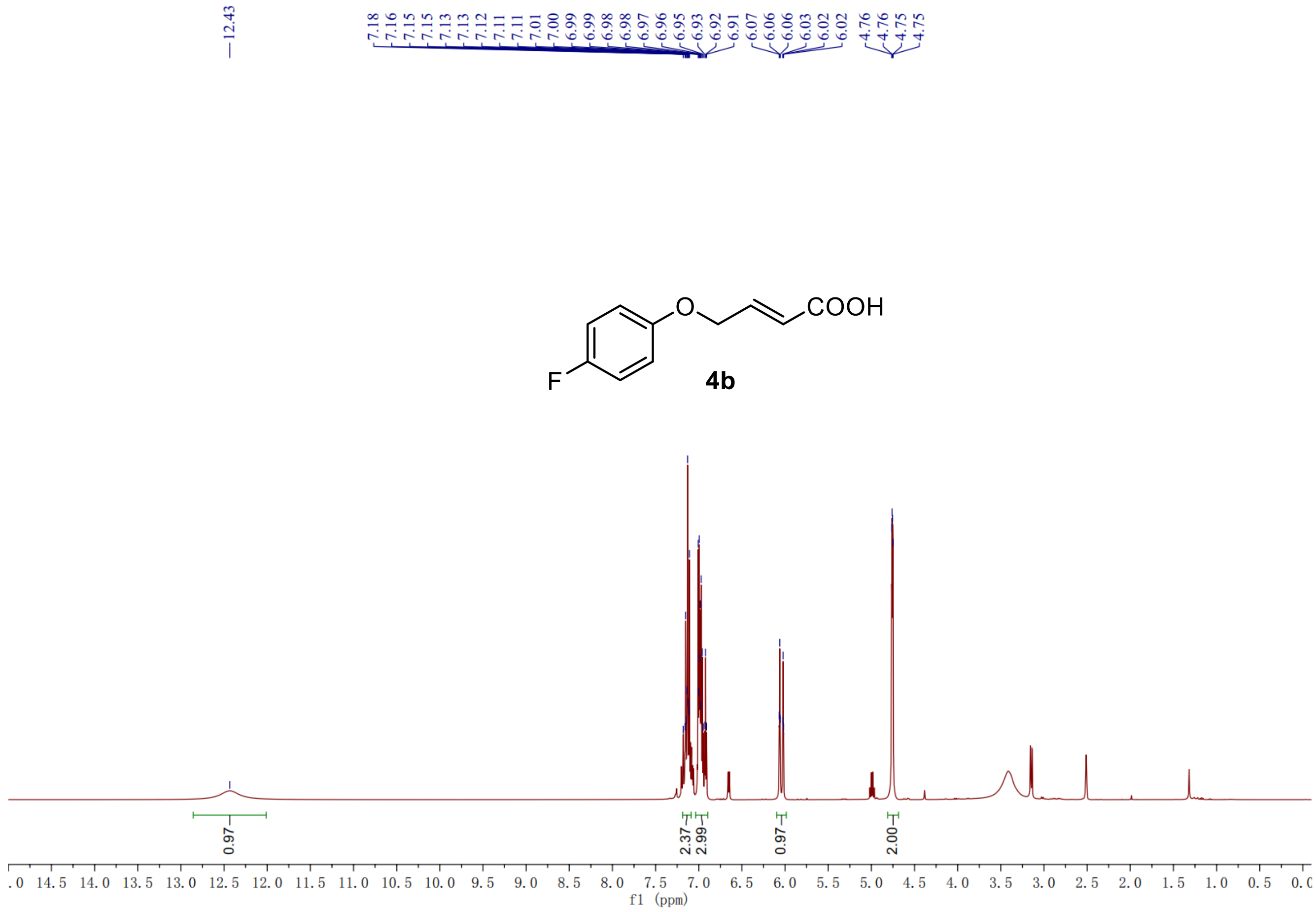

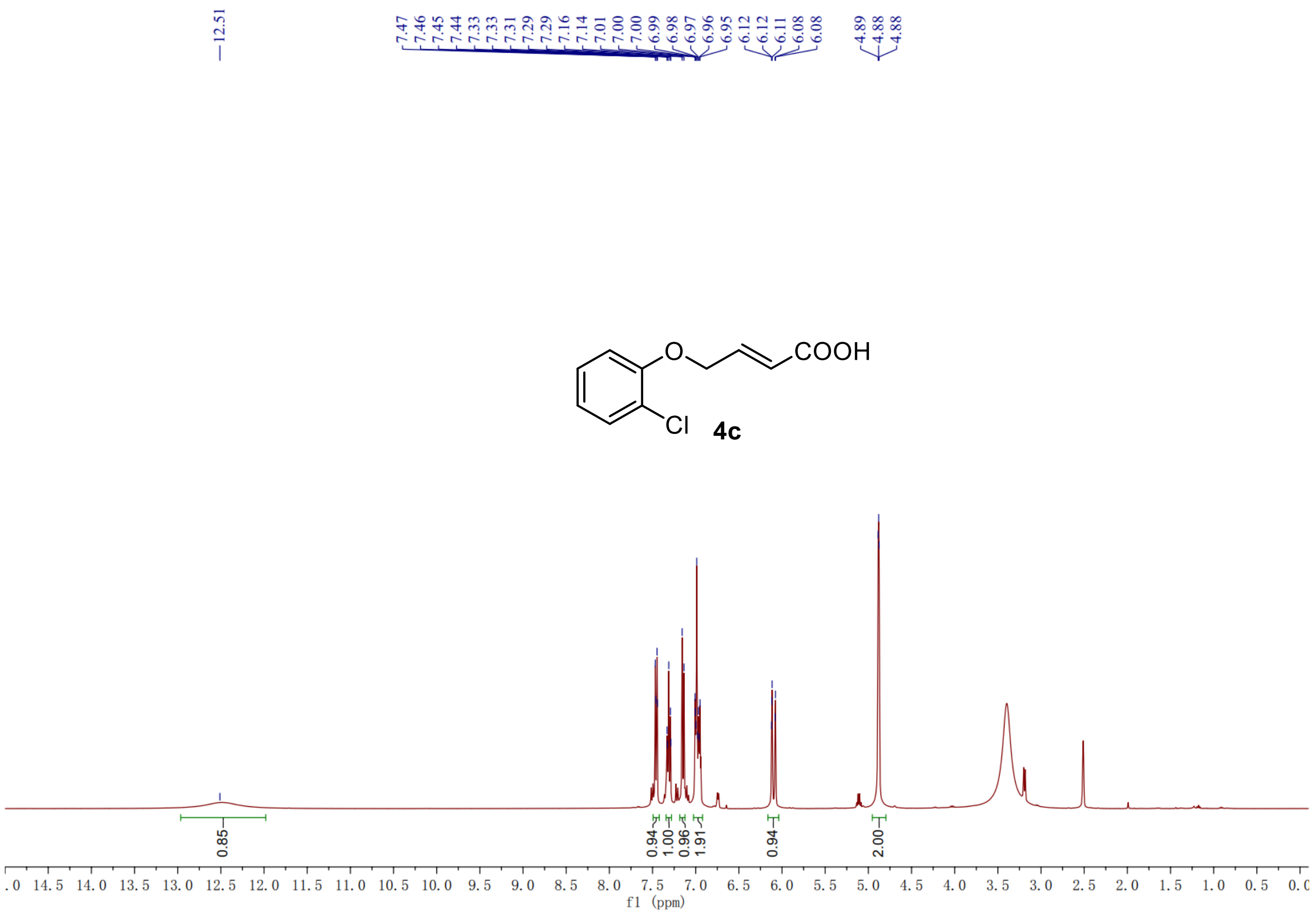
12.51
7.47 7.46 7.45 7.44 7.33 7.33 7.31 7.29 7.29 7.16 7.14 7.01 7.00 7.00 6.99 6.98 6.97 6.96 6.95 6.12 6.12 6.11 6.08 6.08 4.89 4.88 4.88
O
COOH
Cl
4c
0.85 0.94 1.00 0.96 1.91 0.94 2.00
f1 (ppm)

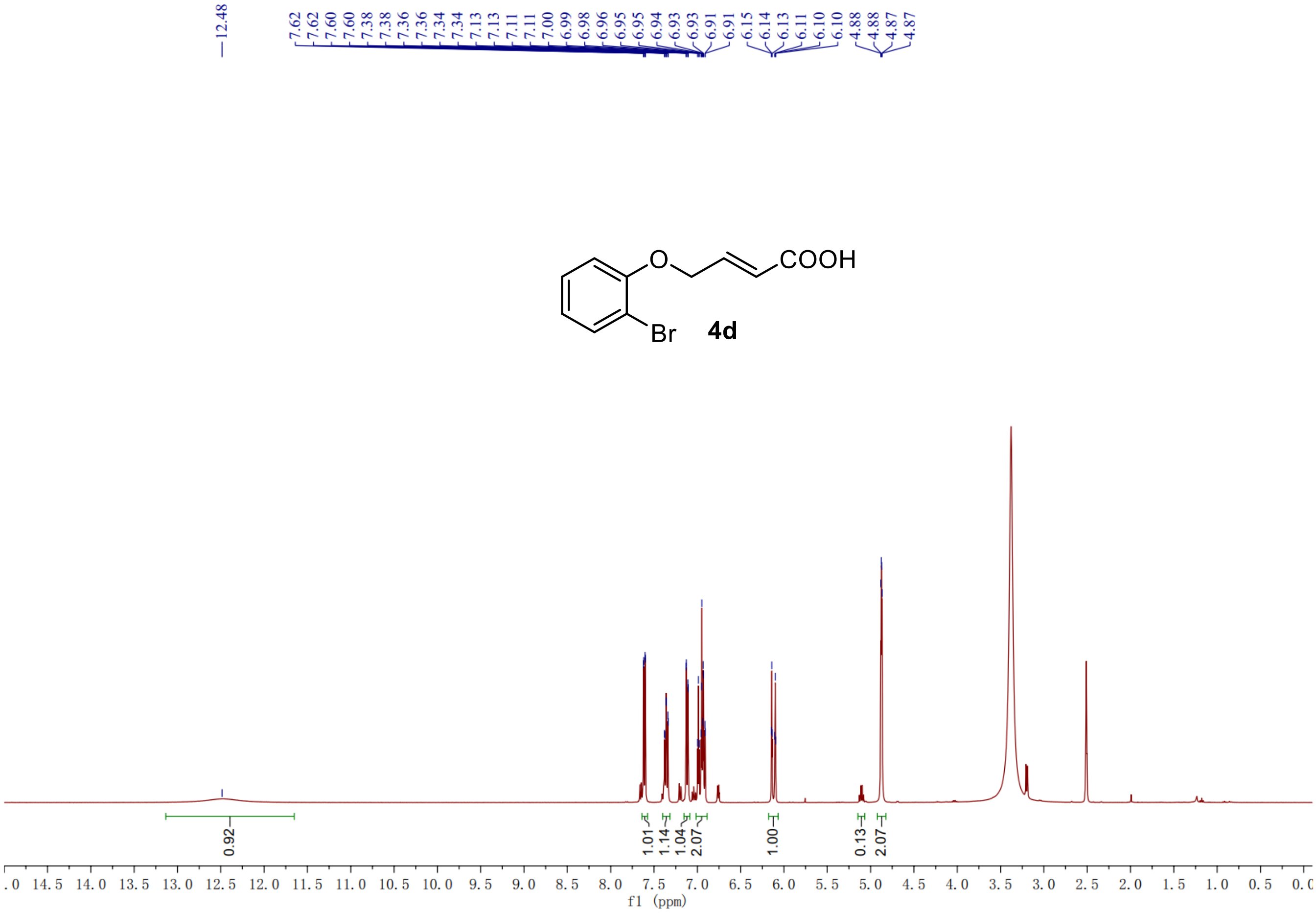
12.48
7.62 7.62 7.60 7.60 7.38 7.38 7.36 7.34 7.34 7.13 7.13 7.11 7.11 7.00 6.99 6.98 6.96 6.95 6.95 6.94 6.93 6.93 6.91 6.91 6.15 6.14 6.13 6.11 6.10 6.10 4.88 4.88 4.87 4.87
O
COOH
Br
4d
0.92 1.01 1.14 1.04 2.07 1.00 0.13 2.07
f1 (ppm)

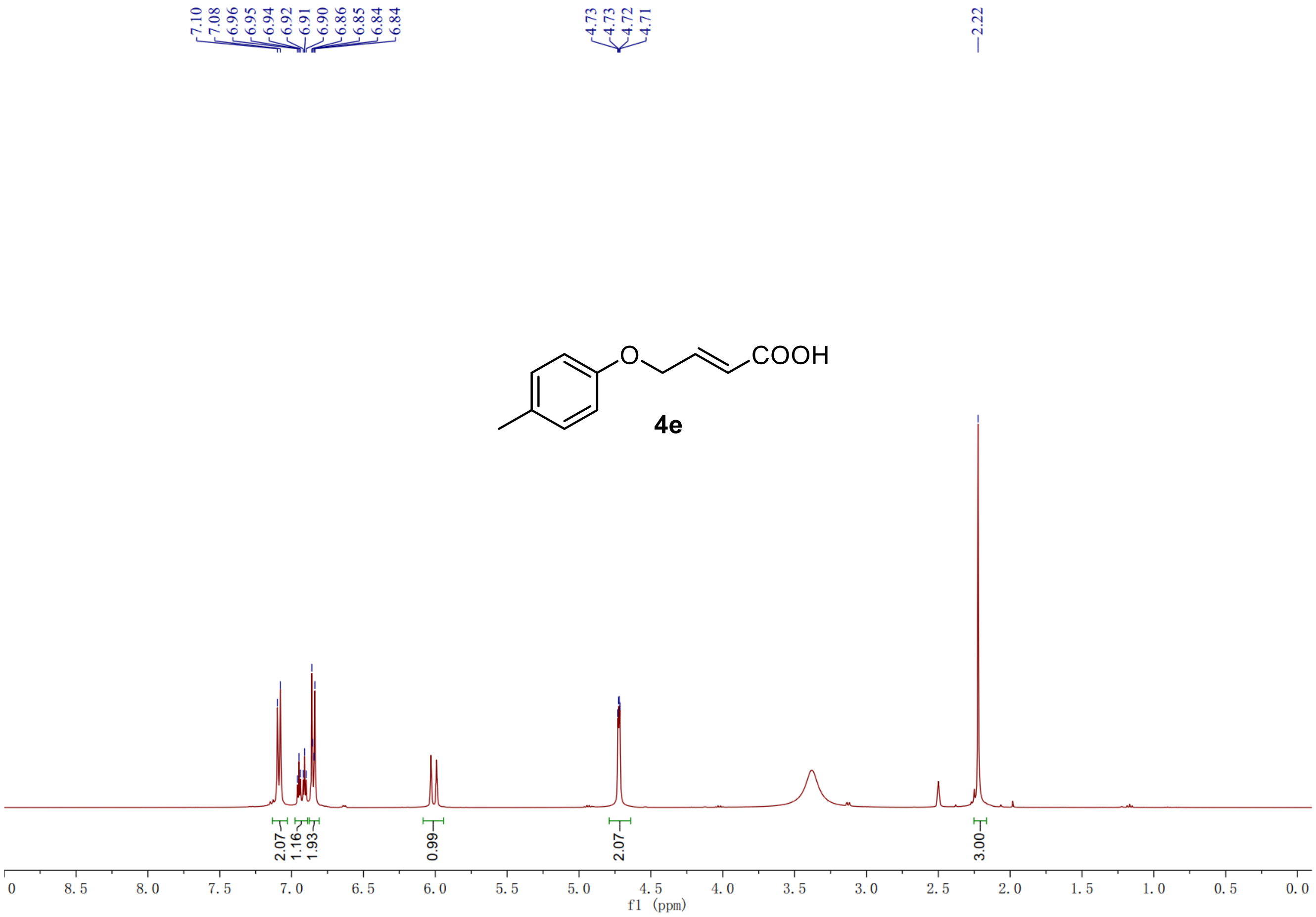
7.10 7.08 6.96 6.95 6.94 6.92 6.91 6.90 6.86 6.85 6.84 6.84
4.73 4.73 4.72 4.71
2.22
O
COOH
4e
2.07
1.16
1.93
0.99
2.07
3.00
f1 (ppm)

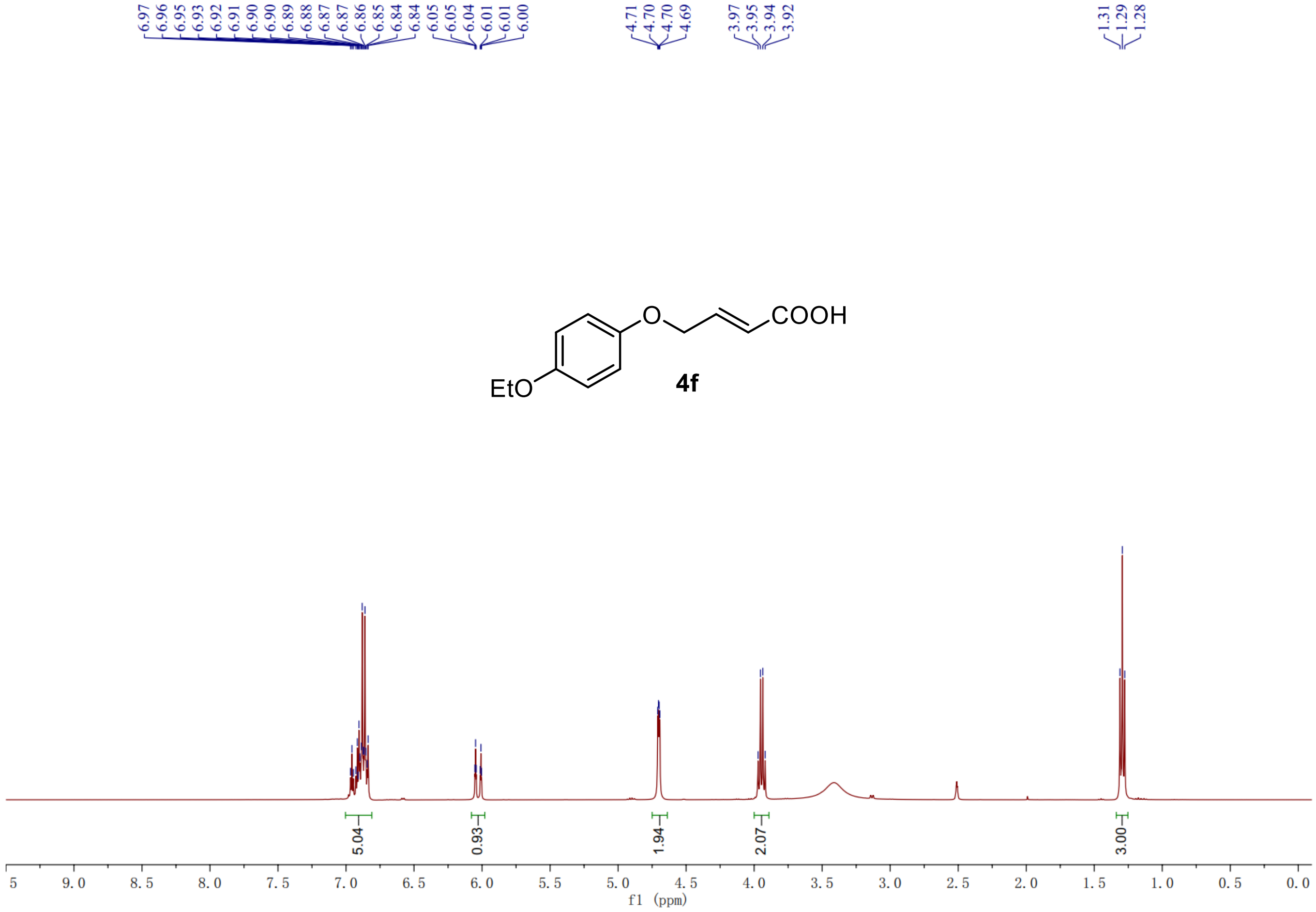
6.97 6.96 6.95 6.93 6.92 6.91 6.90 6.90 6.89 6.88 6.87 6.86 6.85 6.84 6.84 6.05 6.05 6.04 6.01 6.01 6.00
4.71 4.70 4.70 4.69
3.97 3.95 3.94 3.92
1.31 1.29 1.28
O
COOH
EtO
4f
5.04
0.93
1.94
2.07
3.00
f1 (ppm)

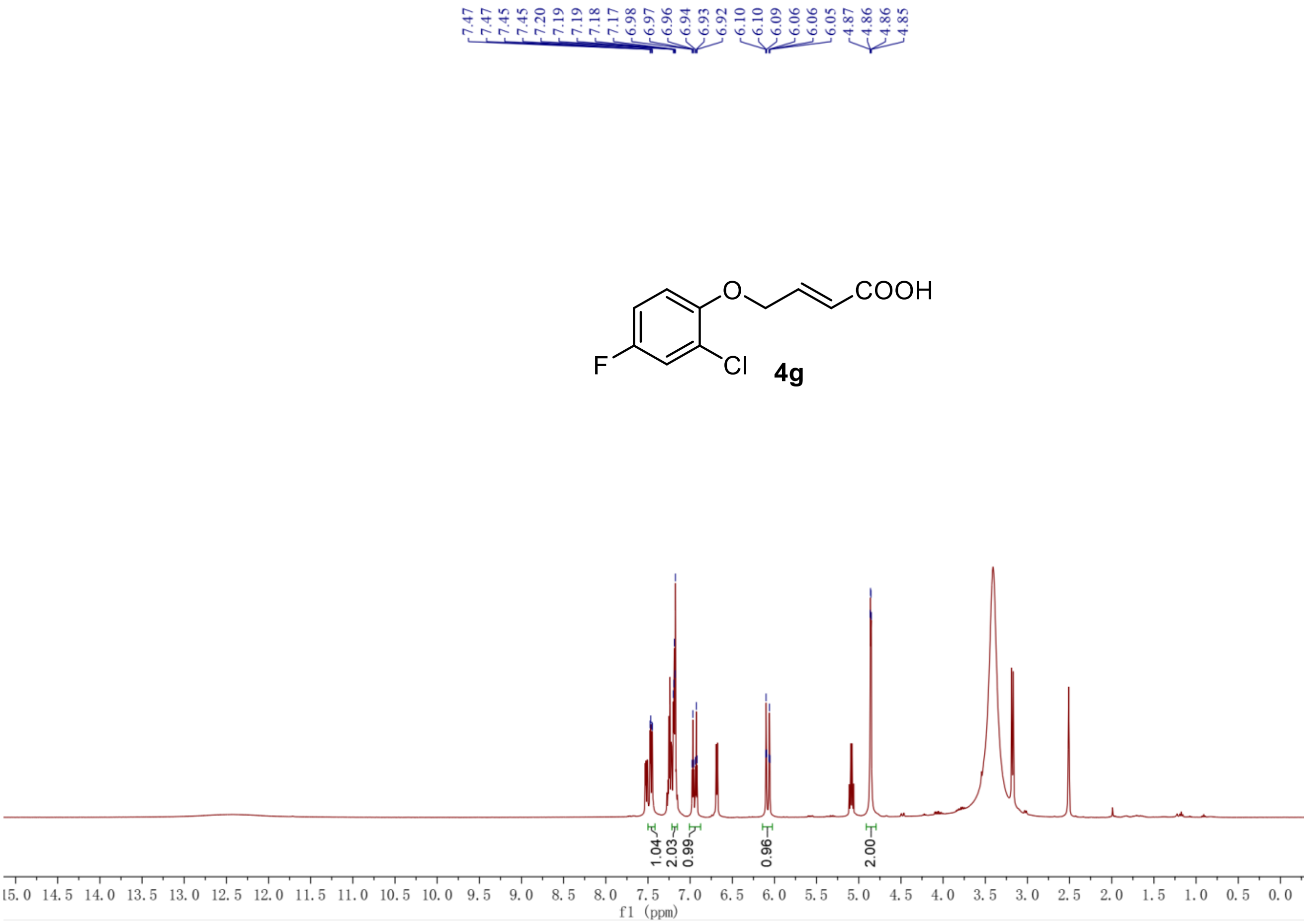
7.47 7.47 7.45 7.45 7.20 7.19 7.19 7.18 7.17 6.98 6.97 6.96 6.94 6.93 6.92 6.10 6.10 6.09 6.06 6.06 6.05 4.87 4.86 4.86 4.85
COOH
F
Cl
4g
1.04
2.03
0.99
0.96
2.00
f1 (ppm)

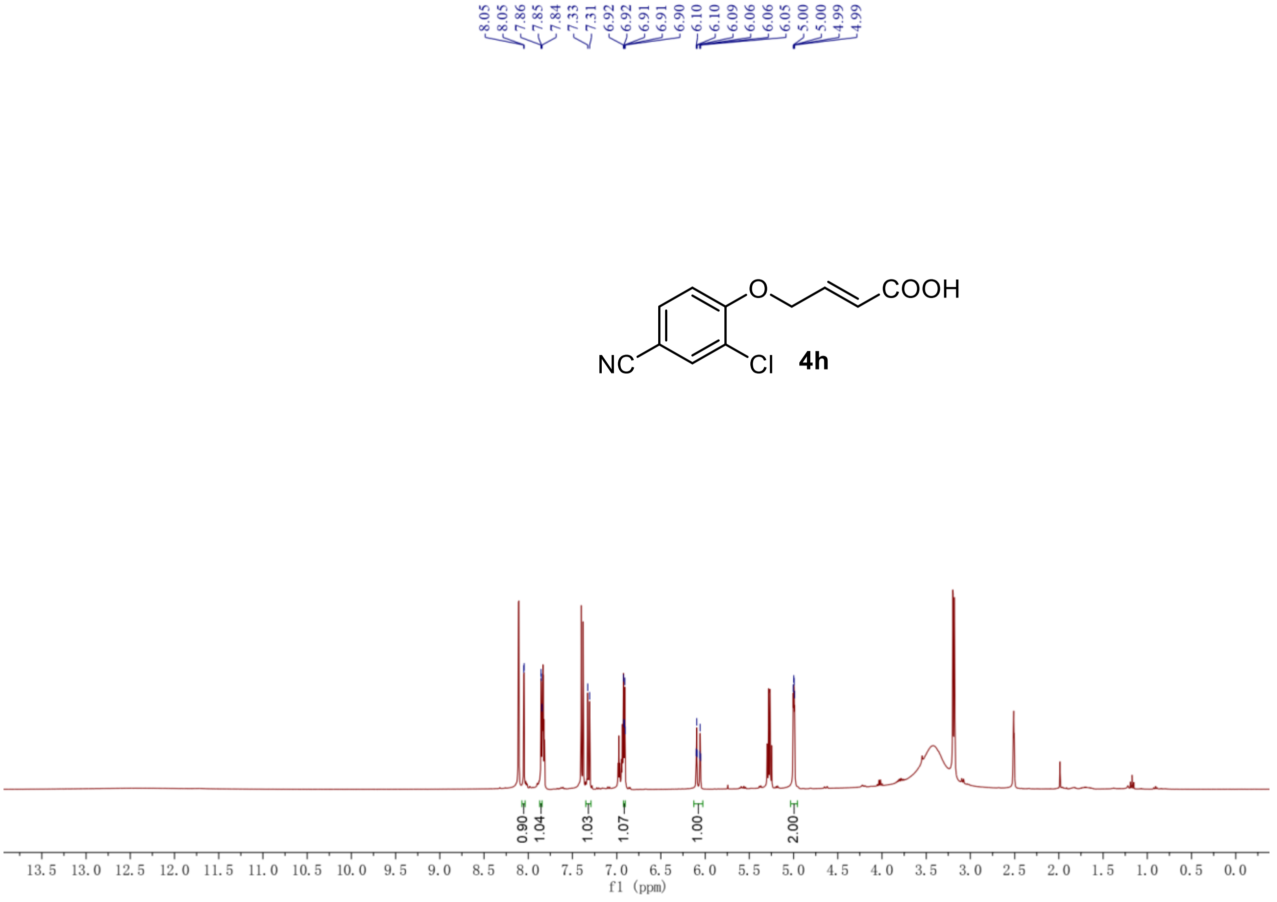
8.05 8.05 7.86 7.85 7.84 7.33 7.31 6.92 6.91 6.91 6.90 6.10 6.10 6.09 6.06 6.06 6.05 5.00 5.00 4.99 4.99
COOH
NC
Cl
4h
0.90
1.04
1.03
1.07
1.00
2.00
f1 (ppm)

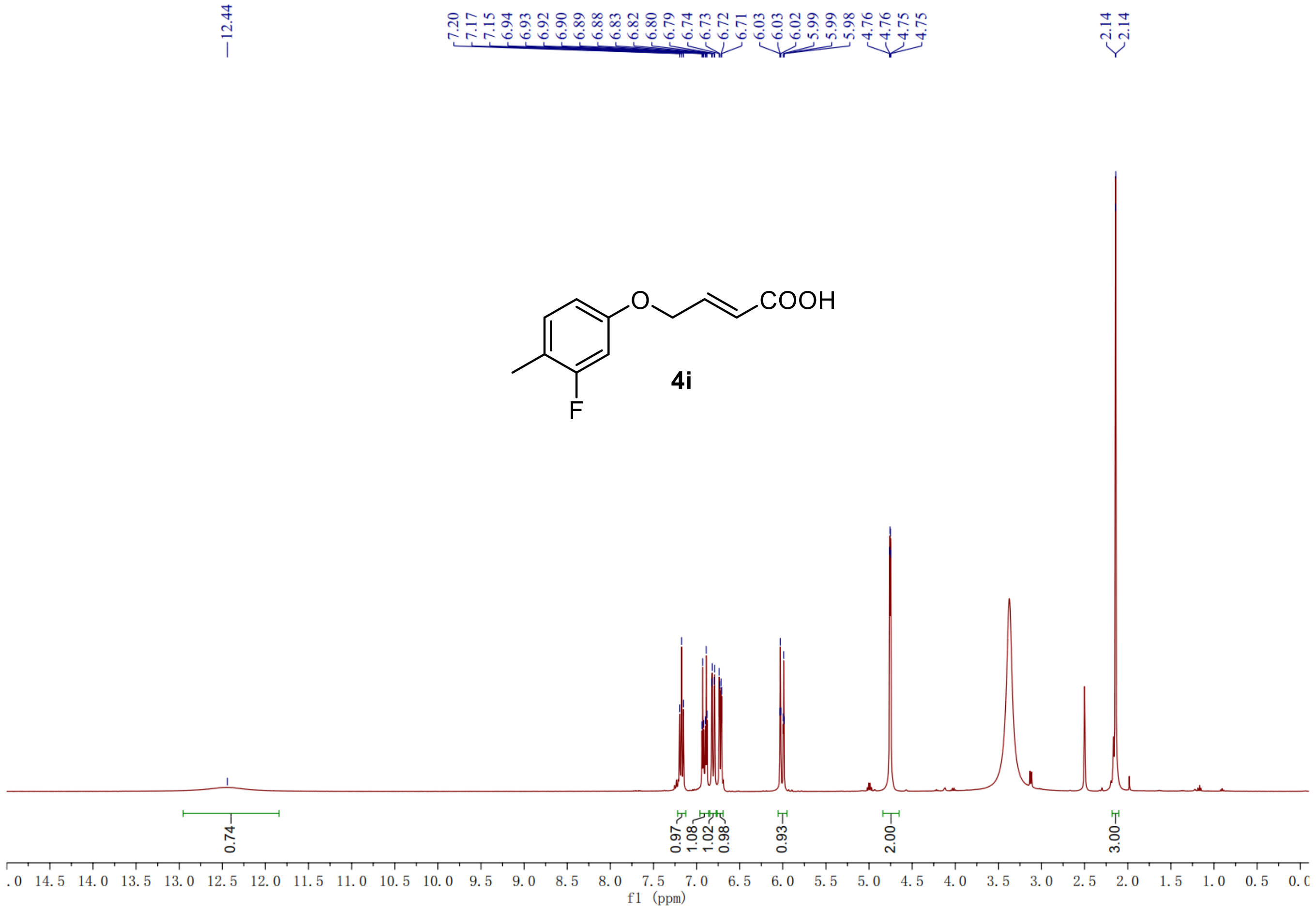
12.44
7.20 7.17 7.15 6.94 6.93 6.92 6.90 6.89 6.88 6.83 6.82 6.80 6.79 6.74 6.73 6.72 6.71 6.03 6.03 6.02 5.99 5.99 5.98 4.76 4.76 4.75 4.75
2.14 2.14
O
COOH
F
4i
0.74 0.97 1.08 1.02 0.98 0.93 2.00 3.00
f1 (ppm)

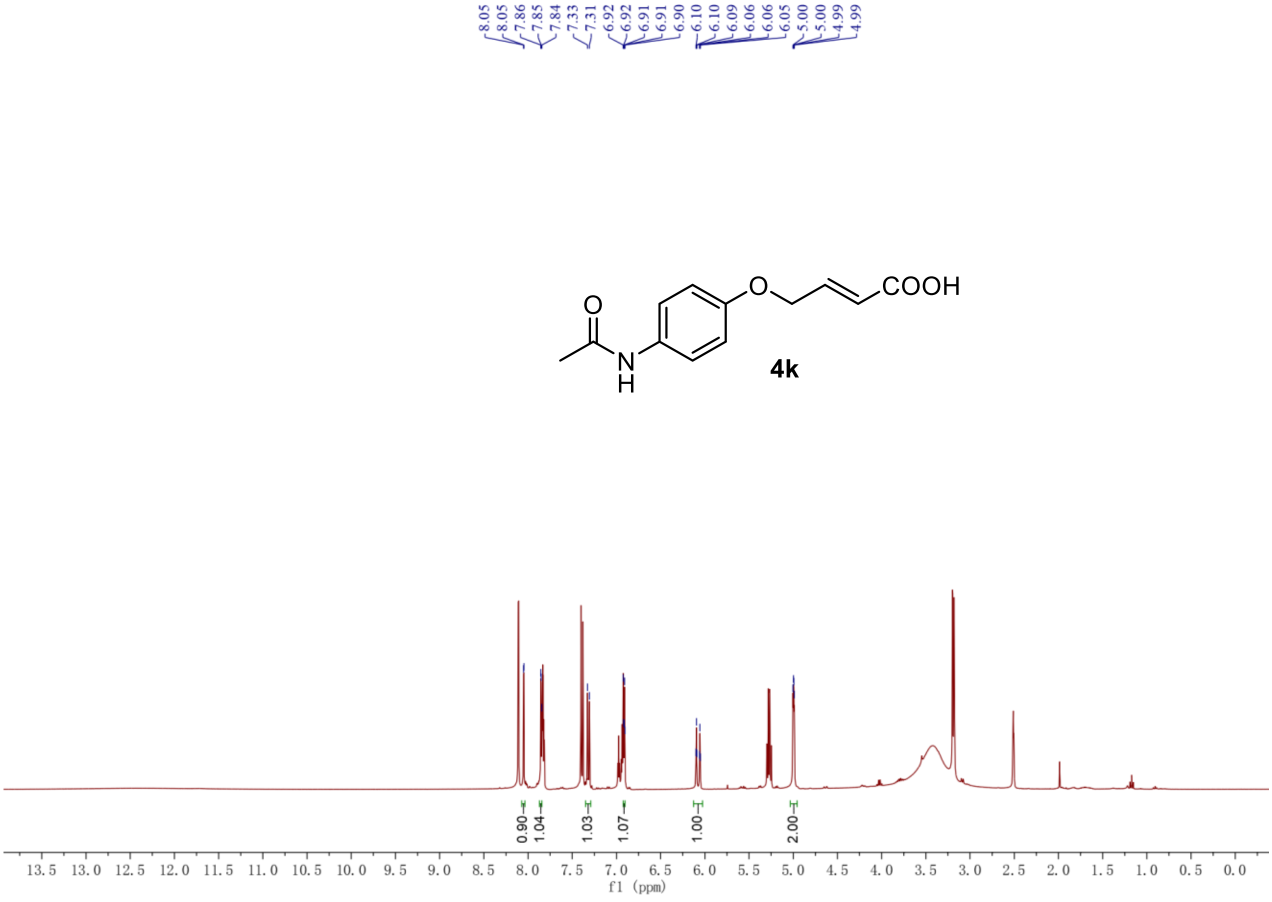
8.05 8.05 7.86 7.85 7.84 7.33 7.31 6.92 6.92 6.91 6.91 6.90 6.10 6.10 6.09 6.06 6.05 5.00 5.00 4.99 4.99
O
N
H
COOH
4k
0.90 1.04 1.03 1.07 1.00 2.00
f1 (ppm)

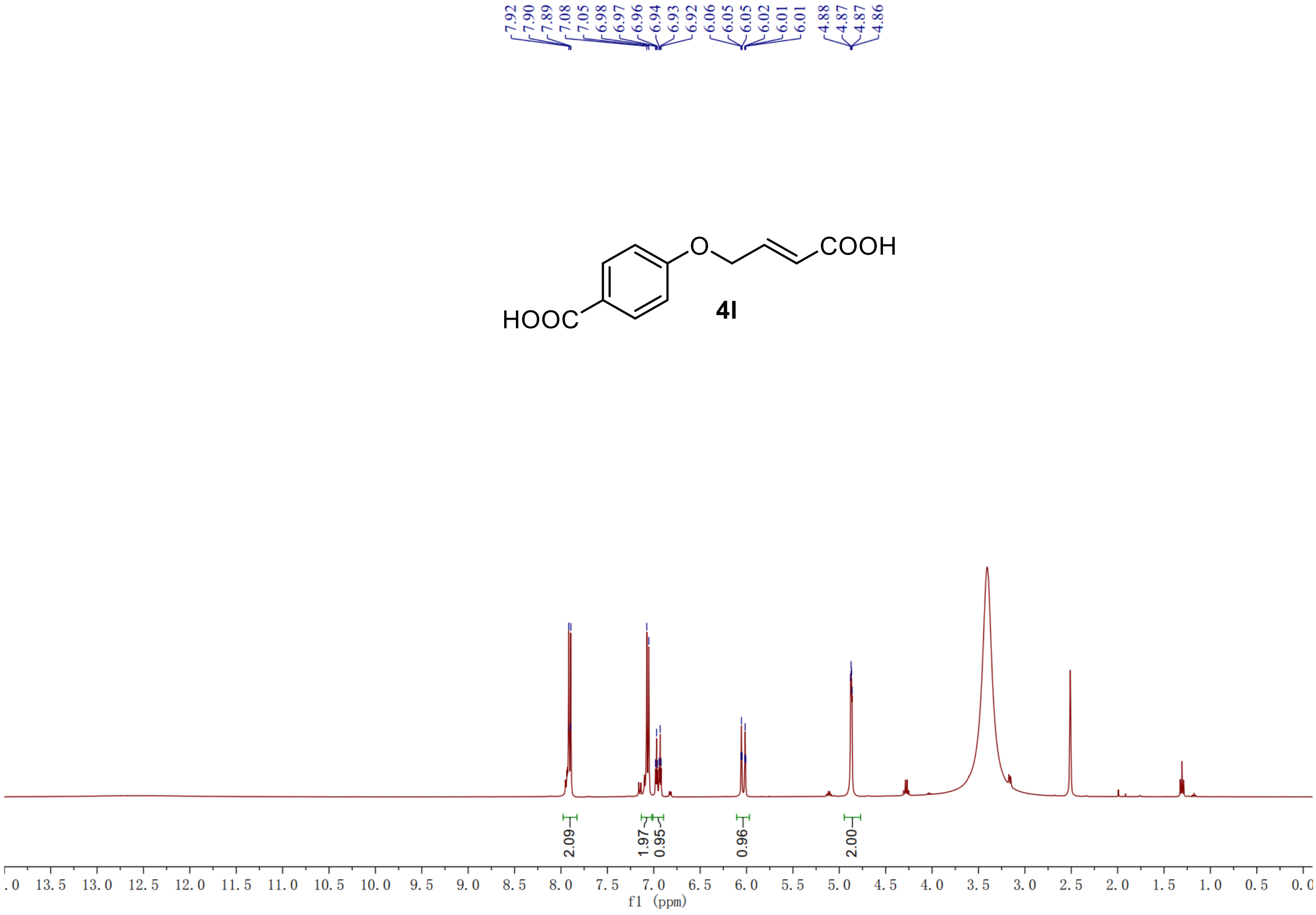
7.92
7.90
7.89
7.08
7.05
6.98
6.97
6.96
6.94
6.93
6.92
6.06
6.05
6.05
6.02
6.01
6.01
4.88
4.87
4.87
4.86
HOOC
O
COOH
4l
2.09
1.97
0.95
0.96
2.00
f1 (ppm)

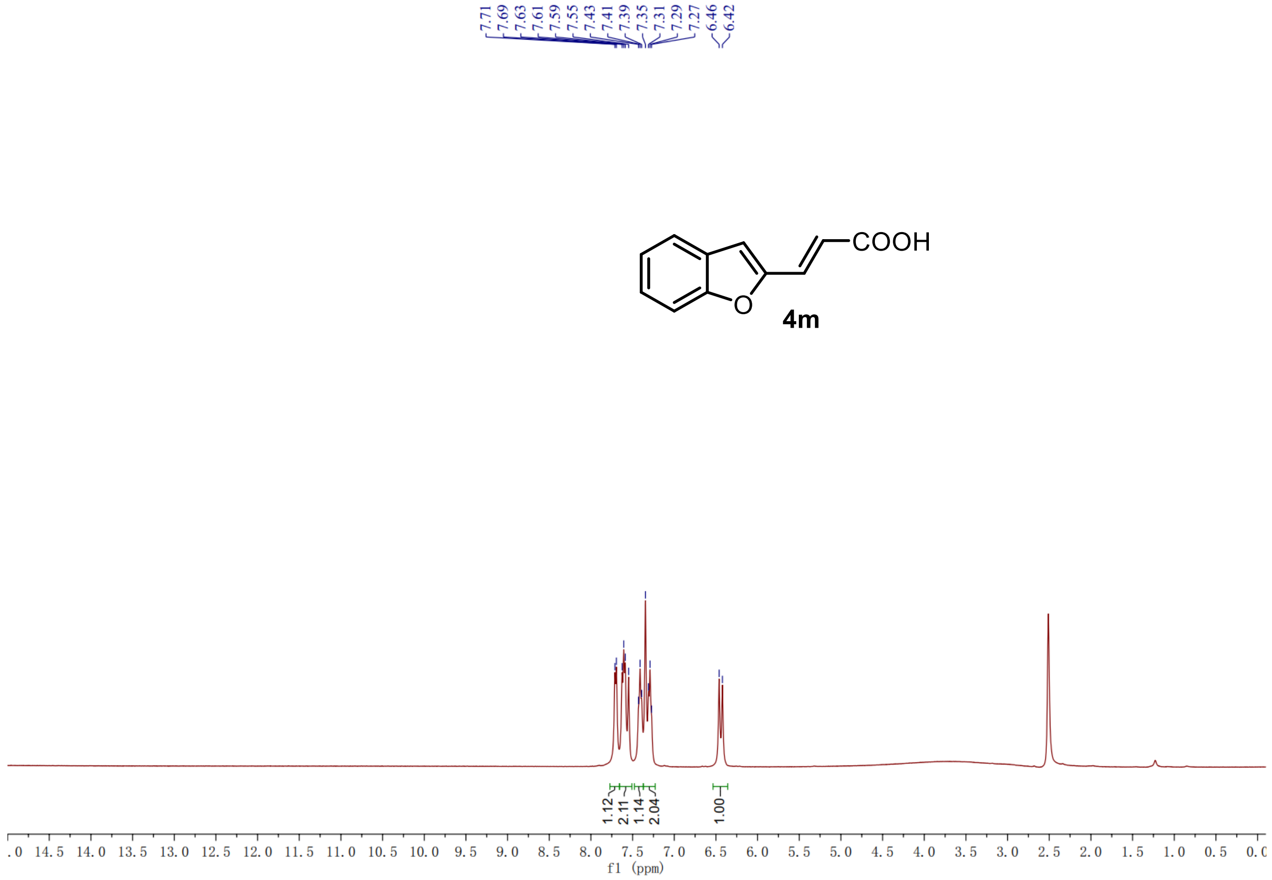
7.71
7.69
7.63
7.61
7.59
7.55
7.43
7.41
7.39
7.35
7.31
7.29
7.27
6.46
6.42
O
COOH
4m
1.12
2.11
1.14
2.04
1.00
f1 (ppm)

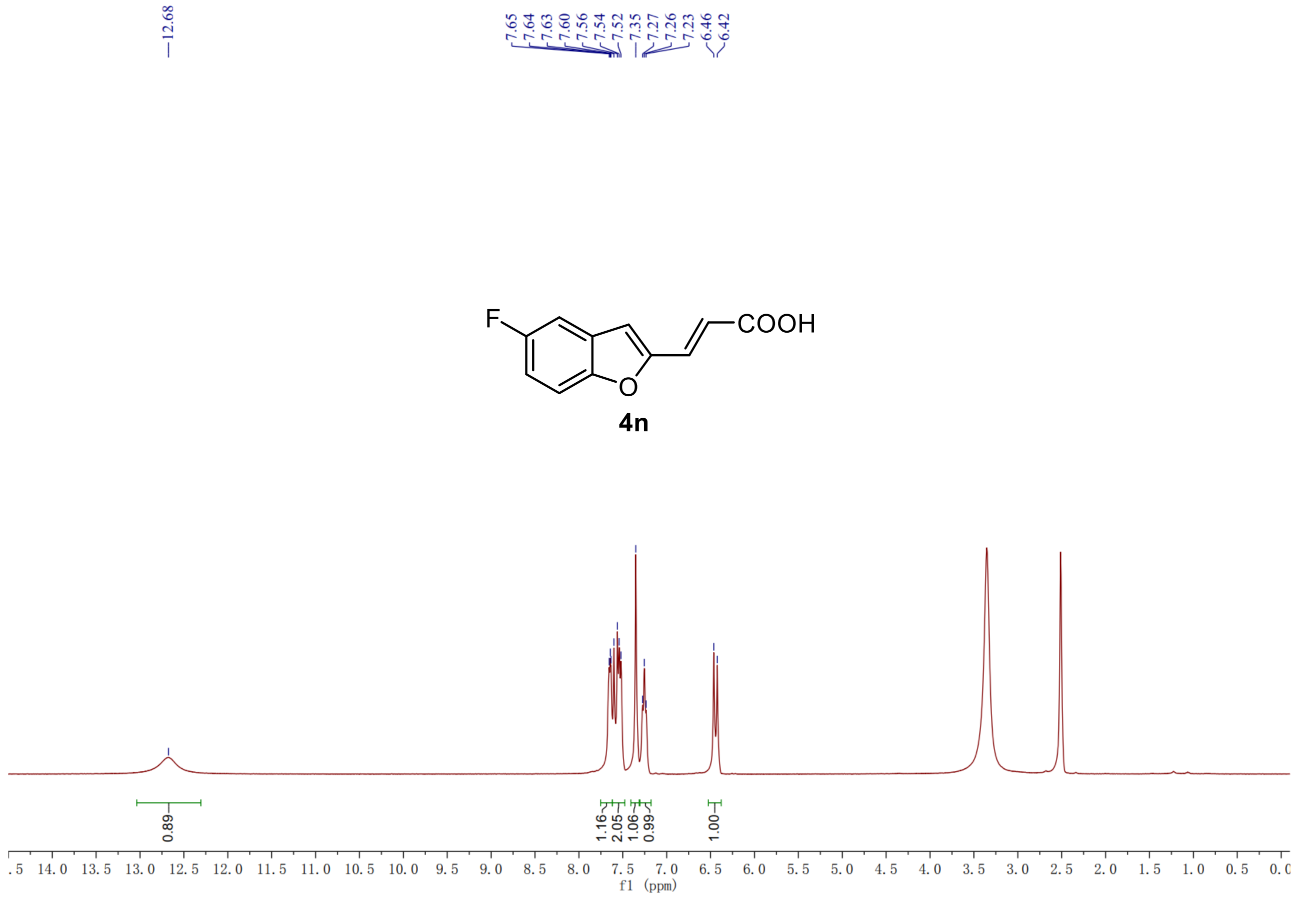
12.68
7.65 7.64 7.63 7.60 7.56 7.54 7.52 7.35 7.27 7.26 7.23 6.46 6.42
F
COOH
O
4n
0.89
1.16 2.05 1.06 0.99
1.00
.5 14.0 13.5 13.0 12.5 12.0 11.5 11.0 10.5 10.0 9.5 9.0 8.5 8.0 7.5 7.0 6.5 6.0 5.5 5.0 4.5 4.0 3.5 3.0 2.5 2.0 1.5 1.0 0.5 0.0
f1 (ppm)

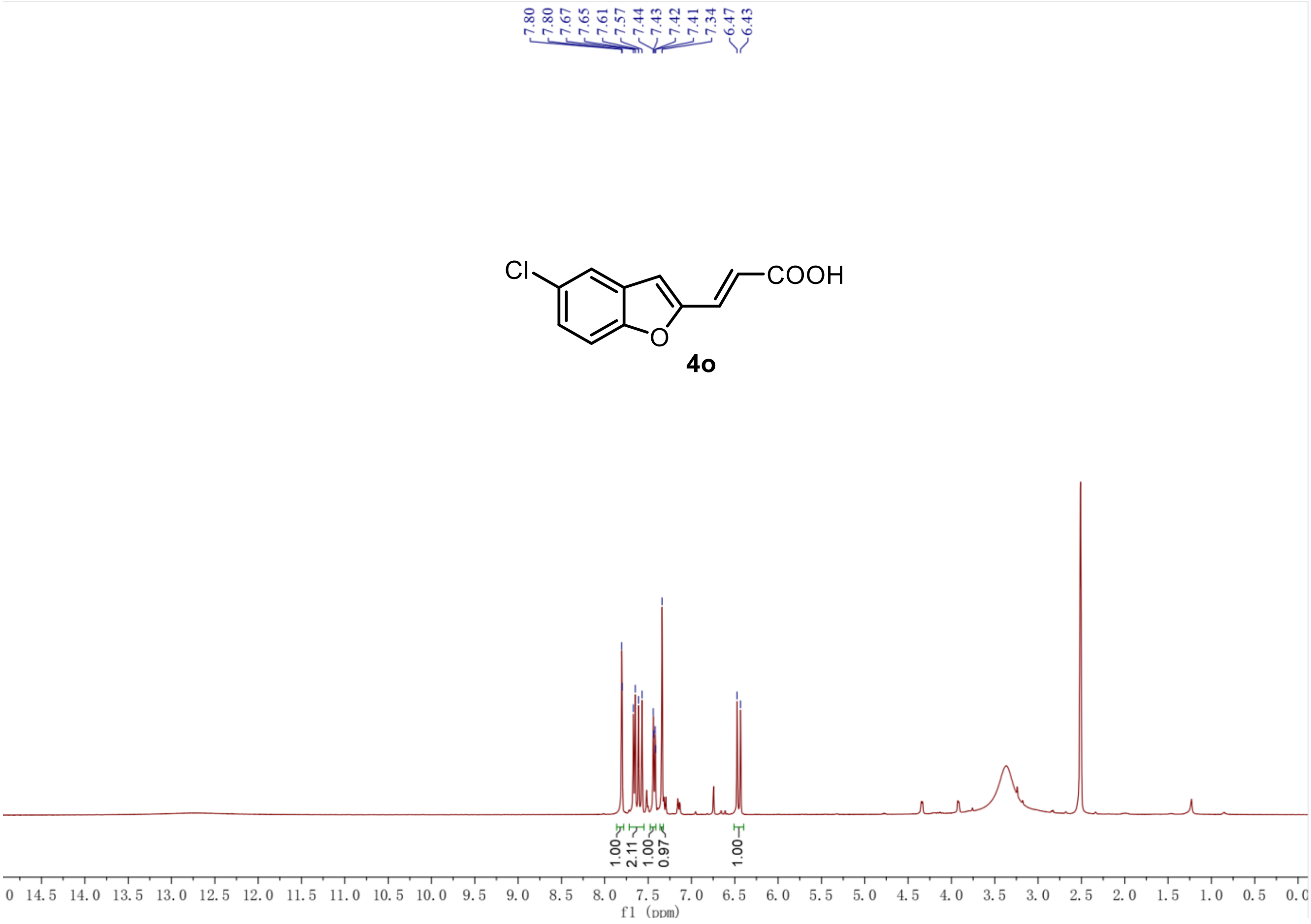
7.80 7.80 7.67 7.65 7.61 7.57 7.44 7.43 7.42 7.41 7.34 6.47 6.43
Cl
COOH
O
4o
1.00 2.11 1.00 0.97
1.00
0 14.5 14.0 13.5 13.0 12.5 12.0 11.5 11.0 10.5 10.0 9.5 9.0 8.5 8.0 7.5 7.0 6.5 6.0 5.5 5.0 4.5 4.0 3.5 3.0 2.5 2.0 1.5 1.0 0.5 0.0
f1 (ppm)

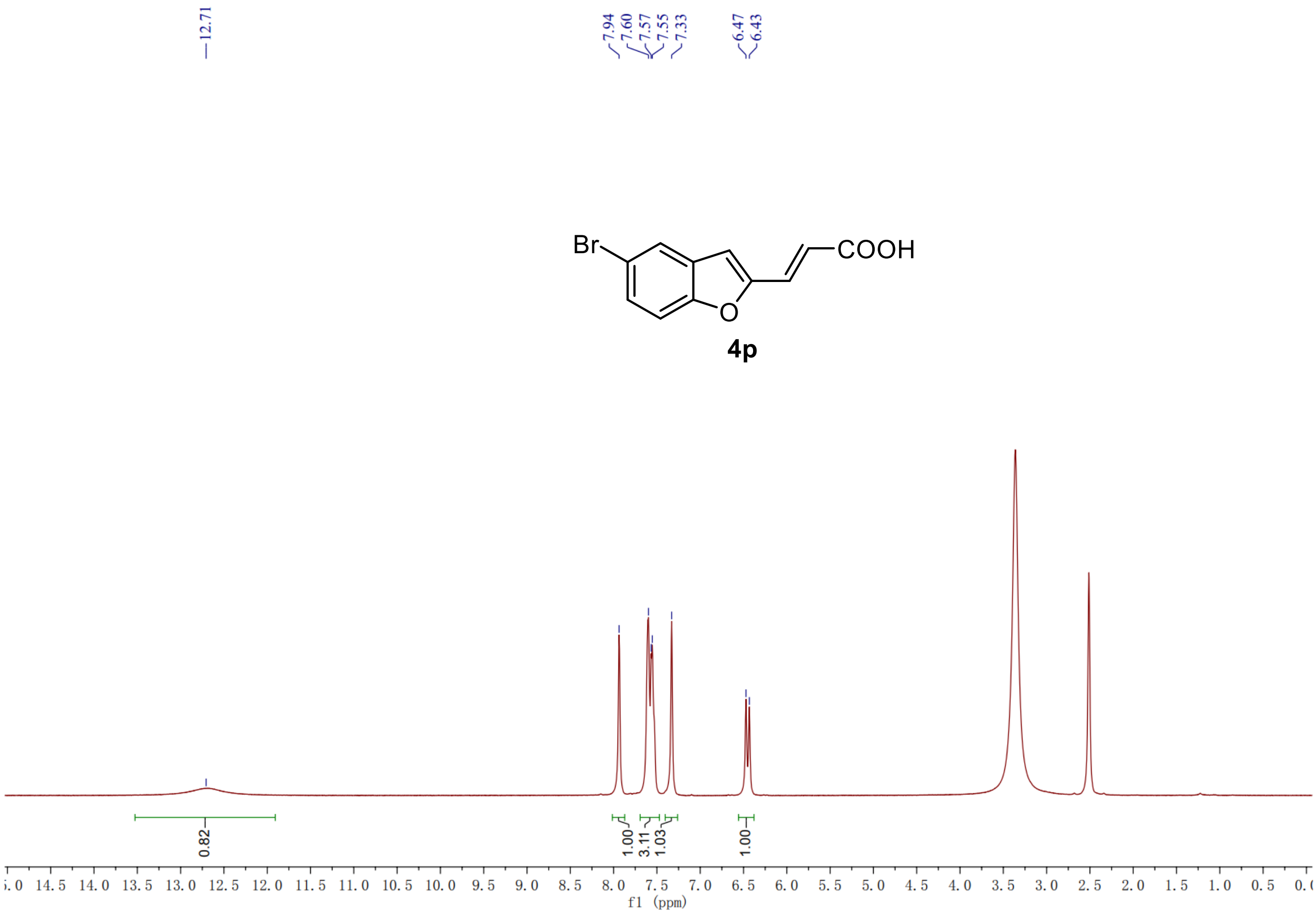
12.71
7.94
7.60
7.57
7.55
7.33
6.47
6.43
Br
COOH
O
4p
0.82
1.00
3.11
1.03
1.00
f1 (ppm)

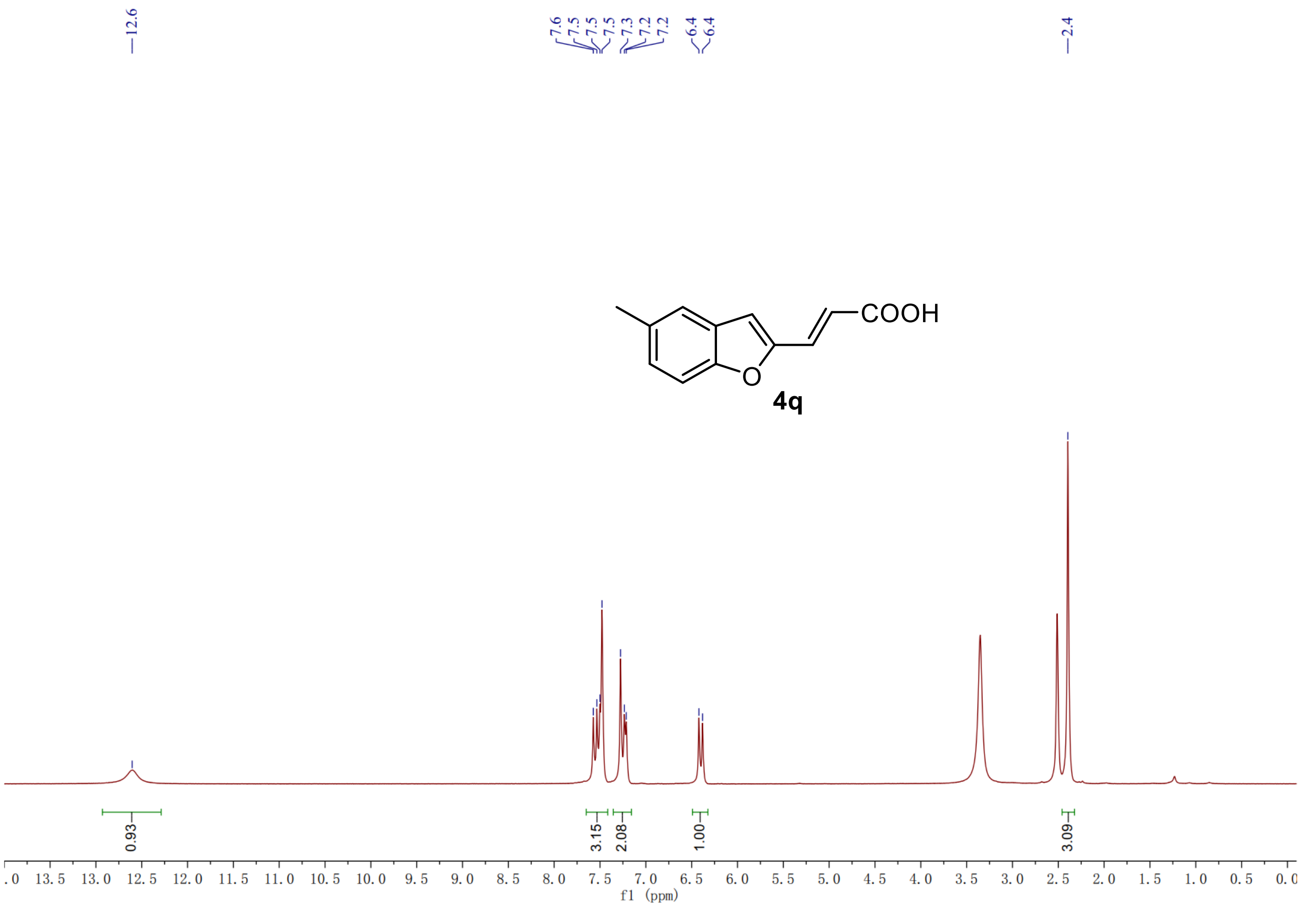
12.6
7.6
7.5
7.5
7.5
7.3
7.2
7.2
6.4
6.4
2.4
COOH
O
4q
0.93
3.15
2.08
1.00
3.09
f1 (ppm)

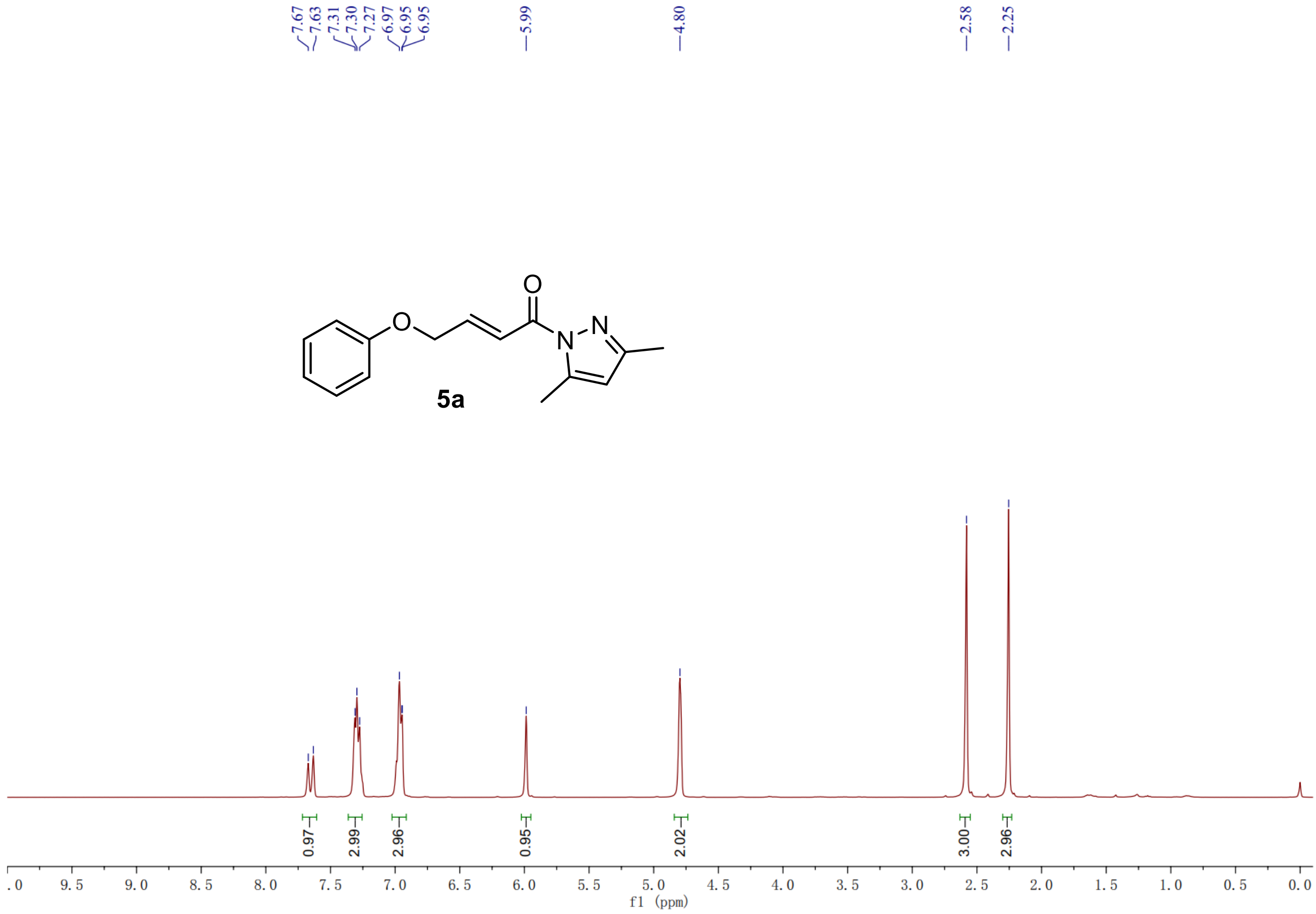
7.67
7.63
7.31
7.30
7.27
6.97
6.95
6.95
5.99
4.80
2.58
2.25
O
O
N
N
5a
0.97
2.99
2.96
0.95
2.02
3.00
2.96
f1 (ppm)

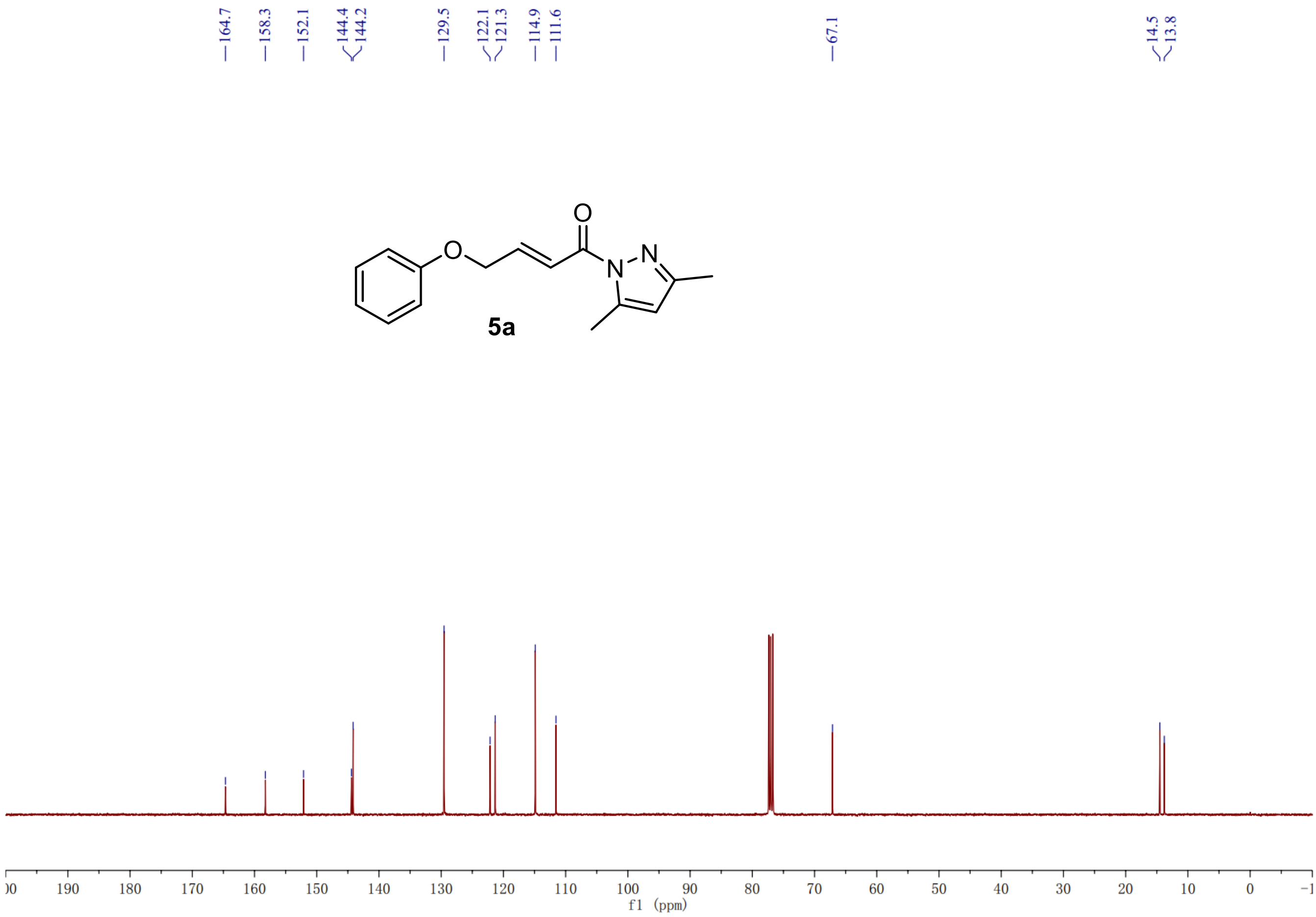
164.7
158.3
152.1
144.4
144.2
129.5
122.1
121.3
114.9
111.6
67.1
14.5
13.8
O
O
N
N
5a
f1 (ppm)

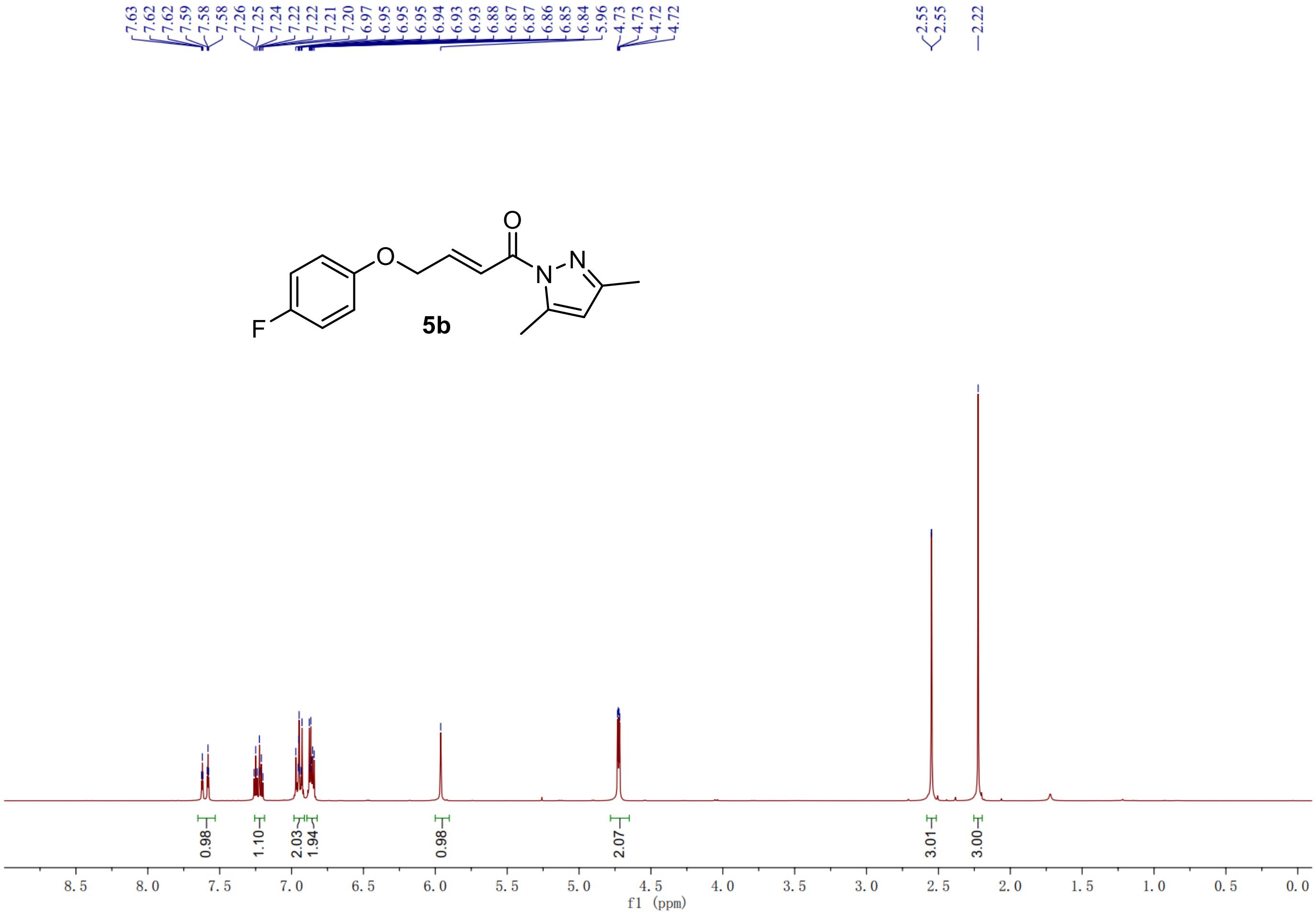
7.63 7.62 7.62 7.59 7.58 7.58 7.26 7.25 7.24 7.22 7.22 7.21 7.20 6.97 6.95 6.95 6.95 6.94 6.93 6.93 6.88 6.87 6.87 6.86 6.85 6.84 5.96 4.73 4.73 4.72 4.72
2.55 2.55
2.22
O
O
N
N
F
5b
0.98
1.10
2.03
1.94
0.98
2.07
3.01
3.00
8.5 8.0 7.5 7.0 6.5 6.0 5.5 5.0 4.5 4.0 3.5 3.0 2.5 2.0 1.5 1.0 0.5 0.0
f1 (ppm)

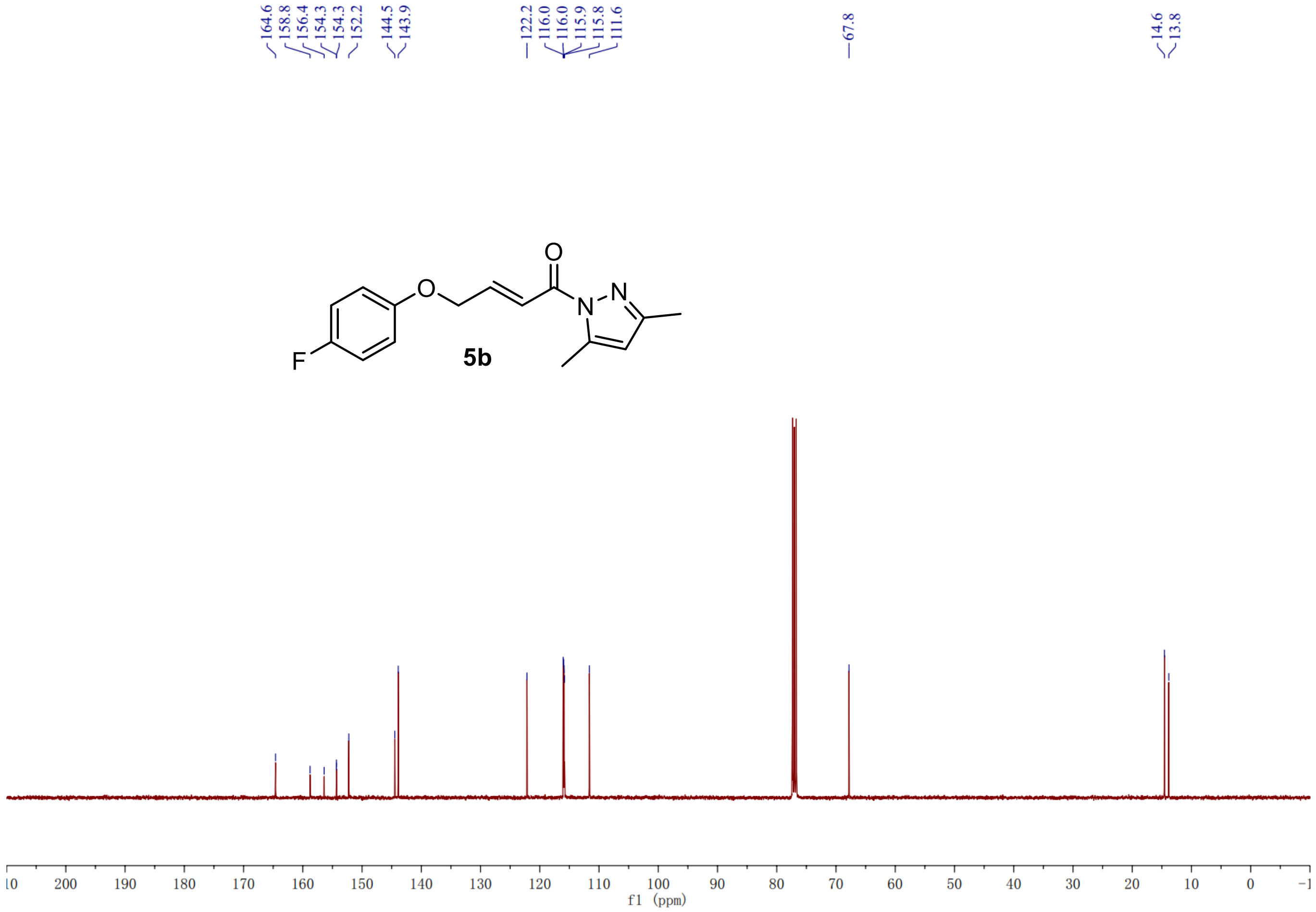
164.6 158.8 156.4 154.3 154.3 152.2 144.5 143.9
122.2 116.0 116.0 115.9 115.8 111.6
67.8
14.6 13.8
O
O
N
N
F
5b
200 190 180 170 160 150 140 130 120 110 100 90 80 70 60 50 40 30 20 10 0
f1 (ppm)

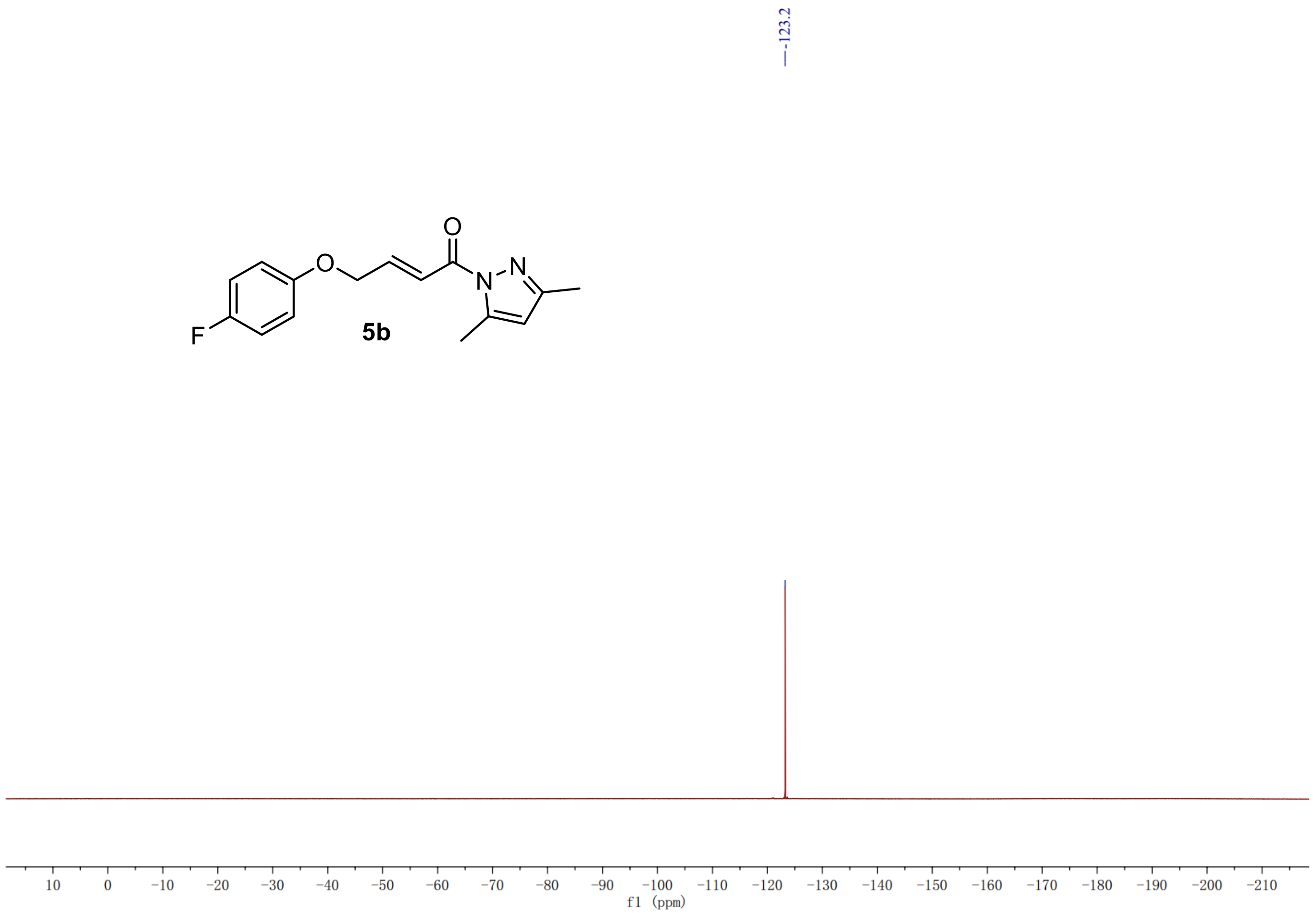
-123.2
F
O
O
N
N
5b
f1 (ppm)

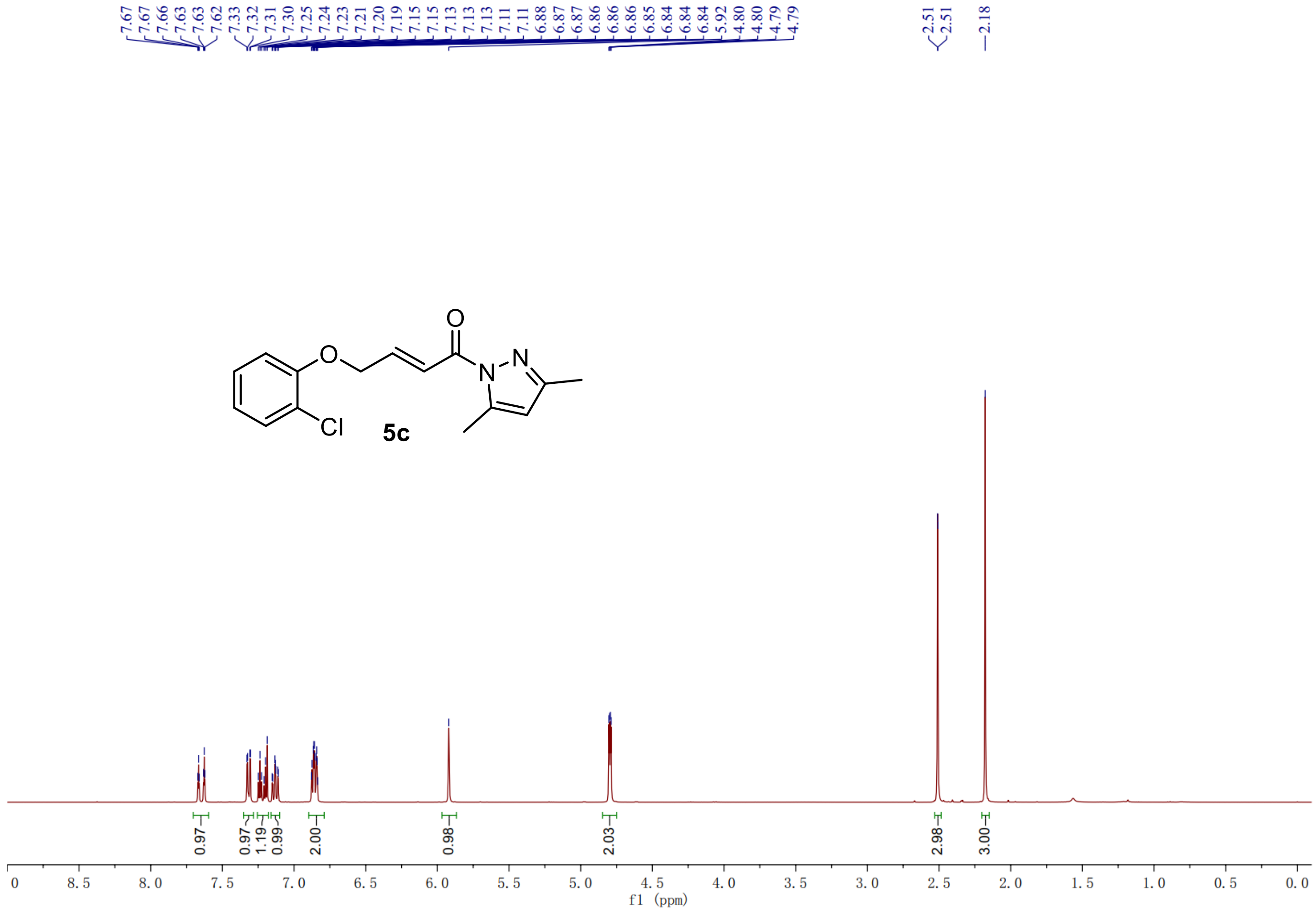
7.67 7.67 7.66 7.63 7.63 7.62 7.33 7.32 7.31 7.30 7.25 7.24 7.23 7.21 7.20 7.19 7.15 7.15 7.13 7.13 7.13 7.11 7.11 6.88 6.87 6.87 6.86 6.86 6.86 6.85 6.84 6.84 6.84 5.92 4.80 4.80 4.79 4.79
2.51 2.51
2.18
O
O
N
N
Cl
5c
0.97
0.97
1.19
0.99
2.00
0.98
2.03
2.98
3.00
f1 (ppm)

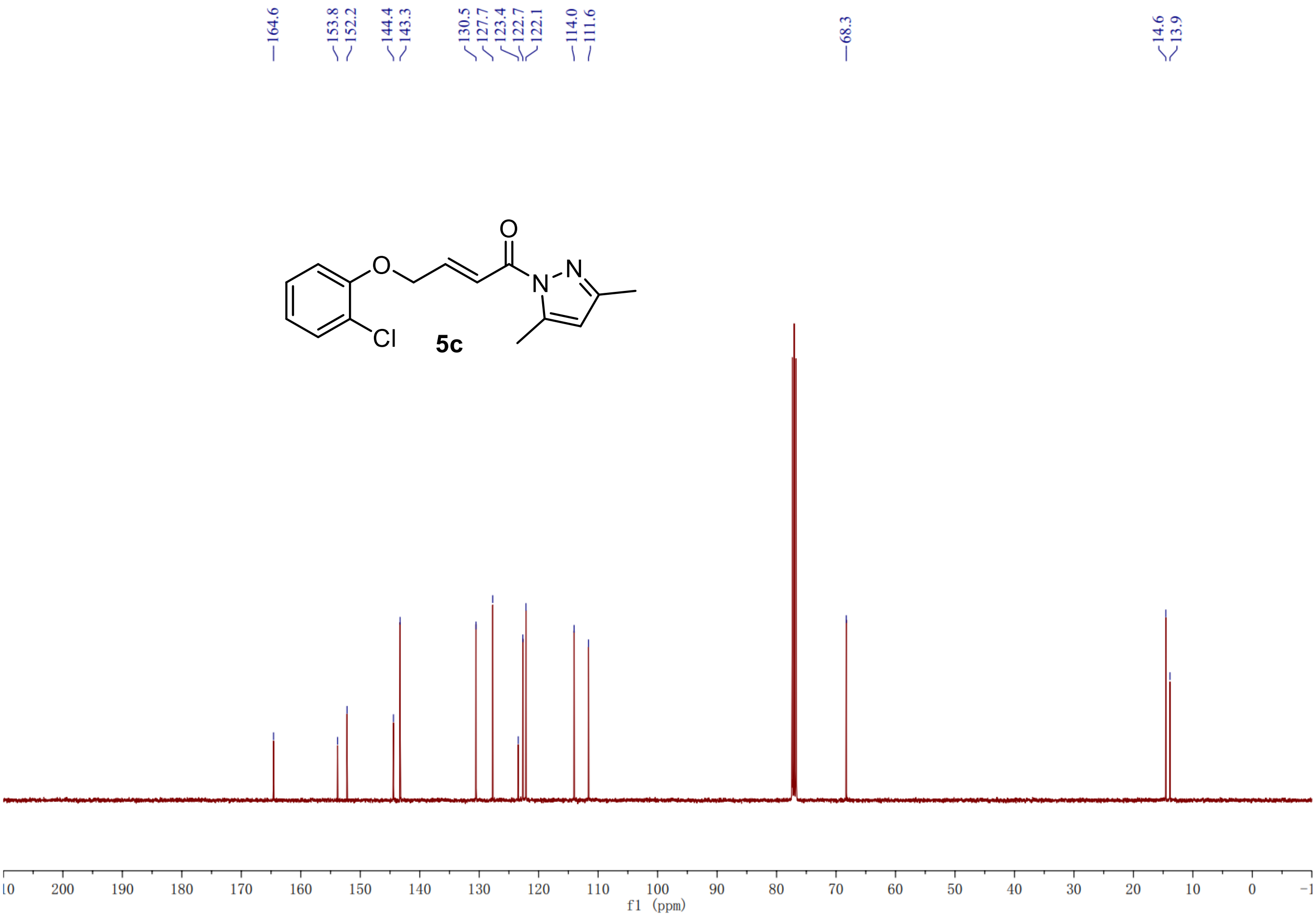
164.6
153.8
152.2
144.4
143.3
130.5
127.7
123.4
122.7
122.1
114.0
111.6
68.3
14.6
13.9
O
O
N
N
Cl
5c
f1 (ppm)

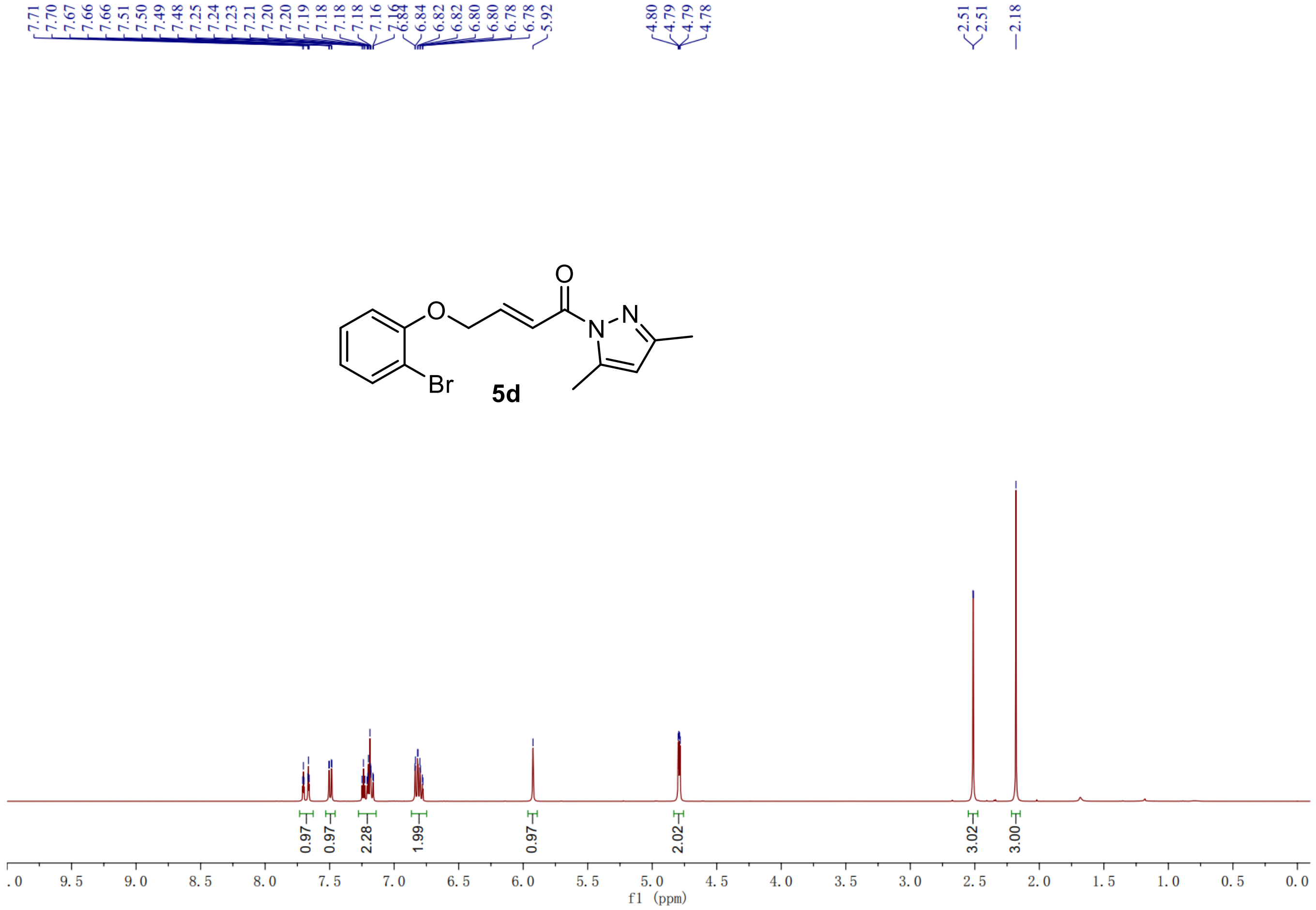
7.71 7.70 7.67 7.66 7.66 7.51 7.50 7.49 7.48 7.25 7.24 7.23 7.21 7.20 7.20 7.19 7.18 7.18 7.18 7.16 7.16 6.84 6.84 6.82 6.82 6.80 6.80 6.78 6.78 5.92
4.80 4.79 4.79 4.78
2.51 2.51
2.18
O
O
N
N
Br
5d
0.97
0.97
2.28
1.99
0.97
2.02
3.02
3.00
f1 (ppm)

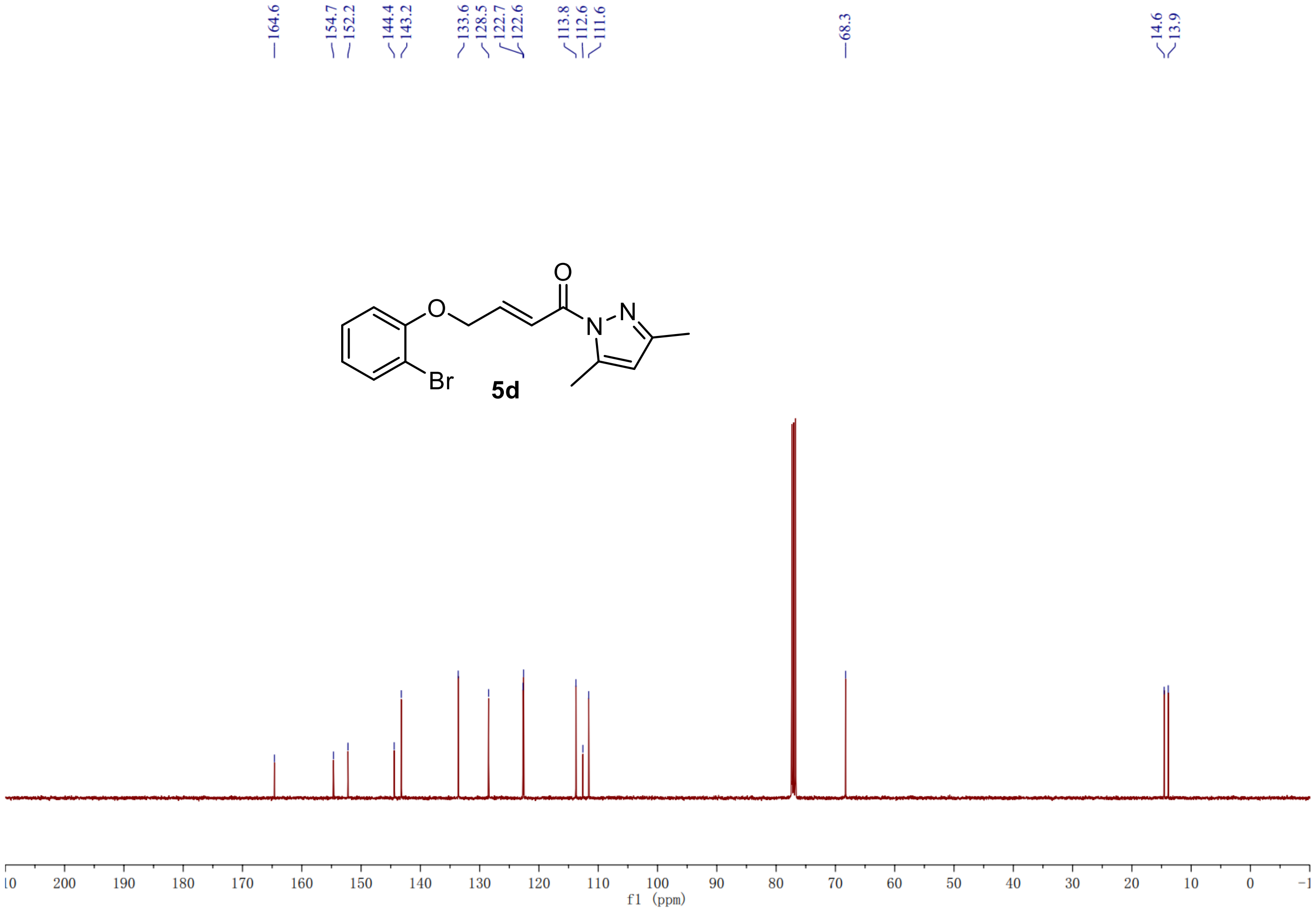
164.6
154.7
152.2
144.4
143.2
133.6
128.5
122.7
122.6
113.8
112.6
111.6
68.3
14.6
13.9
O
O
N
N
Br
5d
f1 (ppm)

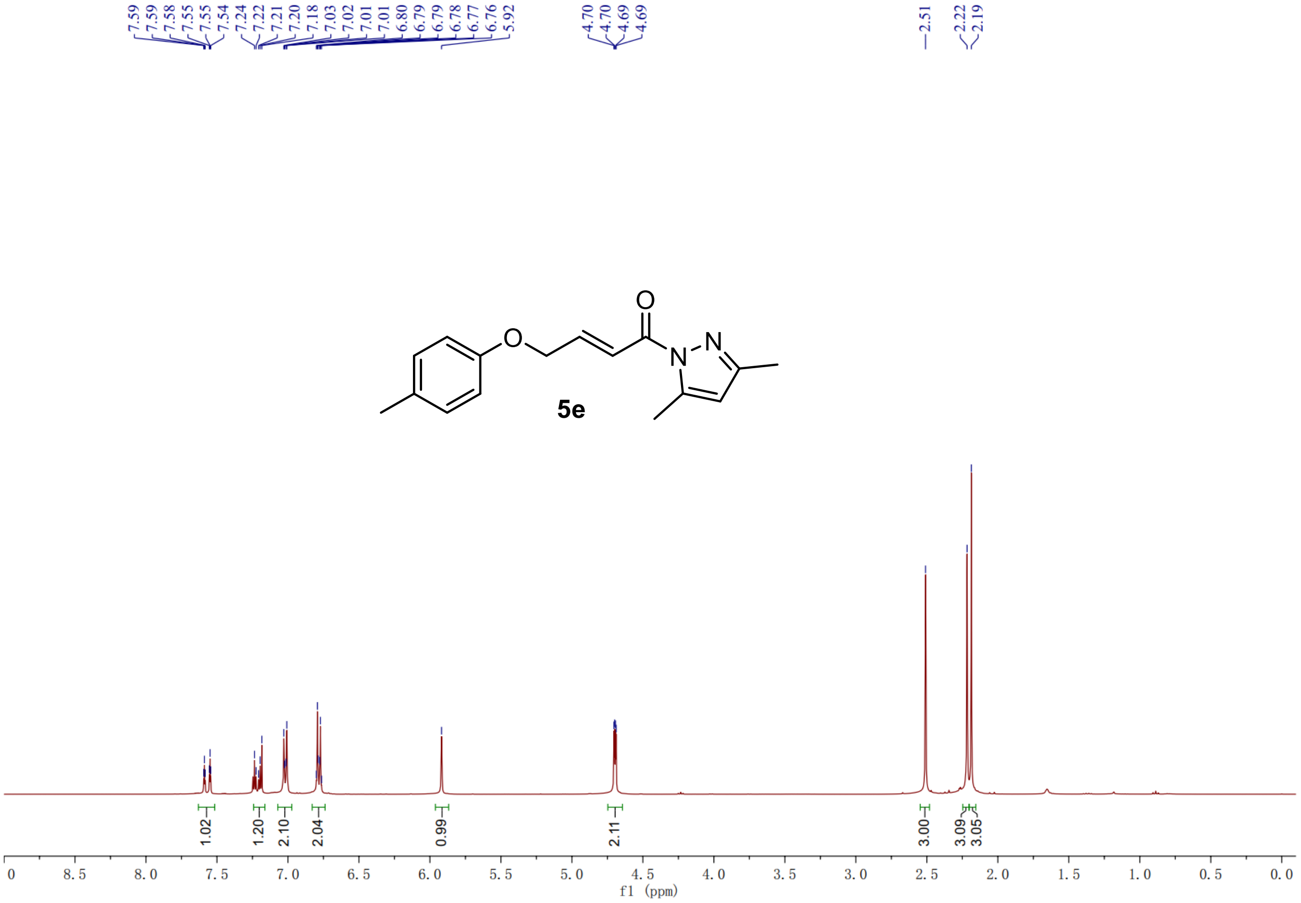
7.59
7.59
7.58
7.55
7.55
7.54
7.24
7.22
7.21
7.20
7.18
7.03
7.02
7.01
7.01
6.80
6.79
6.79
6.78
6.77
6.76
5.92
4.70
4.70
4.69
4.69
2.51
2.22
2.19
O
O
N
N
5e
1.02
1.20
2.10
2.04
0.99
2.11
3.00
3.09
3.05
f1 (ppm)

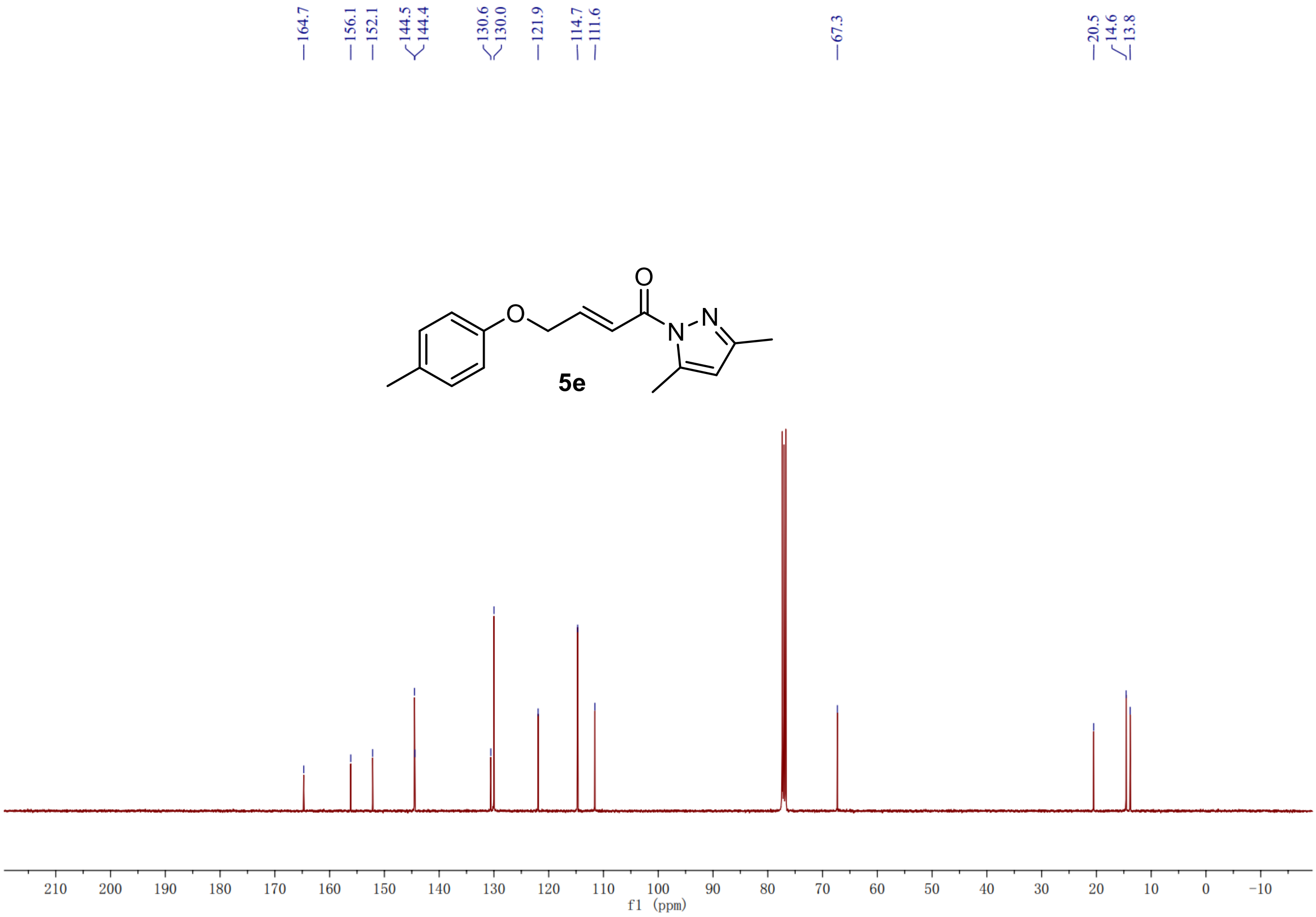

164.7
156.1
152.1
144.5
144.4
130.6
130.0
121.9
114.7
111.6
67.3
20.5
14.6
13.8
5e
f1 (ppm)

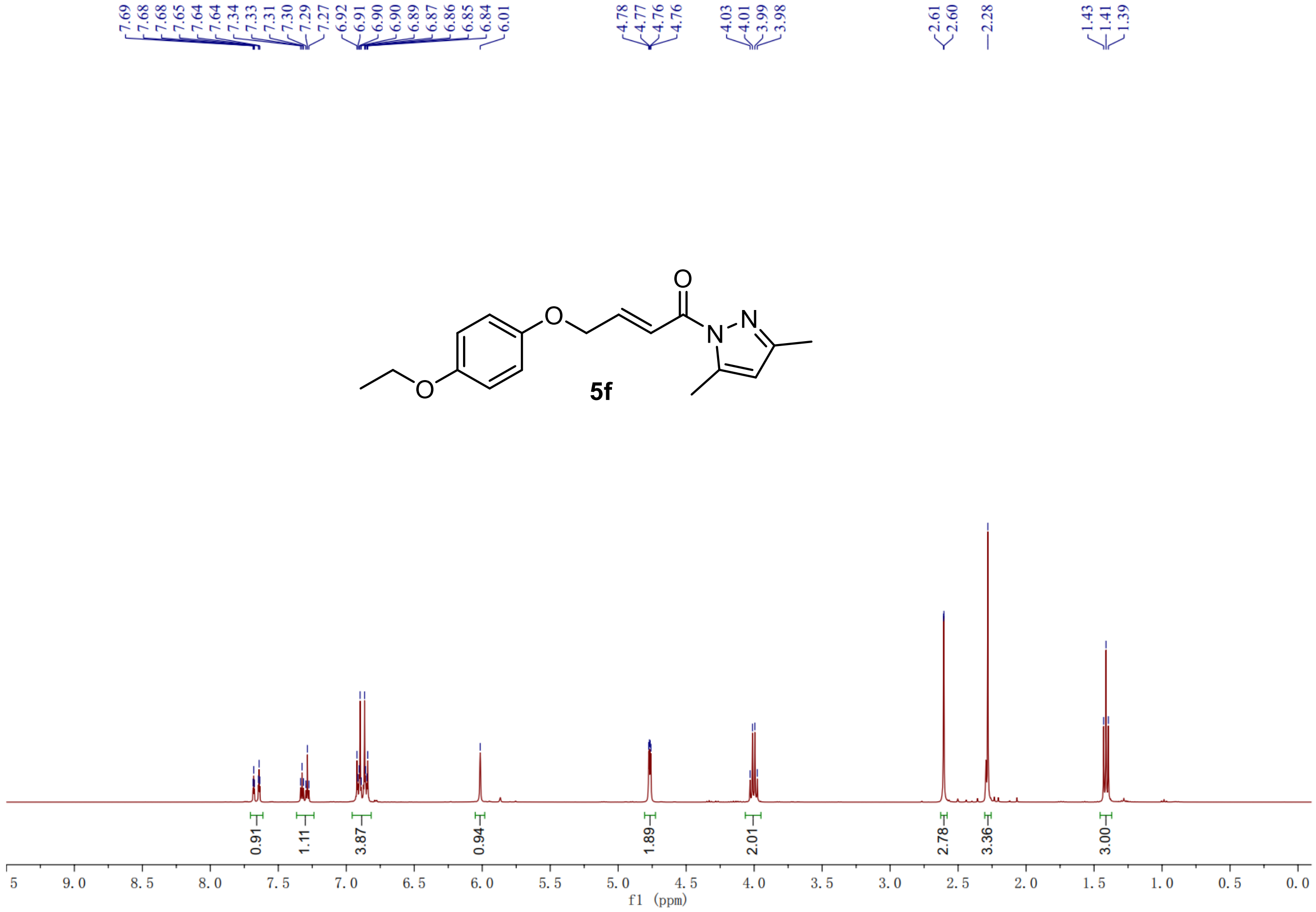

7.69
7.68
7.68
7.65
7.64
7.64
7.34
7.33
7.31
7.30
7.29
7.27
6.92
6.91
6.90
6.90
6.89
6.87
6.86
6.85
6.84
6.01
4.78
4.77
4.76
4.76
4.03
4.01
3.99
3.98
2.61
2.60
2.28
1.43
1.41
1.39
5f
0.91
1.11
3.87
0.94
1.89
2.01
2.78
3.36
3.00
f1 (ppm)

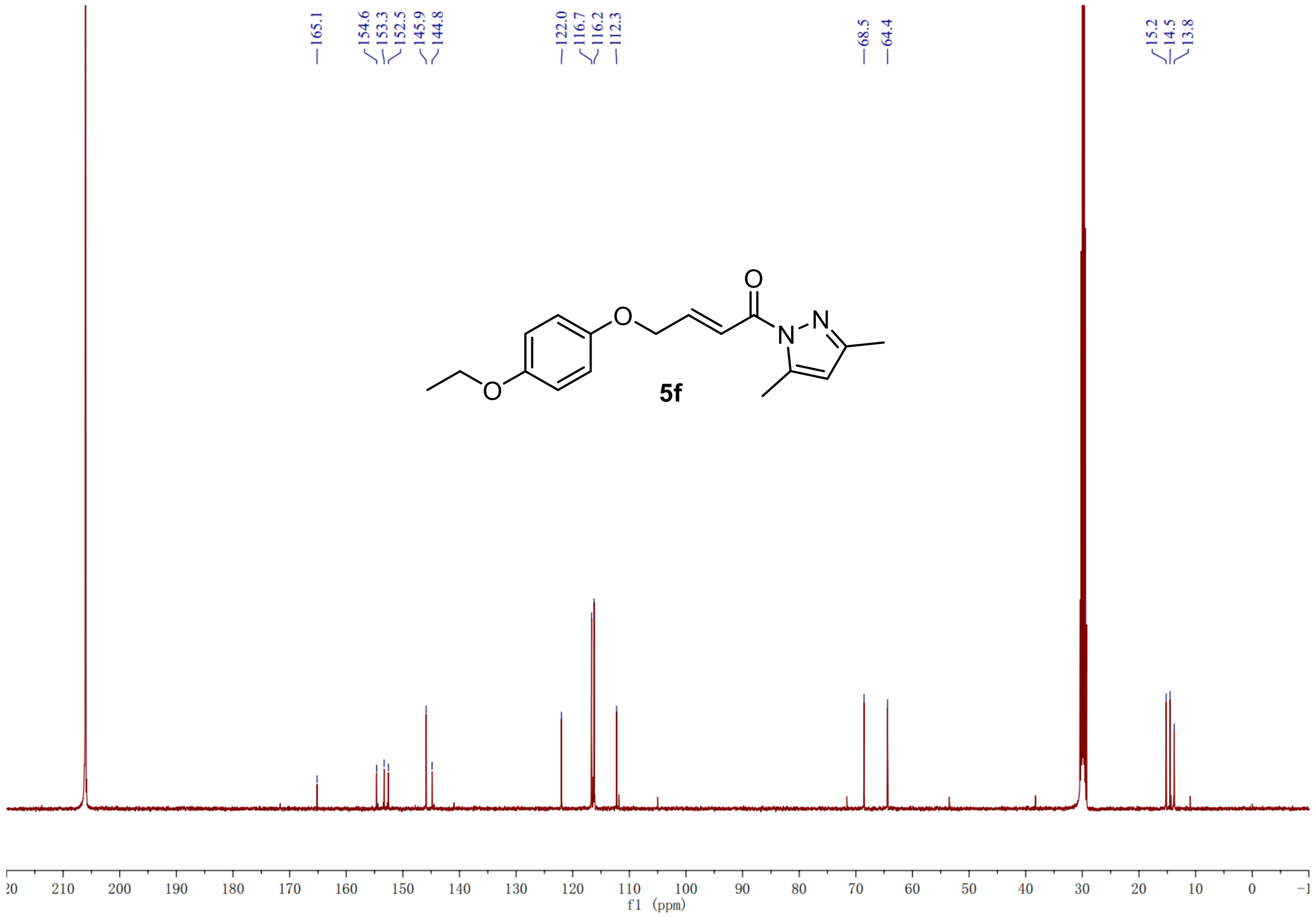
165.1
154.6
153.3
152.5
145.9
144.8
122.0
116.7
116.2
112.3
68.5
64.4
15.2
14.5
13.8
5f
f1 (ppm)

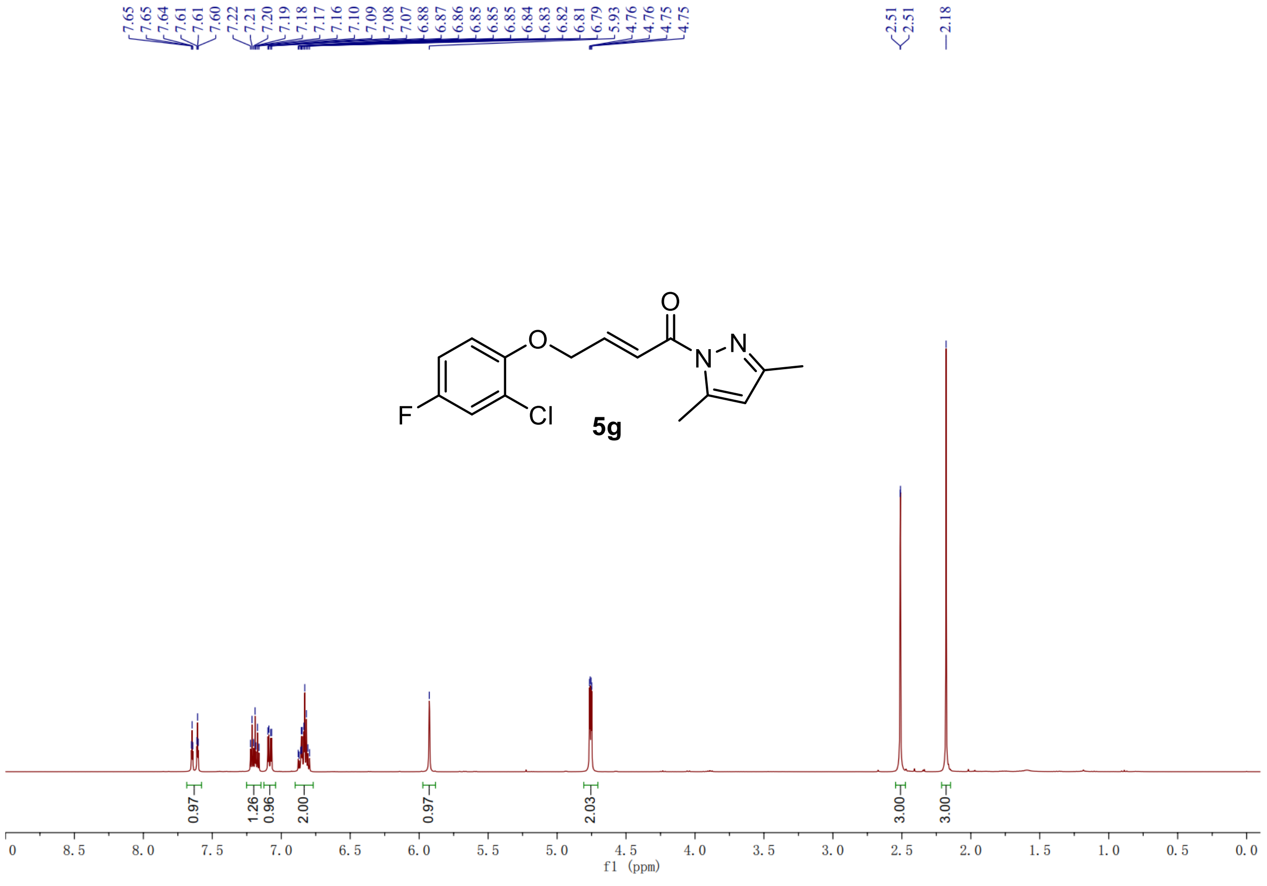
7.65
7.65
7.64
7.61
7.61
7.60
7.22
7.21
7.20
7.19
7.18
7.17
7.16
7.10
7.09
7.08
7.07
6.88
6.87
6.86
6.85
6.85
6.85
6.84
6.83
6.82
6.81
6.79
5.93
4.76
4.76
4.75
4.75
2.51
2.51
2.18
5g
0.97
1.26
0.96
2.00
0.97
2.03
3.00
3.00
f1 (ppm)

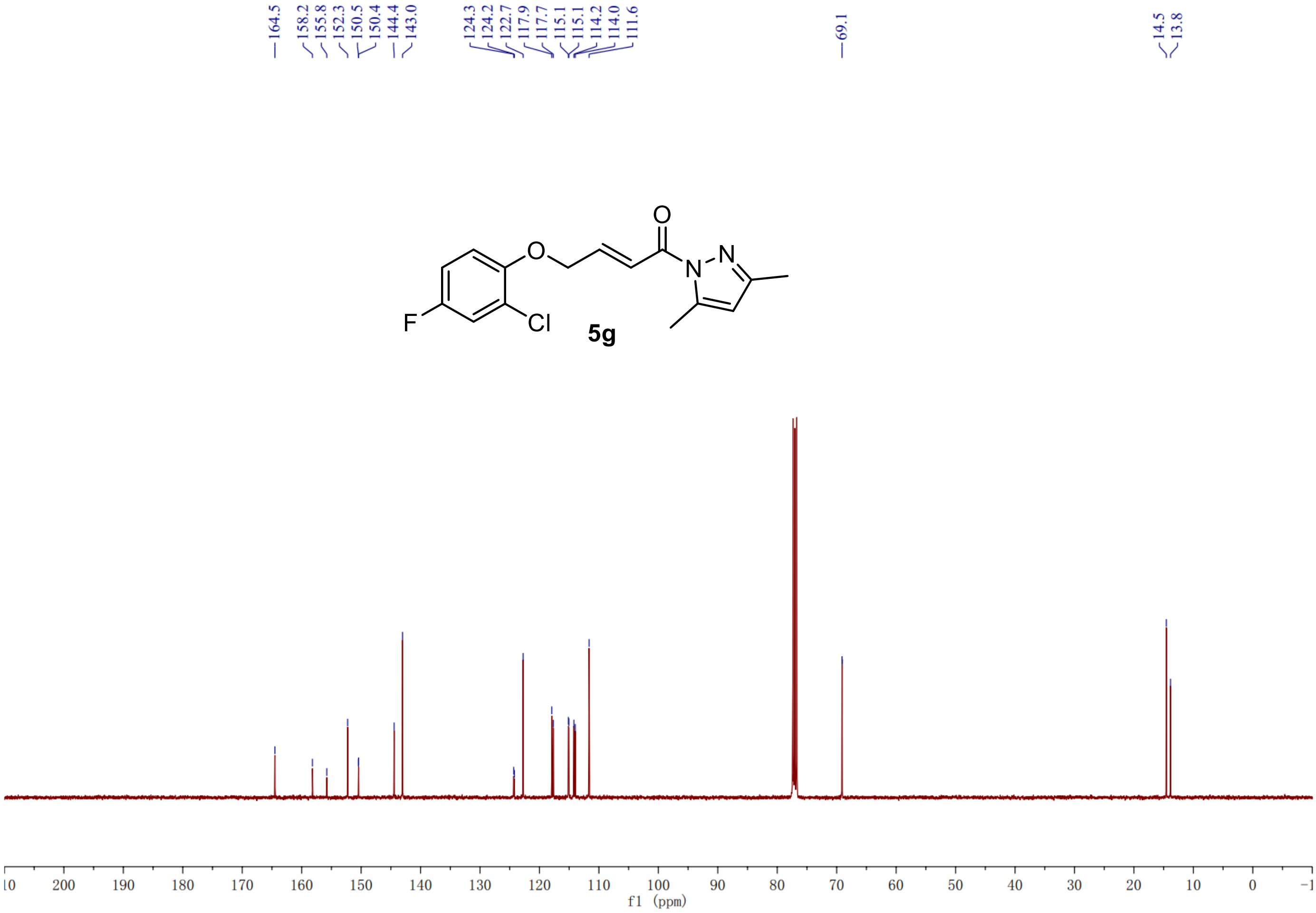
164.5
158.2
155.8
152.3
150.5
150.4
144.4
143.0
124.3
124.2
122.7
117.9
117.7
115.1
115.1
114.2
114.0
111.6
69.1
14.5
13.8
O
N
N
F
Cl
5g
f1 (ppm)

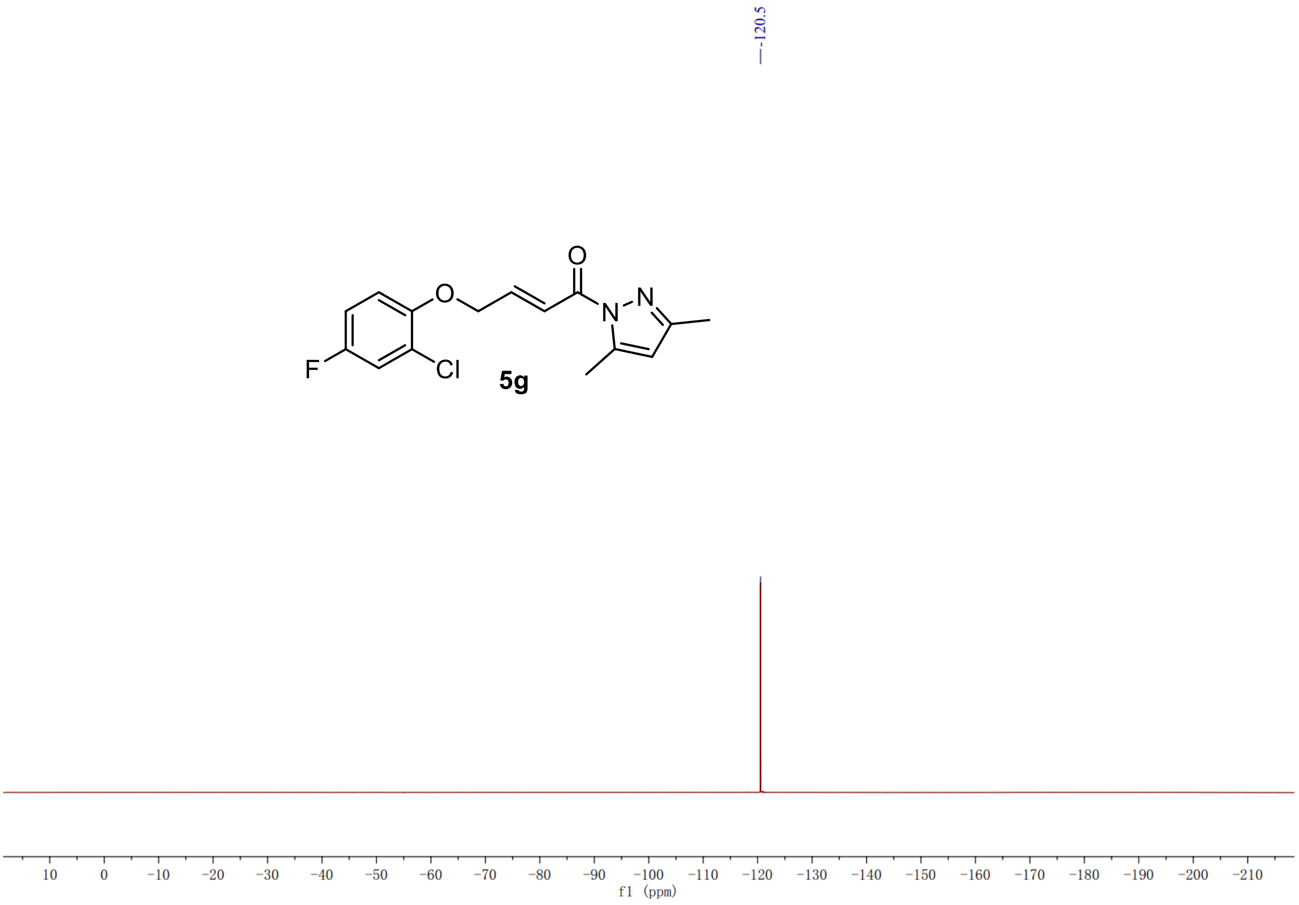
-120.5
O
N
N
F
Cl
5g
f1 (ppm)

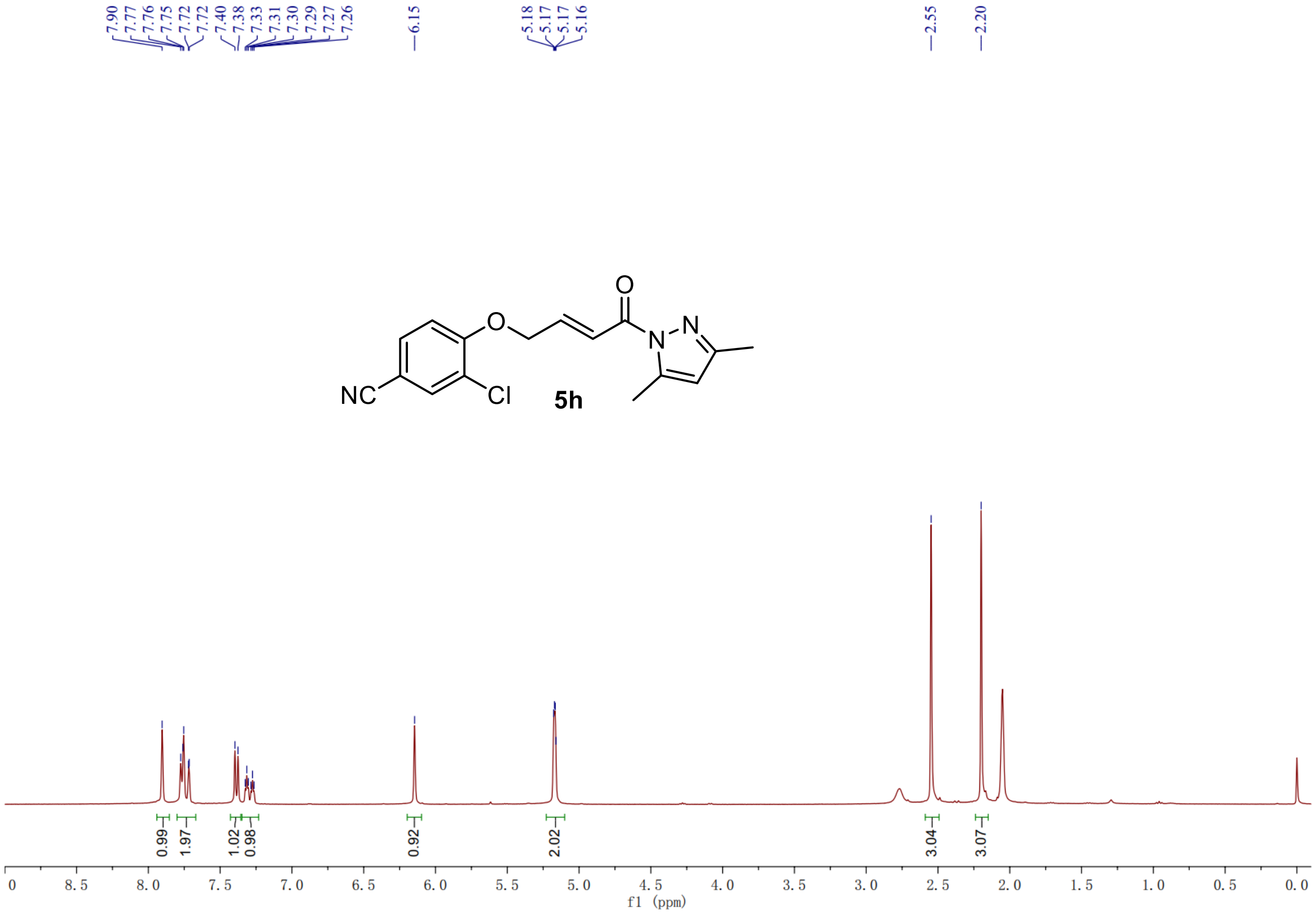
7.90 7.77 7.76 7.75 7.72 7.72 7.40 7.38 7.33 7.31 7.30 7.29 7.27 7.26
6.15
5.18 5.17 5.17 5.16
2.55
2.20
O
N
N
NC
Cl
5h
0.99
1.97
1.02
0.98
0.92
2.02
3.04
3.07
f1 (ppm)

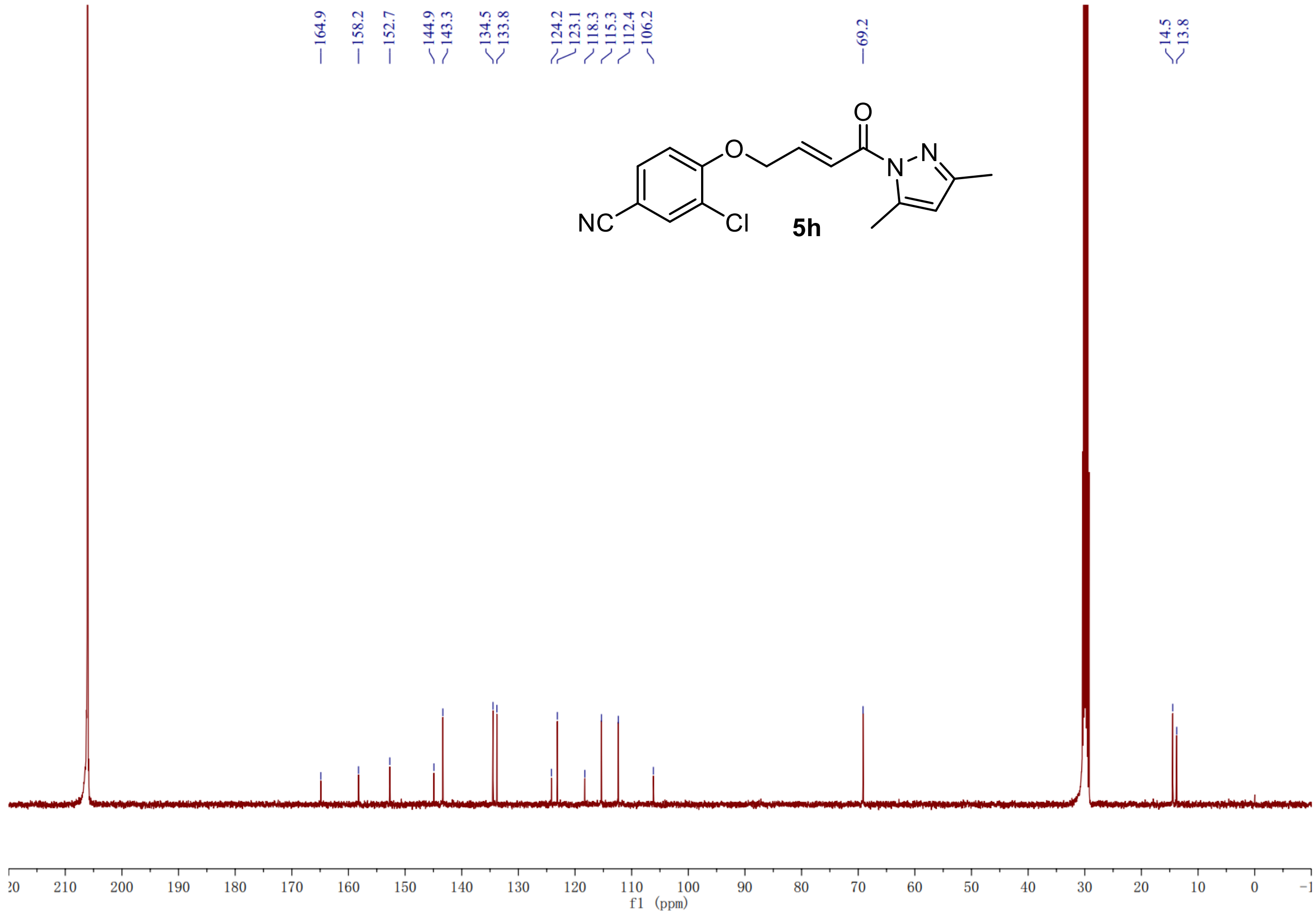
164.9
158.2
152.7
144.9 143.3
134.5 133.8
124.2 123.1 118.3 115.3 112.4 106.2
69.2
14.5 13.8
O
N
N
NC
Cl
5h
f1 (ppm)

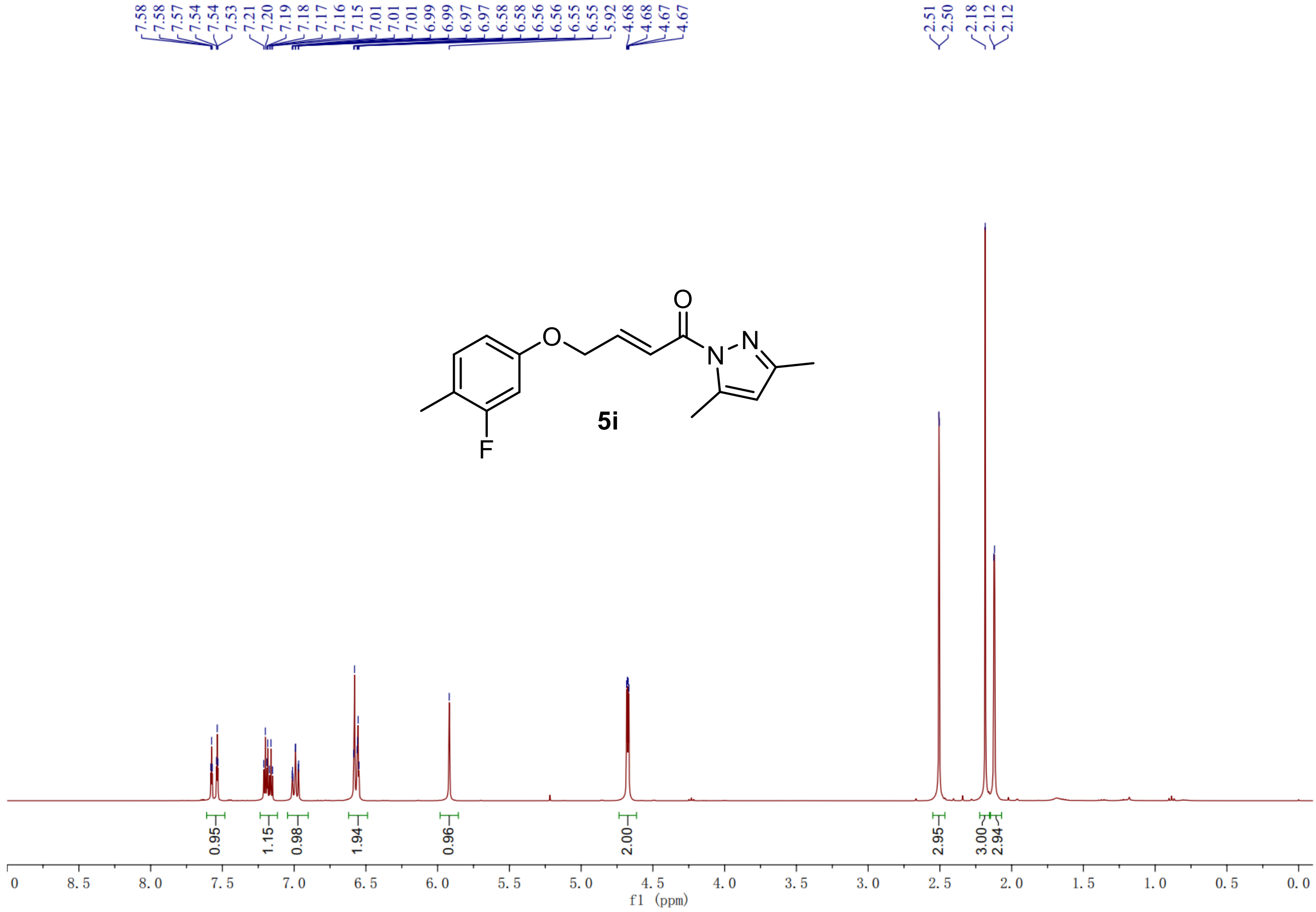
7.58 7.58 7.57 7.54 7.54 7.53 7.21 7.20 7.19 7.18 7.17 7.16 7.15 7.01 7.01 7.01 6.99 6.99 6.97 6.97 6.58 6.58 6.56 6.56 6.55 6.55 5.92 4.68 4.68 4.67 4.67
2.51 2.50 2.18 2.12 2.12
O
N
N
F
0.95 1.15 0.98 1.94 0.96 2.00 2.95 3.00 2.94
f1 (ppm)

5i

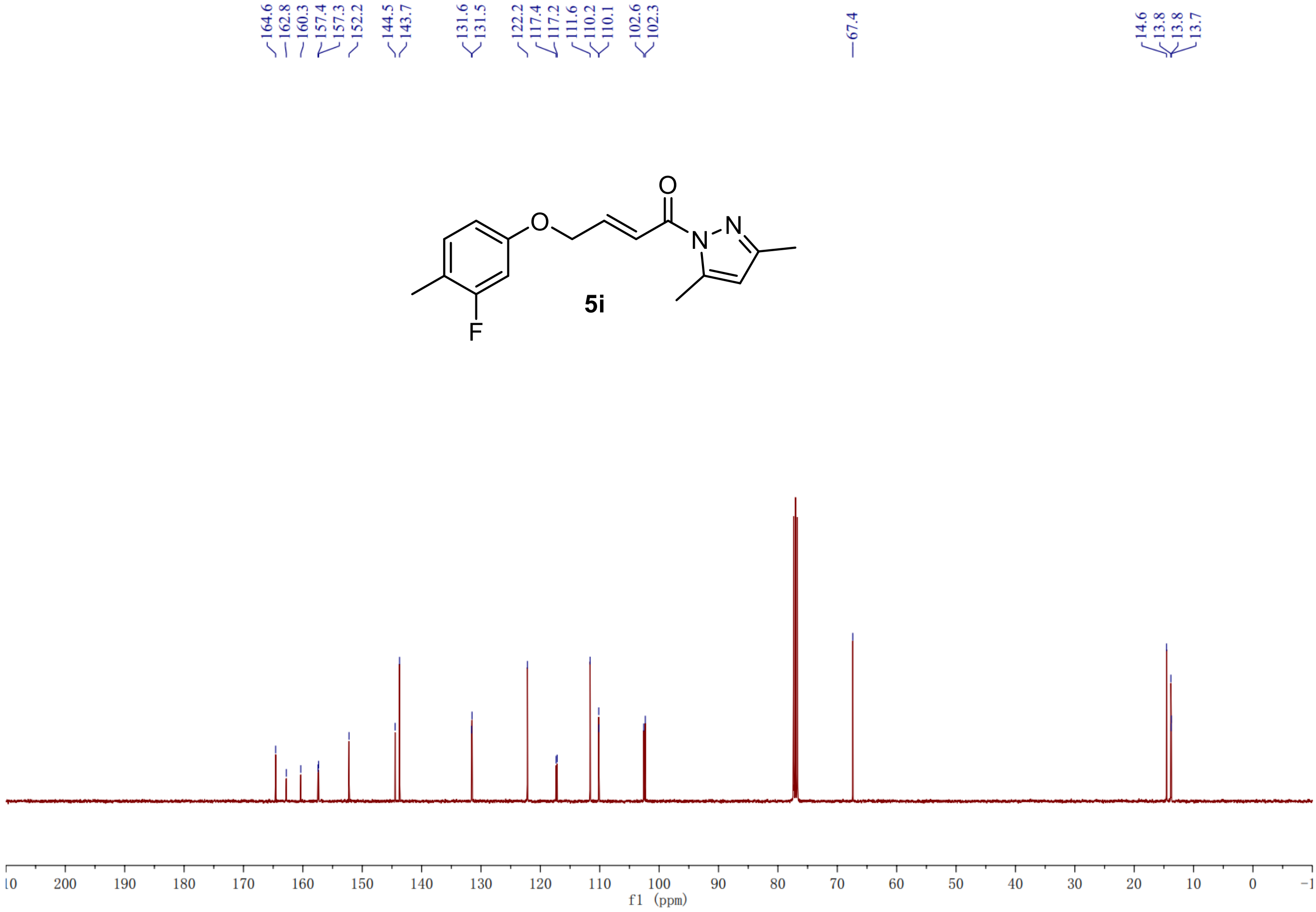
164.6 162.8 160.3 157.4 157.3 152.2 144.5 143.7 131.6 131.5 122.2 117.4 117.2 111.6 110.2 110.1 102.6 102.3
67.4
14.6 13.8 13.8 13.7
O
N
N
F
f1 (ppm)

5i

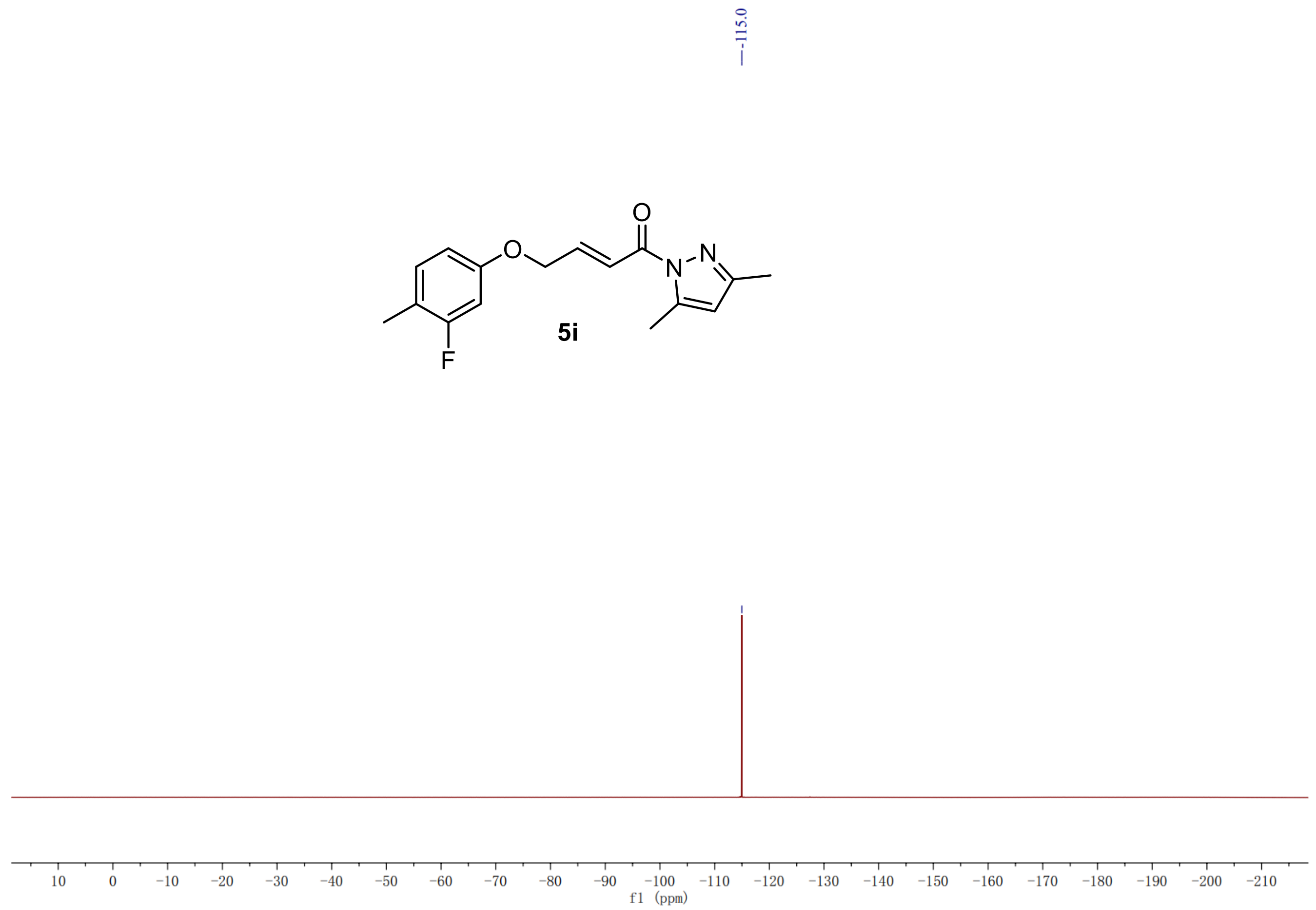
-115.0
O
O
N
N
F
5i
10 0 −10 −20 −30 −40 −50 −60 −70 −80 −90 −100 −110 −120 −130 −140 −150 −160 −170 −180 −190 −200 −210
f1 (ppm)

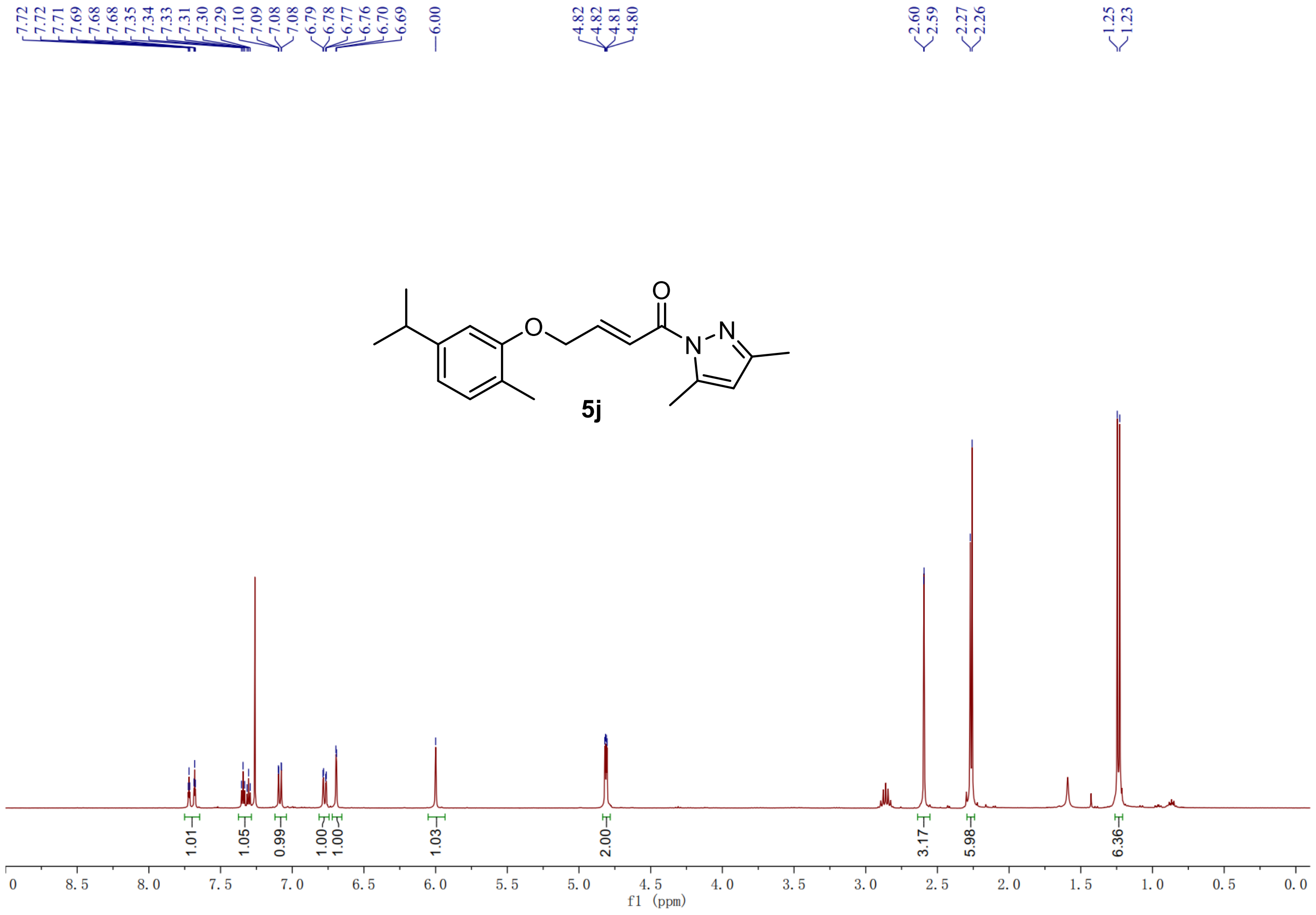
7.72 7.72 7.71 7.69 7.68 7.68 7.35 7.34 7.33 7.31 7.30 7.29 7.10 7.09 7.08 7.08 6.79 6.78 6.77 6.76 6.70 6.69
6.00
4.82 4.82 4.81 4.80
2.60 2.59
2.27 2.26
1.25 1.23
O
O
N
N
5j
1.01 1.05 0.99 1.00 1.00 1.03 2.00 3.17 5.98 6.36
0 8.5 8.0 7.5 7.0 6.5 6.0 5.5 5.0 4.5 4.0 3.5 3.0 2.5 2.0 1.5 1.0 0.5 0.0
f1 (ppm)

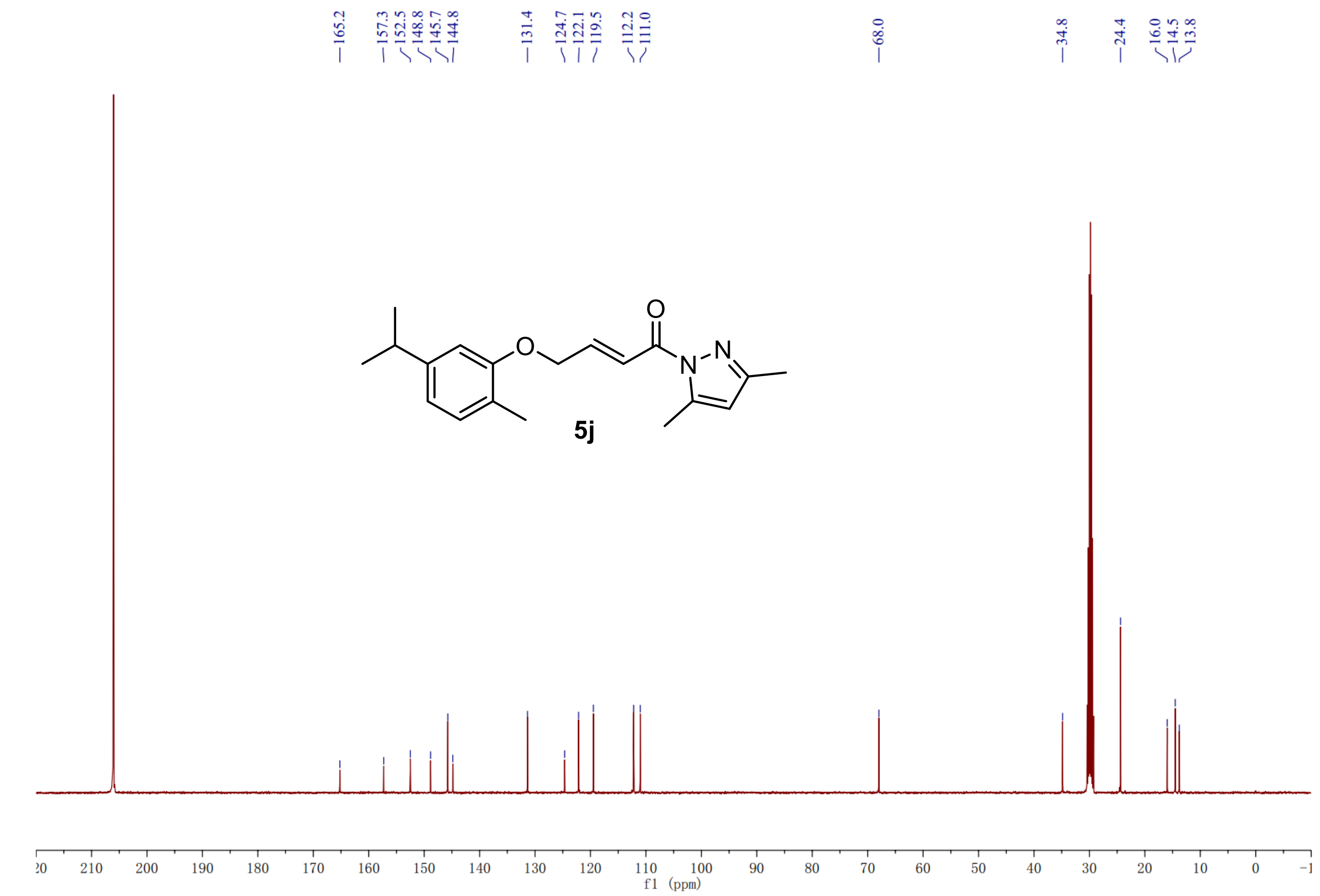
165.2
157.3
152.5
148.8
145.7
144.8
131.4
124.7
122.1
119.5
112.2
111.0
68.0
34.8
24.4
16.0
14.5
13.8
5j
f1 (ppm)

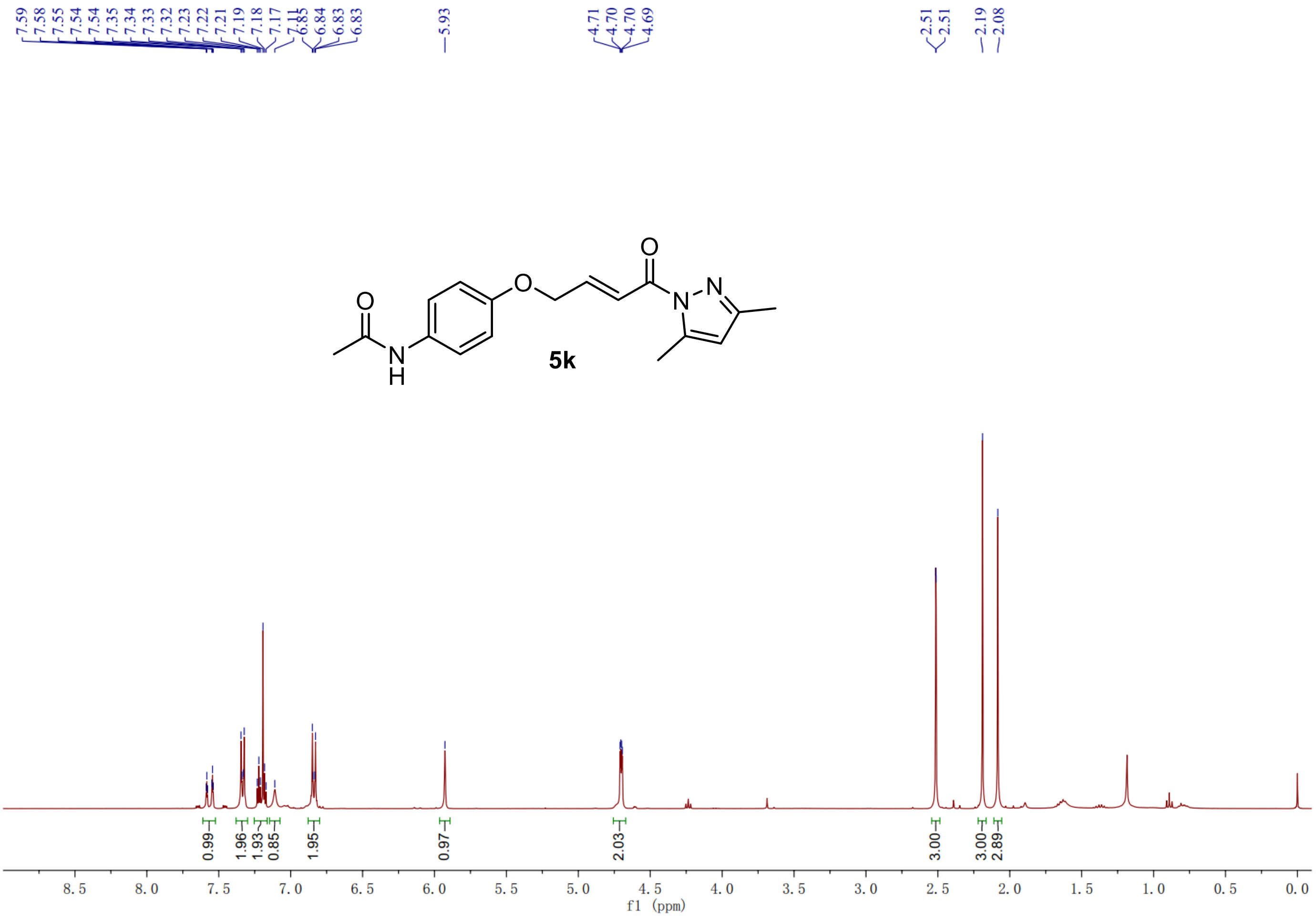
7.59
7.58
7.55
7.54
7.54
7.35
7.34
7.33
7.32
7.23
7.22
7.21
7.19
7.18
7.17
7.11
6.85
6.84
6.83
6.83
5.93
4.71
4.70
4.70
4.69
2.51
2.51
2.19
2.08
5k
0.99
1.96
1.93
0.85
1.95
0.97
2.03
3.00
3.00
2.89
f1 (ppm)

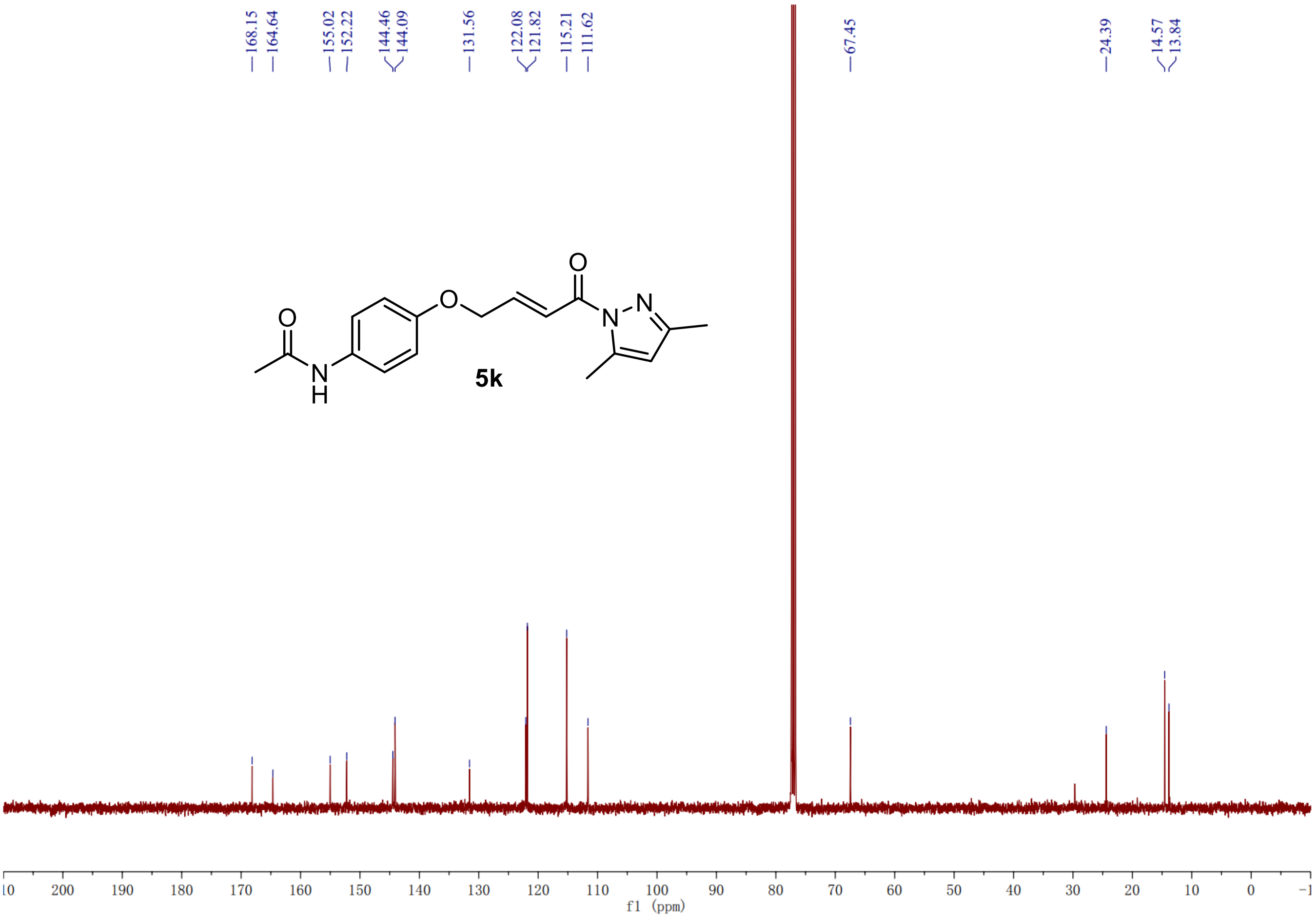

168.15
164.64
155.02
152.22
144.46
144.09
131.56
122.08
121.82
115.21
111.62
67.45
24.39
14.57
13.84
5k
f1 (ppm)

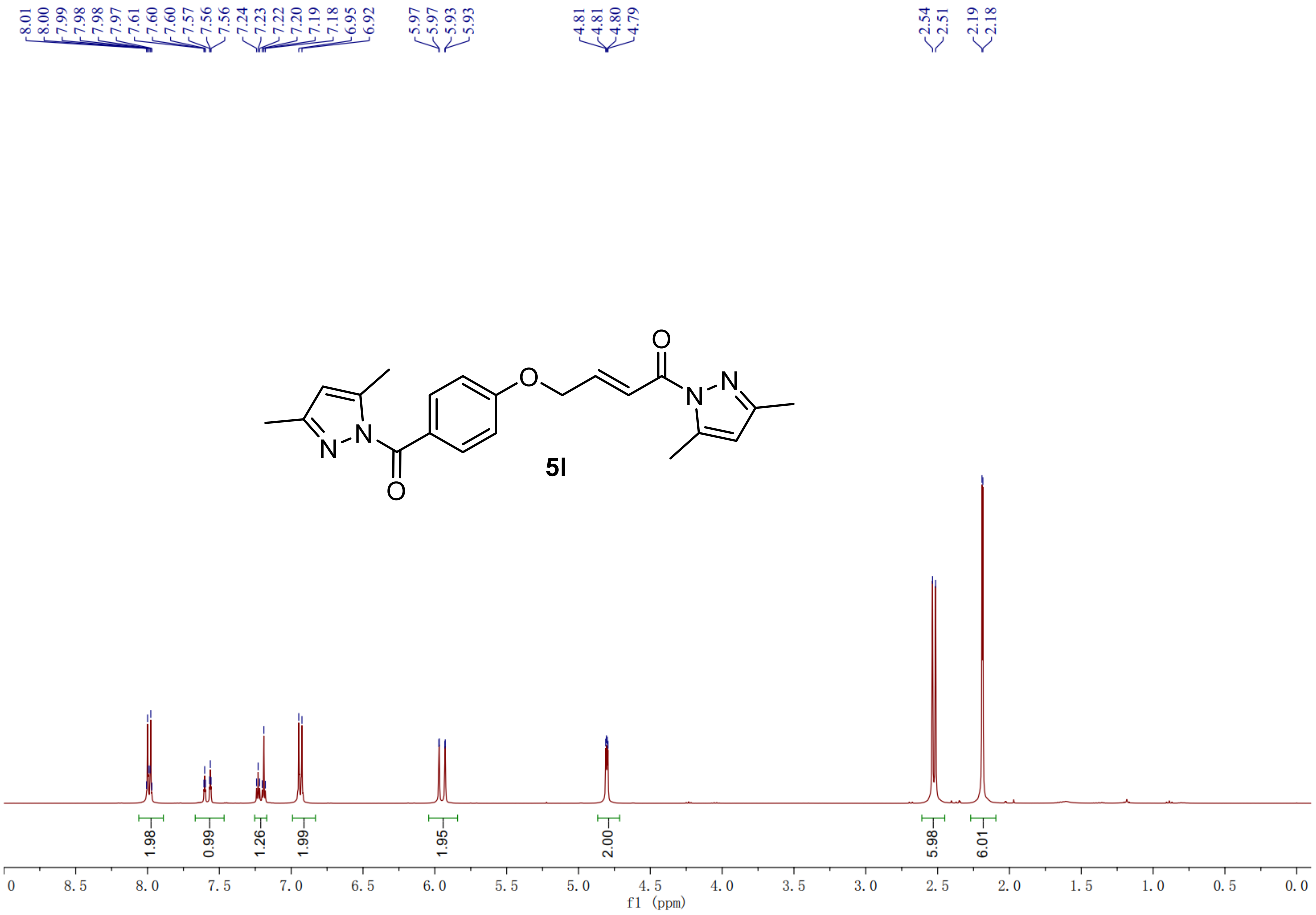

8.01
8.00
7.99
7.98
7.98
7.97
7.61
7.60
7.60
7.57
7.56
7.56
7.24
7.23
7.22
7.20
7.19
7.18
6.95
6.92
5.97
5.97
5.93
5.93
4.81
4.81
4.80
4.79
2.54
2.51
2.19
2.18
5l
1.98
0.99
1.26
1.99
1.95
2.00
5.98
6.01
f1 (ppm)

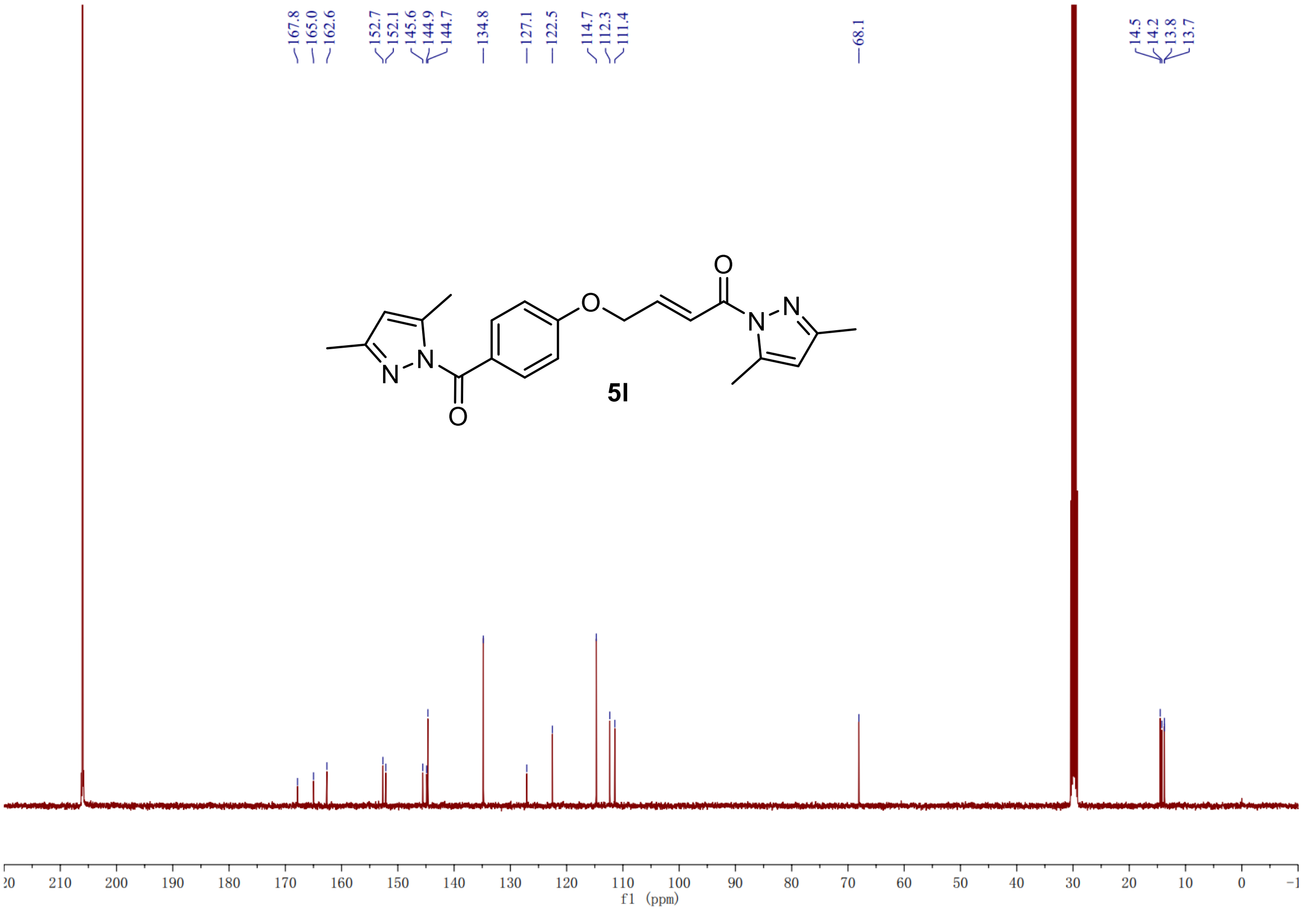
167.8
165.0
162.6
152.7
152.1
145.6
144.9
144.7
134.8
127.1
122.5
114.7
112.3
111.4
68.1
14.5
14.2
13.8
13.7
5l
f1 (ppm)

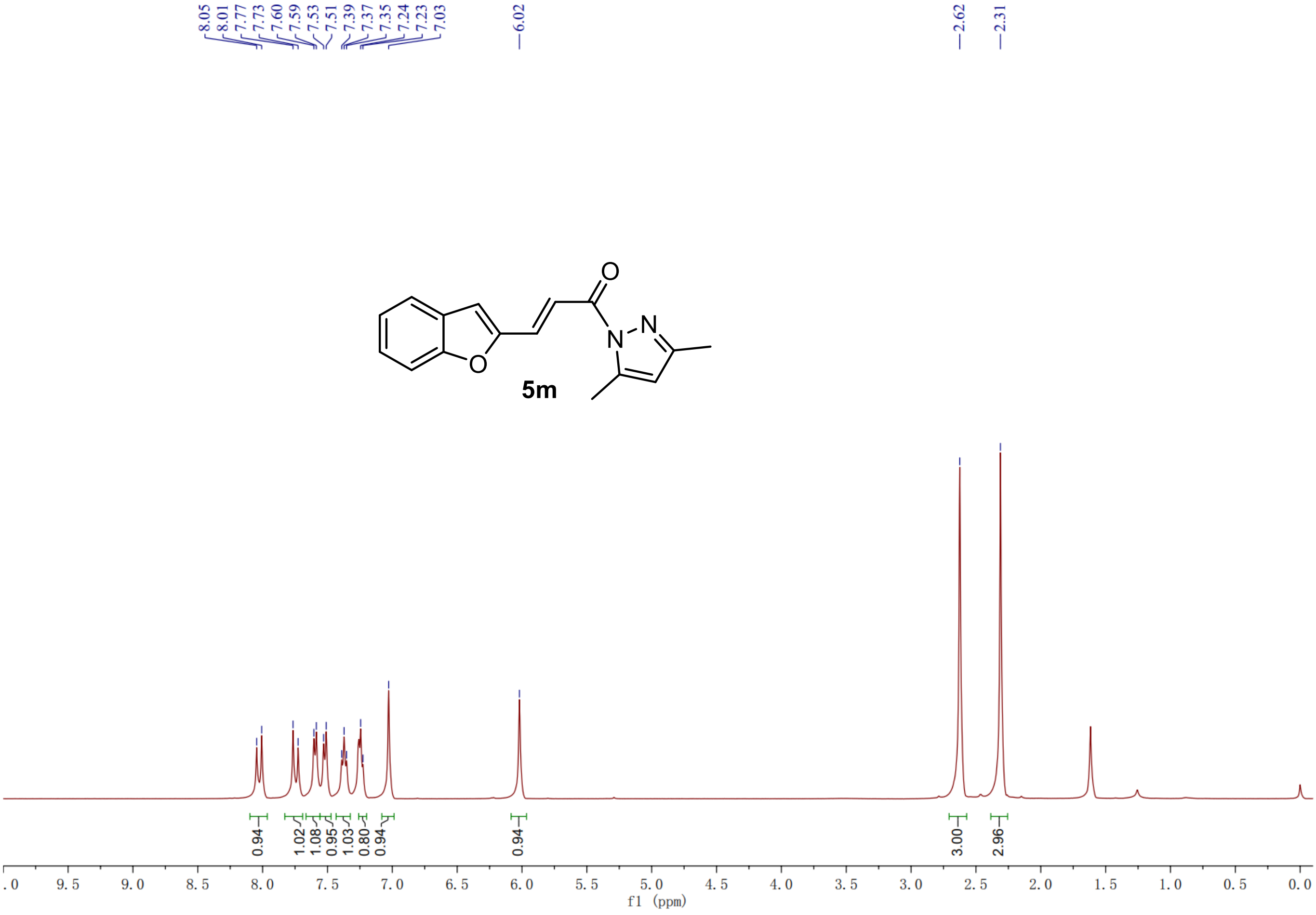
8.05
8.01
7.77
7.73
7.60
7.59
7.53
7.51
7.39
7.37
7.35
7.24
7.23
7.03
6.02
2.62
2.31
5m
0.94
1.02
1.08
0.95
1.03
0.80
0.94
0.94
3.00
2.96
f1 (ppm)

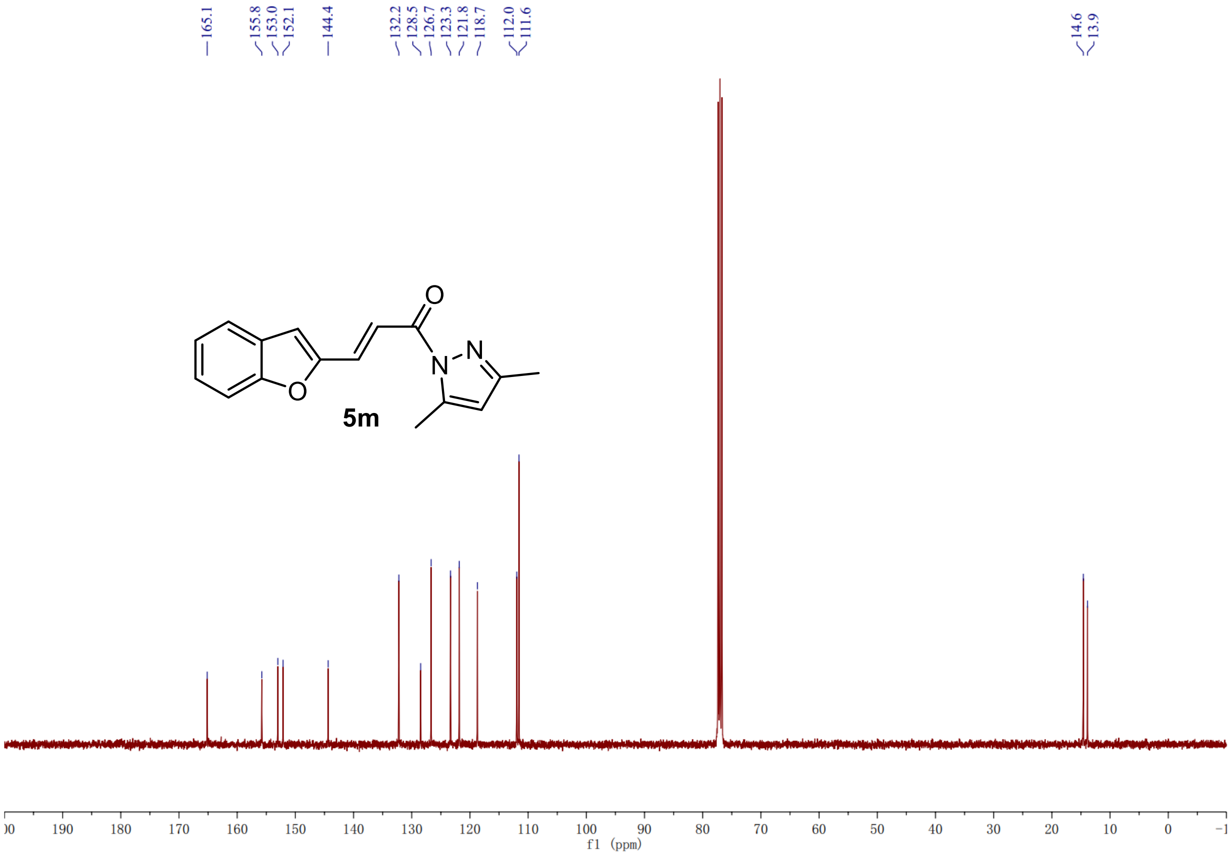
165.1
155.8
153.0
152.1
144.4
132.2
128.5
126.7
123.3
121.8
118.7
112.0
111.6
14.6
13.9
5m
f1 (ppm)

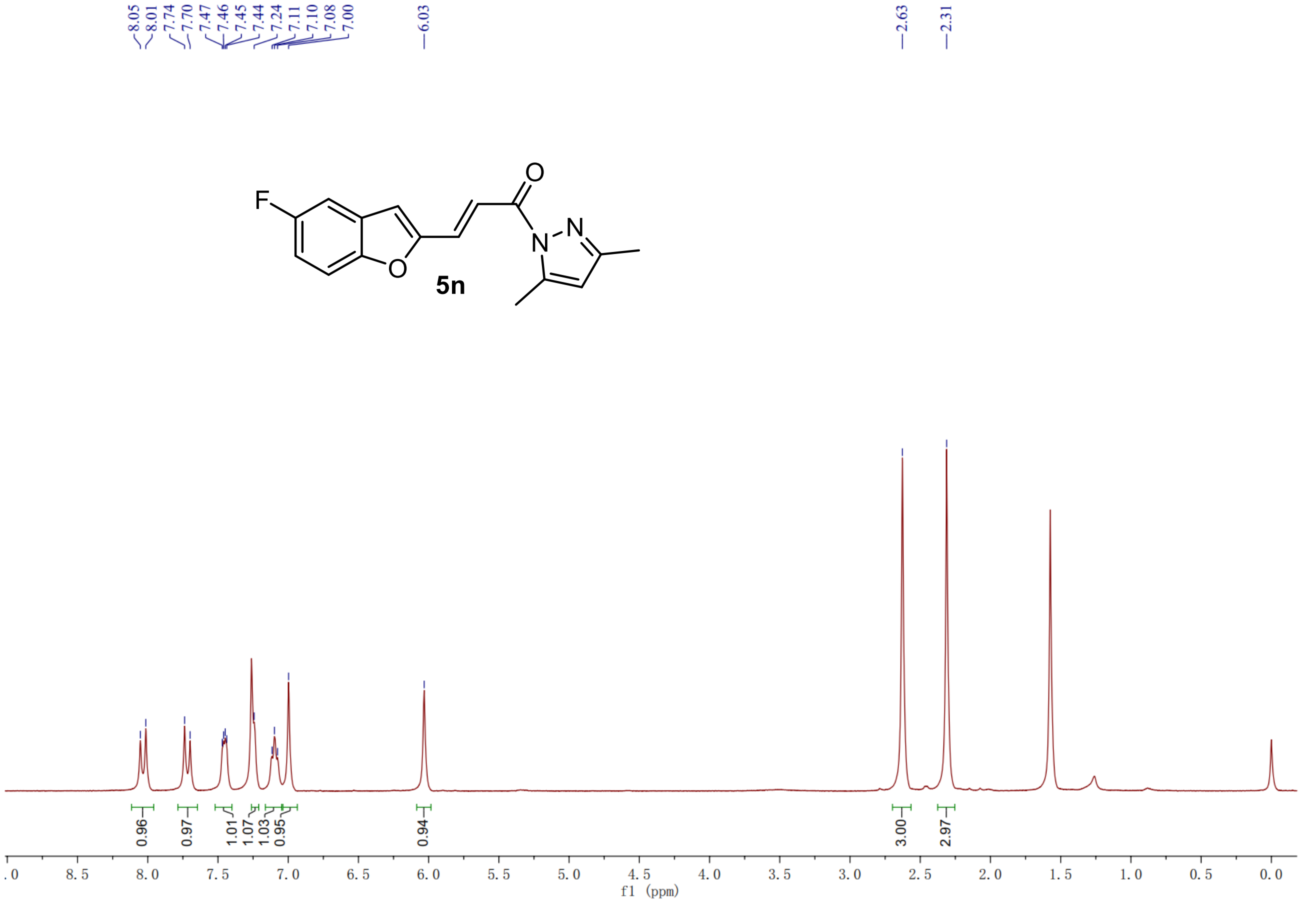
8.05
8.01
7.74
7.70
7.47
7.46
7.45
7.44
7.24
7.11
7.10
7.08
7.00
6.03
2.63
2.31
5n
0.96
0.97
1.01
1.07
1.03
0.95
0.94
3.00
2.97
f1 (ppm)

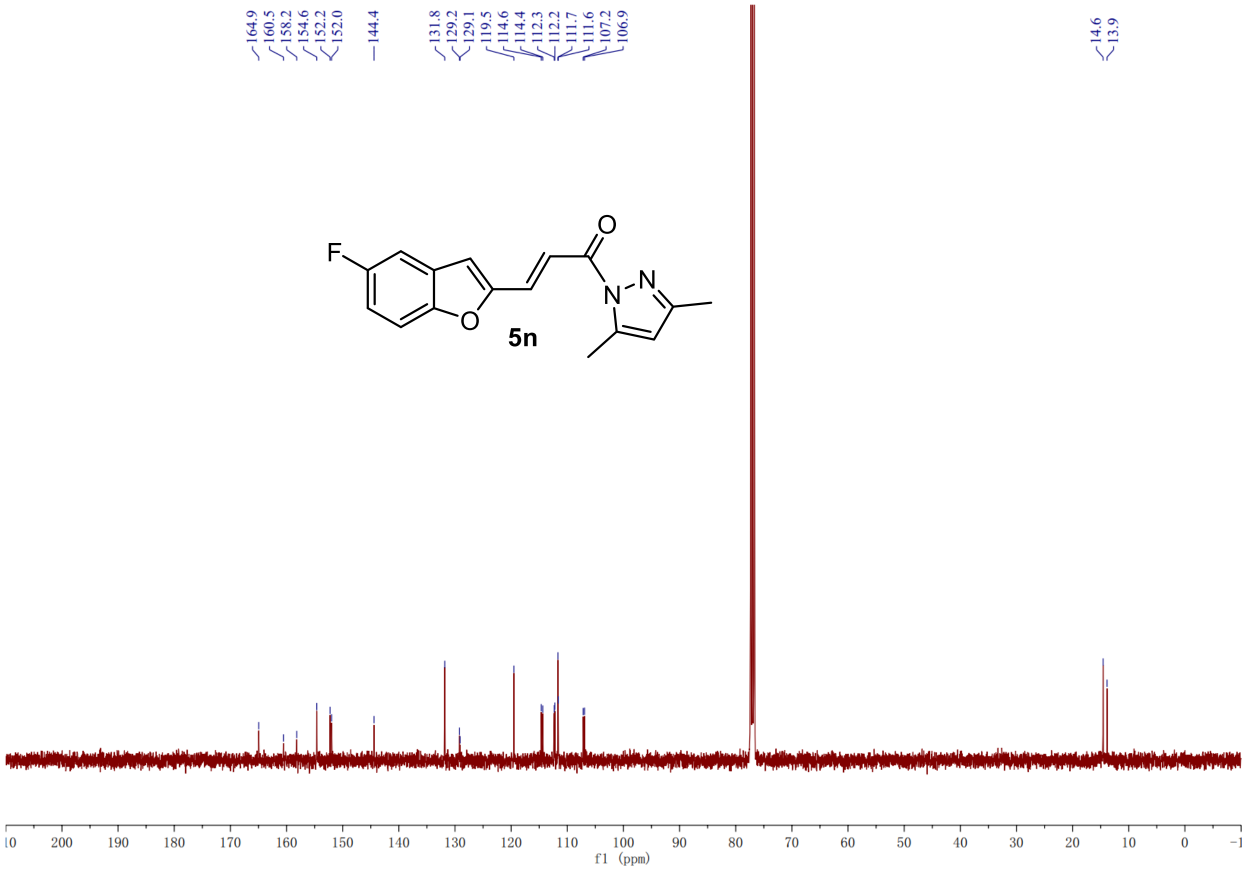
164.9
160.5
158.2
154.6
152.2
152.0
144.4
131.8
129.2
129.1
119.5
114.6
114.4
112.3
112.2
111.7
111.6
107.2
106.9
14.6
13.9
F
O
N
N
O
5n
f1 (ppm)

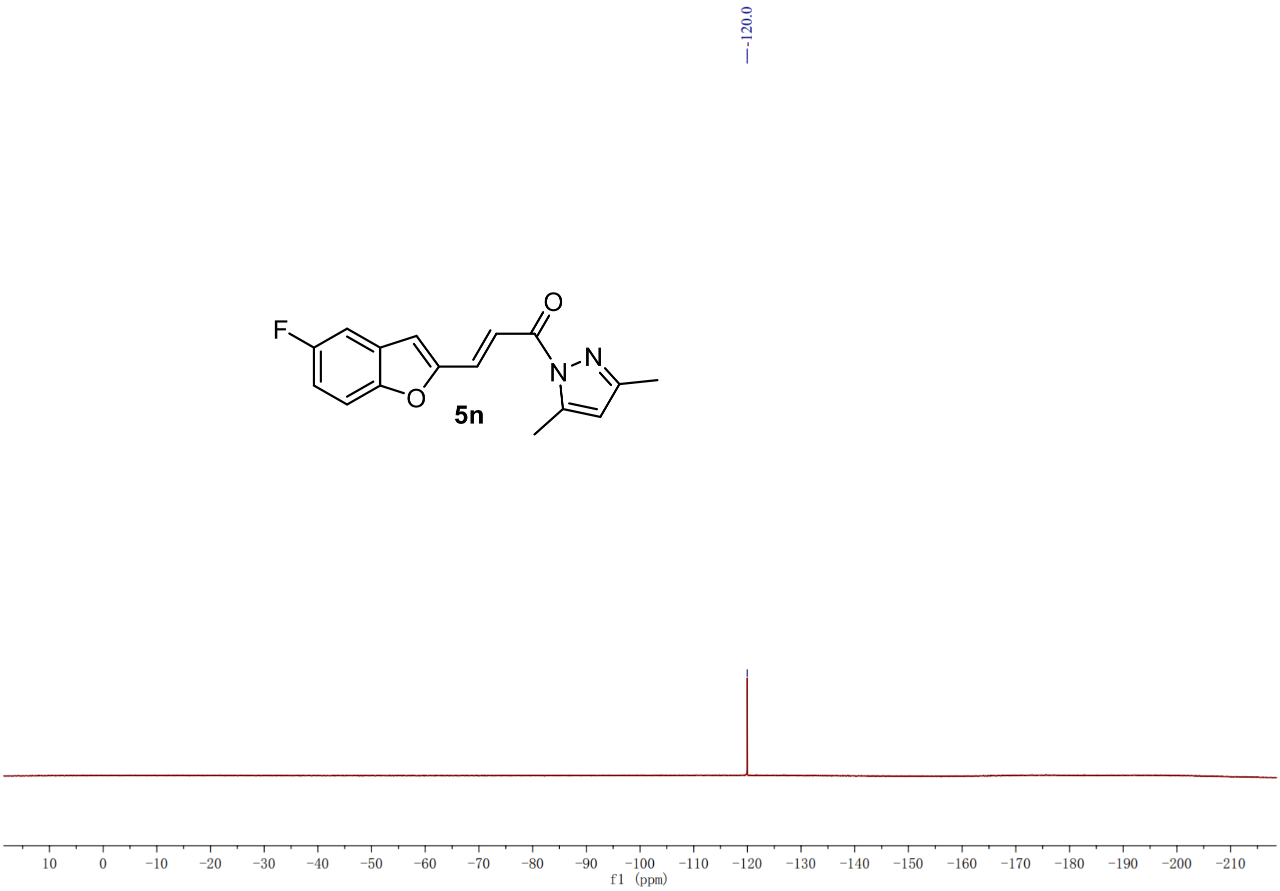
-120.0
F
O
N
N
O
5n
f1 (ppm)

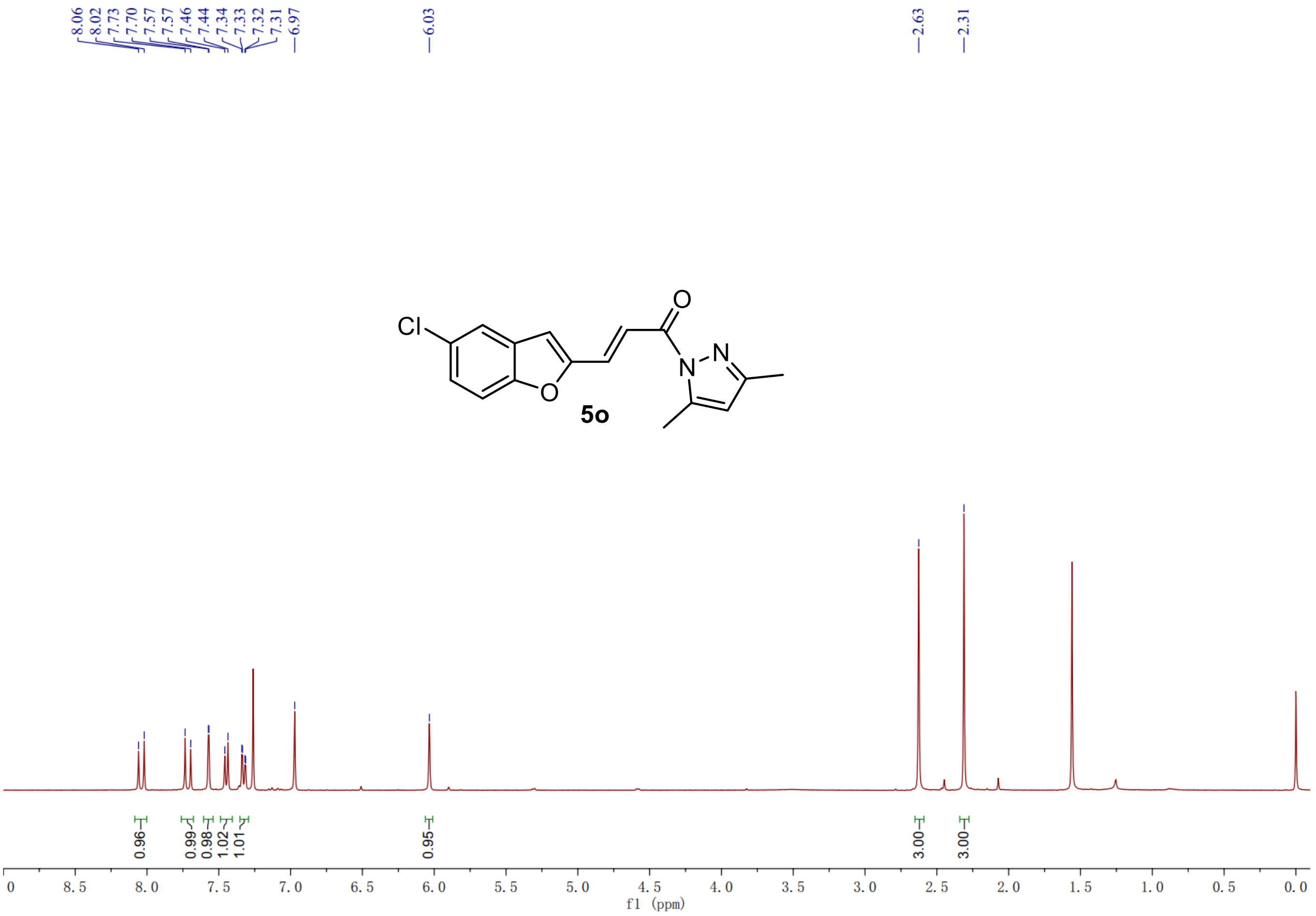
8.06 8.02 7.73 7.70 7.57 7.57 7.46 7.44 7.34 7.33 7.32 7.31 6.97
6.03
2.63
2.31
Cl
O
N
N
5o
0.96 0.99 0.98 1.02 1.01
0.95
3.00 3.00
f1 (ppm)

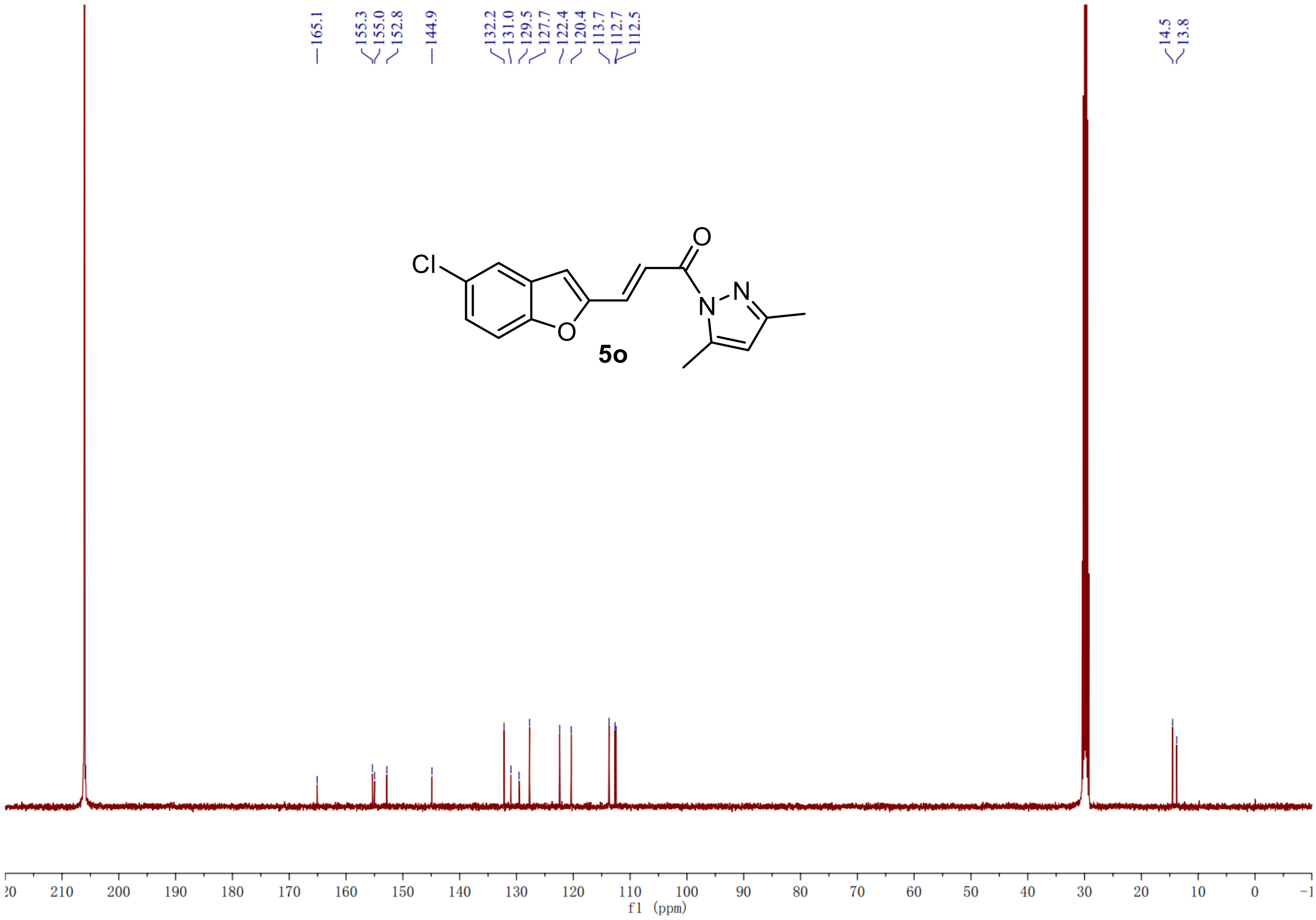
165.1
155.3 155.0 152.8
144.9
132.2 131.0 129.5 127.7 122.4 120.4 113.7 112.7 112.5
14.5 13.8
Cl
O
N
N
5o
f1 (ppm)

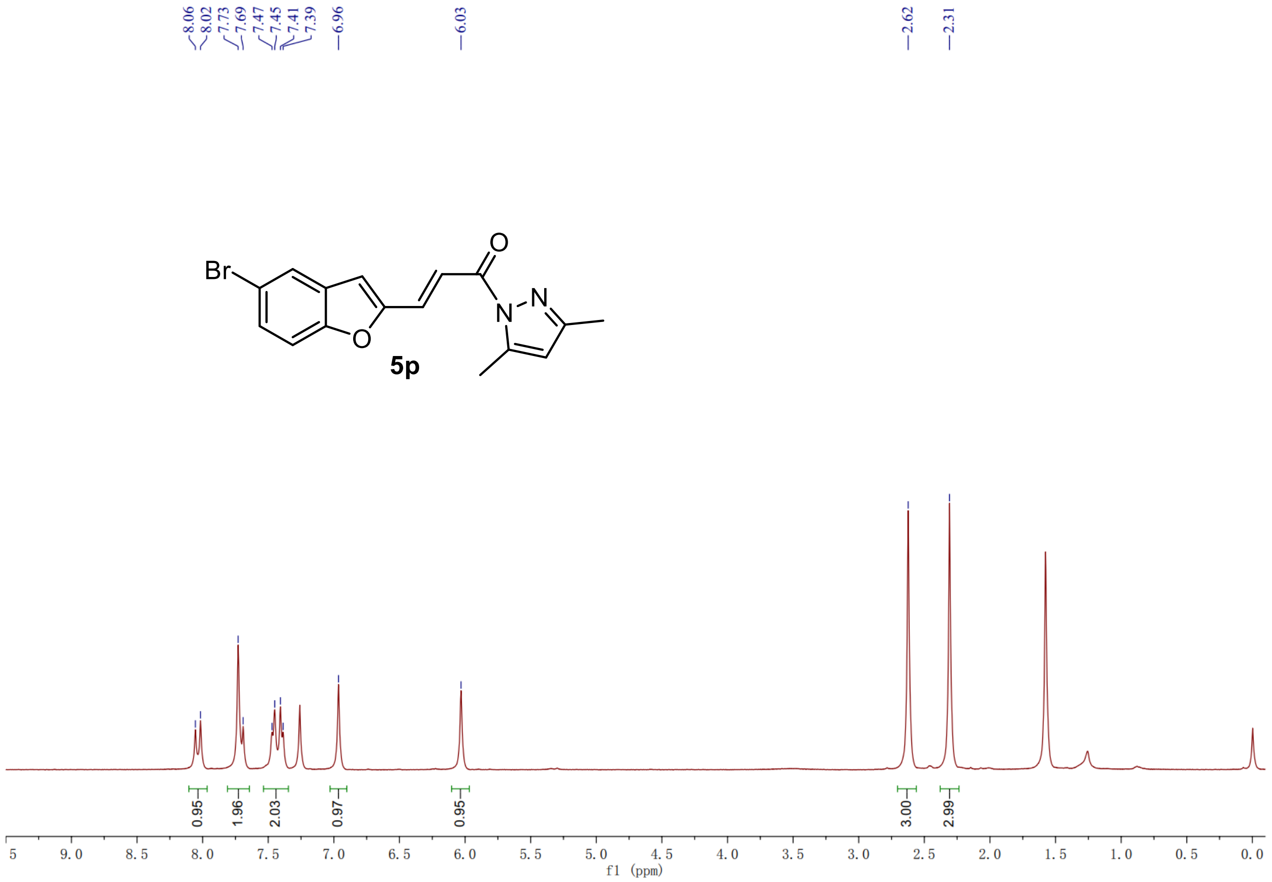
8.06
8.02
7.73
7.69
7.47
7.45
7.41
7.39
6.96
6.03
2.62
2.31
Br
O
N
N
O
5p
0.95
1.96
2.03
0.97
0.95
3.00
2.99
9.0
8.5
8.0
7.5
7.0
6.5
6.0
5.5
5.0
4.5
4.0
3.5
3.0
2.5
2.0
1.5
1.0
0.5
0.0
f1 (ppm)

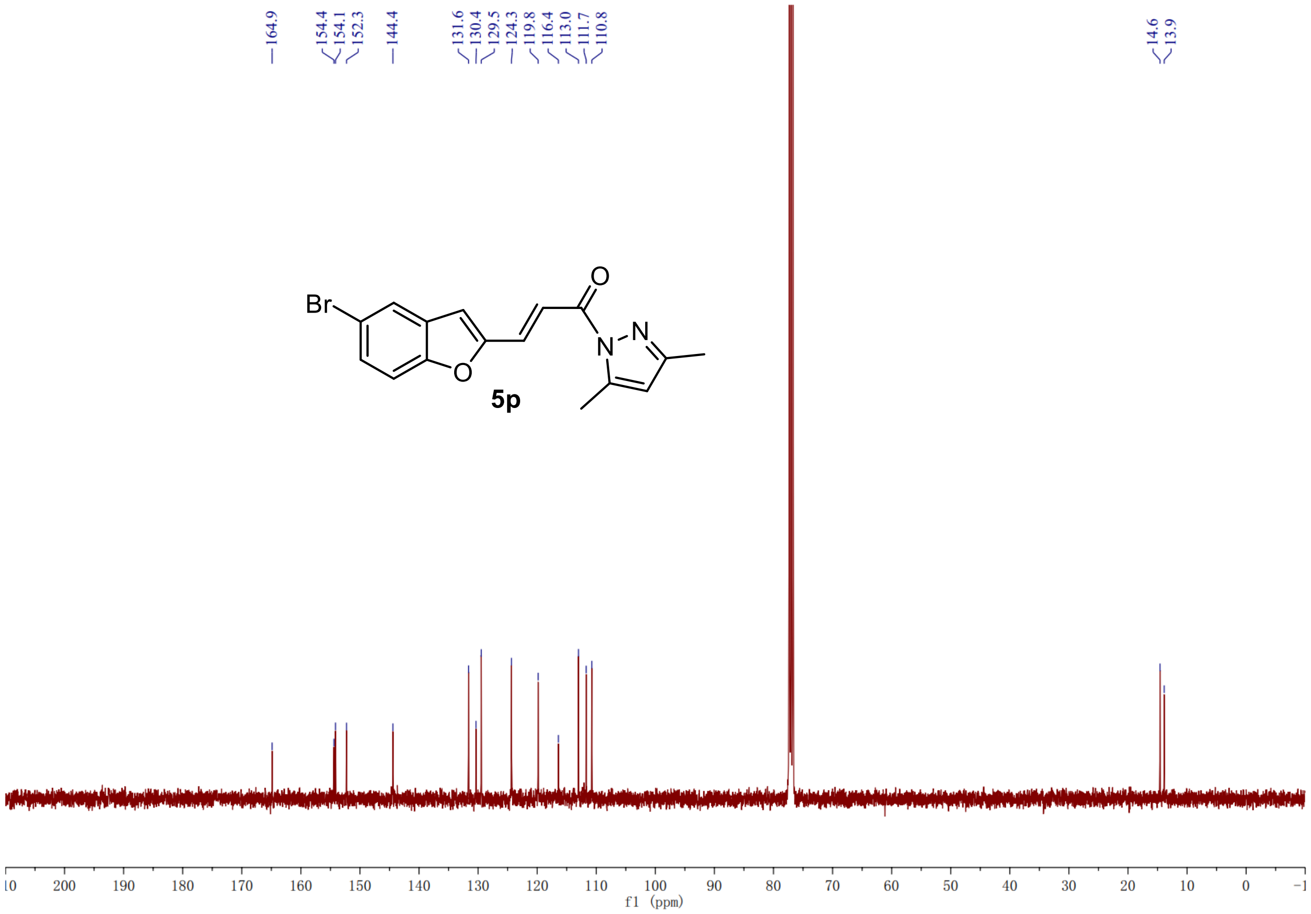
164.9
154.4
154.1
152.3
144.4
131.6
130.4
129.5
124.3
119.8
116.4
113.0
111.7
110.8
14.6
13.9
Br
O
N
N
O
5p
200
190
180
170
160
150
140
130
120
110
100
90
80
70
60
50
40
30
20
10
0
f1 (ppm)

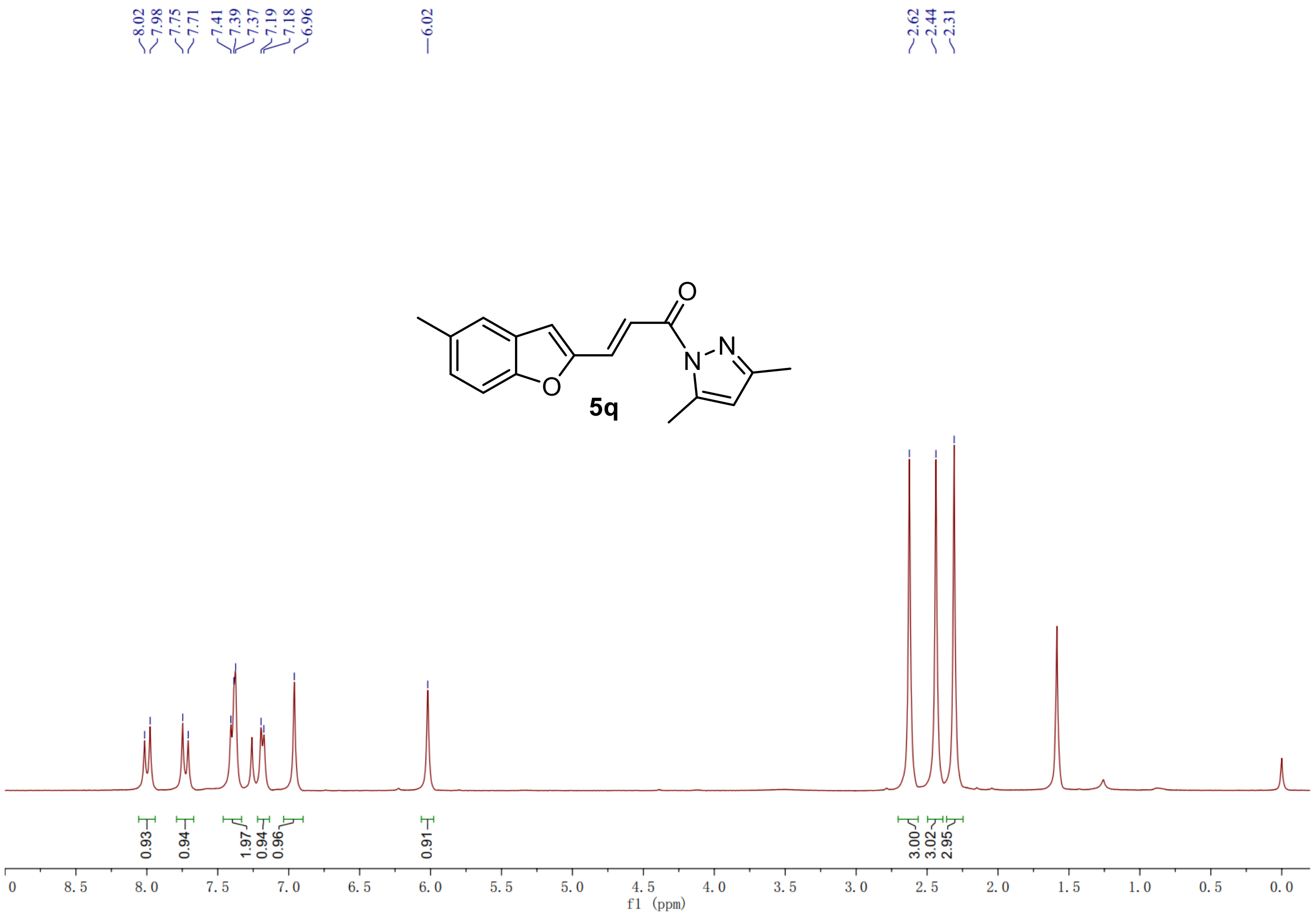
8.02
7.98
7.75
7.71
7.41
7.39
7.37
7.19
7.18
6.96
6.02
2.62
2.44
2.31
O
N
N
5q
0.93
0.94
1.97
0.94
0.96
0.91
3.00
3.02
2.95
f1 (ppm)

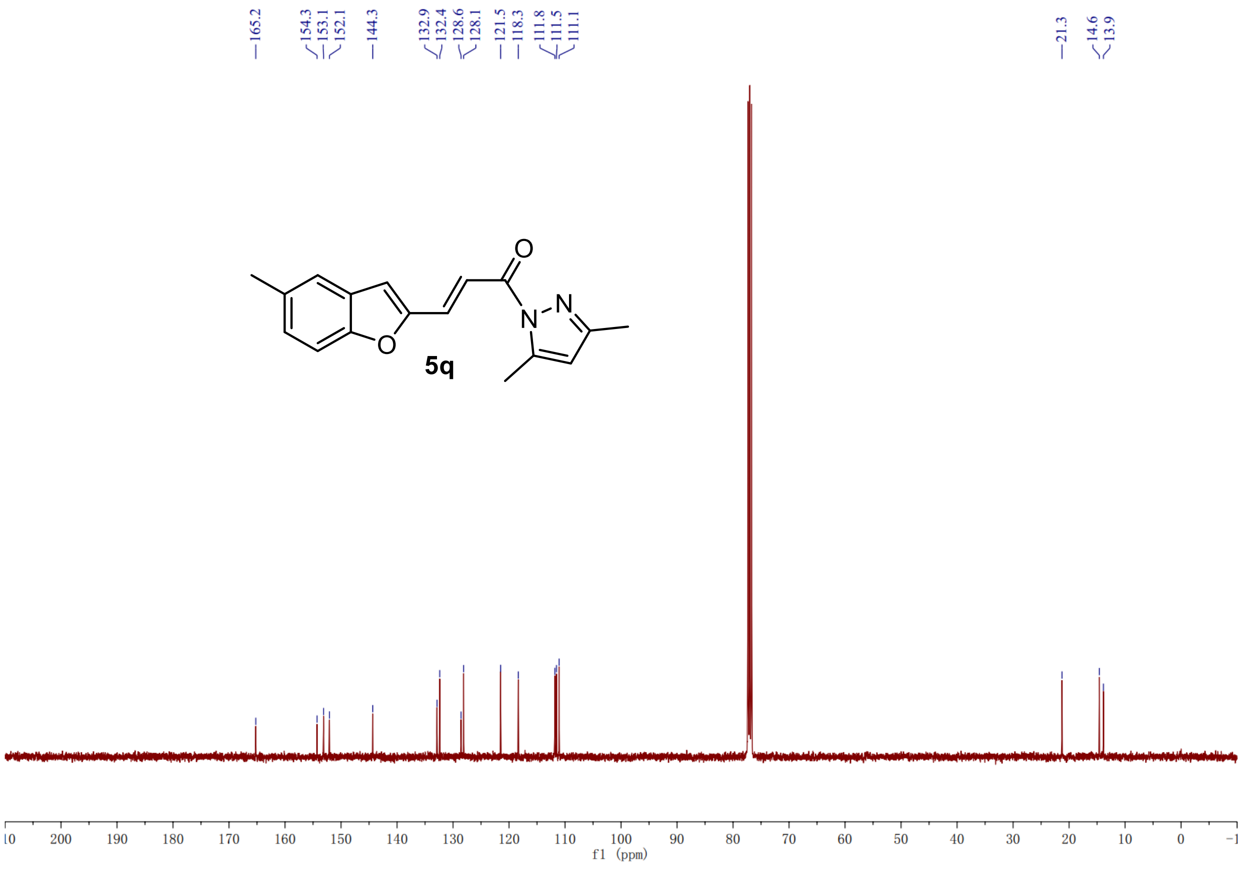
165.2
154.3
153.1
152.1
144.3
132.9
132.4
128.6
128.1
121.5
118.3
111.8
111.5
111.1
21.3
14.6
13.9
O
N
N
5q
f1 (ppm)

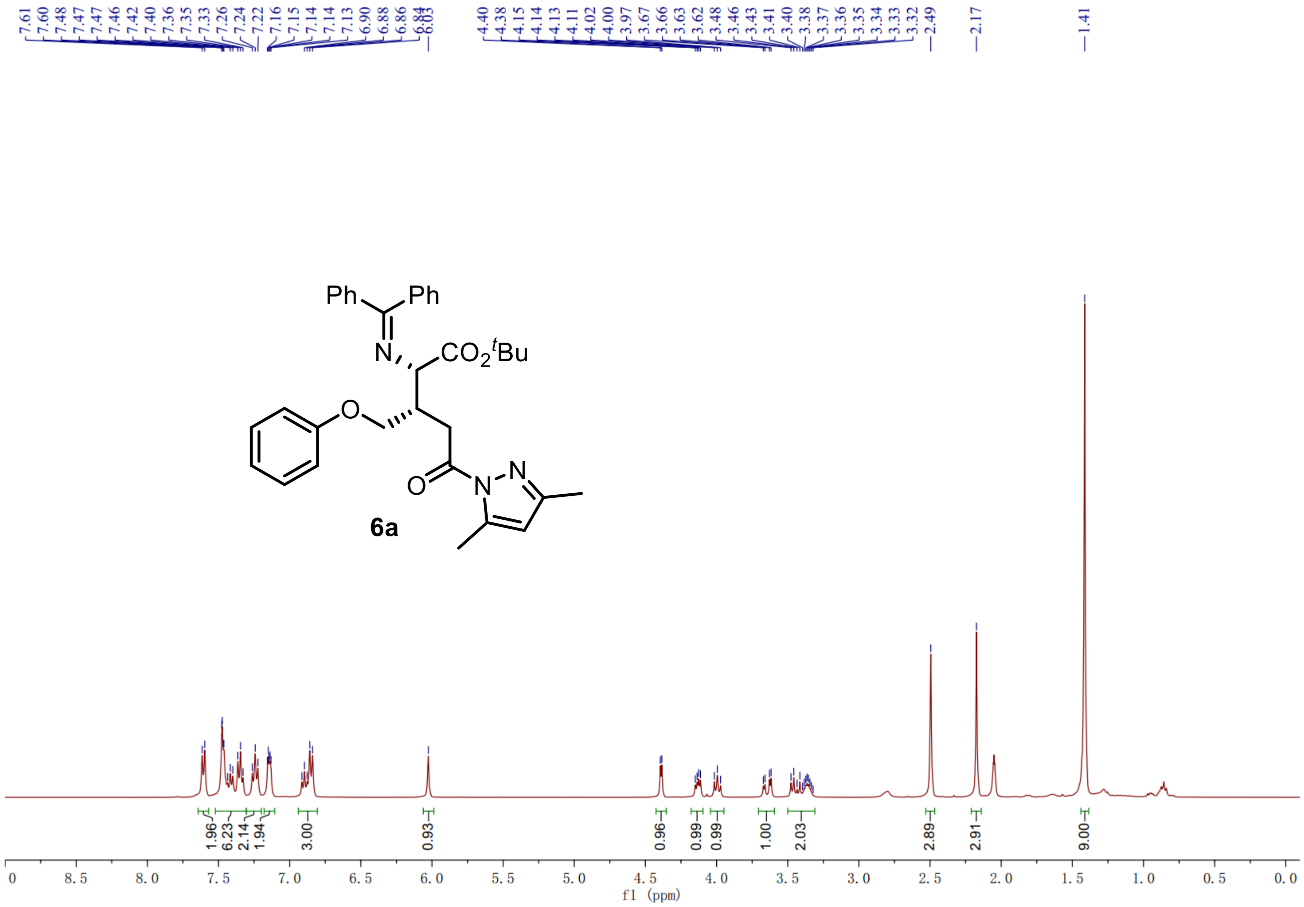
7.61 7.60 7.48 7.47 7.47 7.46 7.42 7.40 7.36 7.35 7.33 7.26 7.24 7.22 7.16 7.15 7.14 7.14 7.13 6.90 6.88 6.86 6.84 6.03
4.40 4.38 4.15 4.14 4.13 4.11 4.02 4.00 3.97 3.67 3.66 3.63 3.62 3.48 3.46 3.43 3.41 3.40 3.38 3.37 3.36 3.35 3.34 3.33 3.32 2.49
2.17
1.41
Ph
Ph
N
CO2tBu
O
O
N
N
6a
1.96 6.23 2.14 1.94 3.00 0.93 0.96 0.99 0.99 1.00 2.03 2.89 2.91 9.00
f1 (ppm)

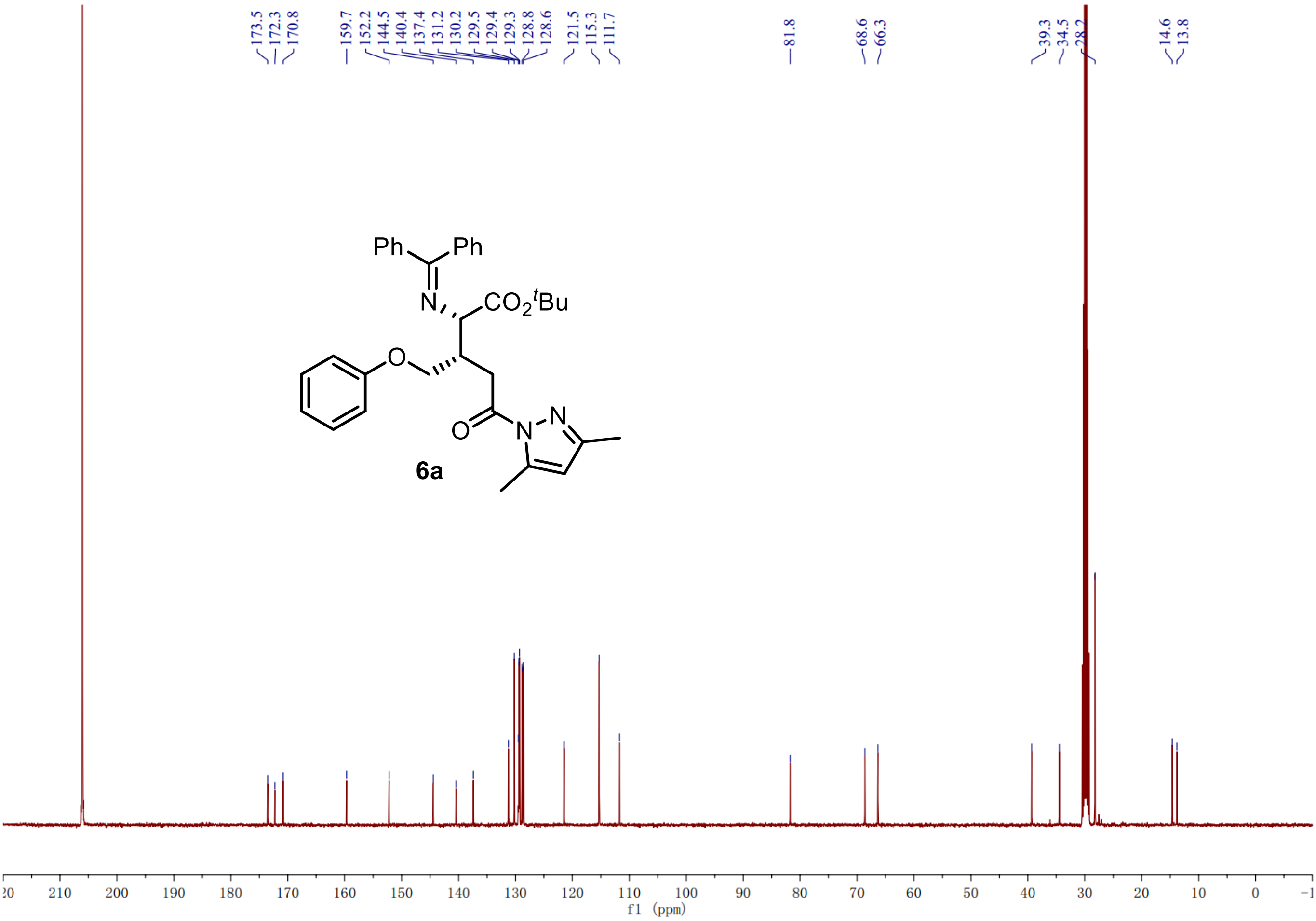
173.5 172.3 170.8 159.7 152.2 144.5 140.4 137.4 131.2 130.2 129.5 129.4 129.3 128.8 128.6 121.5 115.3 111.7
81.8
68.6 66.3
39.3 34.5 28.2
14.6 13.8
Ph
Ph
N
CO2tBu
O
O
N
N
6a
f1 (ppm)

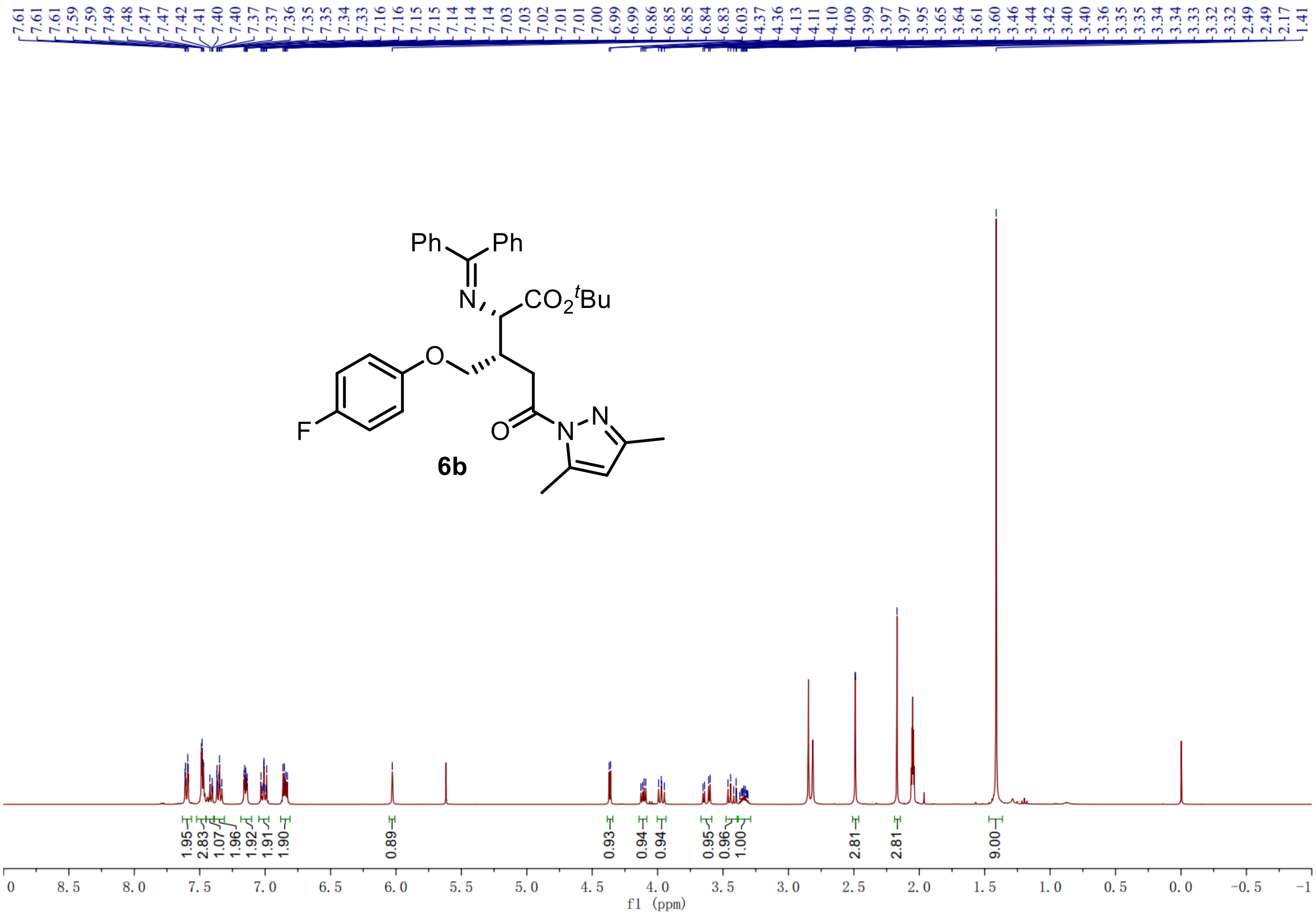

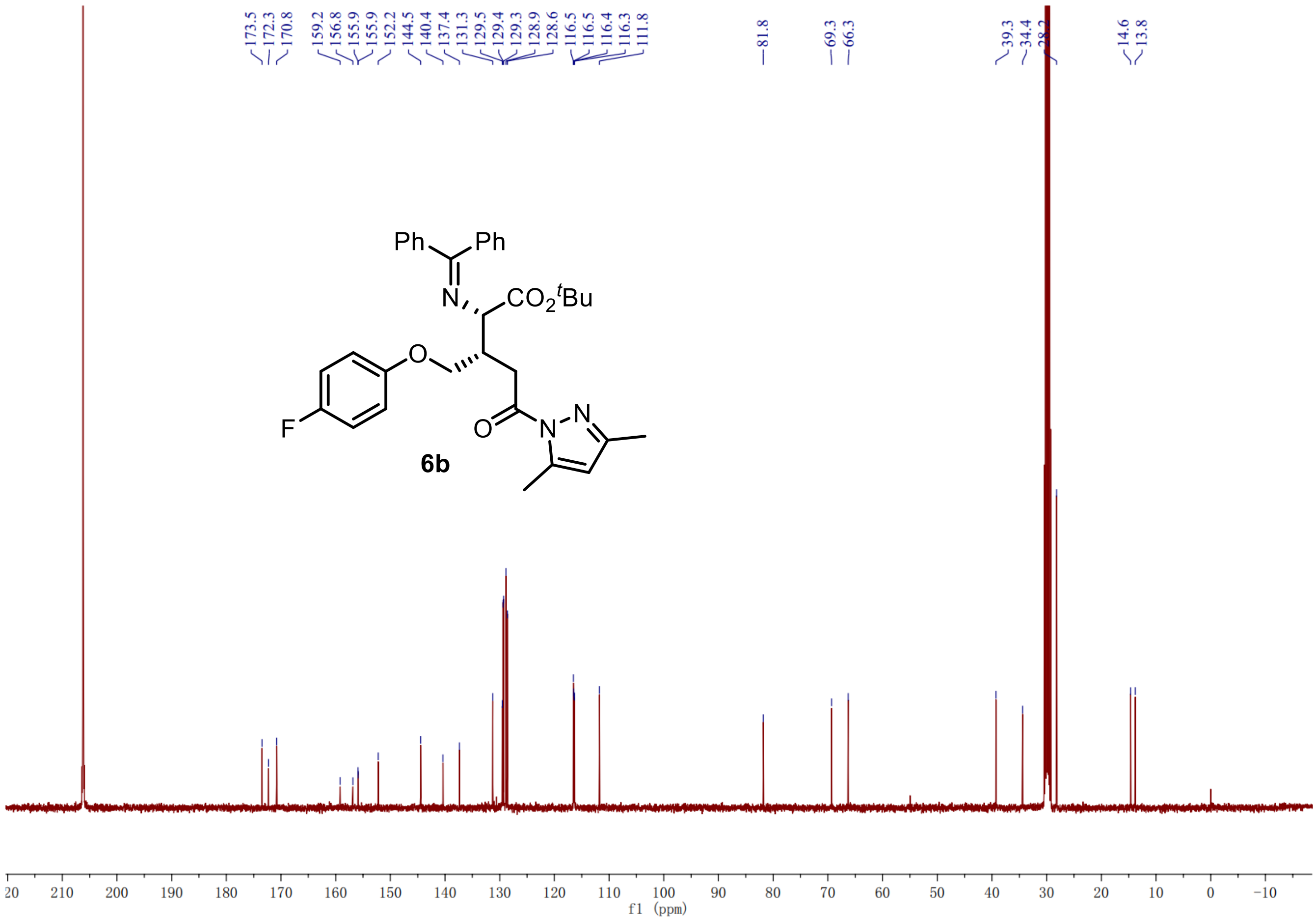

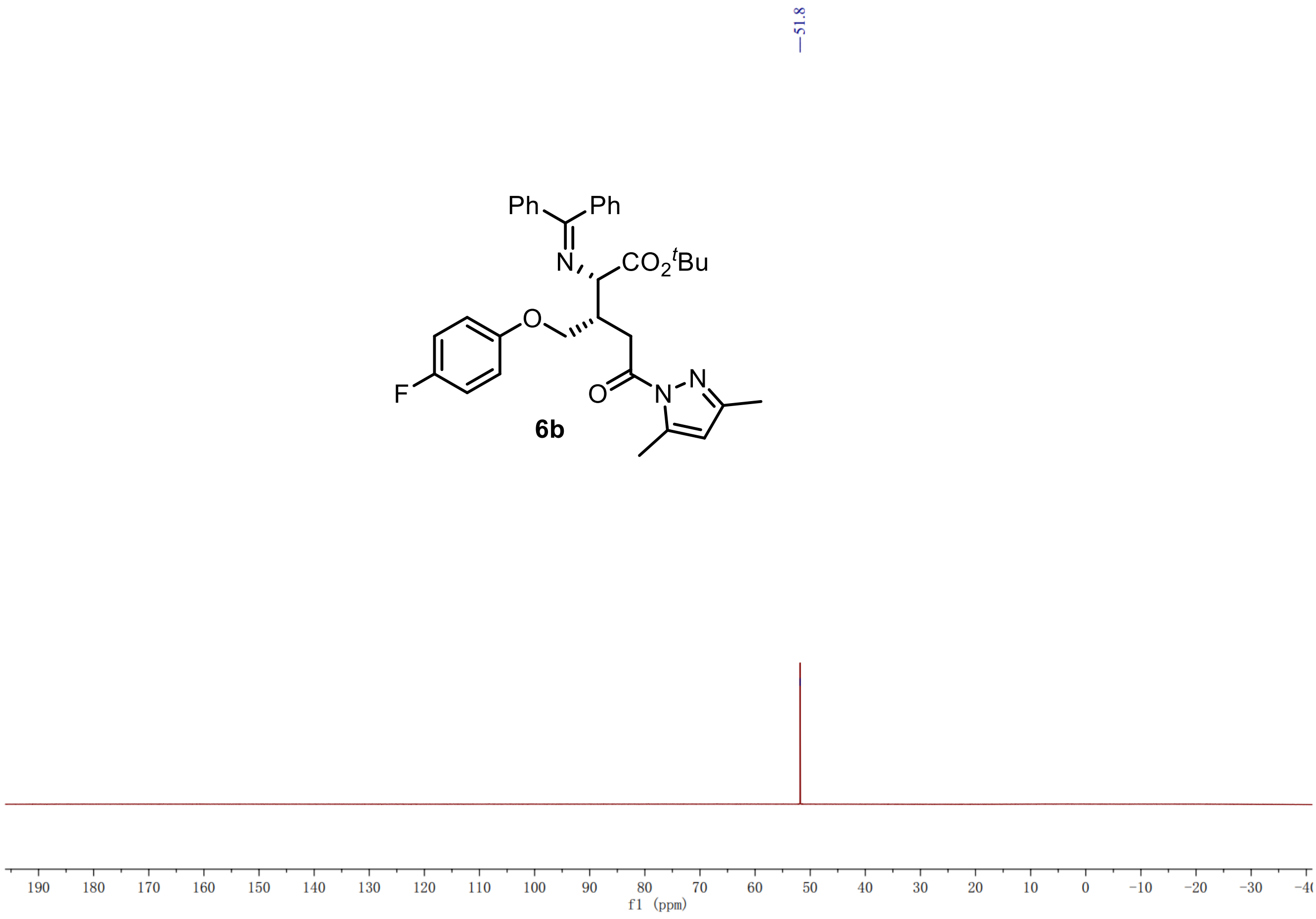
—51.8
Ph
Ph
N
CO2tBu
O
F
O
N
N
6b
f1 (ppm)

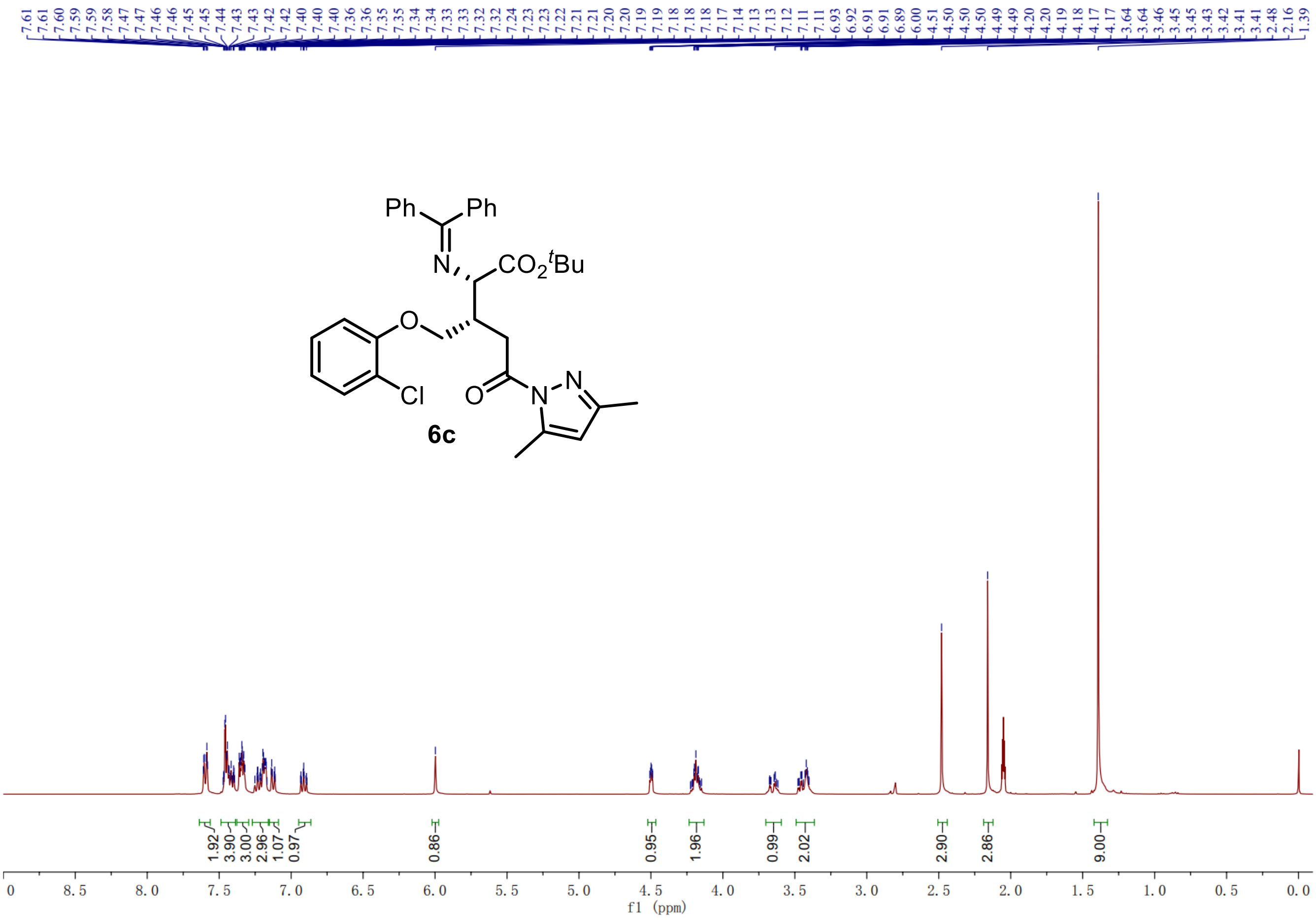
Ph
Ph
N
CO2tBu
O
Cl
O
N
N
6c
1.92
3.00
3.90
2.96
1.07
0.97
0.86
0.95
1.96
0.99
2.02
2.90
2.86
9.00
f1 (ppm)

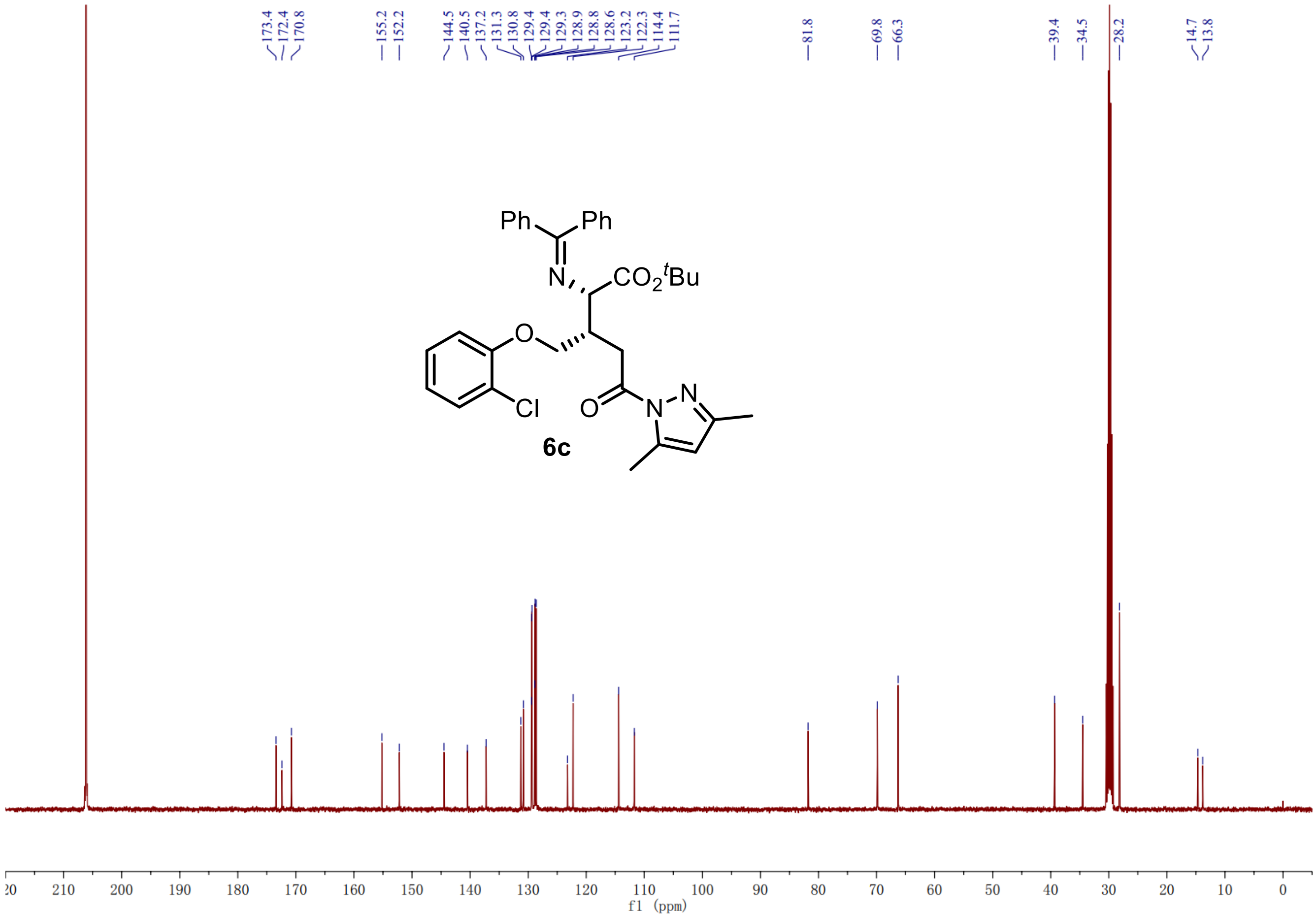
6c
f1 (ppm)

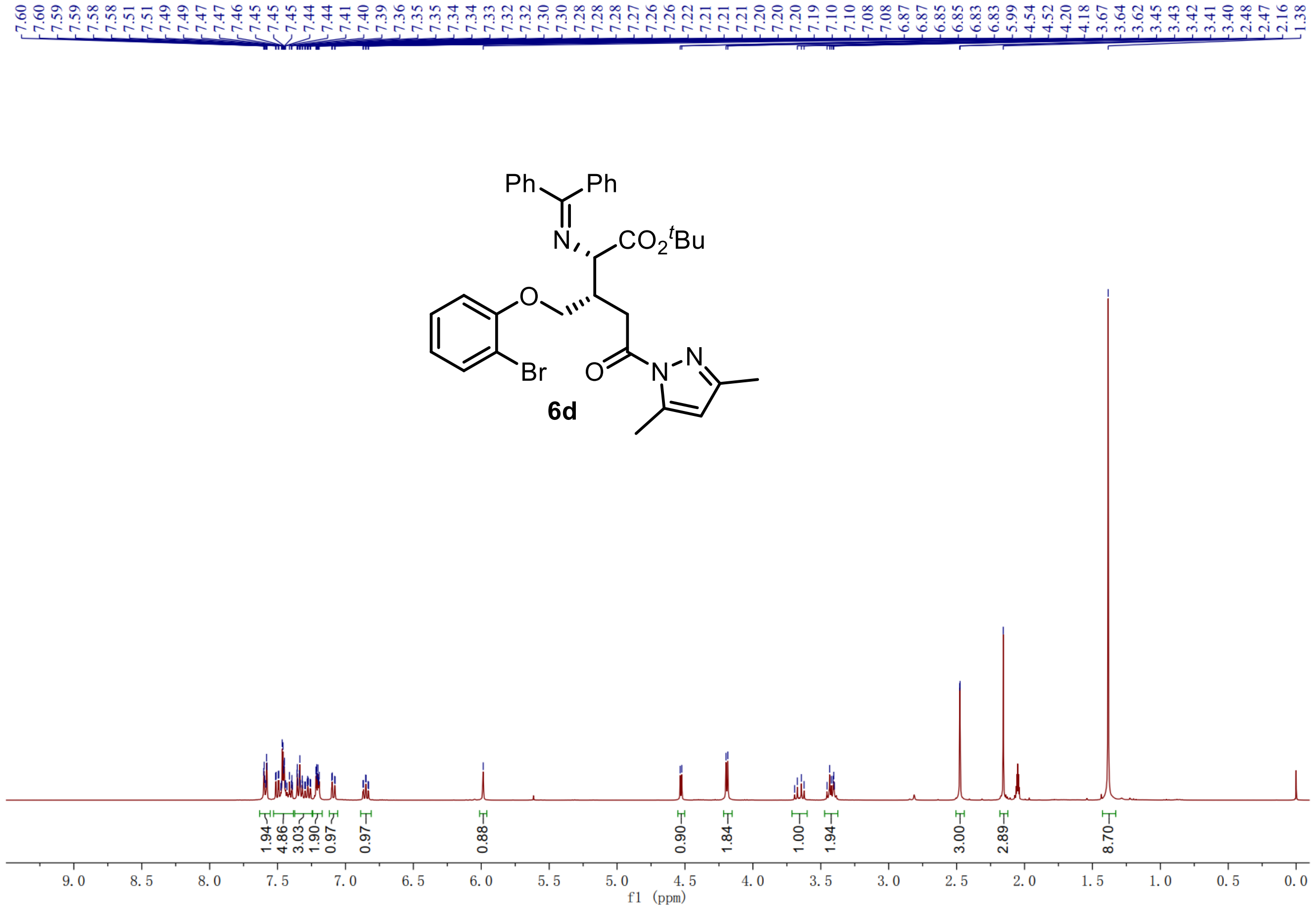
6d
f1 (ppm)

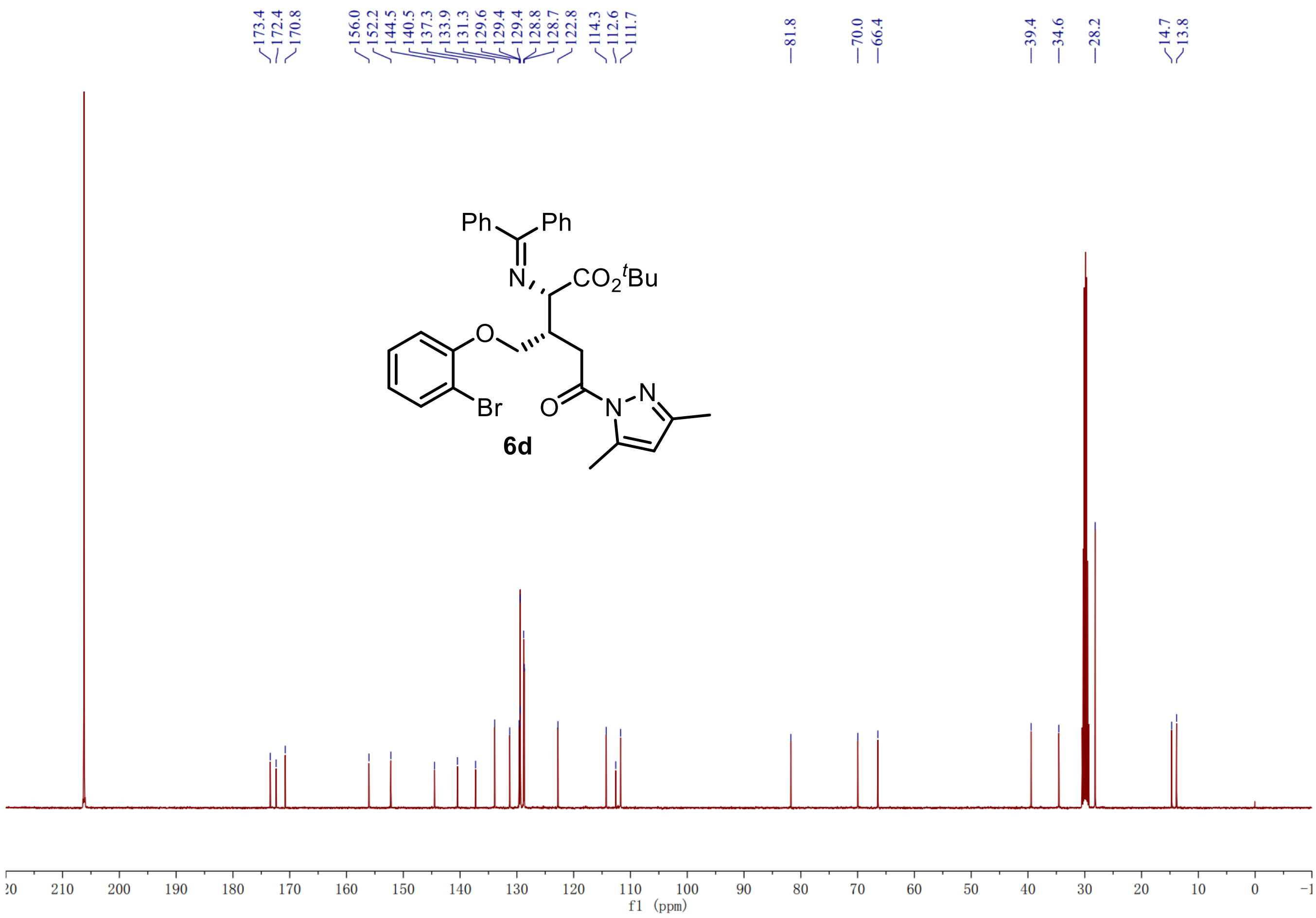
173.4
172.4
170.8
156.0
152.2
144.5
140.5
137.3
133.9
131.3
129.6
129.4
129.4
128.8
128.7
122.8
114.3
112.6
111.7
81.8
70.0
66.4
39.4
34.6
28.2
14.7
13.8
Ph
Ph
N
CO2tBu
O
Br
O
N
N
6d
210 200 190 180 170 160 150 140 130 120 110 100 90 80 70 60 50 40 30 20 10 0
f1 (ppm)

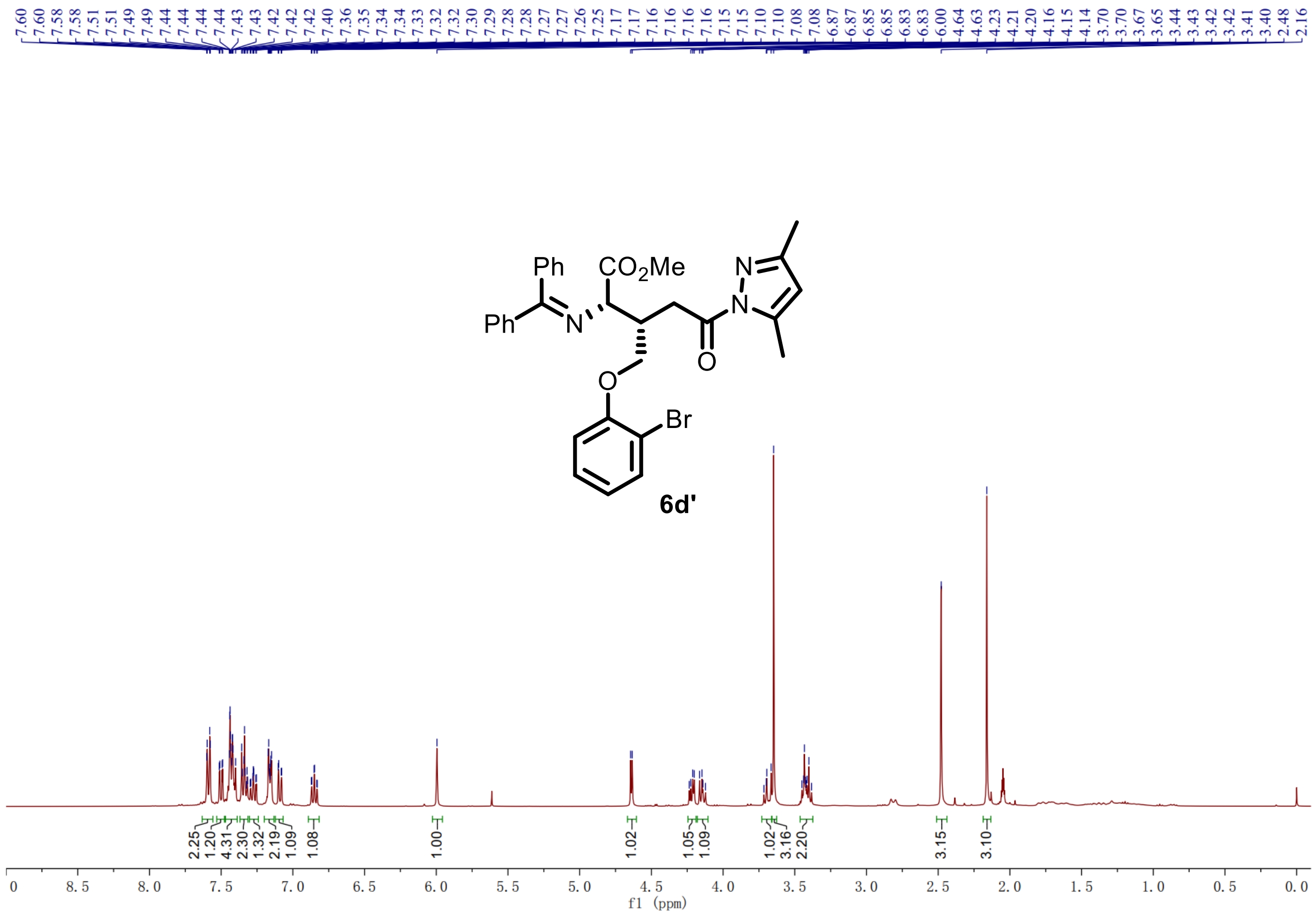
7.60 7.60 7.58 7.58 7.51 7.51 7.49 7.49 7.44 7.44 7.44 7.43 7.43 7.42 7.42 7.40 7.36 7.35 7.34 7.34 7.33 7.32 7.32 7.30 7.29 7.28 7.28 7.27 7.27 7.26 7.25 7.17 7.17 7.16 7.16 7.16 7.15 7.15 7.10 7.10 7.08 7.08 6.87 6.87 6.85 6.85 6.83 6.83 6.00 4.64 4.63 4.23 4.21 4.20 4.16 4.15 4.14 3.70 3.70 3.67 3.65 3.44 3.43 3.42 3.41 3.40 2.48 2.16
Ph
Ph
N
CO2Me
N
N
O
O
Br
6d'
2.25 1.20 4.31 2.30 1.32 2.19 1.09 1.08 1.00 1.02 1.05 1.09 1.02 3.16 2.20 3.15 3.10
8.5 8.0 7.5 7.0 6.5 6.0 5.5 5.0 4.5 4.0 3.5 3.0 2.5 2.0 1.5 1.0 0.5 0.0
f1 (ppm)

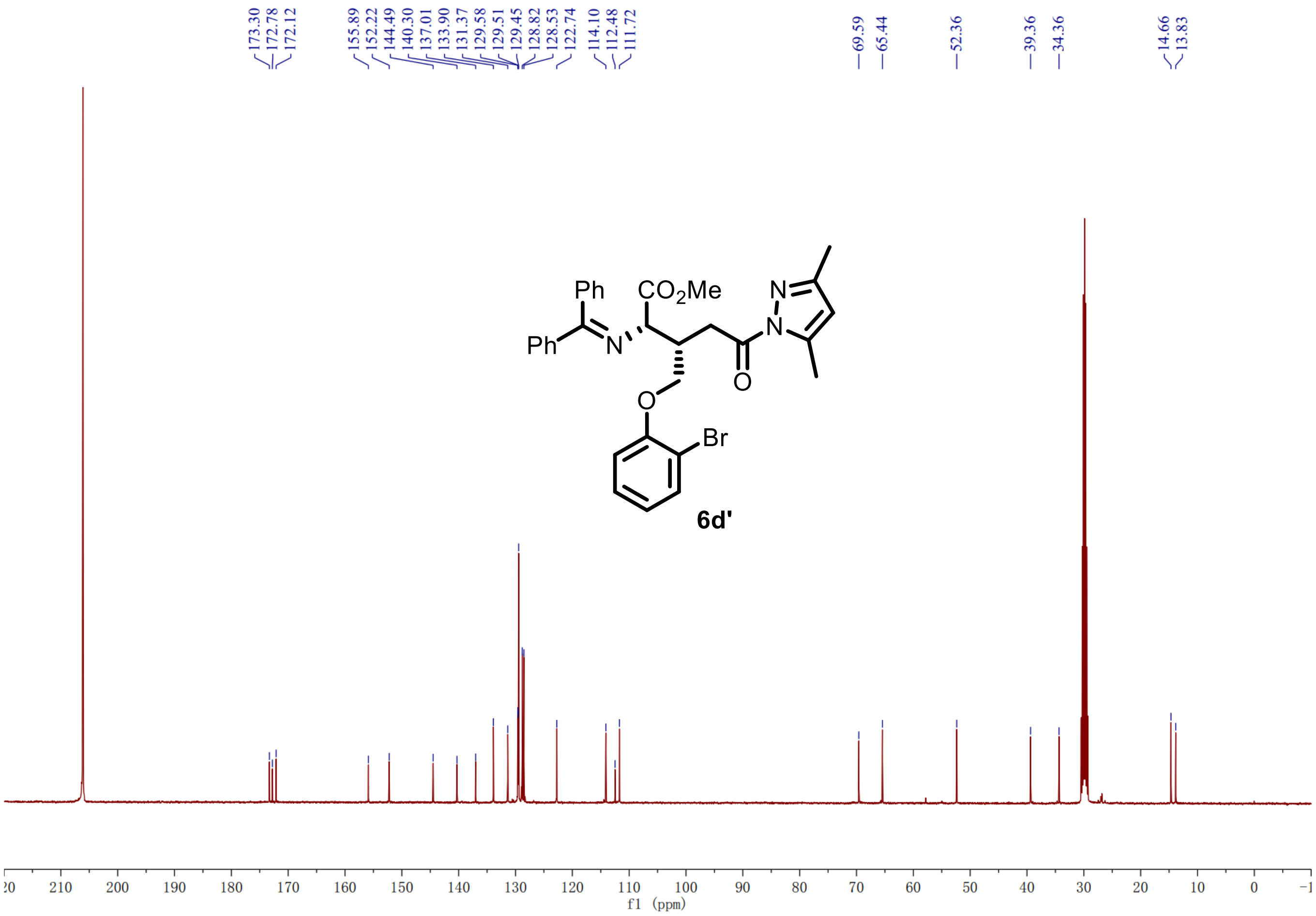
Ph
CO2Me
N
Ph
O
Br
6d'
f1 (ppm)

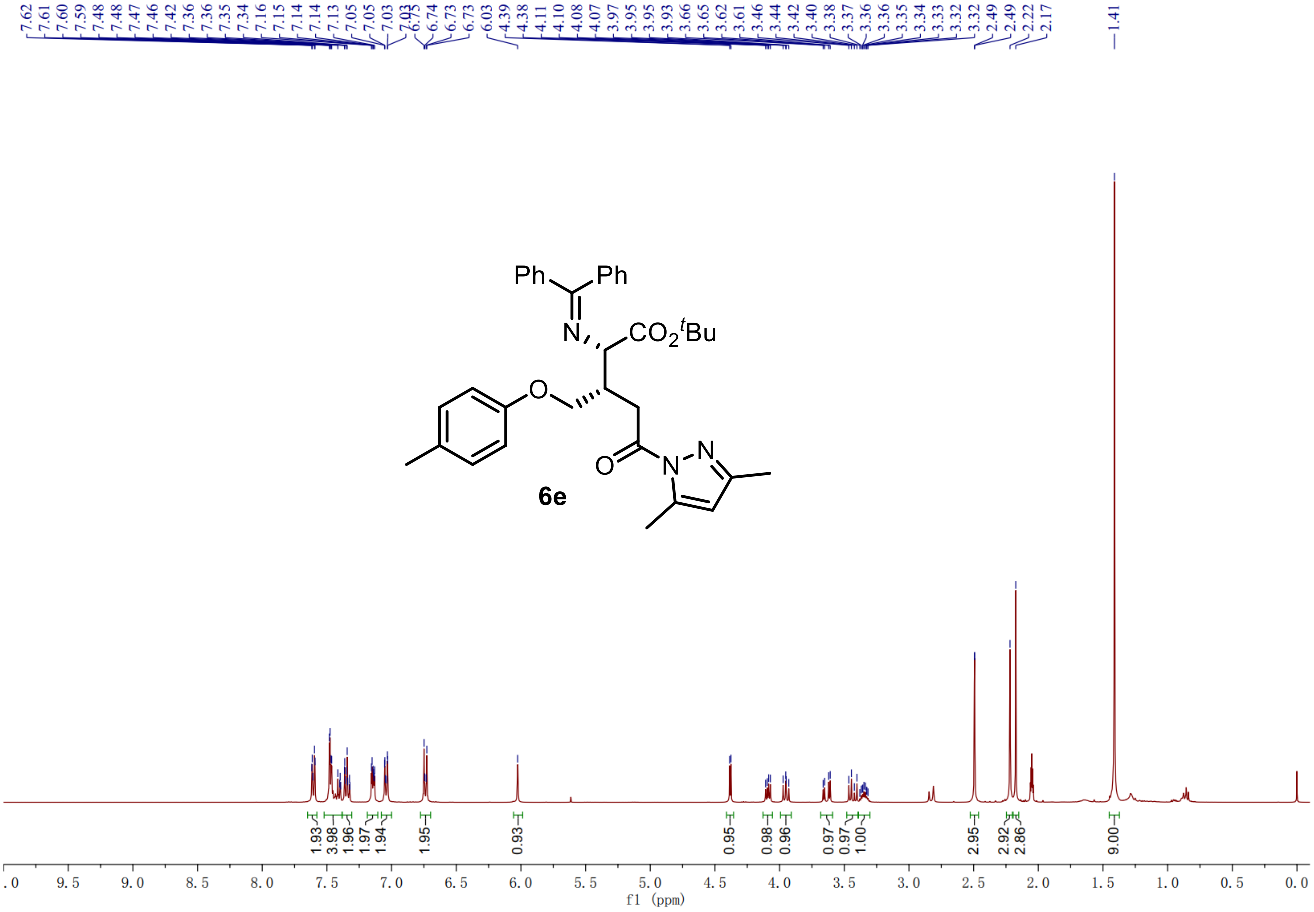
Ph
Ph
N
CO2tBu
O
6e
f1 (ppm)

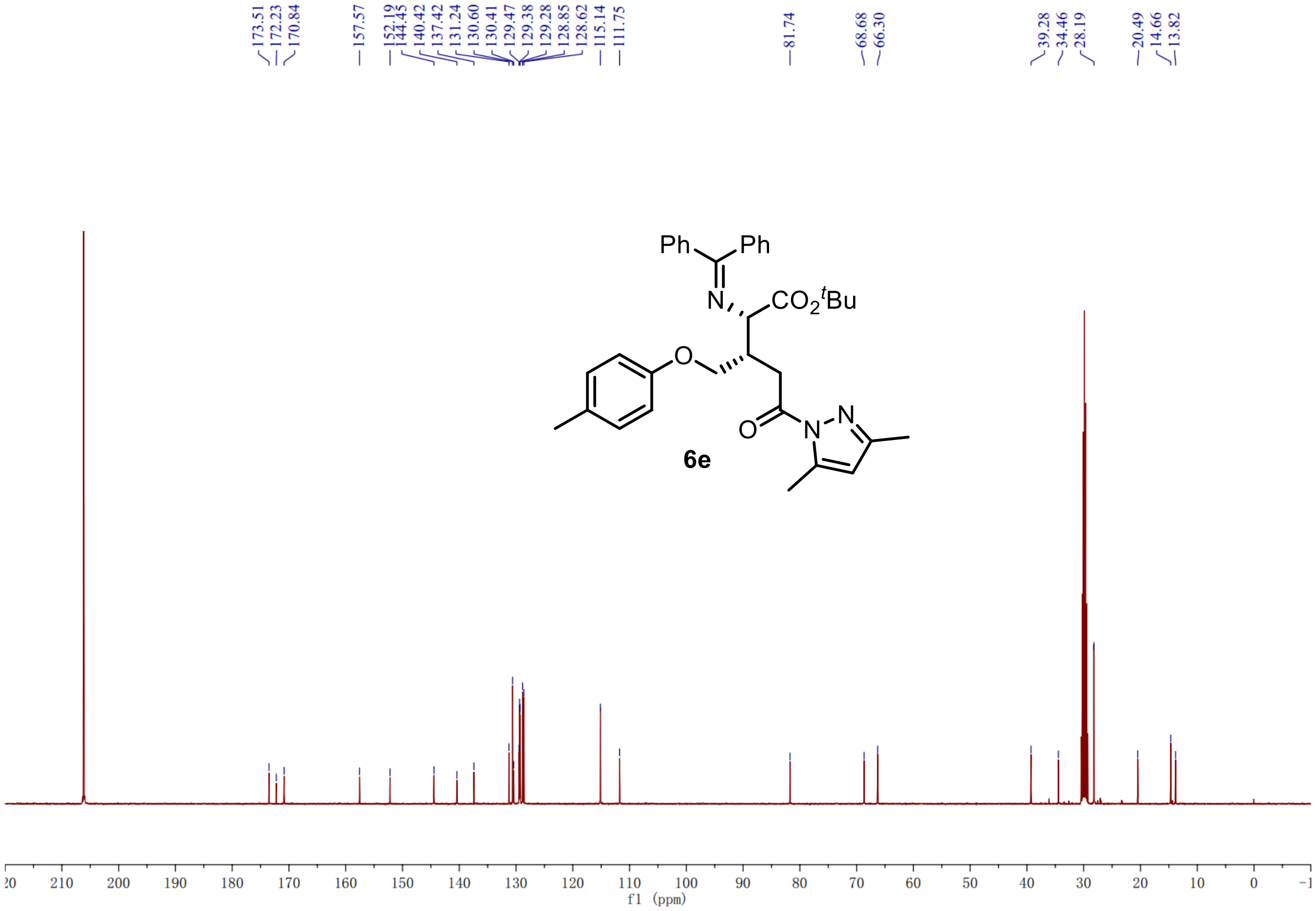
173.51
172.23
170.84
157.57
152.19
144.45
140.42
137.42
131.24
130.60
130.41
129.47
129.38
129.28
128.85
128.62
115.14
111.75
81.74
68.68
66.30
39.28
34.46
28.19
20.49
14.66
13.82
Ph
Ph
N
$CO_2{}^tBu$
O
O
N
N
6e
f1 (ppm)

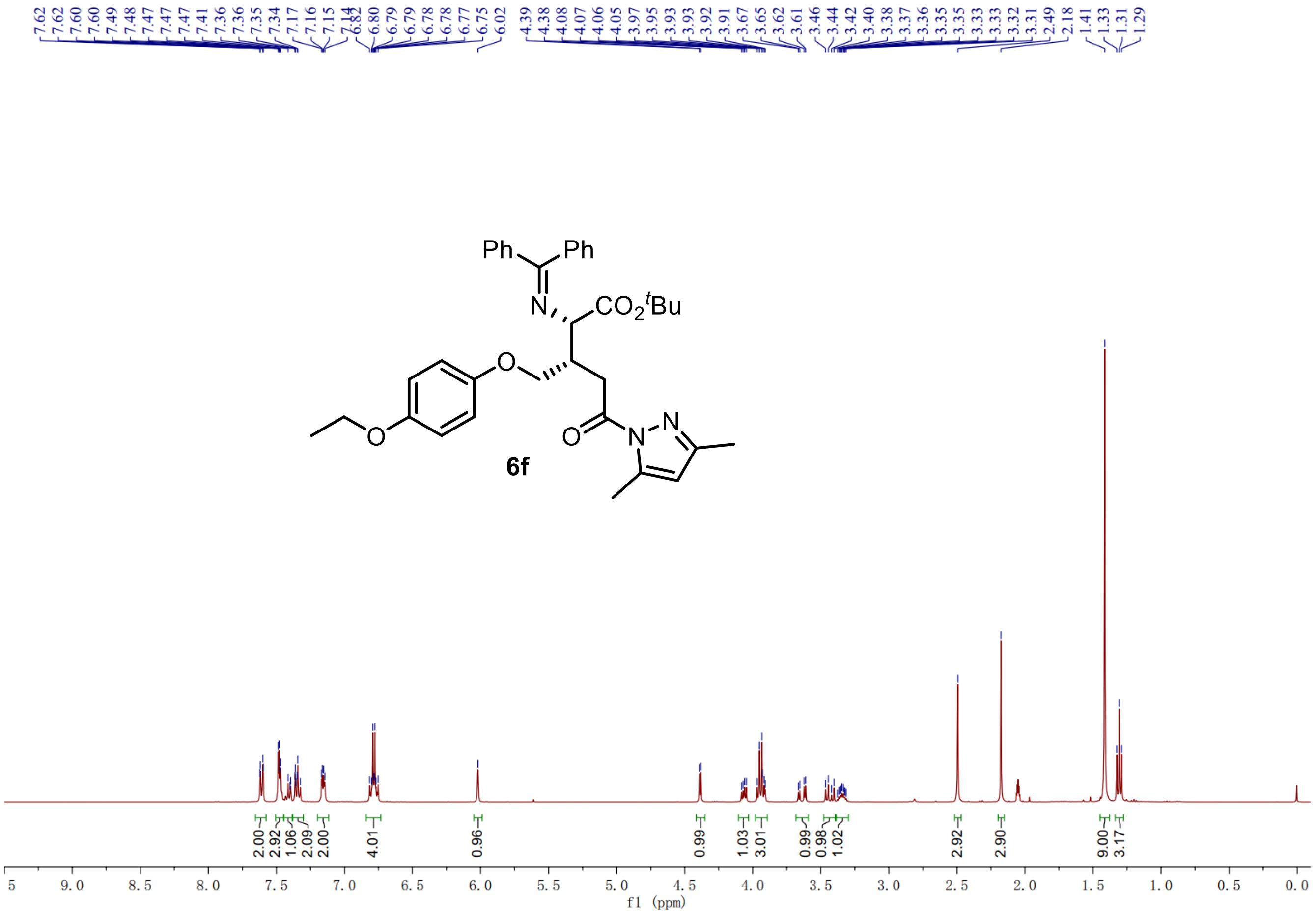
7.62
7.62
7.60
7.60
7.49
7.48
7.47
7.47
7.47
7.41
7.36
7.36
7.35
7.34
7.17
7.16
7.15
7.14
6.82
6.80
6.79
6.79
6.78
6.78
6.77
6.75
6.02
4.39
4.38
4.08
4.07
4.06
4.05
3.97
3.95
3.93
3.93
3.92
3.91
3.67
3.65
3.62
3.61
3.46
3.44
3.42
3.40
3.38
3.37
3.36
3.35
3.35
3.33
3.33
3.32
3.31
2.49
2.18
1.41
1.33
1.31
1.29
Ph
Ph
N
$CO_2{}^tBu$
O
O
O
N
N
6f
2.00
2.92
1.06
2.09
2.00
4.01
0.96
0.99
1.03
3.01
0.99
0.98
1.02
2.92
2.90
9.00
3.17
f1 (ppm)

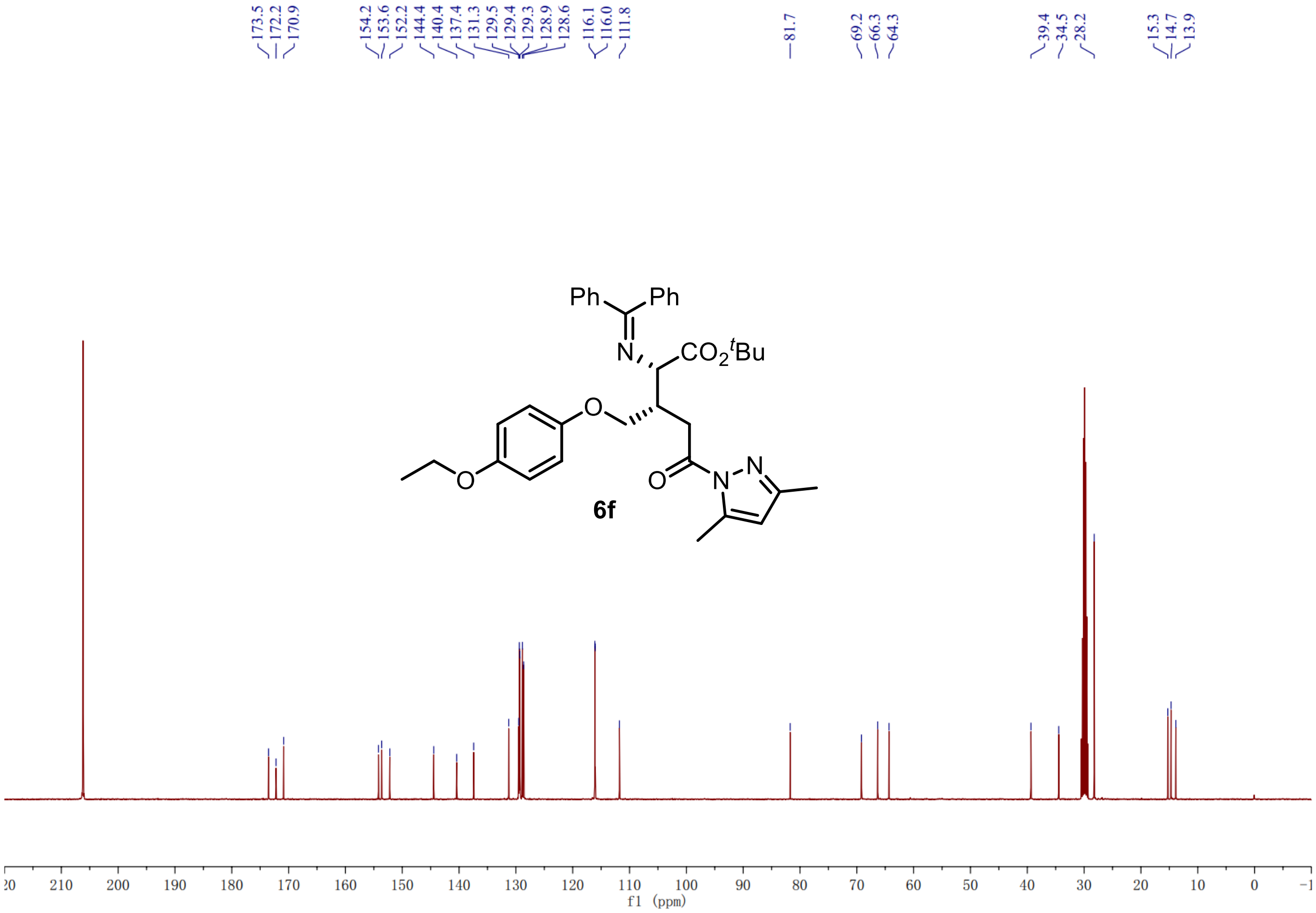

173.5
172.2
170.9
154.2
153.6
152.2
144.4
140.4
137.4
131.3
129.5
129.4
129.3
128.9
128.6
116.1
116.0
111.8
81.7
69.2
66.3
64.3
39.4
34.5
28.2
15.3
14.7
13.9
Ph
Ph
N
$CO_2{}^tBu$
O
O
O
N
N
6f
f1 (ppm)

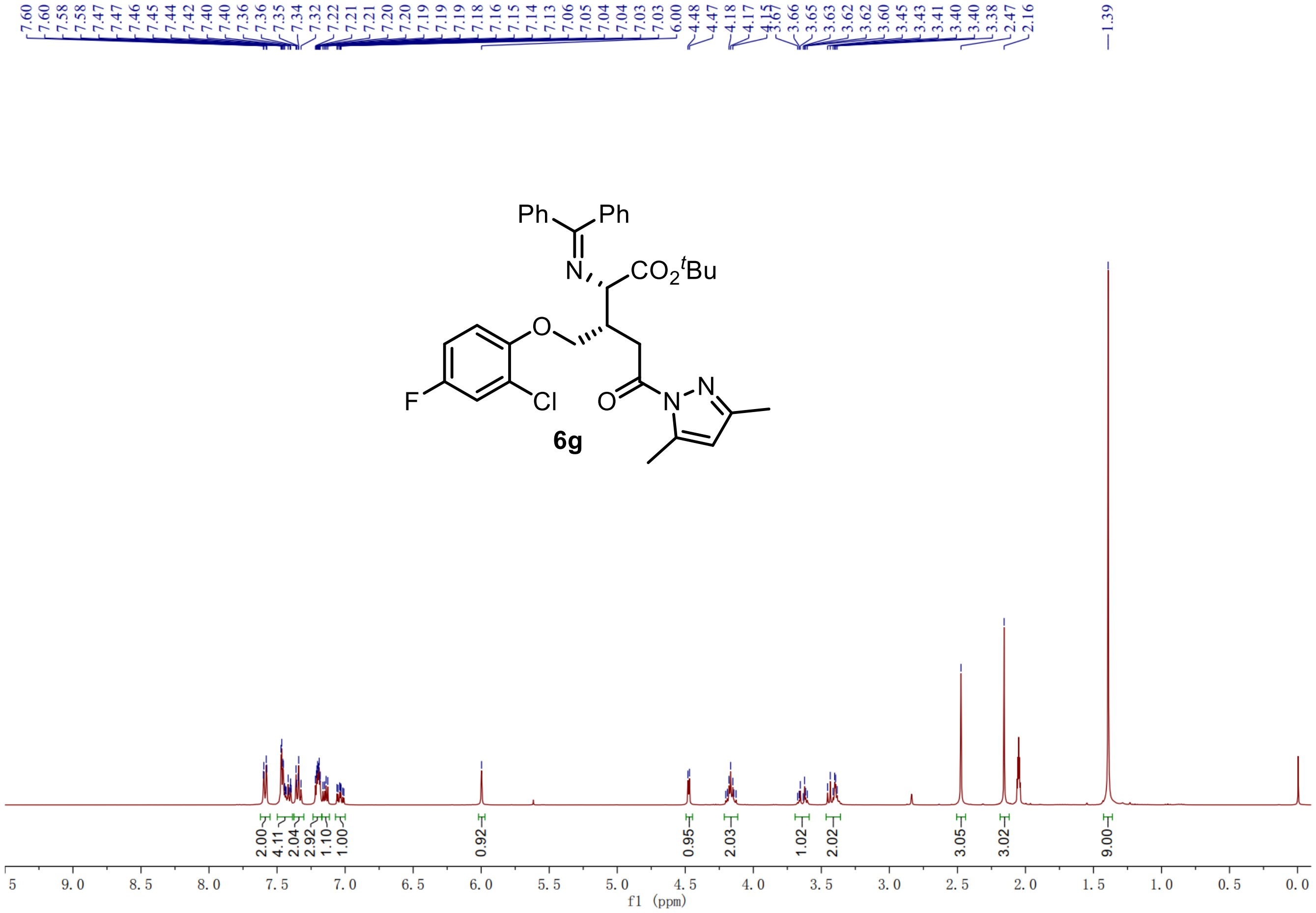

7.60
7.60
7.58
7.58
7.47
7.46
7.45
7.44
7.42
7.40
7.36
7.36
7.35
7.34
7.32
7.22
7.21
7.21
7.20
7.20
7.19
7.19
7.18
7.16
7.15
7.14
7.13
7.06
7.05
7.04
7.04
7.03
6.00
4.48
4.47
4.18
4.17
4.15
3.67
3.66
3.65
3.63
3.62
3.60
3.45
3.43
3.41
3.40
3.40
3.38
2.47
2.16
1.39
Ph
Ph
N
$CO_2{}^tBu$
O
F
Cl
O
N
N
6g
2.00
4.11
2.04
2.92
1.10
1.00
0.92
0.95
2.03
1.02
2.02
3.05
3.02
9.00
f1 (ppm)

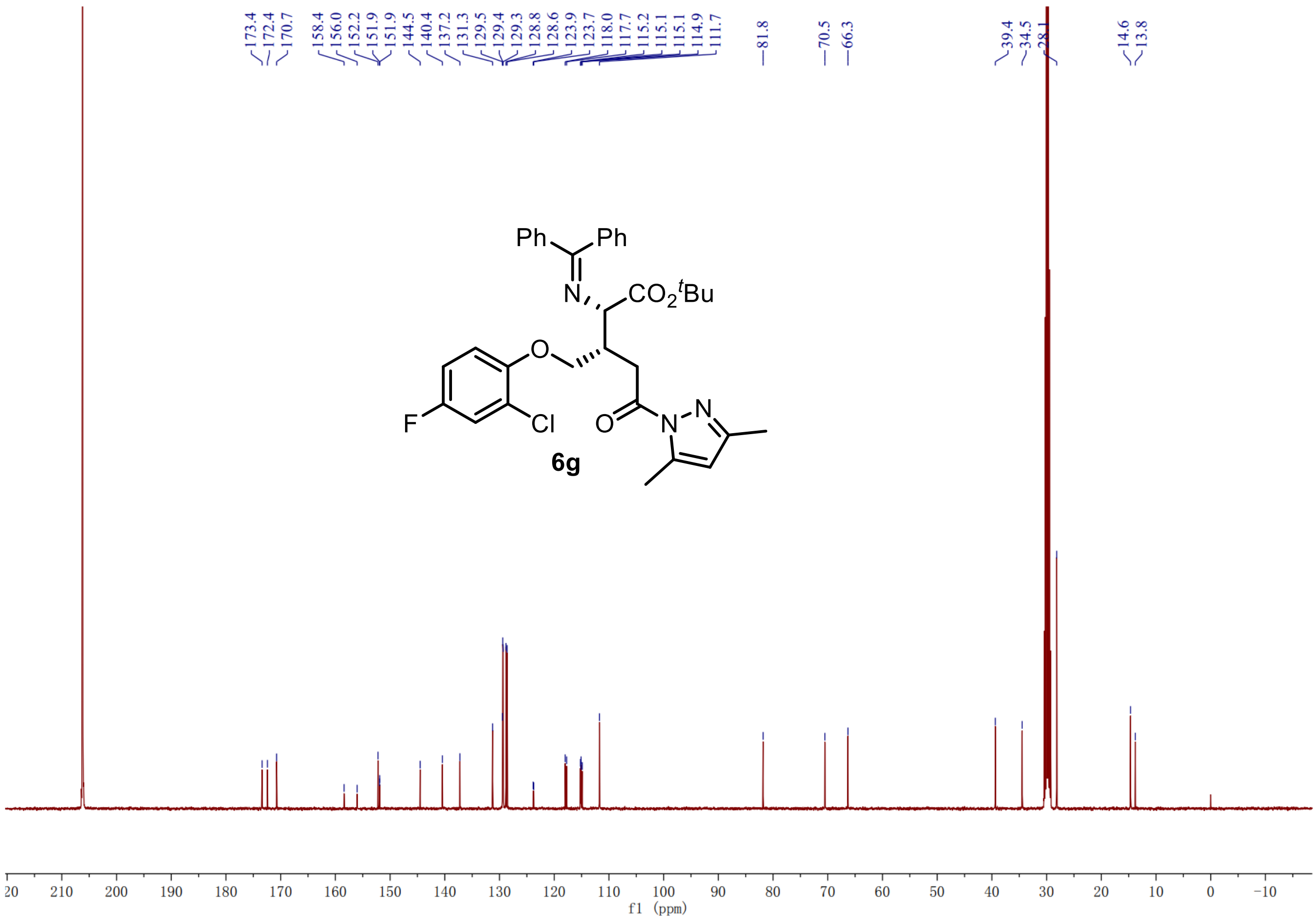
173.4
172.4
170.7
158.4
156.0
152.2
151.9
151.9
144.5
140.4
137.2
131.3
129.5
129.4
129.3
128.8
128.6
123.9
123.7
118.0
117.7
115.2
115.1
115.1
114.9
111.7
81.8
70.5
66.3
39.4
34.5
28.1
14.6
13.8
Ph
Ph
N
CO2tBu
O
F
Cl
O
N
N
6g
f1 (ppm)

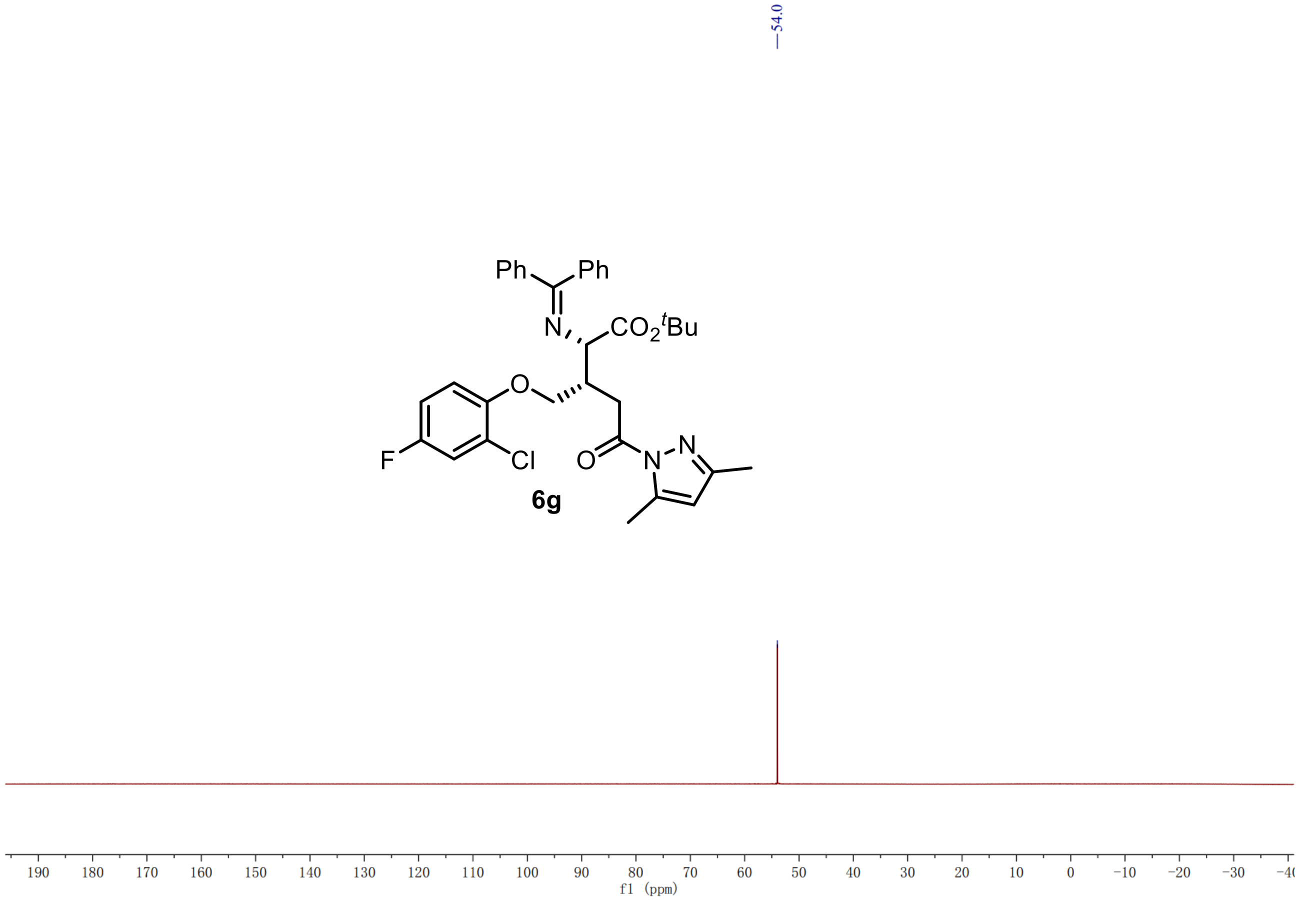
54.0
Ph
Ph
N
CO2tBu
O
F
Cl
O
N
N
6g
f1 (ppm)

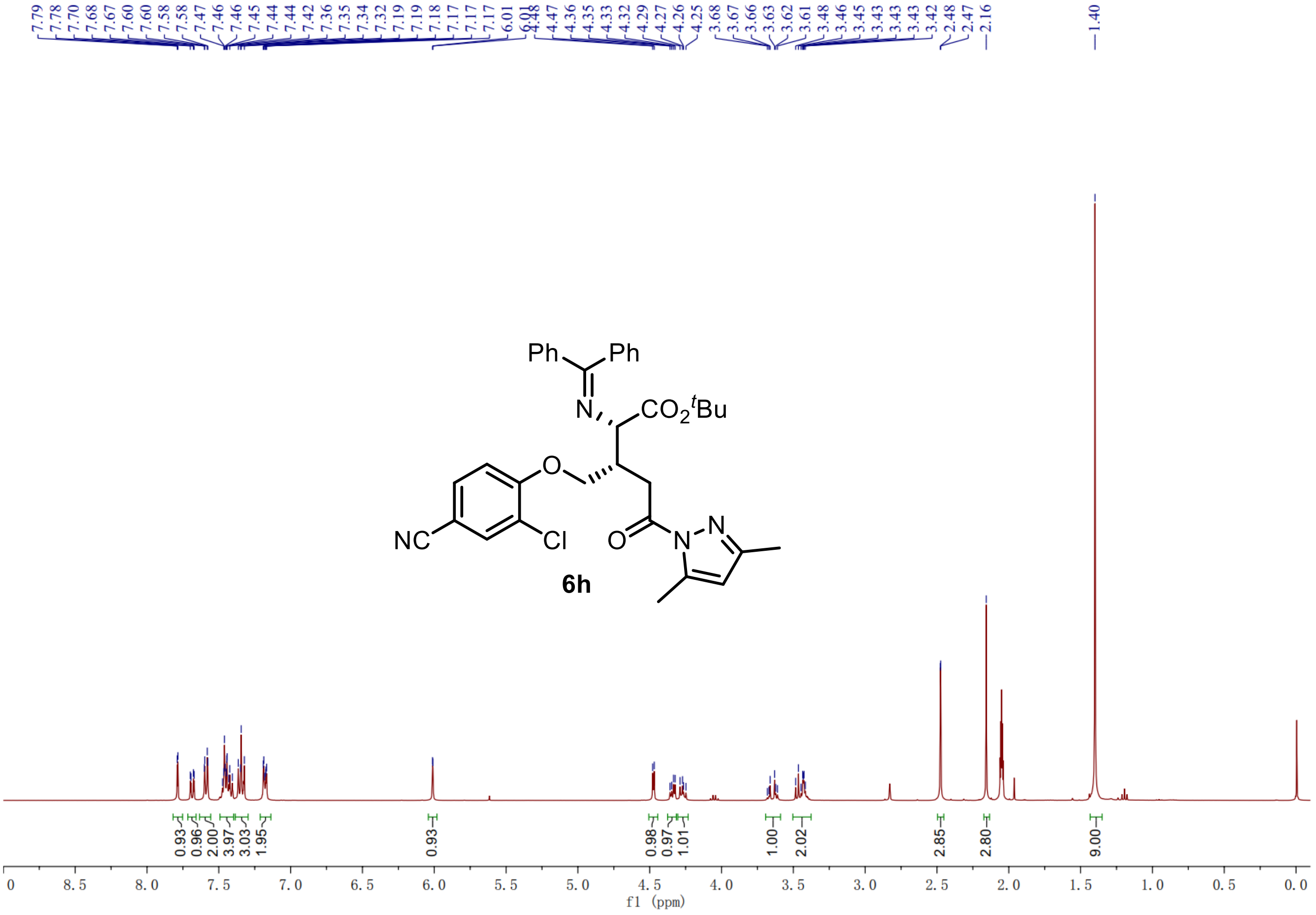
7.79 7.78 7.70 7.68 7.67 7.60 7.60 7.58 7.58 7.47 7.46 7.46 7.45 7.44 7.44 7.42 7.36 7.35 7.34 7.32 7.19 7.18 7.17 7.17 7.17 6.01 4.48 4.47 4.36 4.35 4.33 4.32 4.29 4.27 4.26 4.25 3.68 3.67 3.66 3.63 3.62 3.61 3.48 3.46 3.45 3.43 3.43 3.43 3.42 2.48 2.47 2.16 1.40
Ph
Ph
N
$CO_2{}^tBu$
O
NC
Cl
O
N
N
6h
0.93 0.96 2.00 3.97 3.03 1.95 0.93 0.98 0.97 1.01 1.00 2.02 2.85 2.80 9.00
f1 (ppm)

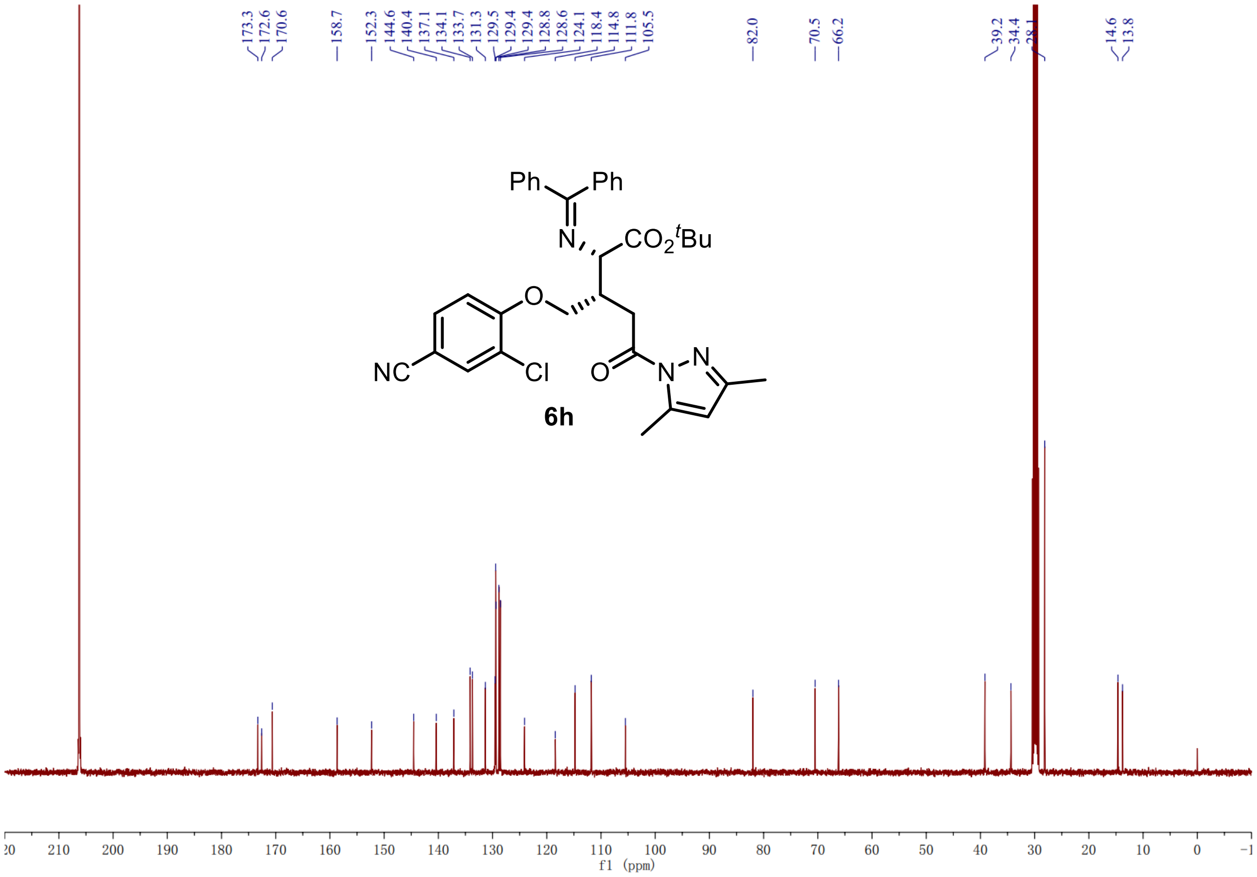
173.3 172.6 170.6 158.7 152.3 144.6 140.4 137.1 134.1 133.7 131.3 129.5 129.4 129.4 128.8 128.6 124.1 118.4 114.8 111.8 105.5 82.0 70.5 66.2 39.2 34.4 28.1 14.6 13.8
Ph
Ph
N
$CO_2{}^tBu$
O
NC
Cl
O
N
N
6h
f1 (ppm)

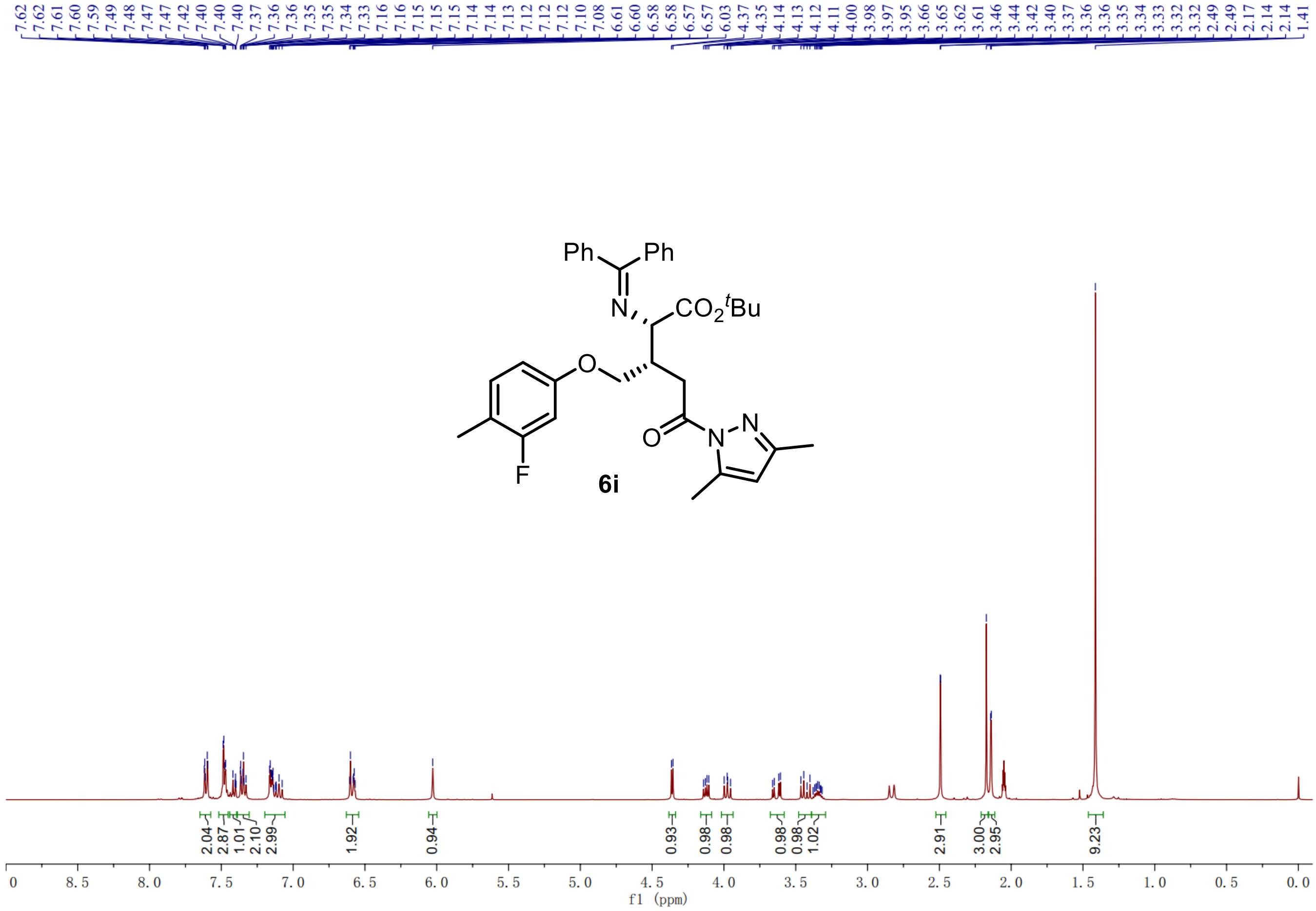
7.62 7.61 7.60 7.59 7.49 7.48 7.47 7.42 7.40 7.40 7.37 7.36 7.36 7.35 7.34 7.33 7.16 7.16 7.15 7.15 7.14 7.14 7.13 7.12 7.12 7.12 7.10 7.08 6.61 6.60 6.58 6.57 6.57 6.03 4.37 4.35 4.14 4.13 4.12 4.11 4.00 3.98 3.97 3.95 3.66 3.65 3.62 3.61 3.46 3.44 3.42 3.40 3.37 3.36 3.36 3.35 3.34 3.33 3.32 3.32 2.49 2.17 2.14 1.41
Ph Ph
N
CO2tBu
O
F
N N
O
6i
2.04 2.87 1.01 2.10 2.99 1.92 0.94 0.93 0.98 0.98 0.98 0.98 1.02 2.91 3.00 2.95 9.23
f1 (ppm)

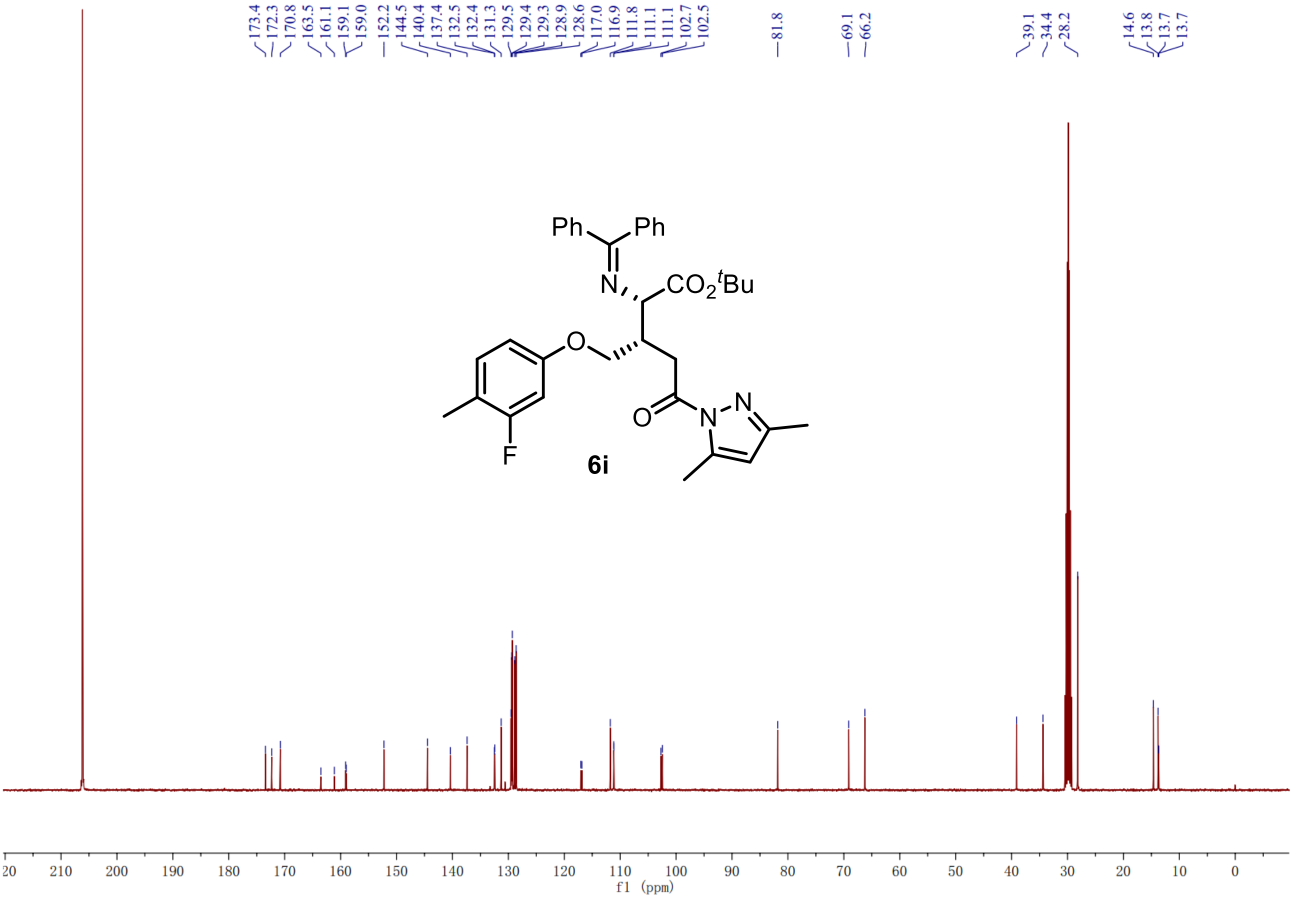
173.4 172.3 170.8 163.5 161.1 159.1 159.0 152.2 144.5 140.4 137.4 132.5 132.4 131.3 129.5 129.4 129.3 128.9 128.6 117.0 116.9 111.8 111.1 111.1 102.7 102.5 81.8 69.1 66.2 39.1 34.4 28.2 14.6 13.8 13.7 13.7
Ph Ph
N
CO2tBu
O
F
N N
O
6i
f1 (ppm)

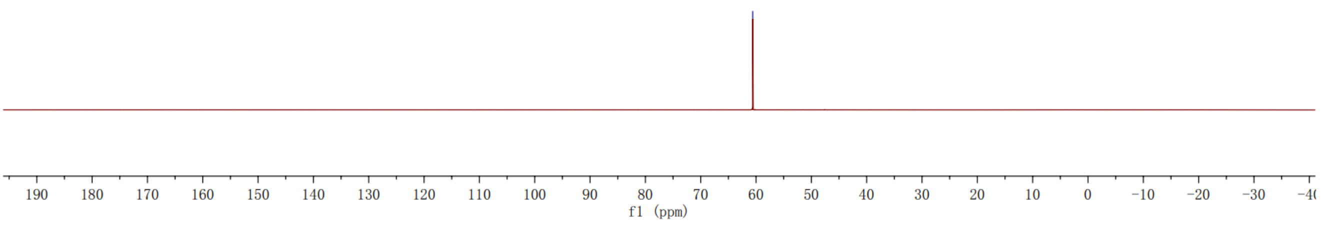
—60.6
190
180
170
160
150
140
130
120
110
100
90
80
70
60
50
40
30
20
10
0
−10
−20
−30
f1 (ppm)

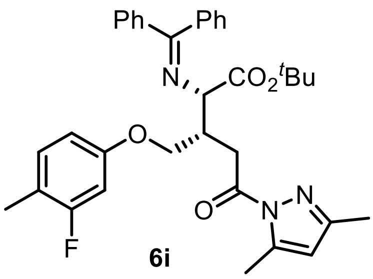
Ph
Ph
N
CO2tBu
O
F
O
N
N

6i

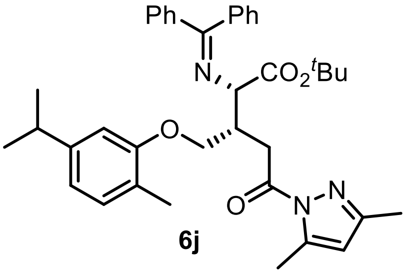
Ph
Ph
N
CO2tBu
O
O
N
N

6j

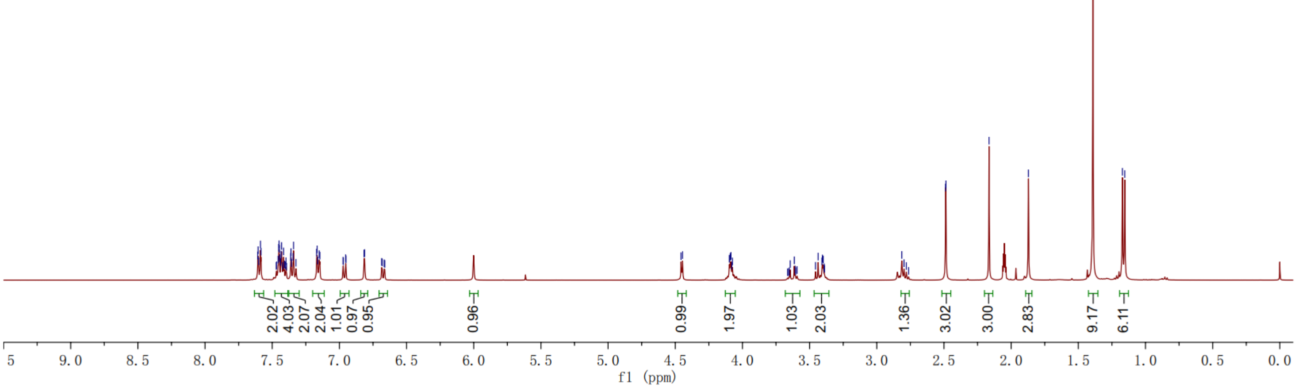
7.61
7.61
7.60
7.59
7.58
7.47
7.47
7.46
7.45
7.45
7.44
7.44
7.43
7.43
7.42
7.42
7.41
7.40
7.40
7.39
7.36
7.36
7.35
7.34
7.32
7.17
7.17
7.16
7.15
7.15
6.97
6.97
6.95
6.95
6.82
6.81
6.69
6.68
6.67
6.66
4.46
4.44
4.10
4.09
4.09
4.08
4.07
3.64
3.61
3.61
3.60
3.59
3.46
3.44
3.41
3.40
3.40
3.39
3.39
2.81
2.80
2.78
2.76
2.49
2.49
2.16
1.87
1.39
1.17
1.15
2.02
4.03
2.07
2.04
1.01
0.97
0.95
0.96
0.99
1.97
1.03
2.03
1.36
3.02
3.00
2.83
9.17
6.11
9.0
8.5
8.0
7.5
7.0
6.5
6.0
5.5
5.0
4.5
4.0
3.5
3.0
2.5
2.0
1.5
1.0
0.5
0.0
f1 (ppm)

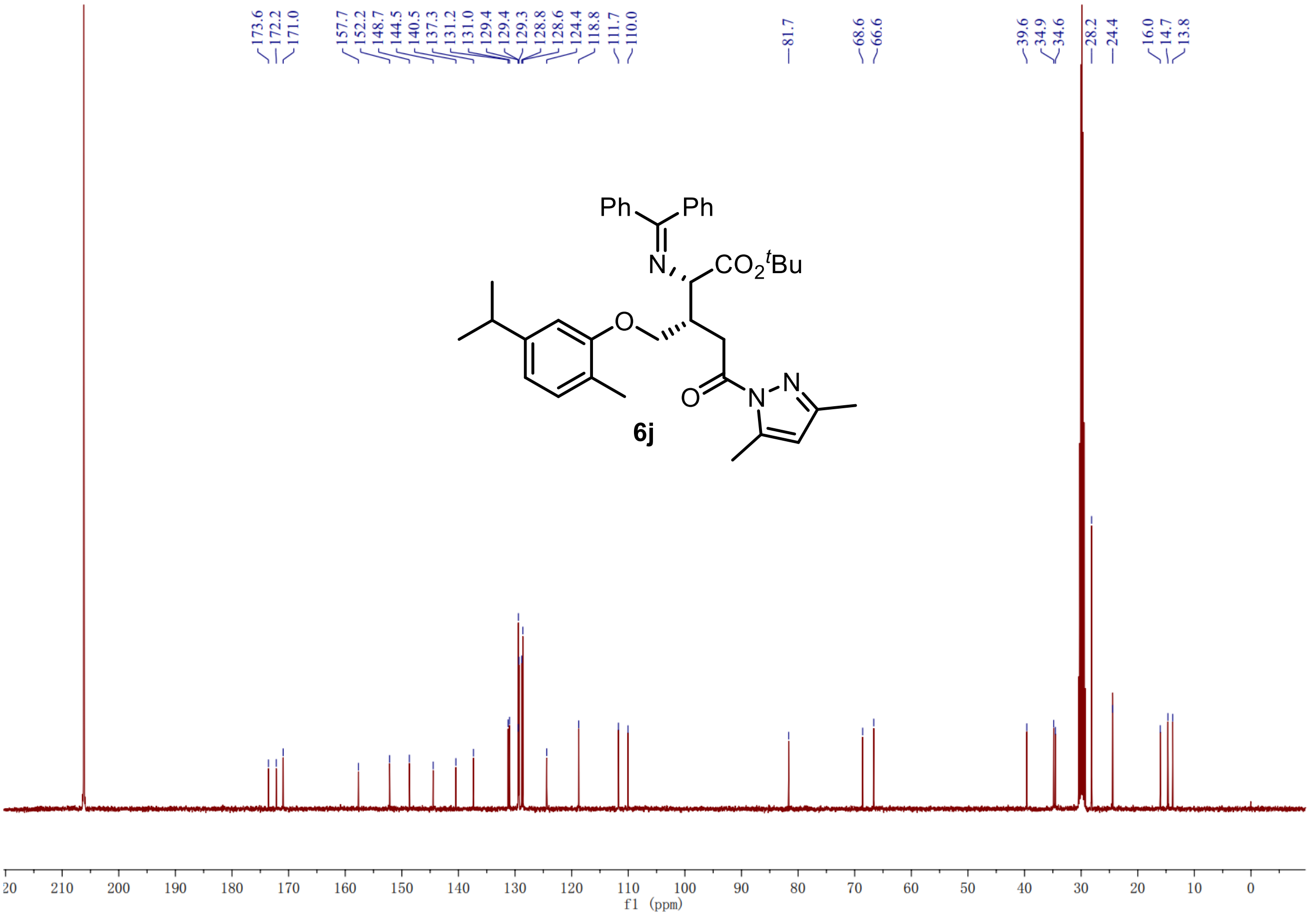
173.6
172.2
171.0
157.7
152.2
148.7
144.5
140.5
137.3
131.2
131.0
129.4
129.4
129.3
128.8
128.6
124.4
118.8
111.7
110.0
81.7
68.6
66.6
39.6
34.9
34.6
28.2
24.4
16.0
14.7
13.8
Ph
Ph
N
$CO_2{}^tBu$
O
O
N
N
6j
f1 (ppm)

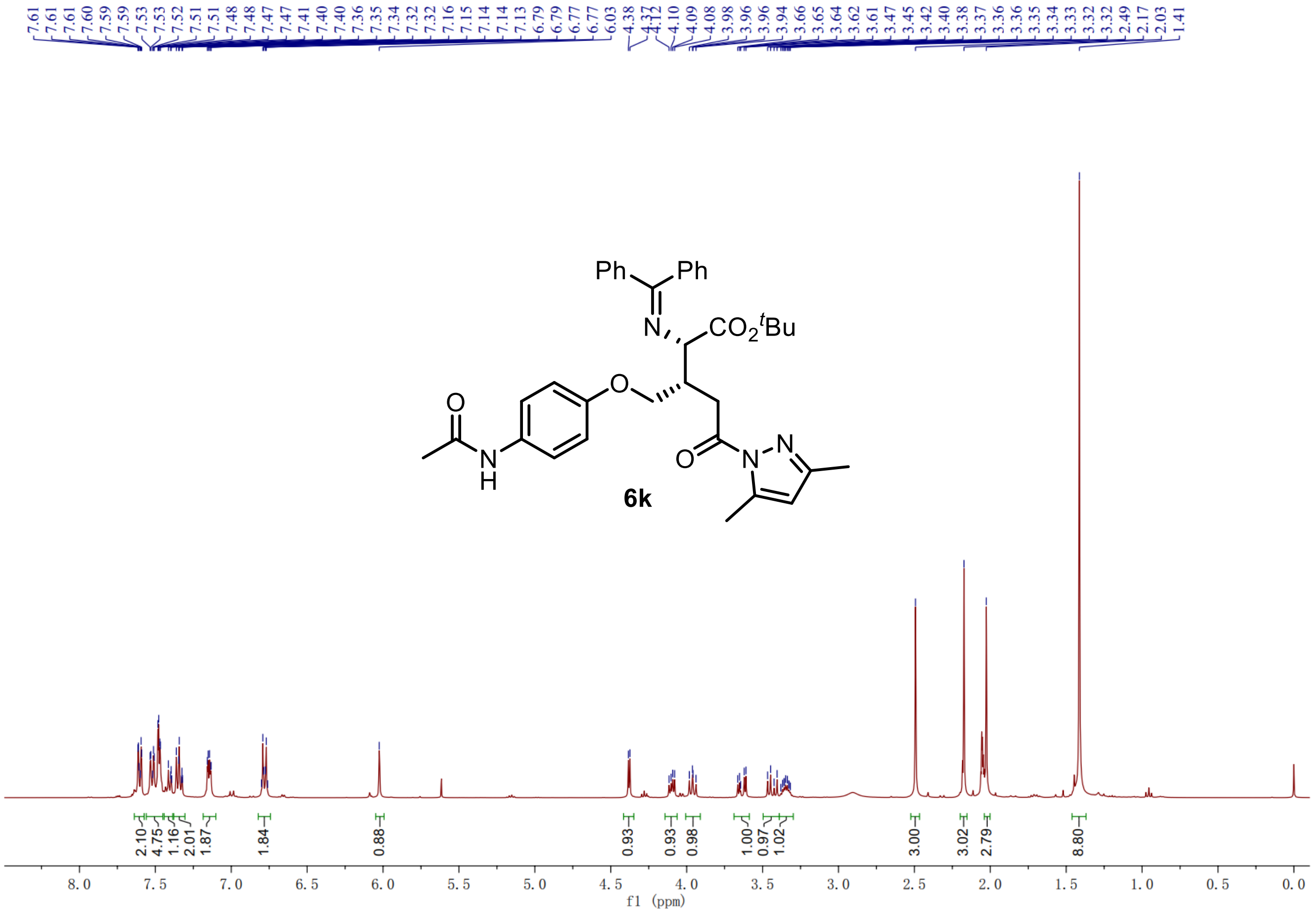
7.61 7.61 7.60 7.59 7.59 7.53 7.53 7.52 7.51 7.51 7.48 7.48 7.47 7.47 7.41 7.40 7.40 7.36 7.35 7.34 7.32 7.32 7.16 7.15 7.14 7.14 7.13 6.79 6.79 6.77 6.77 6.03 4.38 4.37 4.12 4.10 4.09 4.08 3.98 3.96 3.96 3.94 3.66 3.65 3.64 3.62 3.61 3.47 3.45 3.42 3.40 3.38 3.37 3.36 3.36 3.35 3.34 3.33 3.33 3.32 3.32 2.49 2.17 2.03 1.41
Ph
Ph
N
$CO_2{}^tBu$
O
O
N
H
O
N
N
6k
2.10 4.75 1.16 2.01 1.87 1.84 0.88 0.93 0.93 0.98 1.00 0.97 1.02 3.00 3.02 2.79 8.80
f1 (ppm)

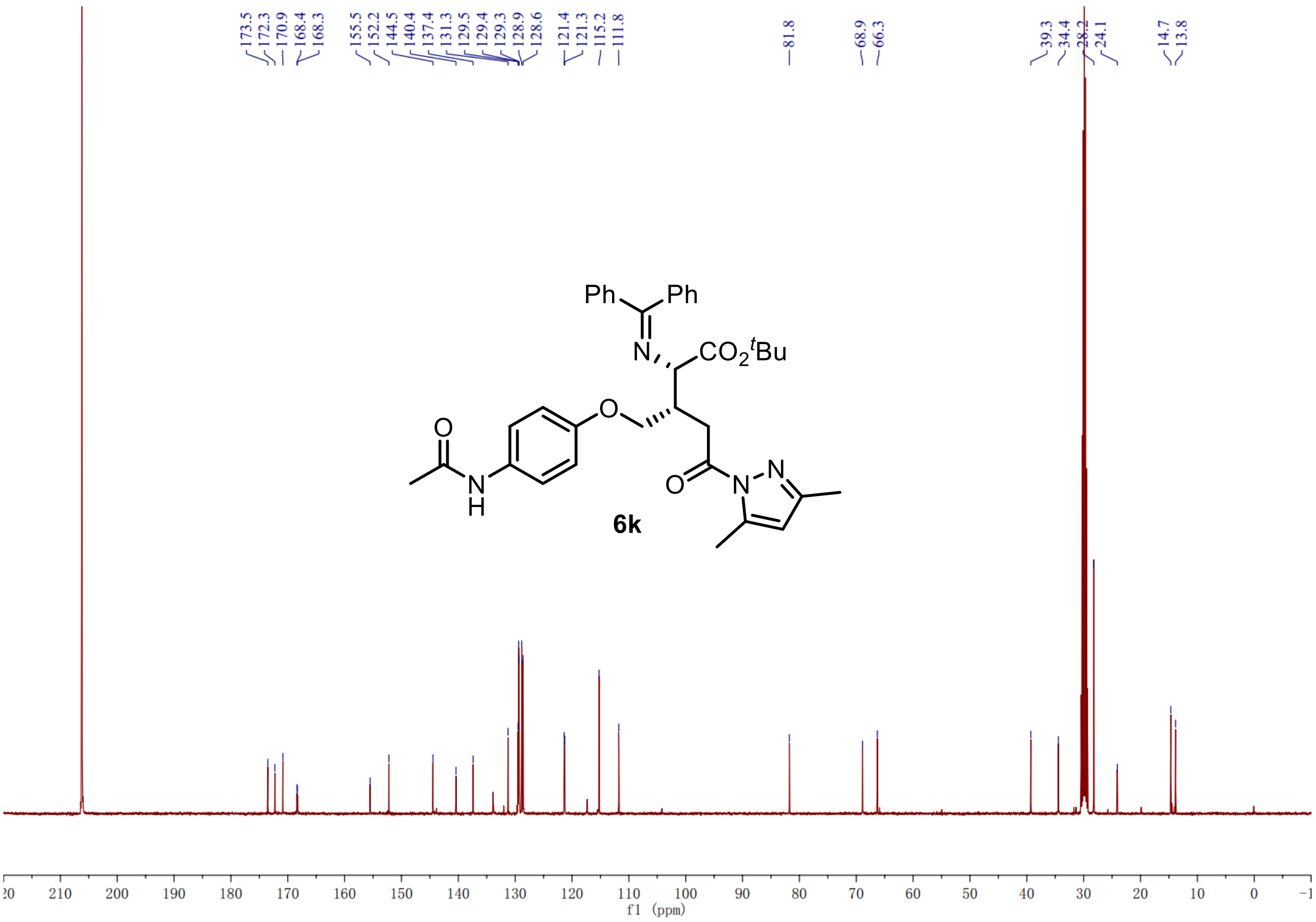
173.5
172.3
170.9
168.4
168.3
155.5
152.2
144.5
140.4
137.4
131.3
129.5
129.4
129.3
128.9
128.6
121.4
121.3
115.2
111.8
81.8
68.9
66.3
39.3
34.4
28.2
24.1
14.7
13.8
Ph
Ph
N
CO2tBu
O
O
N
H
O
N
N
f1 (ppm)

6k

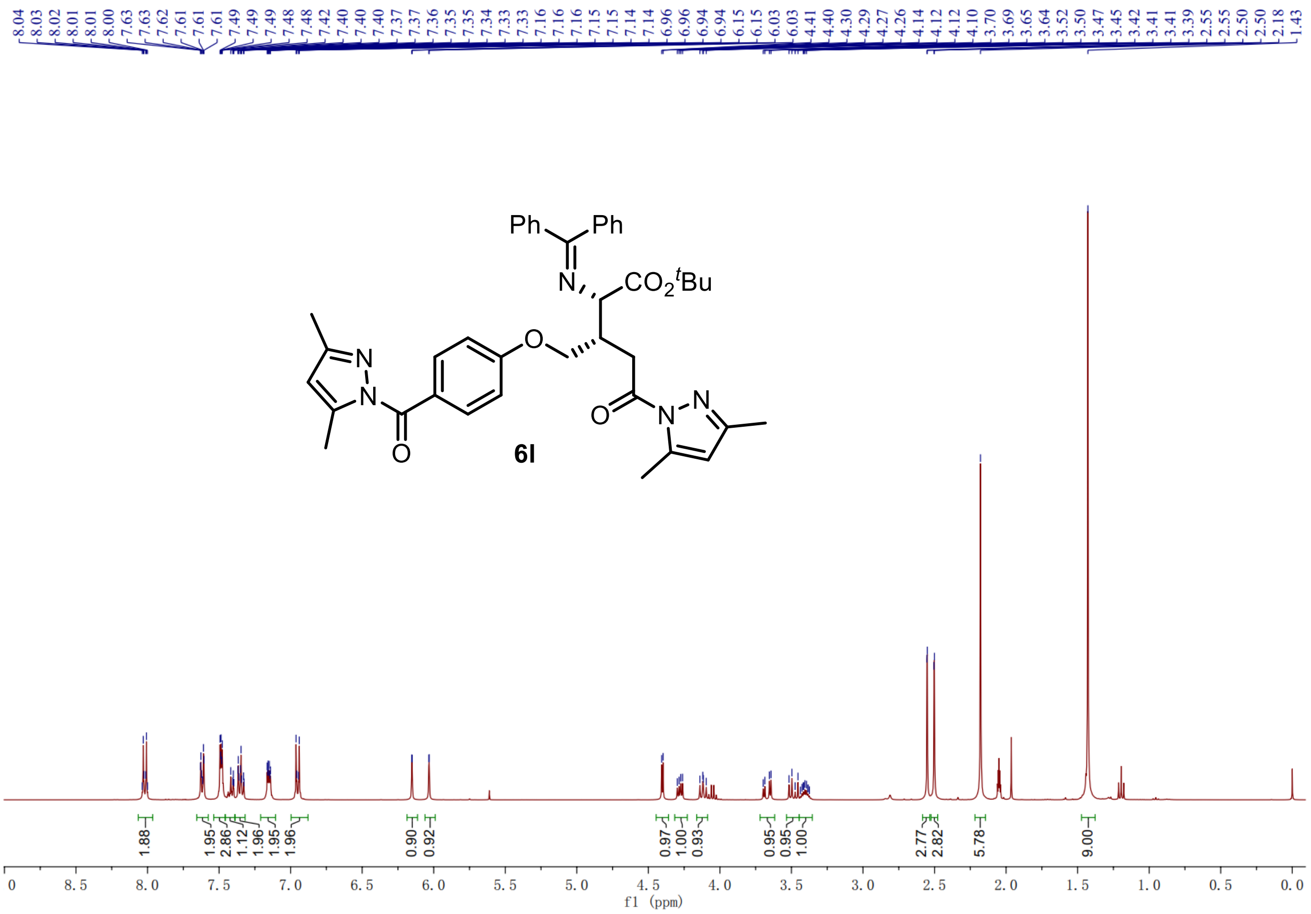
8.04
8.03
8.02
8.01
8.01
8.00
7.63
7.62
7.61
7.61
7.61
7.49
7.49
7.49
7.48
7.42
7.40
7.40
7.40
7.37
7.36
7.35
7.35
7.34
7.33
7.33
7.16
7.16
7.16
7.15
7.14
6.96
6.96
6.94
6.94
6.15
6.15
6.03
6.03
4.41
4.40
4.30
4.29
4.27
4.26
4.14
4.12
4.12
4.10
3.70
3.69
3.65
3.64
3.52
3.50
3.47
3.45
3.42
3.41
3.41
3.39
2.55
2.55
2.50
2.50
2.18
1.43
Ph
Ph
N
CO2tBu
O
N
N
O
O
N
N
1.88
1.95
2.86
1.12
1.96
1.95
1.96
0.90
0.92
0.97
1.00
0.93
0.95
0.95
1.00
2.77
2.82
5.78
9.00
f1 (ppm)

6l

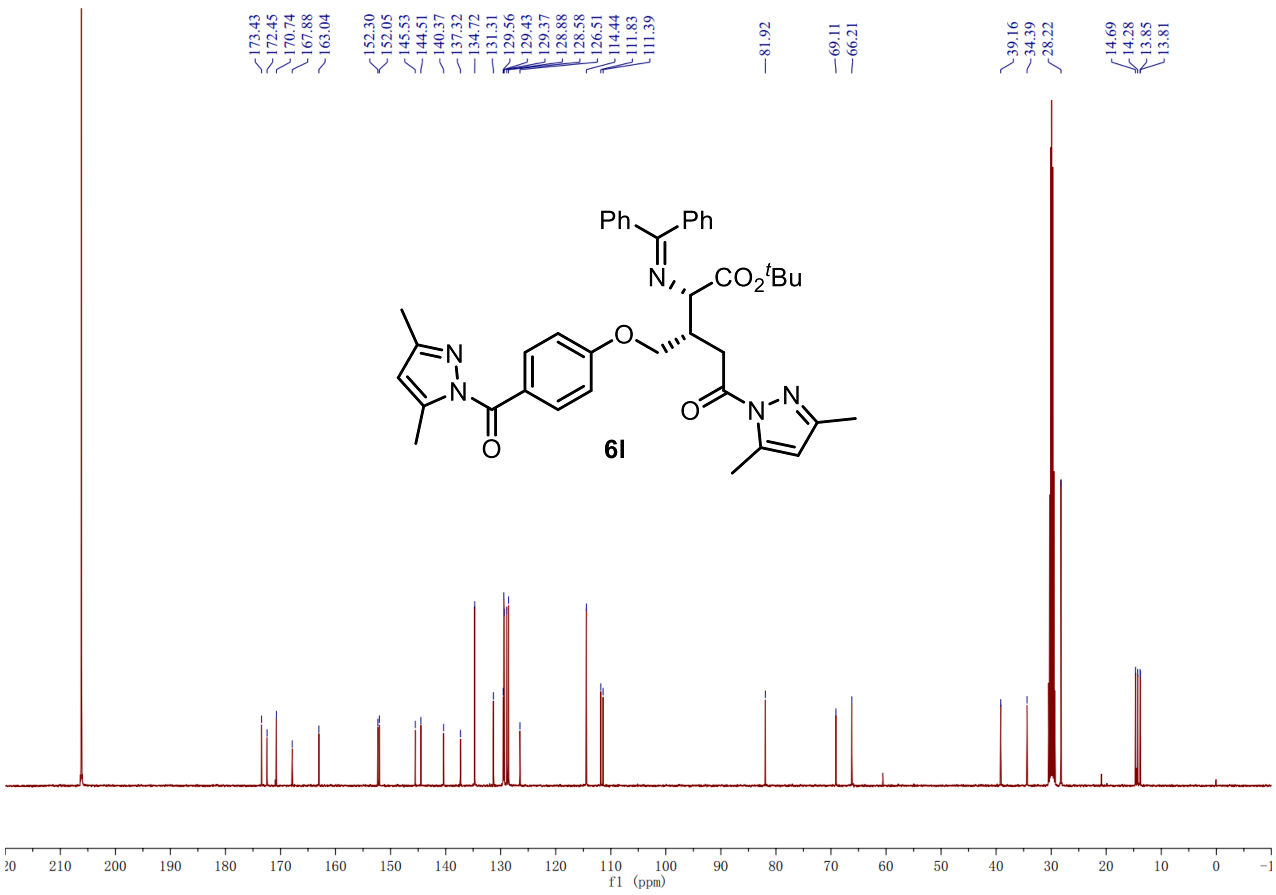

173.43
172.45
170.74
167.88
163.04
152.30
152.05
145.53
144.51
140.37
137.32
134.72
131.31
129.56
129.43
129.37
128.88
128.58
126.51
114.44
111.83
111.39
81.92
69.11
66.21
39.16
34.39
28.22
14.69
14.28
13.85
13.81
6l
f1 (ppm)

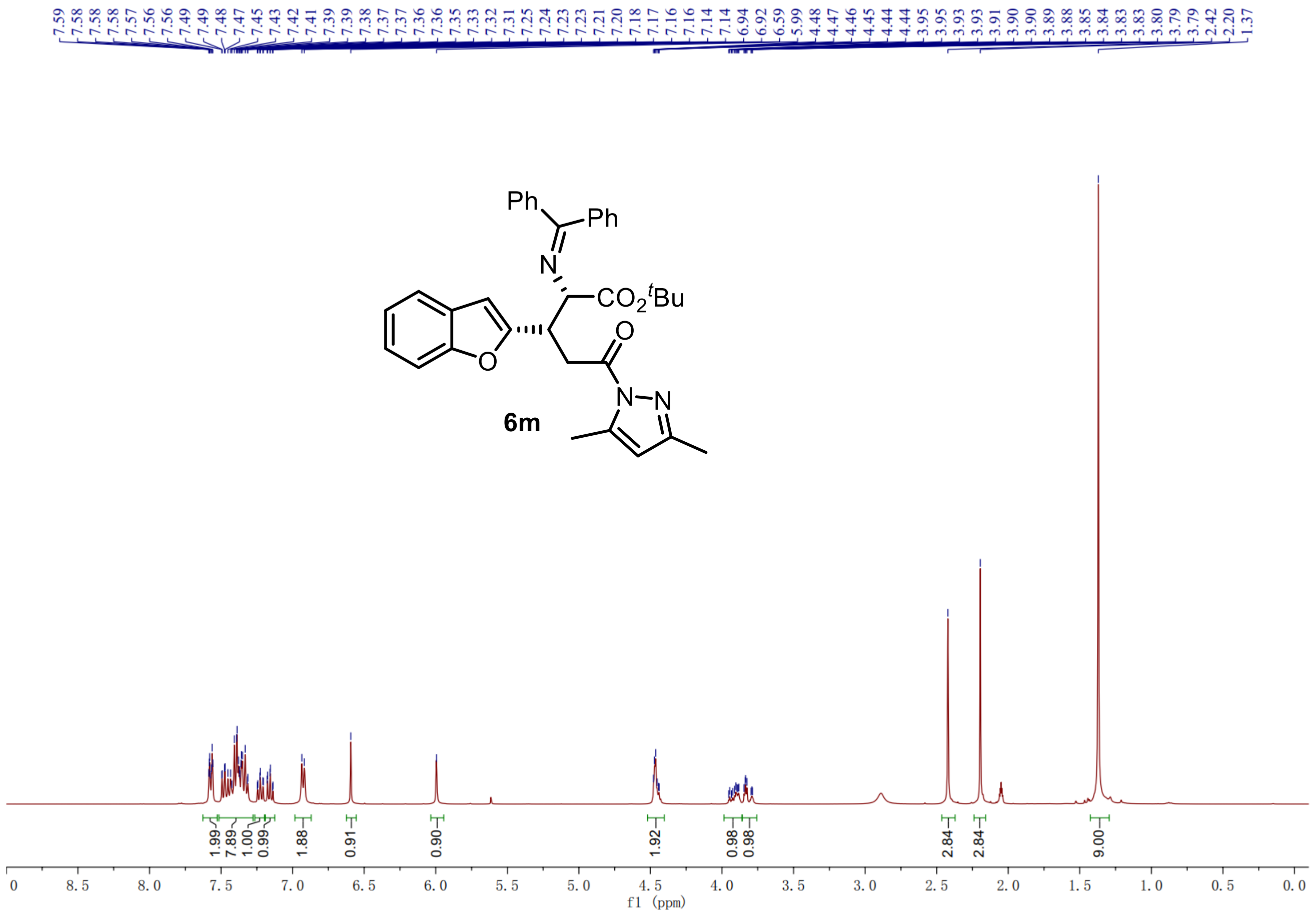

7.59
7.58
7.58
7.58
7.57
7.56
7.56
7.49
7.48
7.47
7.45
7.43
7.42
7.41
7.39
7.39
7.38
7.37
7.37
7.36
7.35
7.33
7.32
7.31
7.25
7.24
7.23
7.23
7.21
7.20
7.18
7.17
7.16
7.16
7.14
6.94
6.92
6.59
5.99
4.48
4.47
4.46
4.45
4.44
4.44
3.95
3.95
3.93
3.93
3.91
3.90
3.89
3.88
3.85
3.84
3.83
3.83
3.80
3.79
2.42
2.20
1.37
6m
1.99
7.89
1.00
0.99
1.88
0.91
0.90
1.92
0.98
0.98
2.84
2.84
9.00
f1 (ppm)

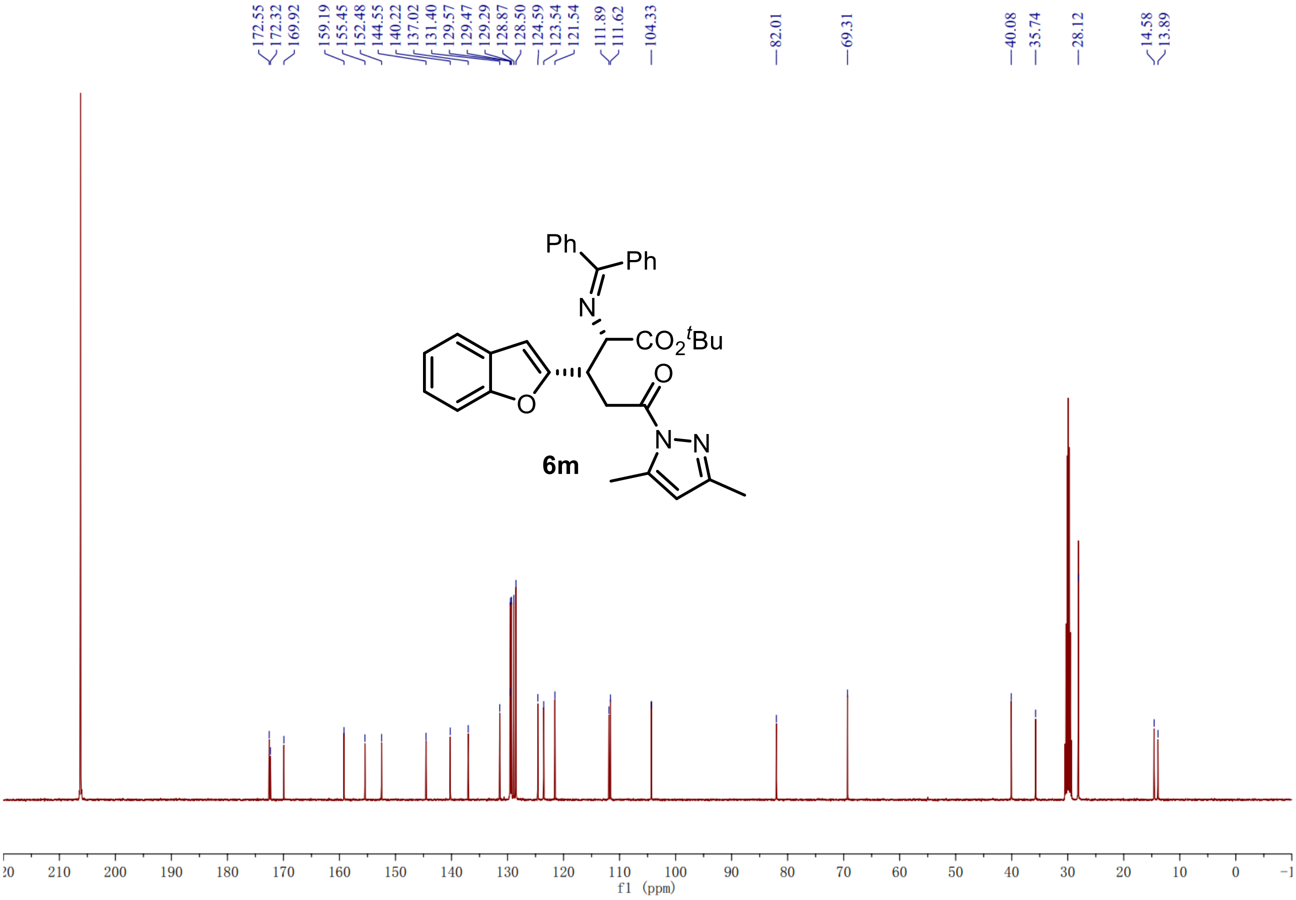
172.55
172.32
169.92
159.19
155.45
152.48
144.55
140.22
137.02
131.40
129.57
129.47
129.29
128.87
128.50
124.59
123.54
121.54
111.89
111.62
104.33
82.01
69.31
40.08
35.74
28.12
14.58
13.89
Ph
Ph
N
CO2tBu
O
O
N
N
6m
f1 (ppm)

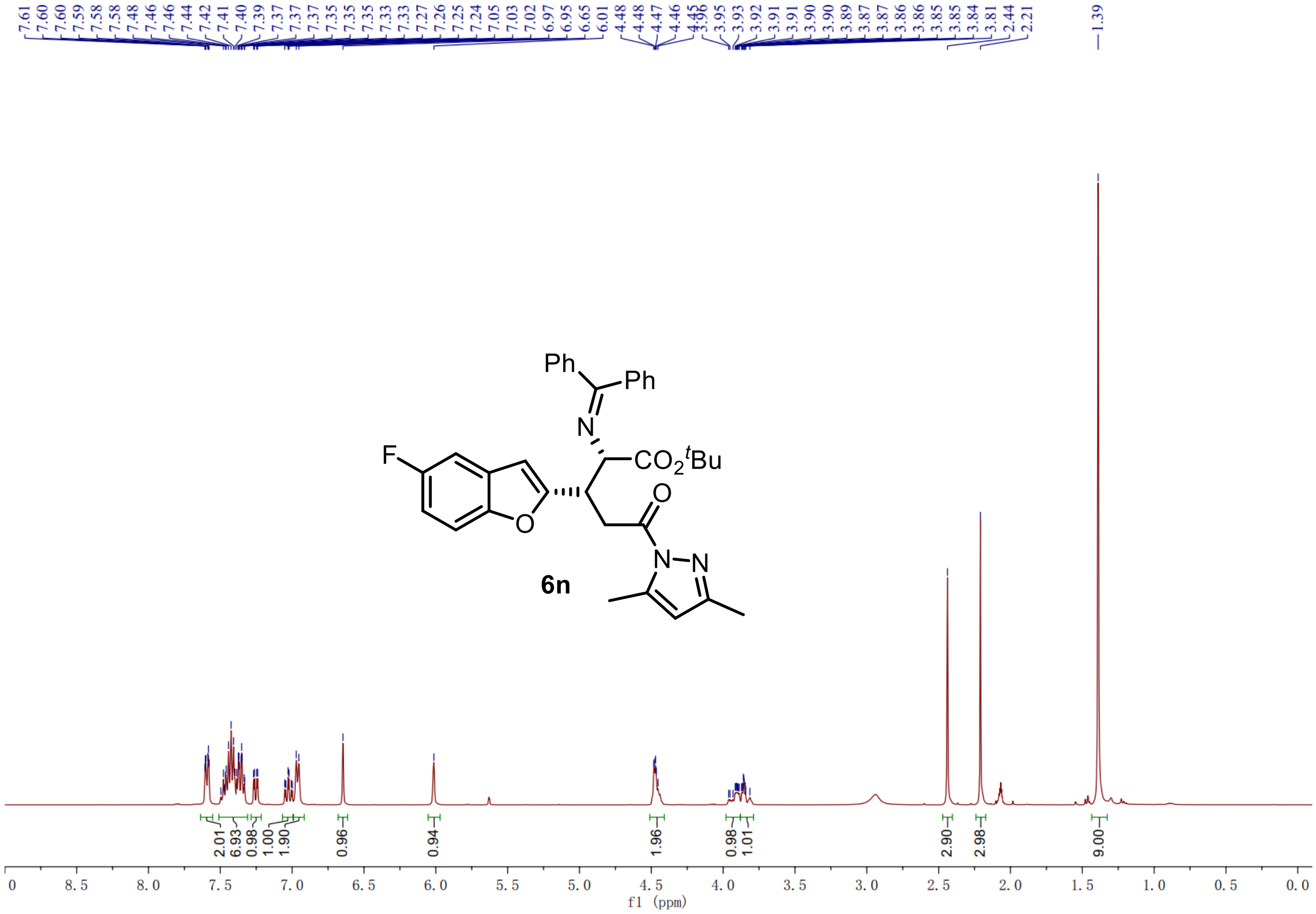
7.61
7.60
7.60
7.59
7.58
7.58
7.48
7.46
7.46
7.44
7.42
7.41
7.40
7.39
7.37
7.37
7.37
7.35
7.35
7.35
7.33
7.33
7.27
7.26
7.25
7.24
7.05
7.03
7.02
6.97
6.95
6.65
6.01
4.48
4.48
4.47
4.46
4.45
3.96
3.95
3.93
3.92
3.91
3.91
3.90
3.89
3.87
3.87
3.86
3.86
3.85
3.85
3.84
3.81
2.44
2.21
1.39
Ph
Ph
N
CO2tBu
F
O
O
N
N
6n
2.01
6.93
0.98
1.00
1.90
0.96
0.94
1.96
0.98
1.01
2.90
2.98
9.00
f1 (ppm)

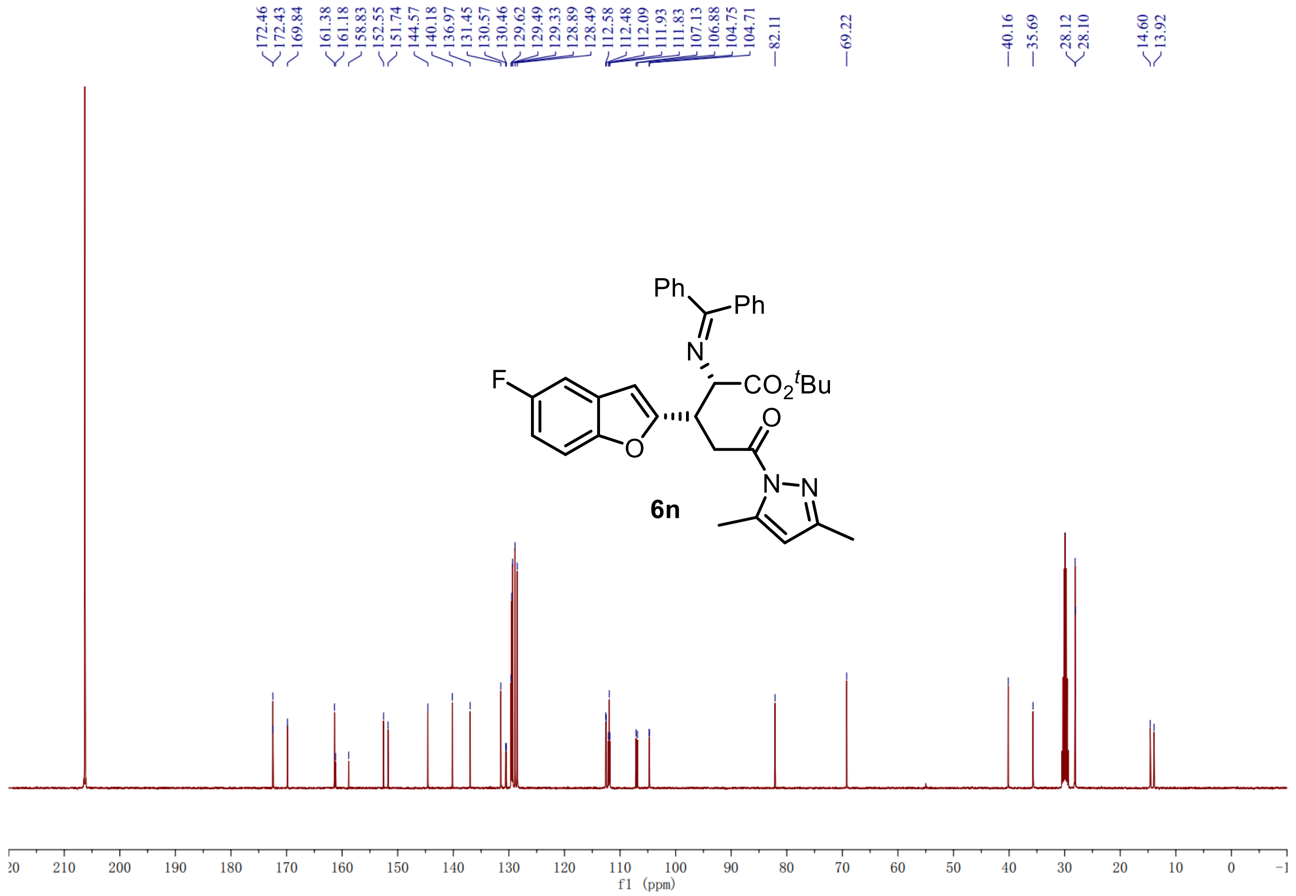
172.46
172.43
169.84
161.38
161.18
158.83
152.55
151.74
144.57
140.18
136.97
131.45
130.57
130.46
129.62
129.49
129.33
128.89
128.49
112.58
112.48
112.09
111.93
111.83
107.13
106.88
104.75
104.71
82.11
69.22
40.16
35.69
28.12
28.10
14.60
13.92
Ph
Ph
N
F
CO2tBu
O
O
N
N
6n
f1 (ppm)

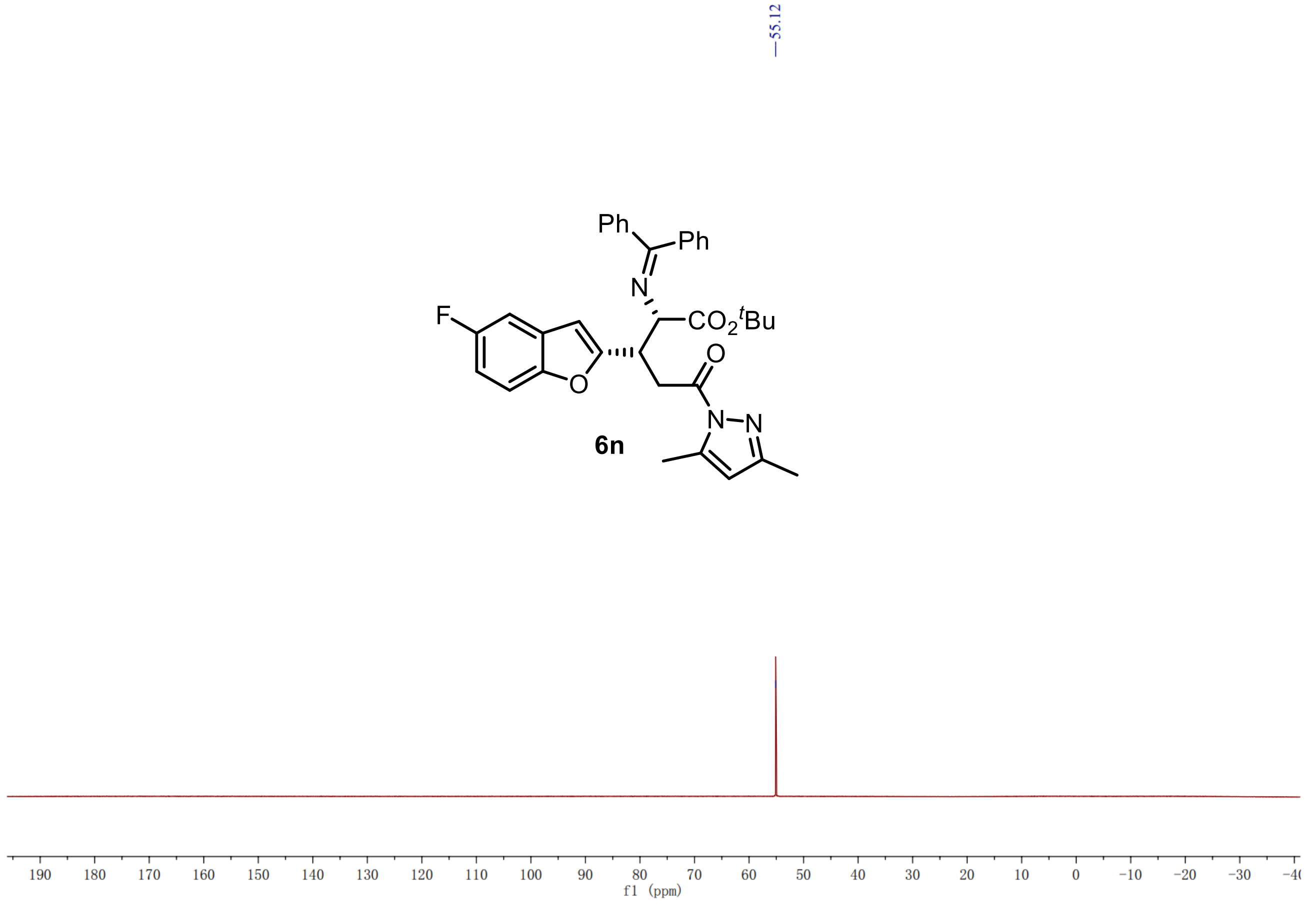
55.12
Ph
Ph
N
F
CO2tBu
O
O
N
N
6n
f1 (ppm)

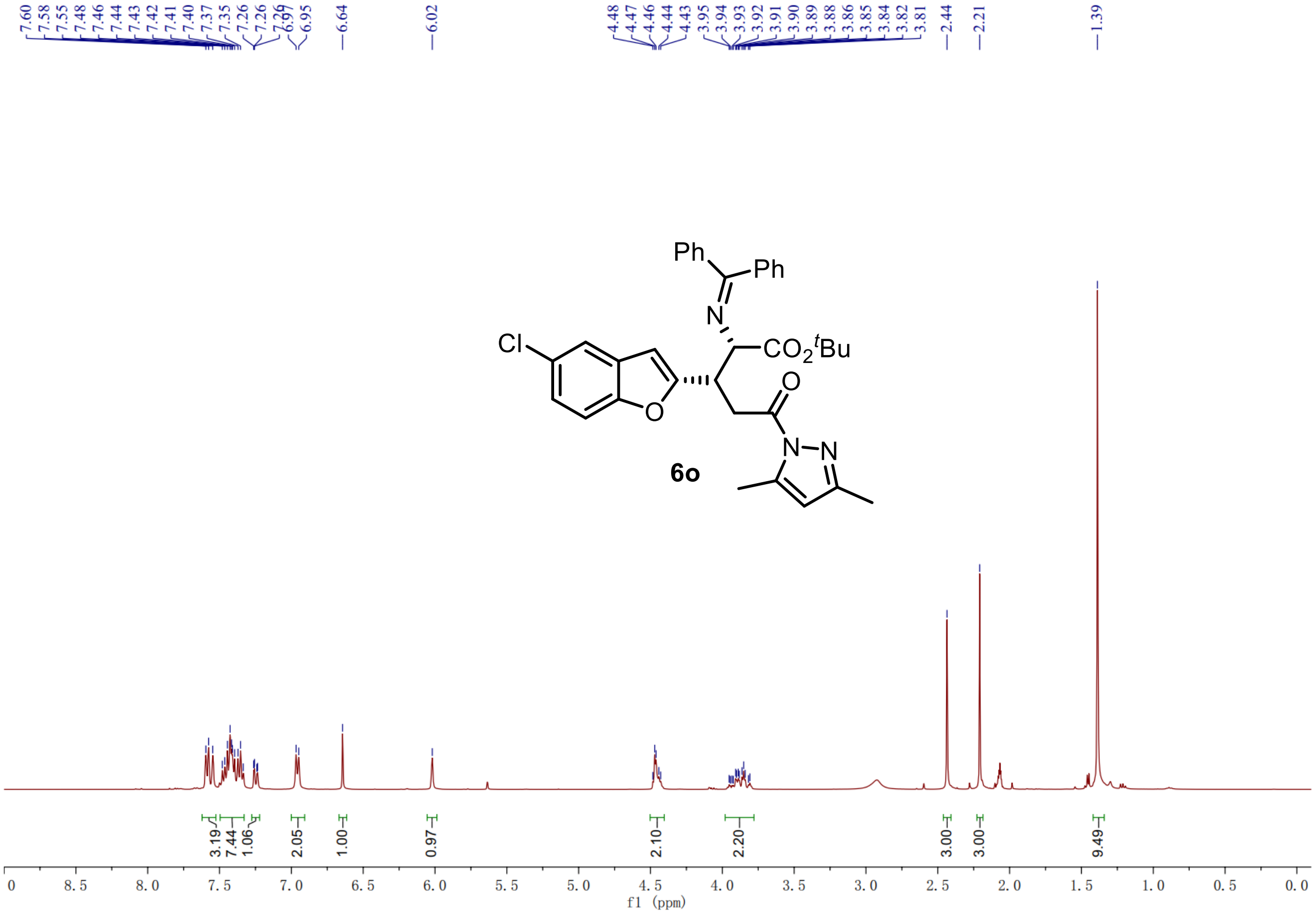
7.60
7.58
7.55
7.48
7.46
7.44
7.43
7.42
7.41
7.40
7.37
7.35
7.26
7.26
6.97
6.95
6.64
6.02
4.48
4.47
4.46
4.44
4.43
3.95
3.94
3.93
3.92
3.91
3.90
3.89
3.88
3.86
3.85
3.84
3.82
3.81
2.44
2.21
1.39
Ph
Ph
N
CO2tBu
Cl
O
O
N
N
3.19
7.44
1.06
2.05
1.00
0.97
2.10
2.20
3.00
3.00
9.49
f1 (ppm)

6o

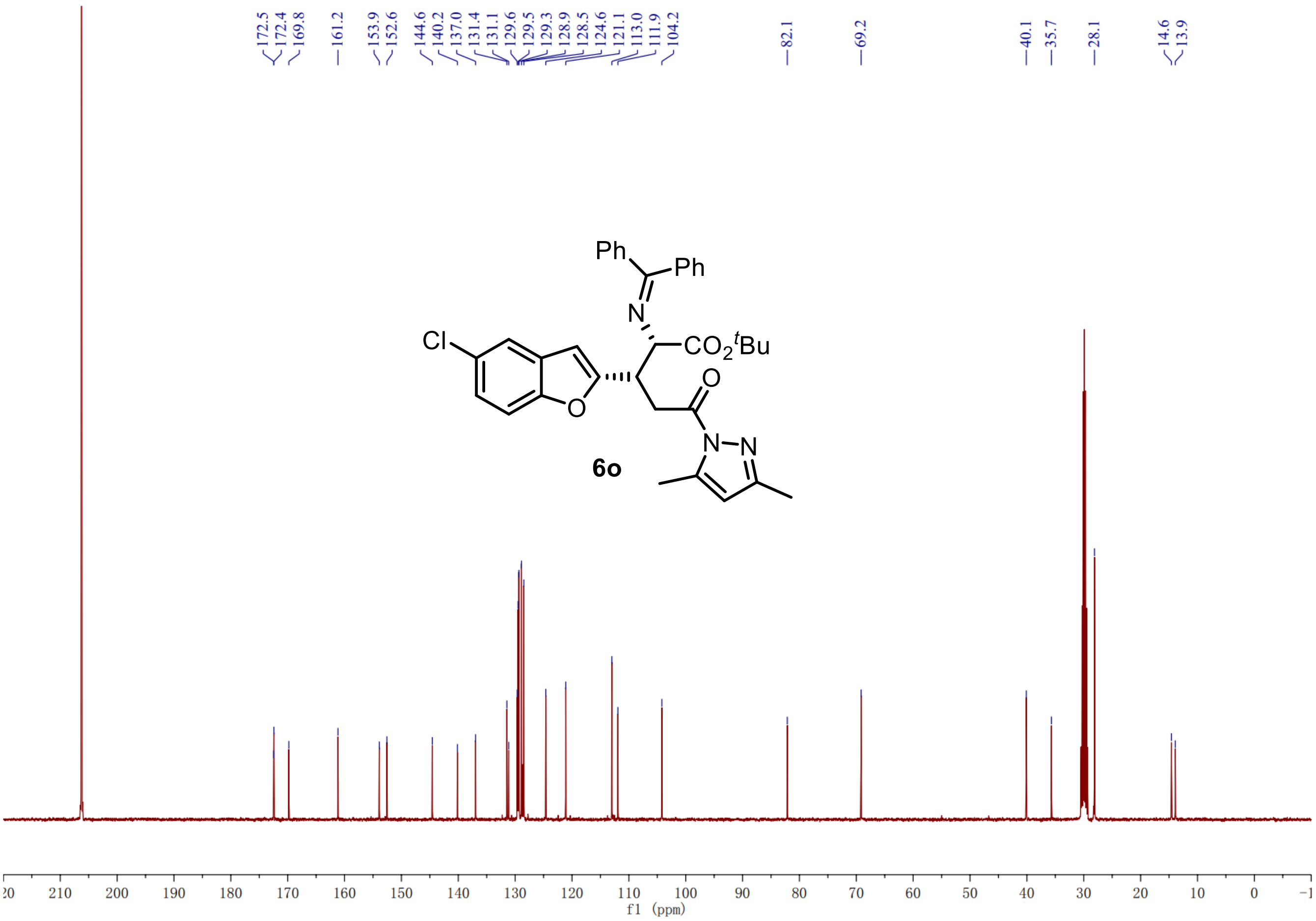
172.5
172.4
169.8
161.2
153.9
152.6
144.6
140.2
137.0
131.4
131.1
129.6
129.5
129.3
128.9
128.5
124.6
121.1
113.0
111.9
104.2
82.1
69.2
40.1
35.7
28.1
14.6
13.9
Ph
Ph
N
CO2tBu
Cl
O
O
N
N
f1 (ppm)

6o

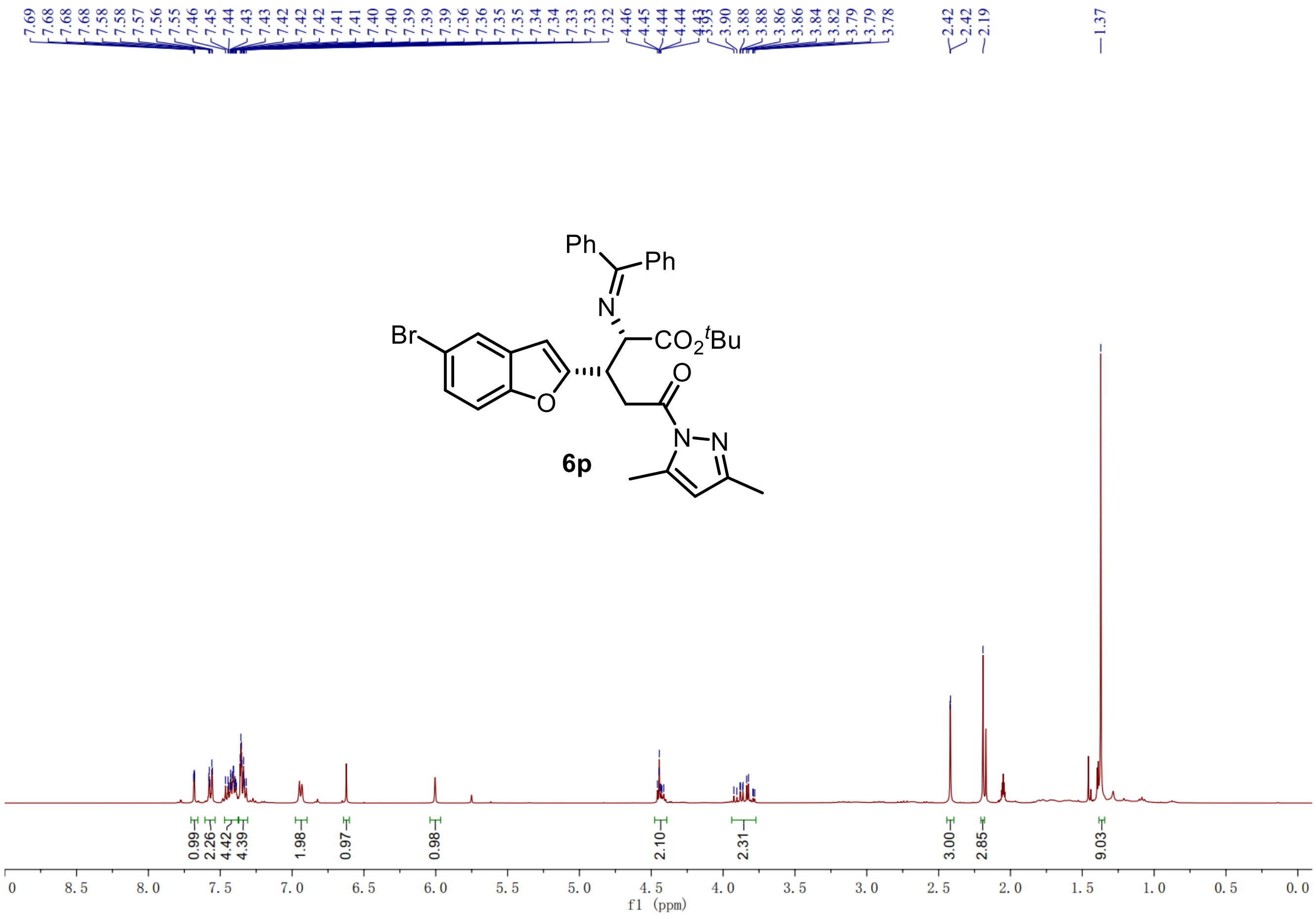

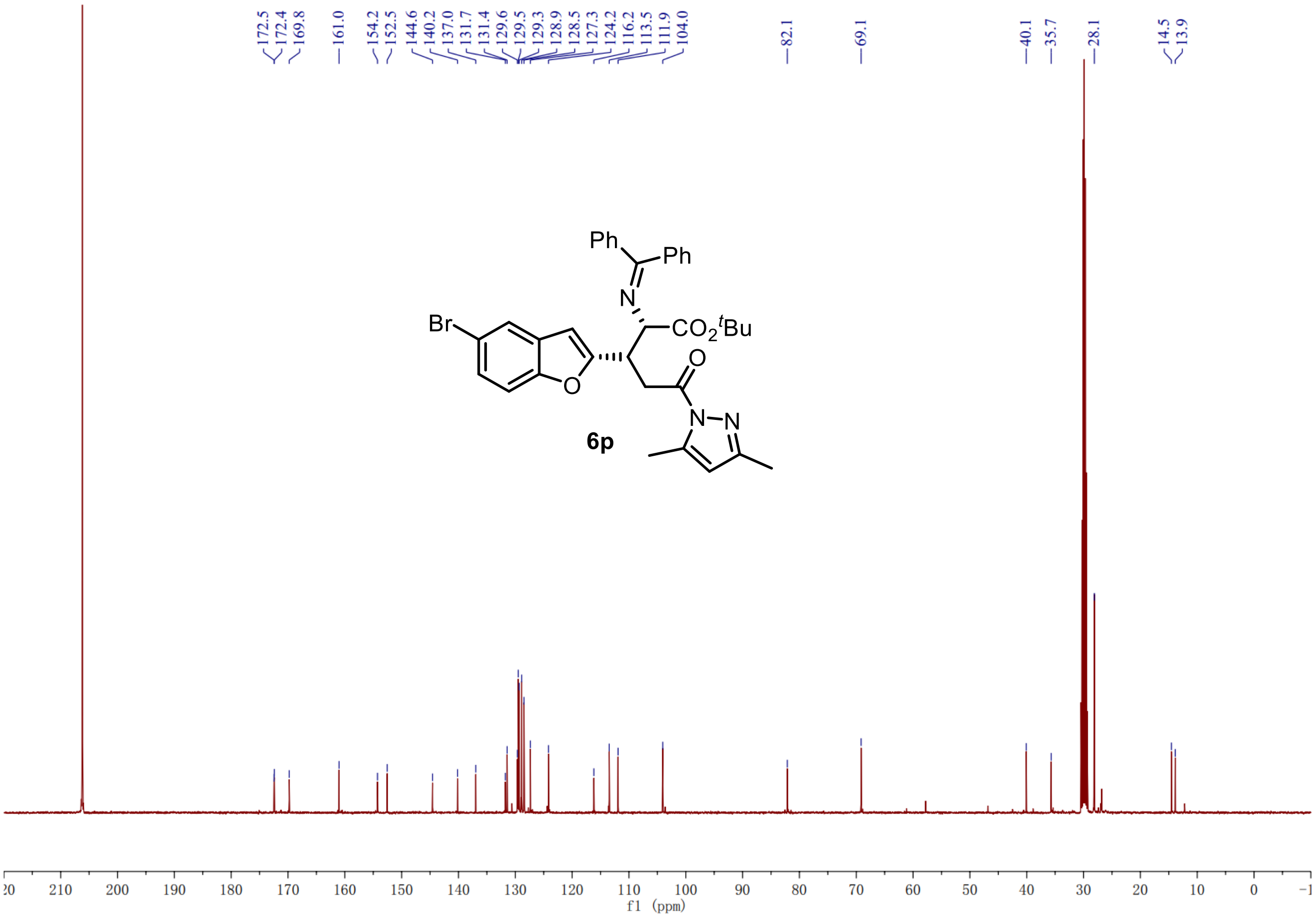

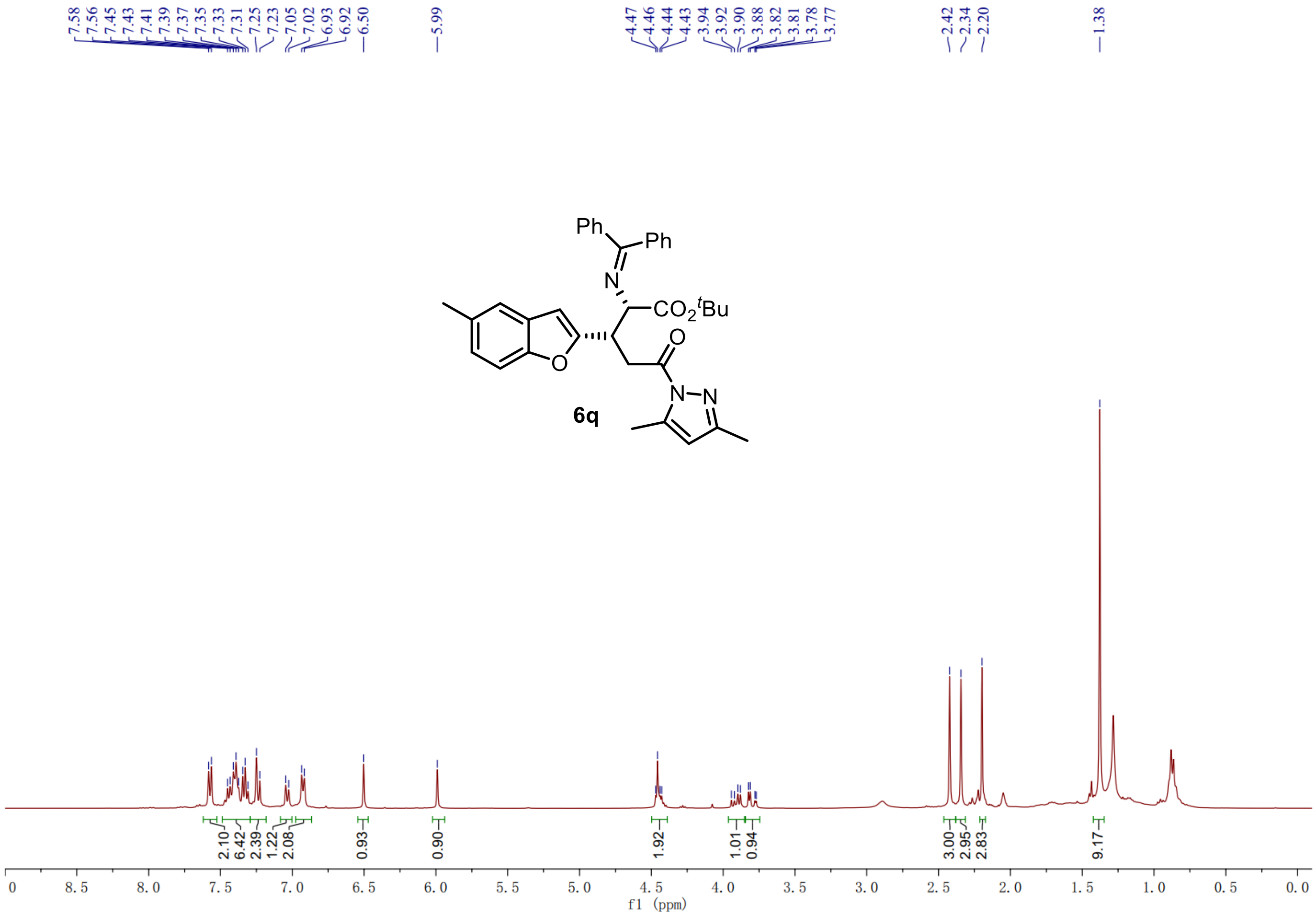

7.58
7.56
7.45
7.43
7.41
7.39
7.37
7.35
7.33
7.31
7.25
7.23
7.05
7.02
6.93
6.92
6.50
5.99
4.47
4.46
4.44
4.43
3.94
3.92
3.90
3.88
3.82
3.81
3.78
3.77
2.42
2.34
2.20
1.38
Ph
Ph
N
CO2tBu
O
O
N
N
6q
2.10
6.42
2.39
1.22
2.08
0.93
0.90
1.92
1.01
0.94
3.00
2.95
2.83
9.17
f1 (ppm)

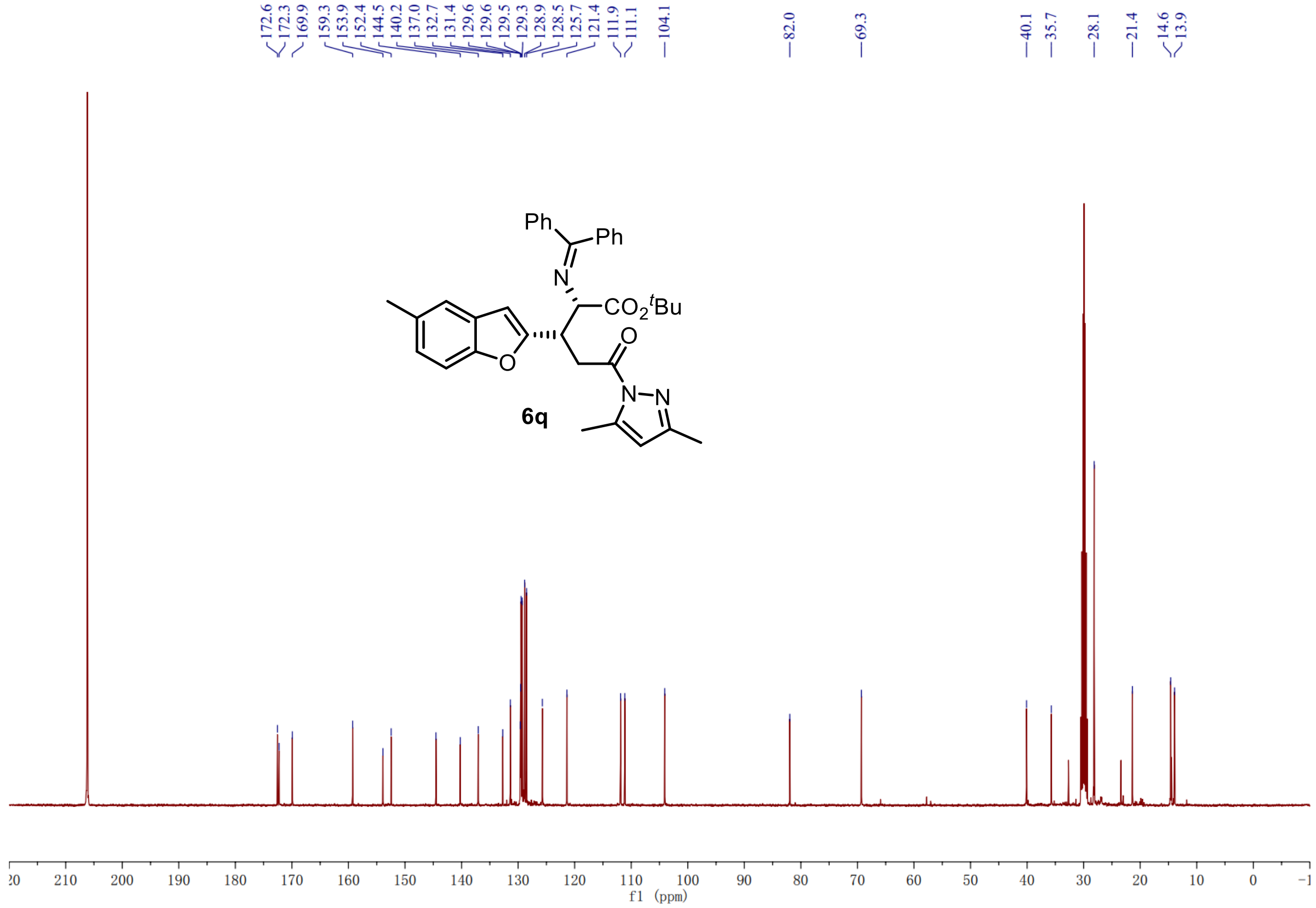

172.6
172.3
169.9
159.3
153.9
152.4
144.5
140.2
137.0
132.7
131.4
129.6
129.6
129.5
129.3
128.9
128.5
125.7
121.4
111.9
111.1
104.1
82.0
69.3
40.1
35.7
28.1
21.4
14.6
13.9
Ph
Ph
N
CO2tBu
O
O
N
N
6q
f1 (ppm)

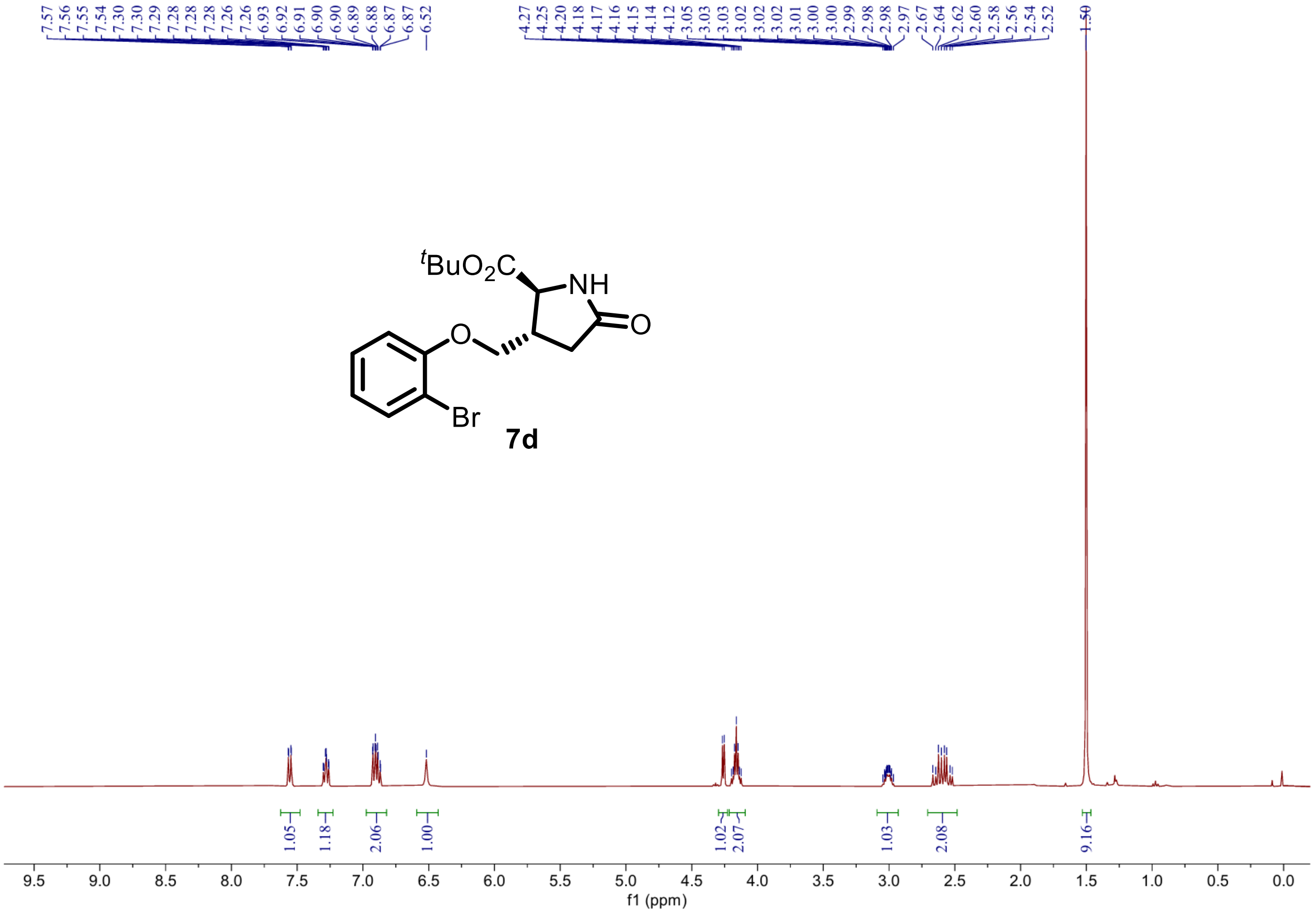
7.57 7.56 7.55 7.54 7.30 7.29 7.28 7.28 7.28 7.26 7.26 6.93 6.92 6.91 6.90 6.90 6.89 6.88 6.87 6.87 6.52
4.27 4.25 4.20 4.18 4.17 4.16 4.15 4.14 4.12 3.05 3.03 3.03 3.02 3.02 3.02 3.01 3.00 3.00 2.99 2.98 2.98 2.97 2.67 2.64 2.62 2.60 2.58 2.56 2.54 2.52
1.50
^{t}BuO$_2$C
NH
O
O
Br
7d
1.05 1.18 2.06 1.00 1.02 2.07 1.03 2.08 9.16
9.5 9.0 8.5 8.0 7.5 7.0 6.5 6.0 5.5 5.0 4.5 4.0 3.5 3.0 2.5 2.0 1.5 1.0 0.5 0.0
f1 (ppm)

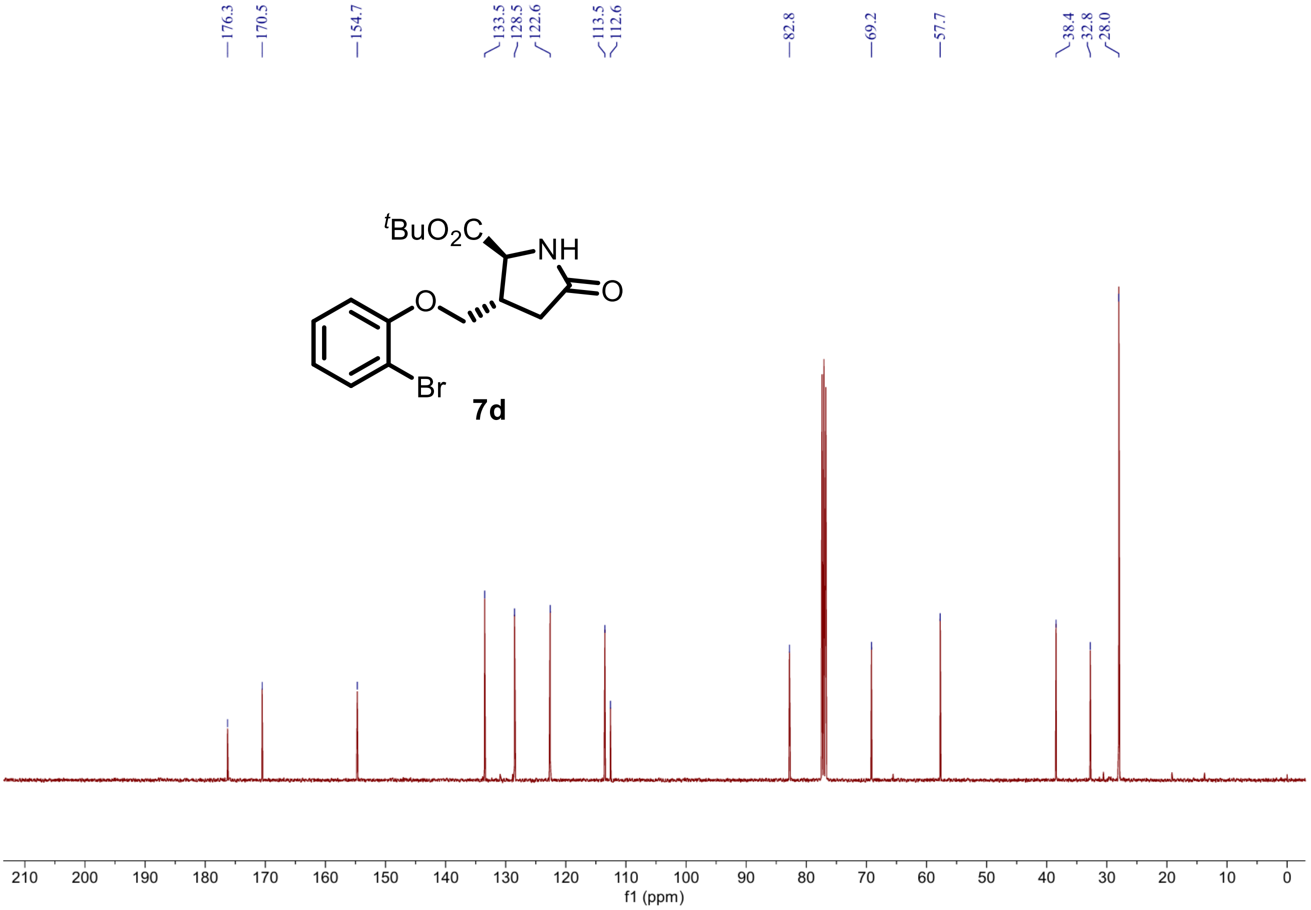
176.3
170.5
154.7
133.5 128.5 122.6
113.5 112.6
82.8
69.2
57.7
38.4 32.8 28.0
^{t}BuO$_2$C
NH
O
O
Br
7d
210 200 190 180 170 160 150 140 130 120 110 100 90 80 70 60 50 40 30 20 10 0
f1 (ppm)

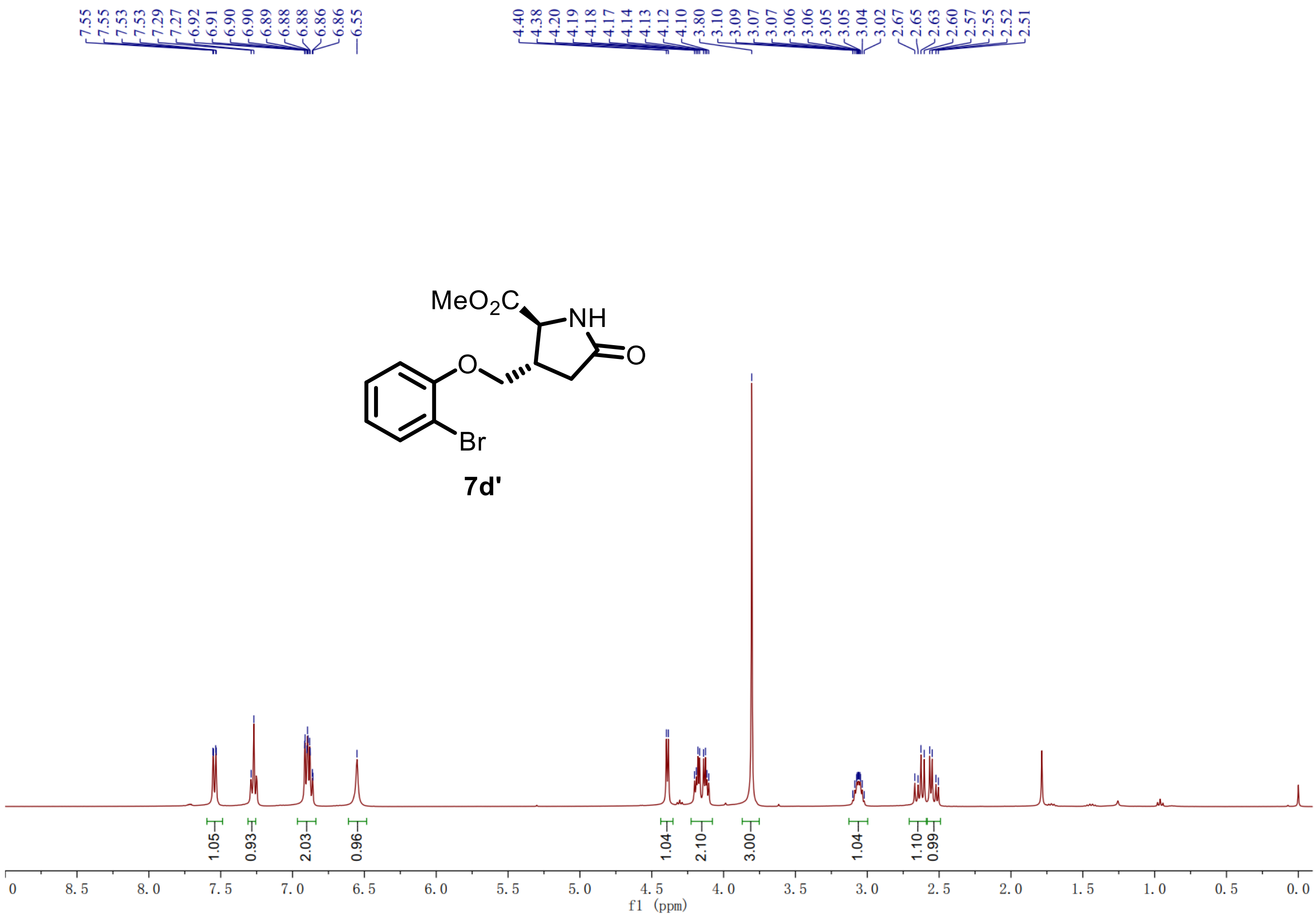

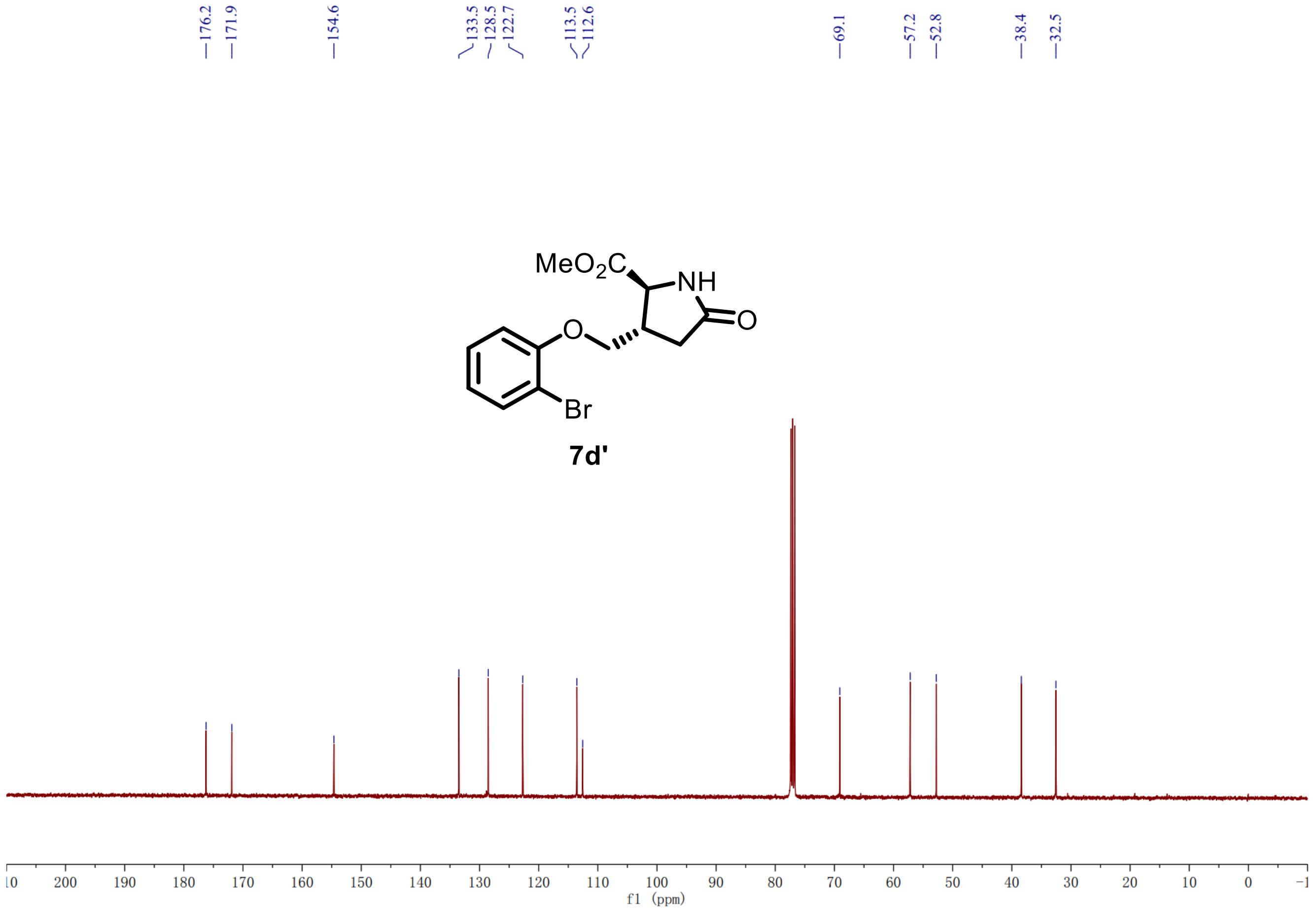

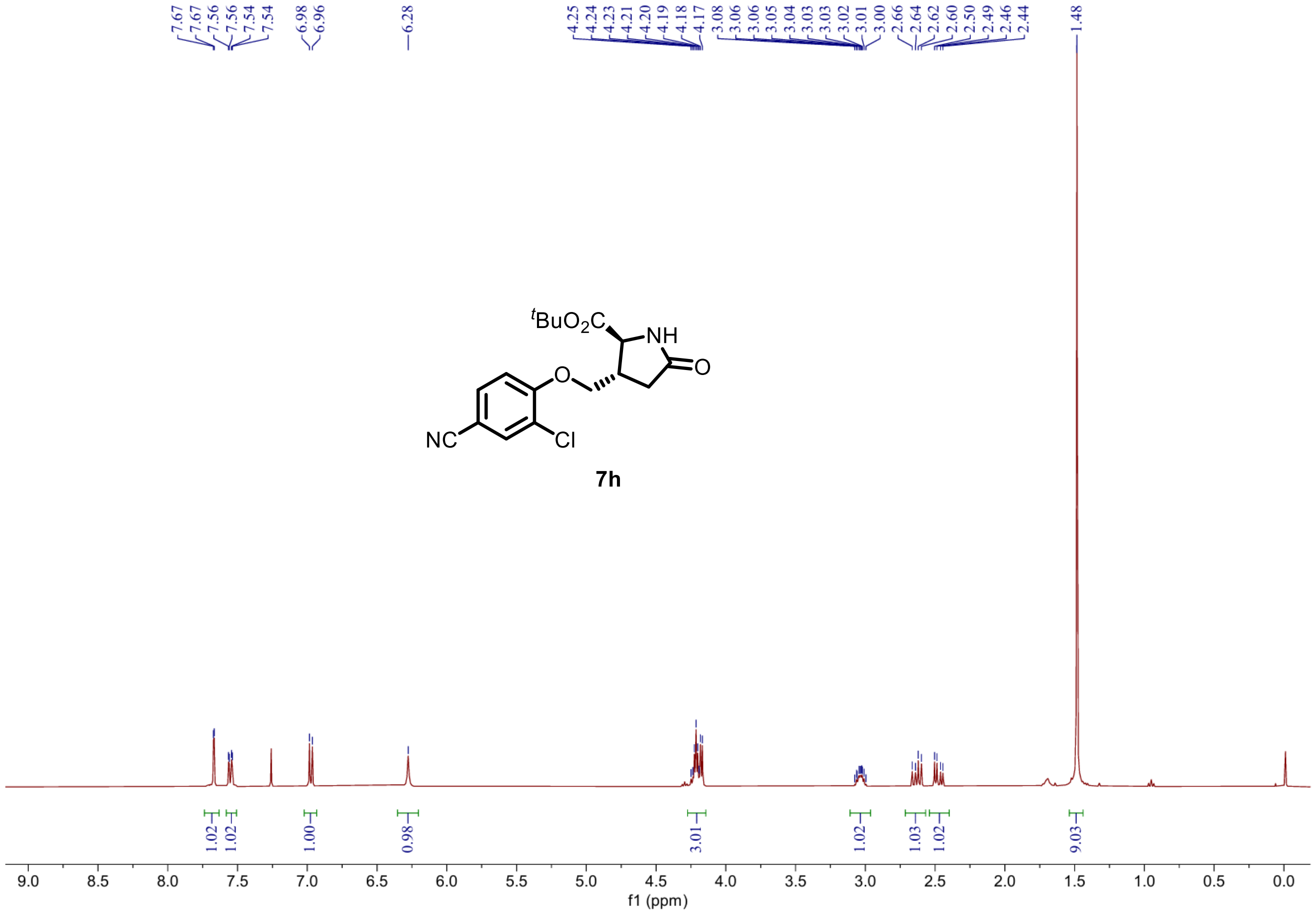
7.67
7.67
7.56
7.56
7.54
7.54
6.98
6.96
6.28
4.25
4.24
4.23
4.21
4.20
4.19
4.18
4.17
3.08
3.06
3.05
3.04
3.03
3.03
3.02
3.01
3.00
2.66
2.64
2.62
2.60
2.50
2.49
2.46
2.44
1.48
^{t}BuO$_2$C
NH
O
O
NC
Cl
7h
1.02
1.02
1.00
0.98
3.01
1.02
1.03
1.02
9.03
f1 (ppm)

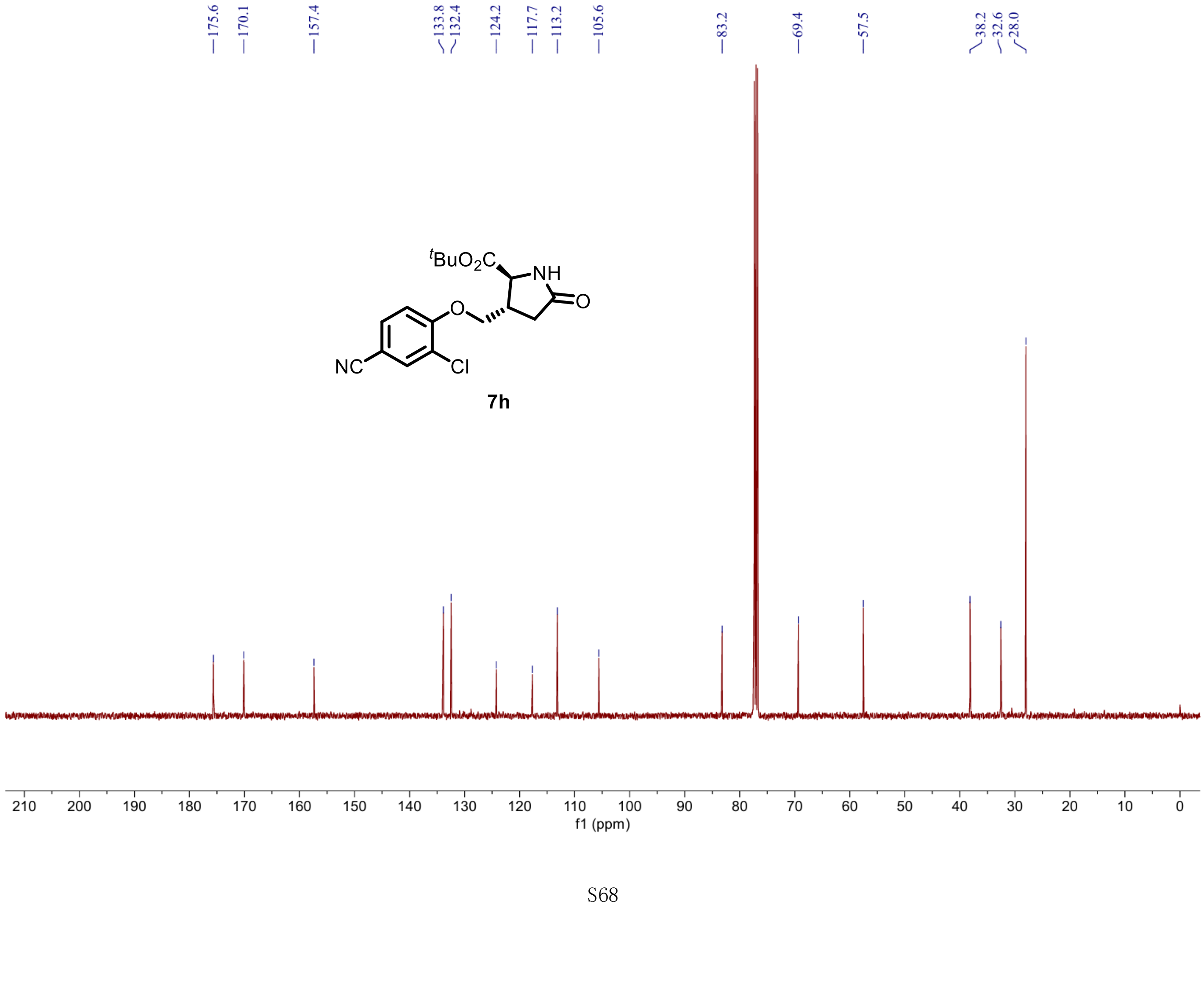
175.6
170.1
157.4
133.8
132.4
124.2
117.7
113.2
105.6
83.2
69.4
57.5
38.2
32.6
28.0
^{t}BuO$_2$C
NH
O
O
NC
Cl
7h
f1 (ppm)

5. HRMS scanning copies

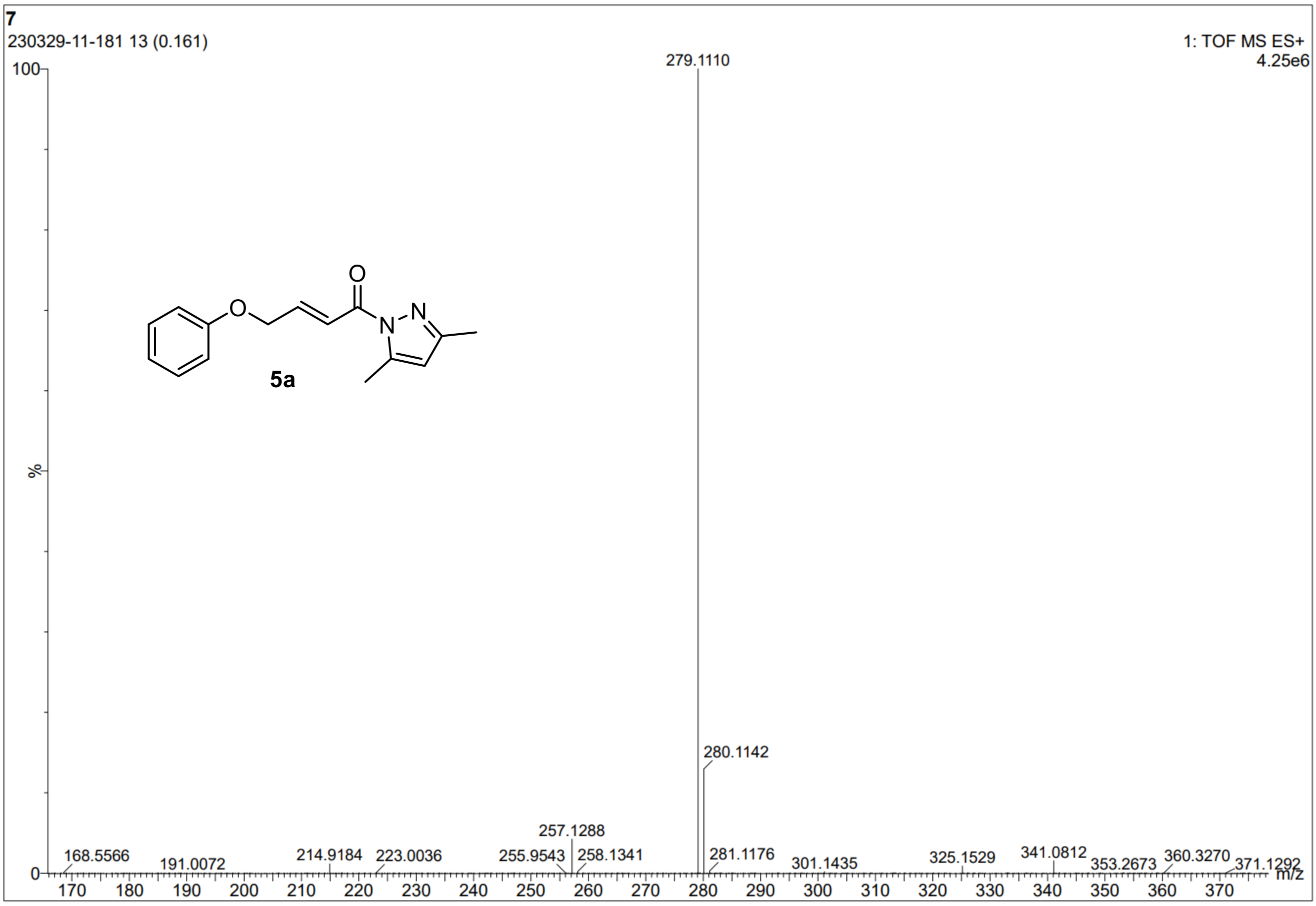

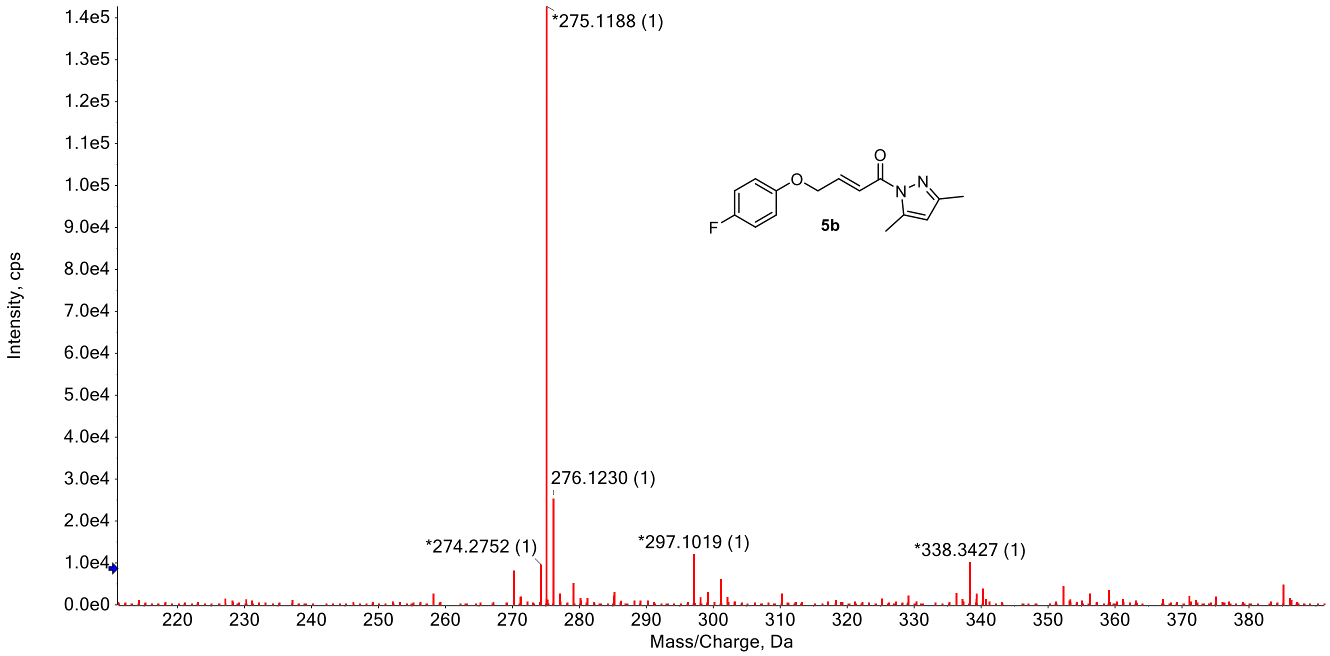

Spectrum from WPA-2024.wiff2 (sample 18) - 366, +TOF MS (50 - 2000) from 0.287 min

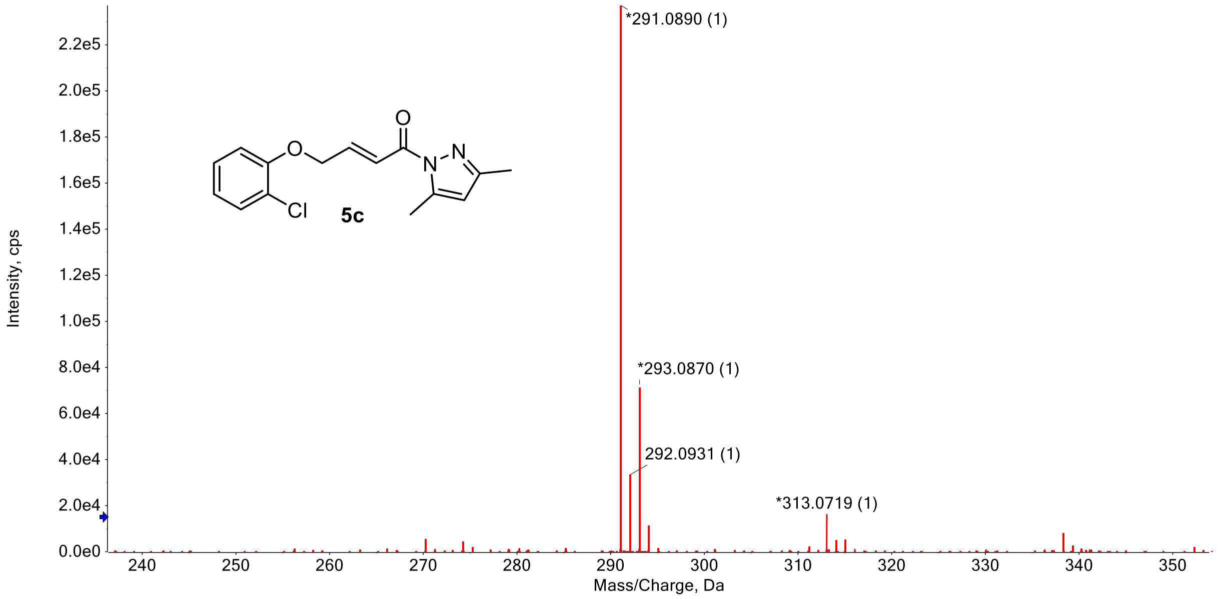

Spectrum from WPA-2024.wiff2 (sample 7) - 356, +TOF MS (50 - 2000) from 0.245 min

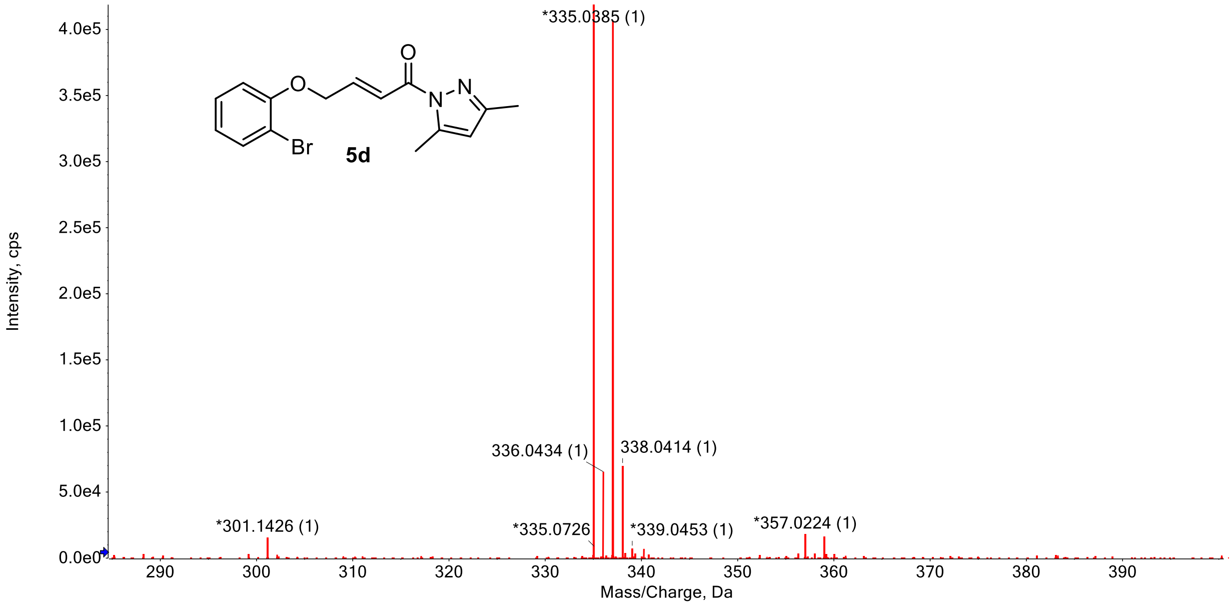

Spectrum from WPA-2024.wiff2 (sample 5) - 354, +TOF MS (50 - 2000) from 0.310 min

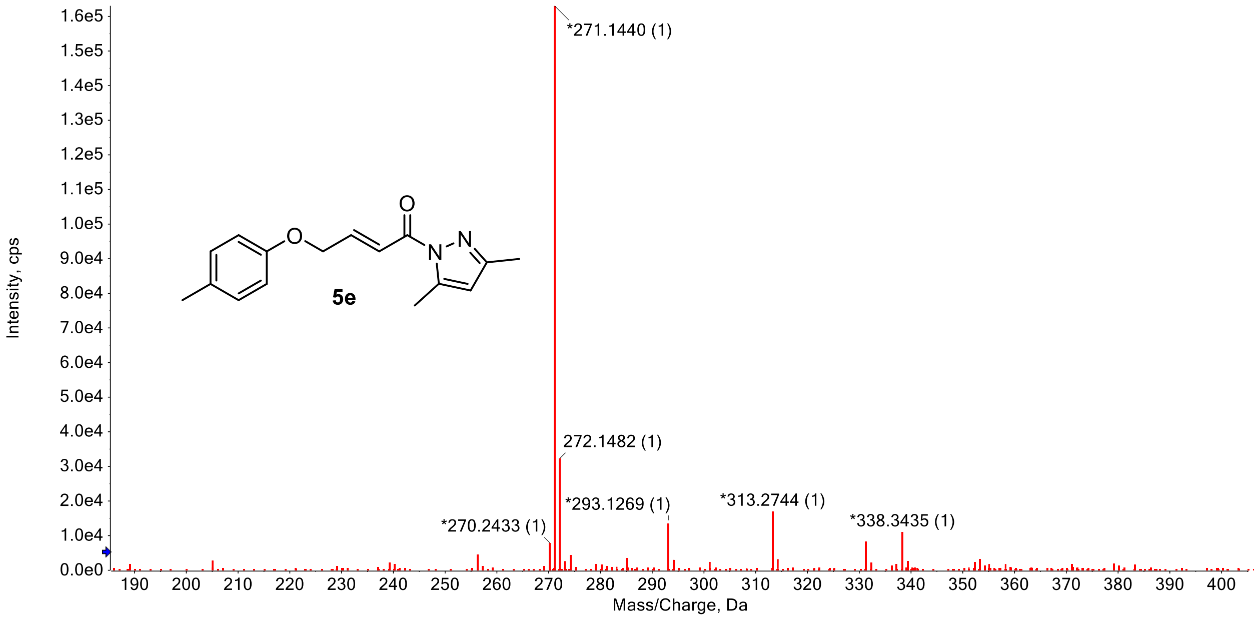

O

Spectrum from WPA-2024.wiff2 (sample 9) - 358, +TOF MS (50 - 2000) from 0.301 min

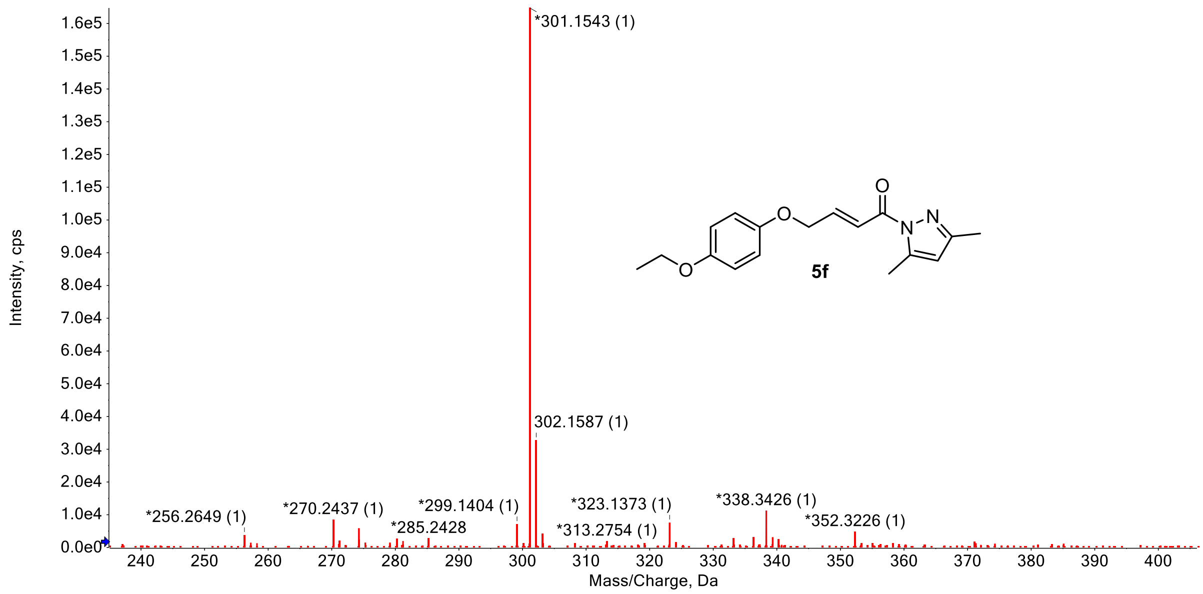

Spectrum from WPA-2024.wiff2 (sample 25) - 373, +TOF MS (50 - 2000) from 0.273 min

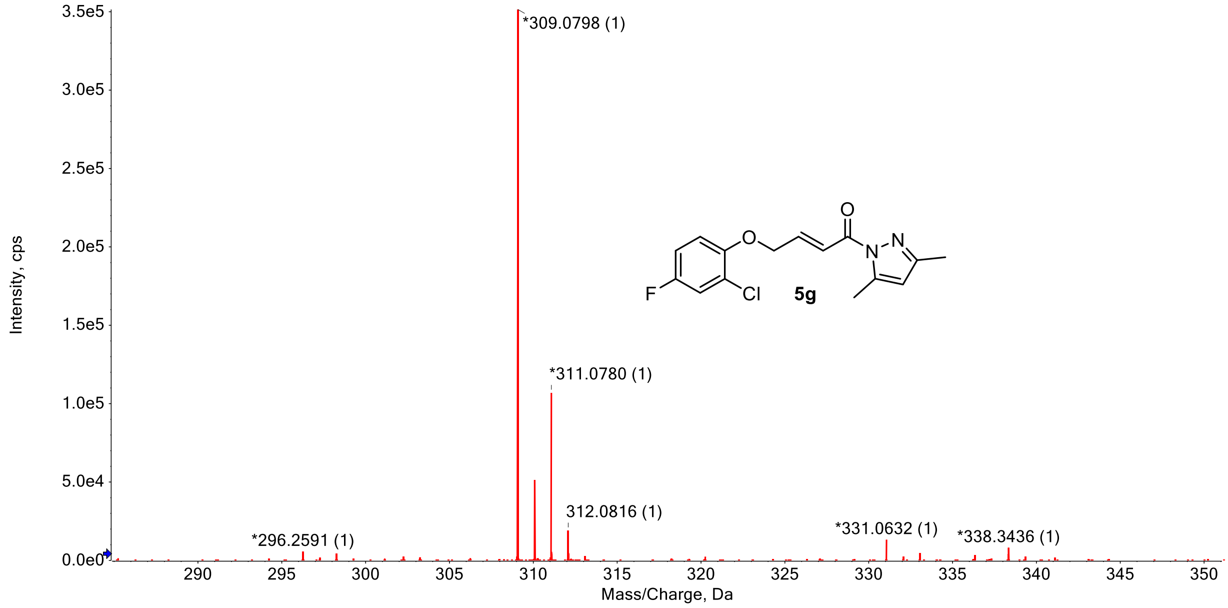

Spectrum from WPA-2024.wiff2 (sample 13) - 362, +TOF MS (50 - 2000) from 0.310 min

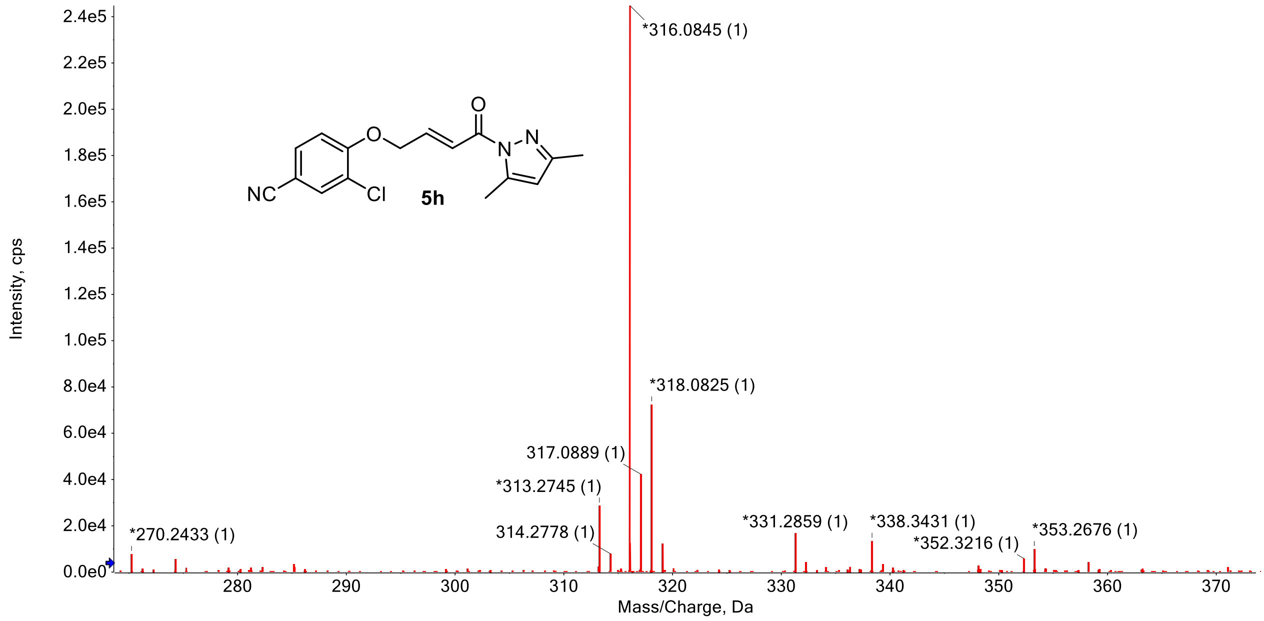

Spectrum from WPA-2024.wiff2 (sample 20) - 368, +TOF MS (50 - 2000) from 0.329 min

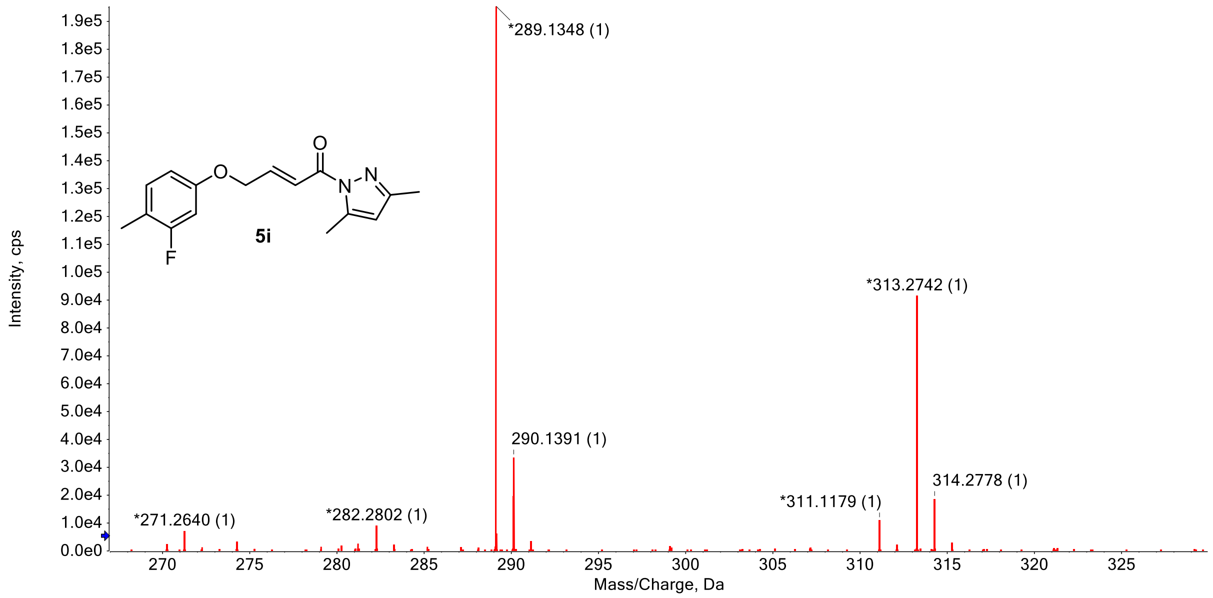

Spectrum from WPA-2024.wiff2 (sample 11) - 360, +TOF MS (50 - 2000) from 0.422 min

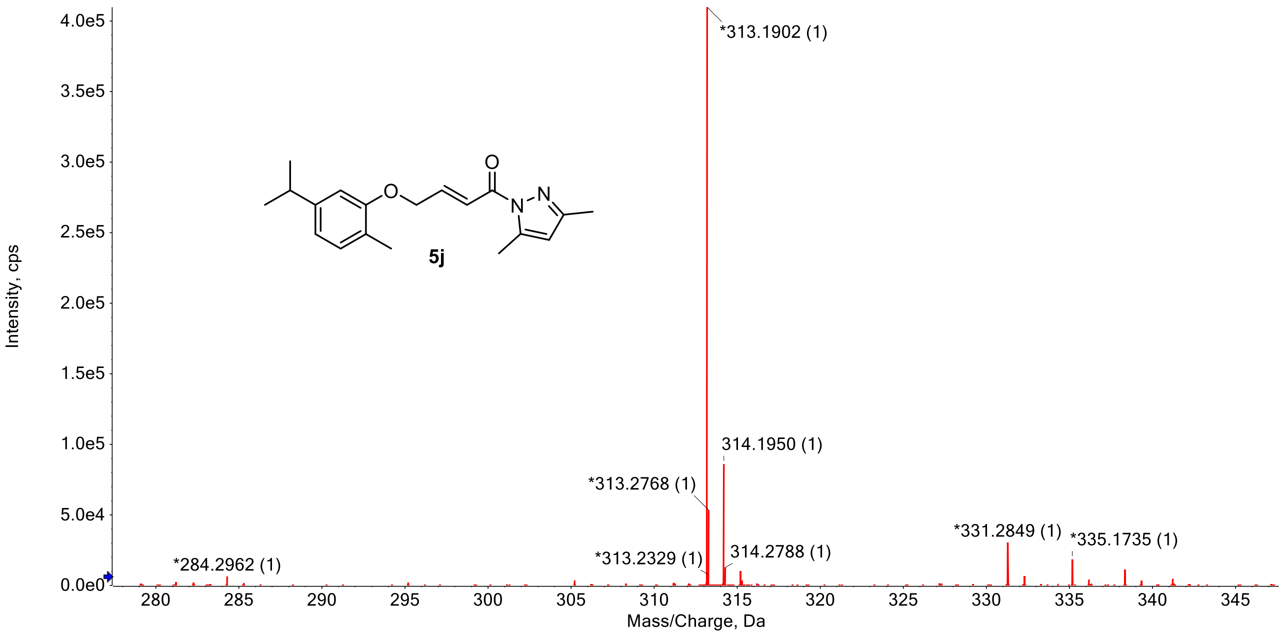

Spectrum from WPA-2024.wiff2 (sample 3) - 352, +TOF MS (50 - 2000) from 0.236 min

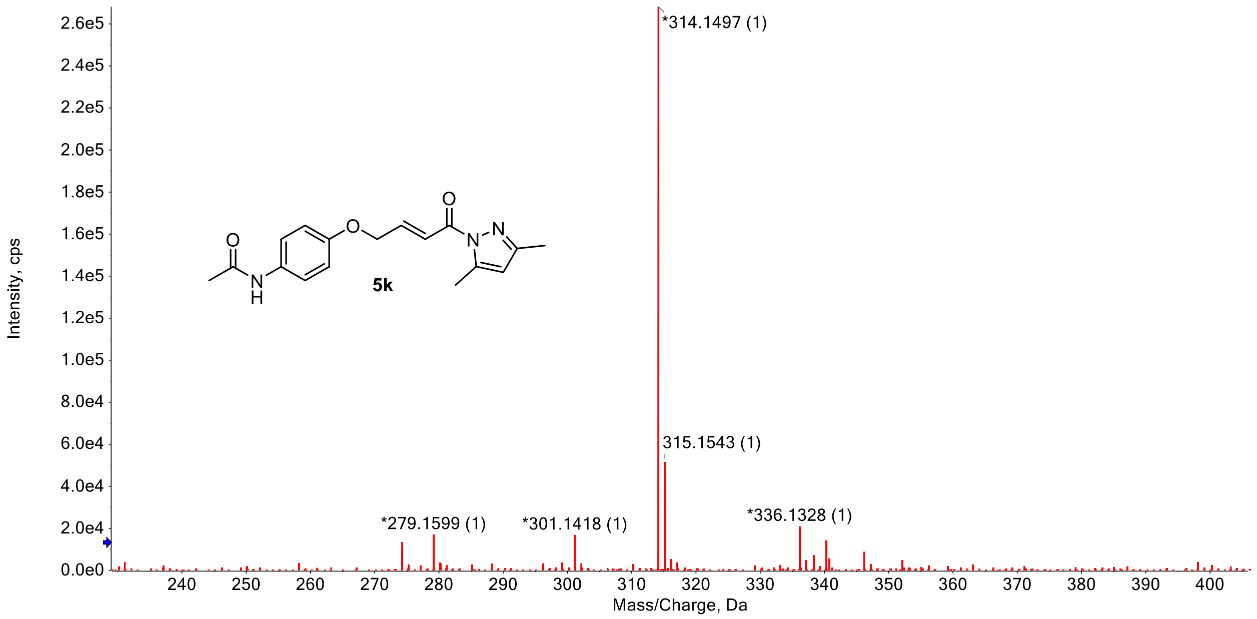

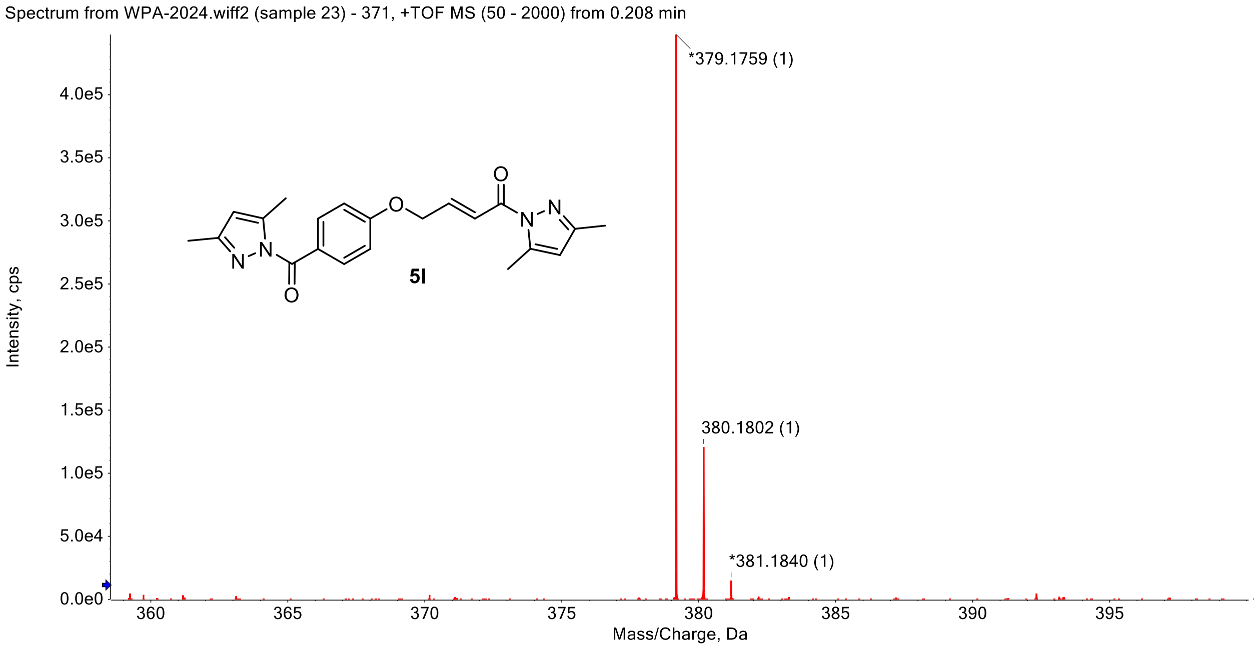

Spectrum from WPA-2024.wiff2 (sample 23) - 371, +TOF MS (50 - 2000) from 0.208 min
*379.1759 (1)
380.1802 (1)
*381.1840 (1)
Intensity, cps
Mass/Charge, Da
5l

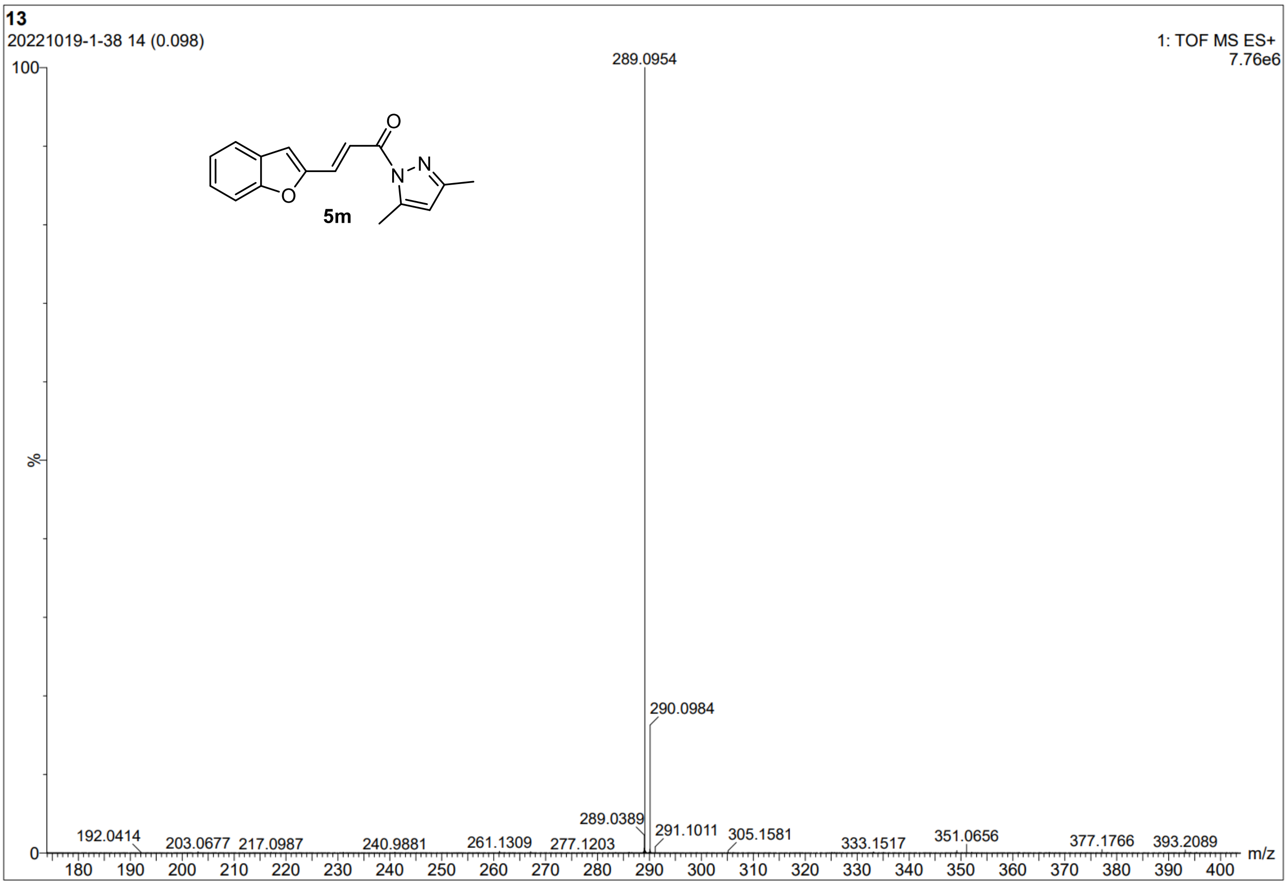

13
20221019-1-38 14 (0.098)
1: TOF MS ES+
7.76e6
289.0954
290.0984
289.0389
291.1011
192.0414
203.0677
217.0987
240.9881
261.1309
277.1203
305.1581
333.1517
351.0656
377.1766
393.2089
m/z
%
5m

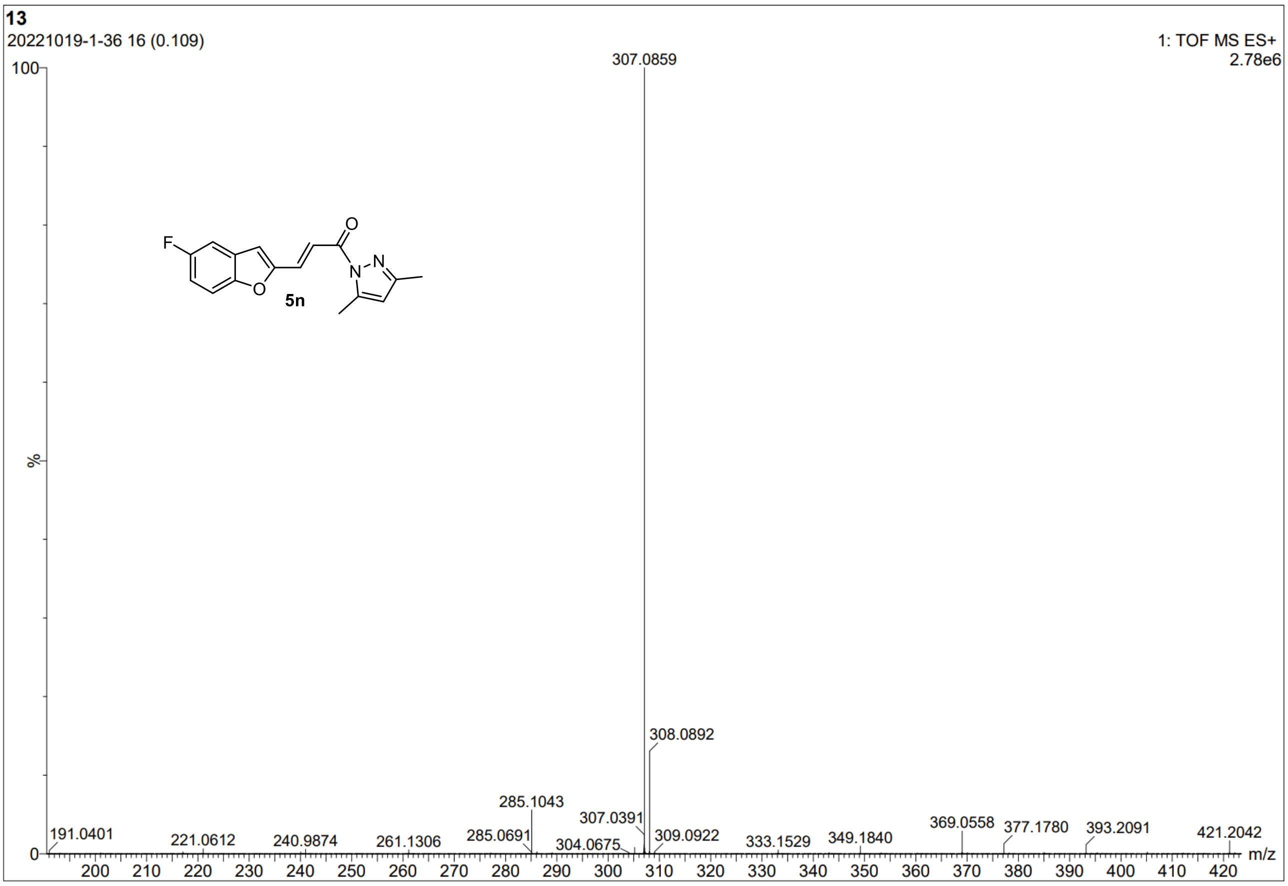
13
20221019-1-36 16 (0.109)
1: TOF MS ES+
2.78e6
307.0859
308.0892
285.1043
307.0391
191.0401
221.0612
240.9874
261.1306
285.0691
304.0675
309.0922
333.1529
349.1840
369.0558
377.1780
393.2091
421.2042
%
m/z
F
O
N
N
5n

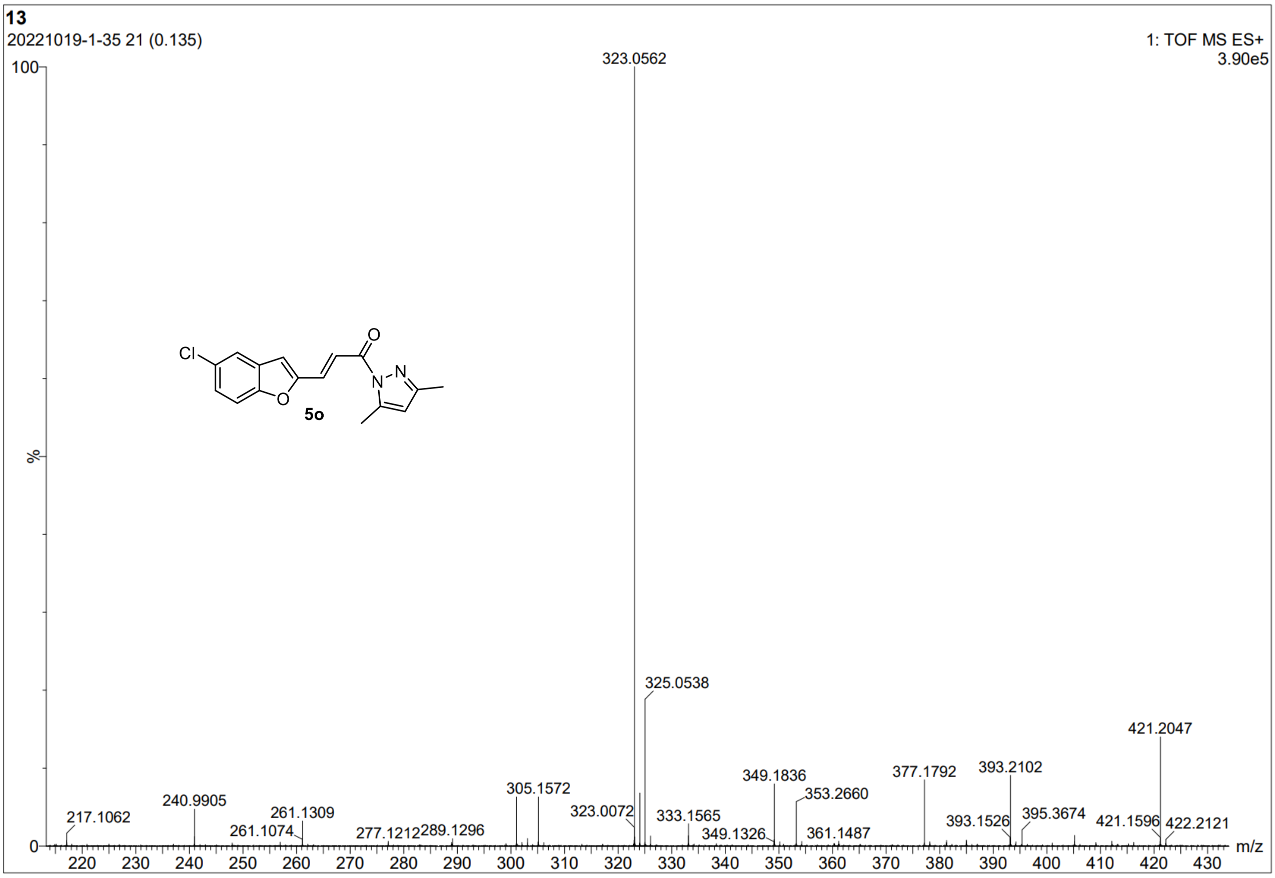
13
20221019-1-35 21 (0.135)
1: TOF MS ES+
3.90e5
323.0562
325.0538
421.2047
349.1836
377.1792
393.2102
353.2660
305.1572
240.9905
261.1309
217.1062
261.1074
277.1212
289.1296
323.0072
333.1565
349.1326
361.1487
393.1526
395.3674
421.1596
422.2121
%
m/z
Cl
O
N
N
5o

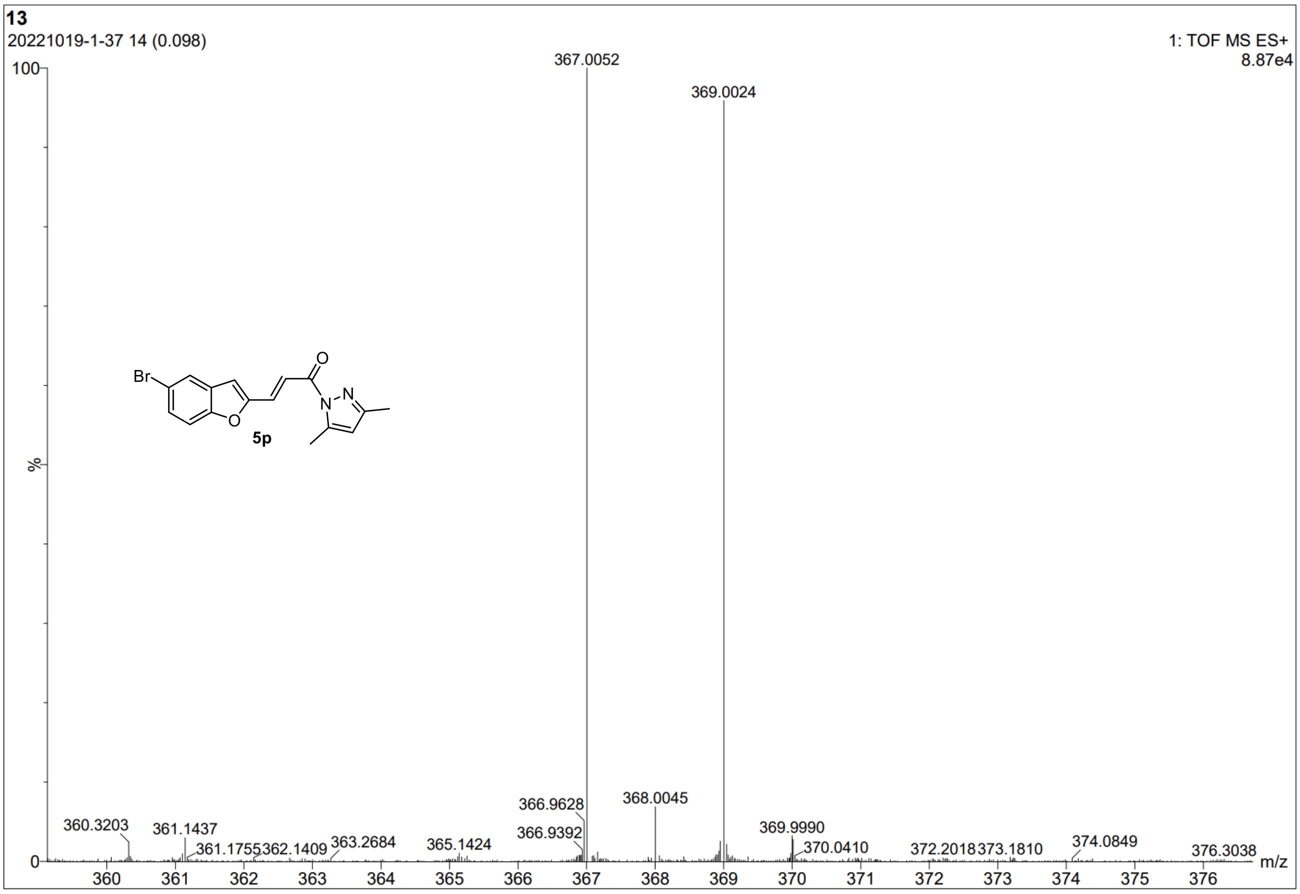

13
20221019-1-37 14 (0.098)
1: TOF MS ES+
8.87e4
100
367.0052
369.0024
Br
O
N
N
O
5p
%
366.9628
368.0045
360.3203
361.1437
366.9392
369.9990
361.1755
362.1409
363.2684
365.1424
370.0410
372.2018
373.1810
374.0849
376.3038
0
m/z
360
361
362
363
364
365
366
367
368
369
370
371
372
373
374
375
376

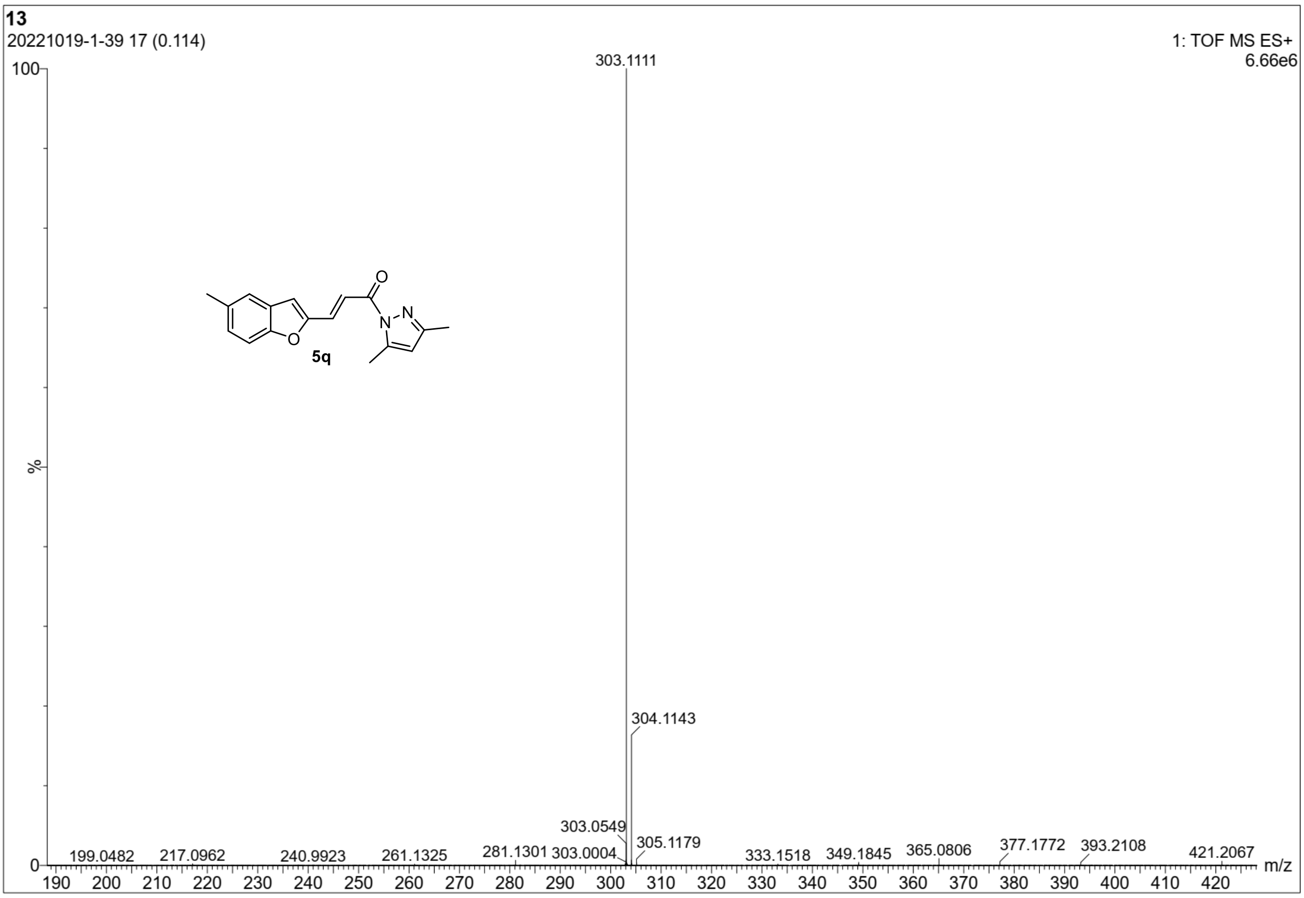

13
20221019-1-39 17 (0.114)
1: TOF MS ES+
6.66e6
100
303.1111
O
N
N
O
5q
%
304.1143
303.0549
305.1179
199.0482
217.0962
240.9923
261.1325
281.1301
303.0004
333.1518
349.1845
365.0806
377.1772
393.2108
421.2067
0
m/z
190
200
210
220
230
240
250
260
270
280
290
300
310
320
330
340
350
360
370
380
390
400
410
420

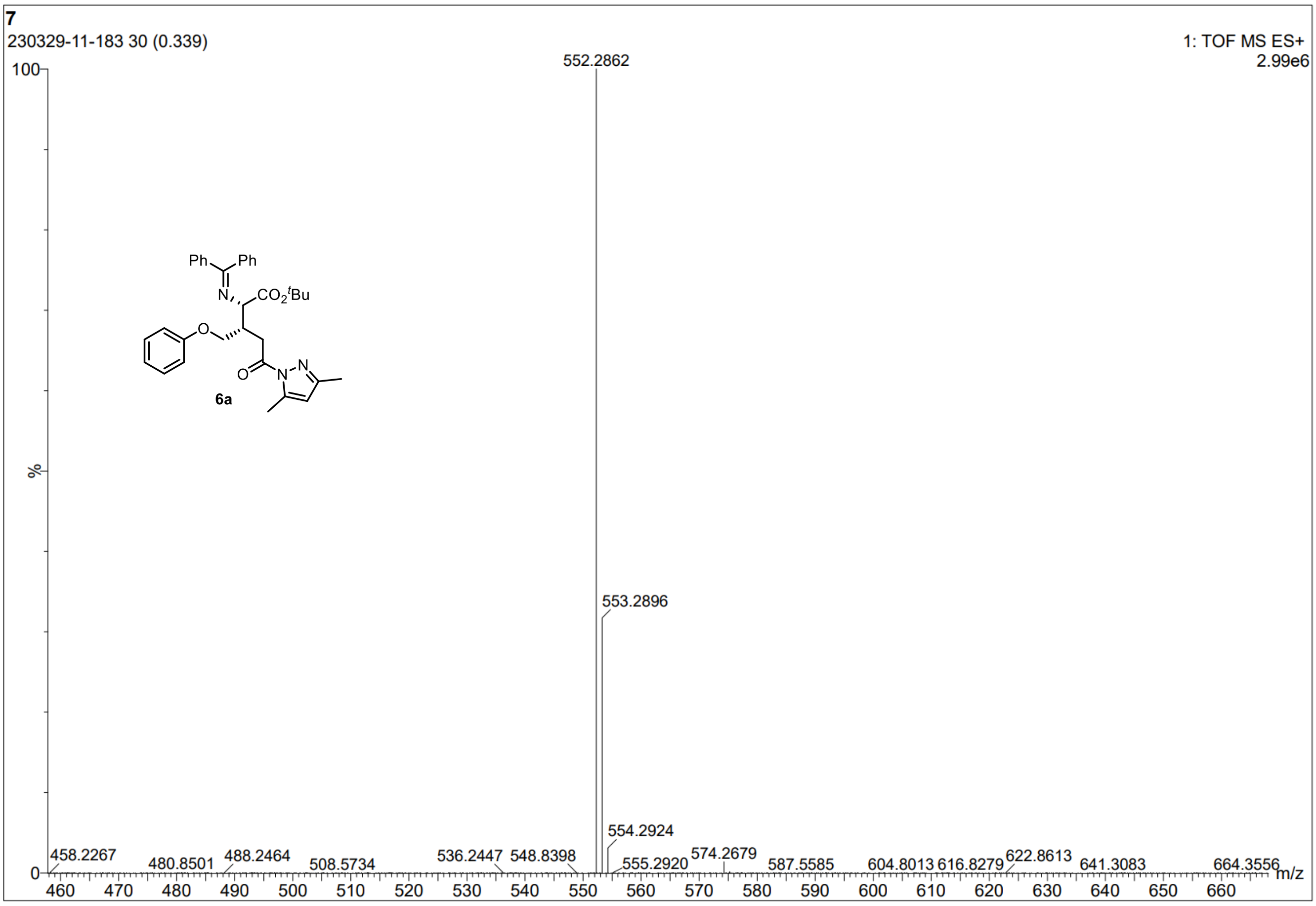
7
230329-11-183 30 (0.339)
1: TOF MS ES+
2.99e6
552.2862
553.2896
554.2924
458.2267
480.8501
488.2464
508.5734
536.2447
548.8398
555.2920
574.2679
587.5585
604.8013
616.8279
622.8613
641.3083
664.3556
%
m/z
6a

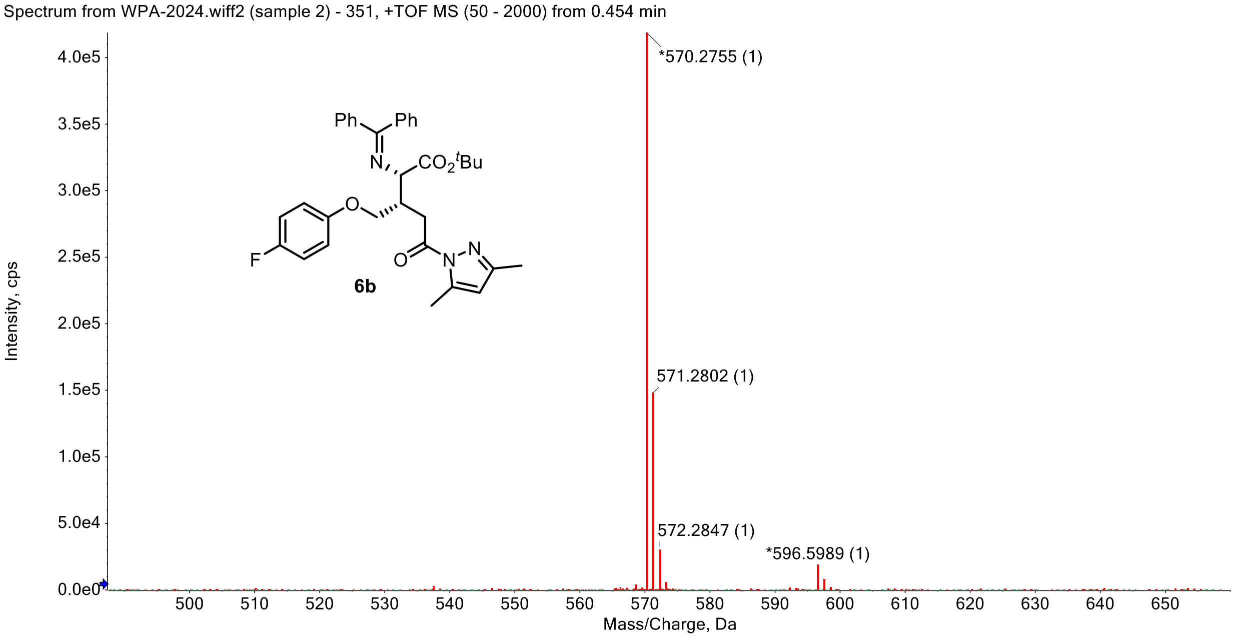
Spectrum from WPA-2024.wiff2 (sample 2) - 351, +TOF MS (50 - 2000) from 0.454 min
*570.2755 (1)
571.2802 (1)
572.2847 (1)
*596.5989 (1)
Intensity, cps
Mass/Charge, Da
6b

Spectrum from WPA-2024.wiff2 (sample 19) - 367, +TOF MS (50 - 2000) from 0.556 min

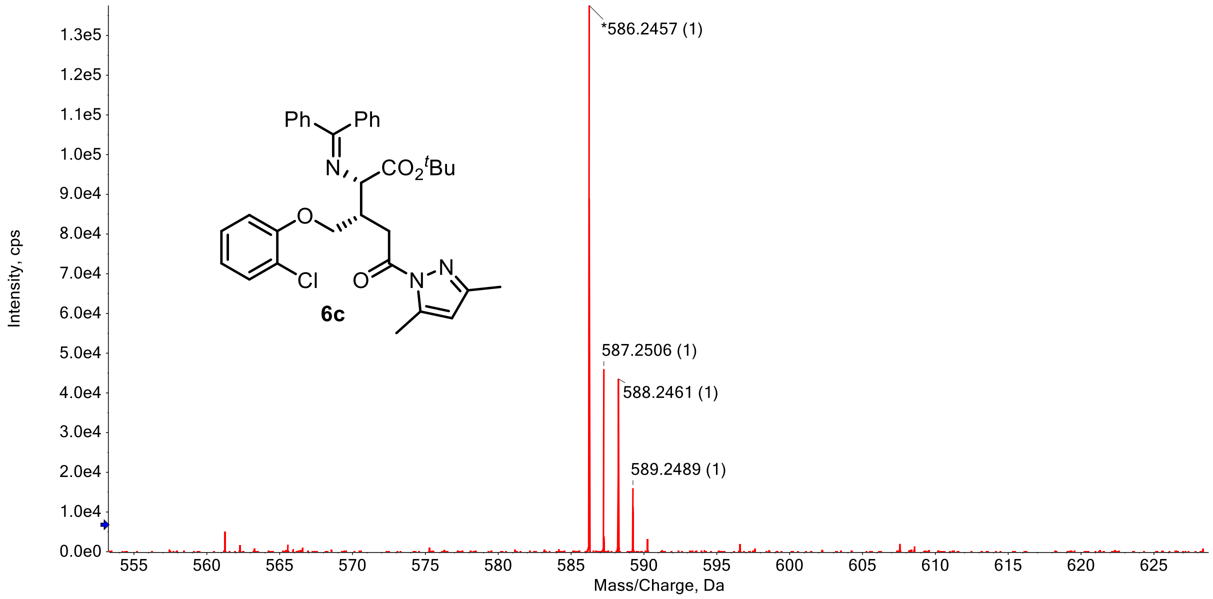

Spectrum from WPA-2024.wiff2 (sample 8) - 357, +TOF MS (50 - 2000) from 0.510 min

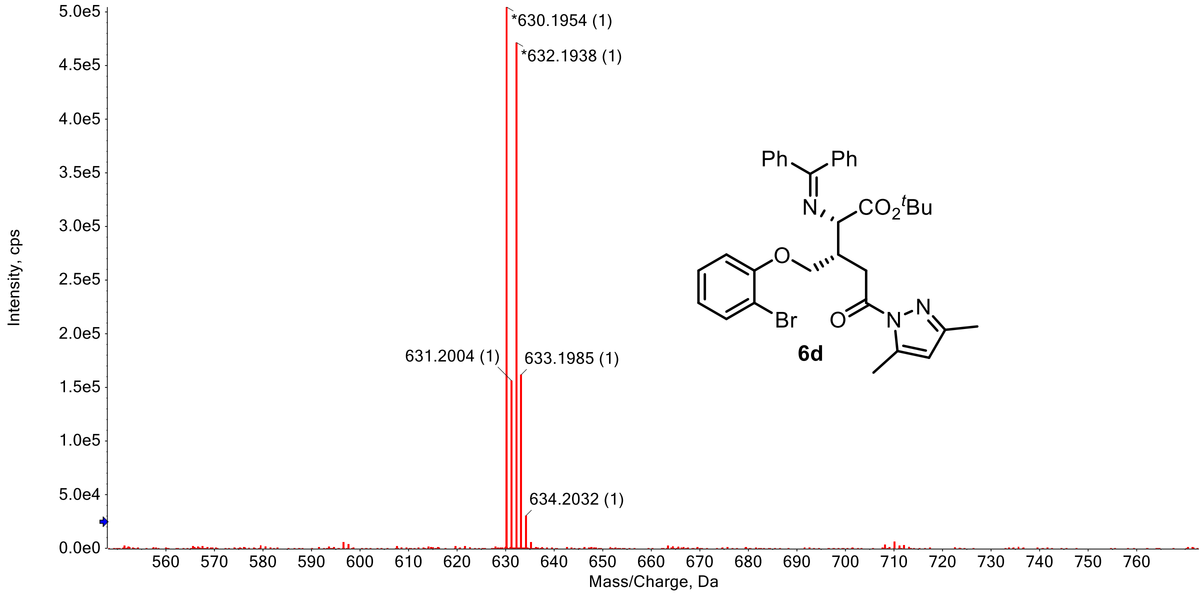

Spectrum from WPA-1022.wiff2 (sample 3) - WXY-3, +TOF MS (50 - 2000) from 0.272 min

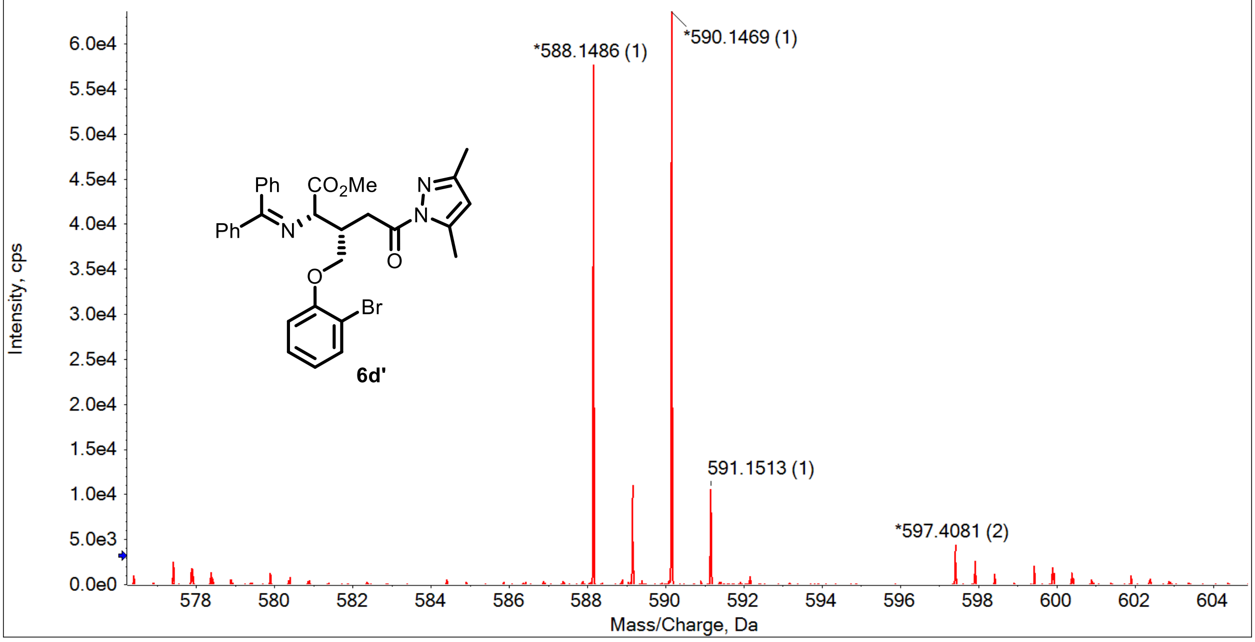

Spectrum from WPA-2024.wiff2 (sample 6) - 355, +TOF MS (50 - 2000) from 0.533 min

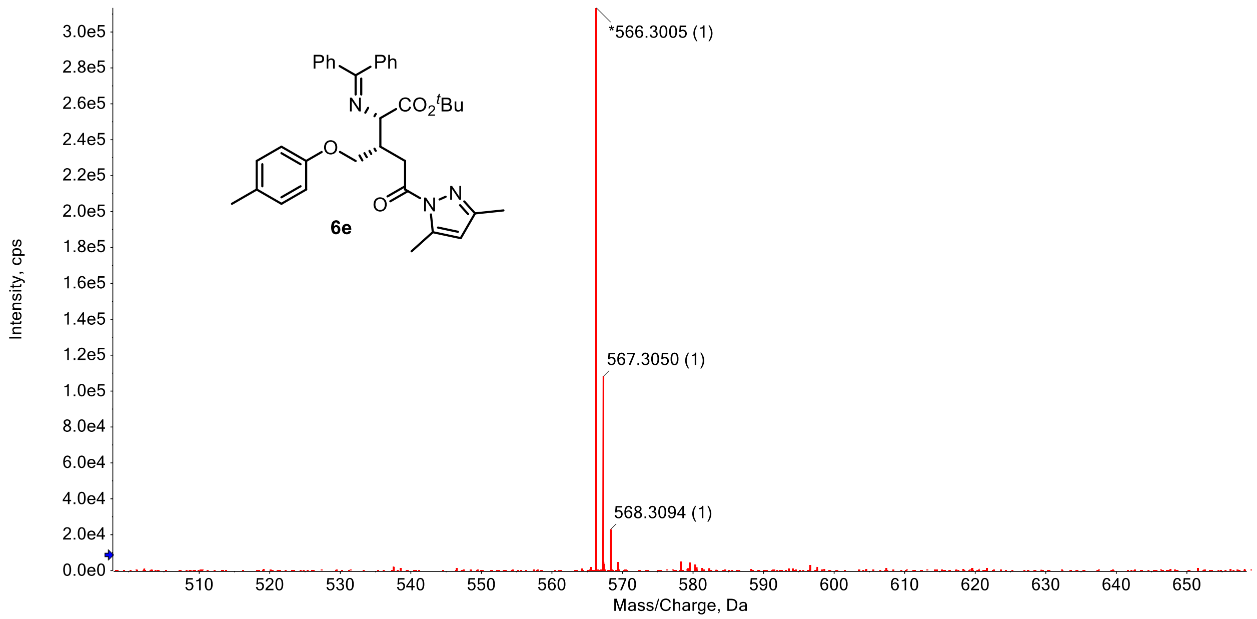

Spectrum from WPA-2024.wiff2 (sample 10) - 359, +TOF MS (50 - 2000) from 0.454 min

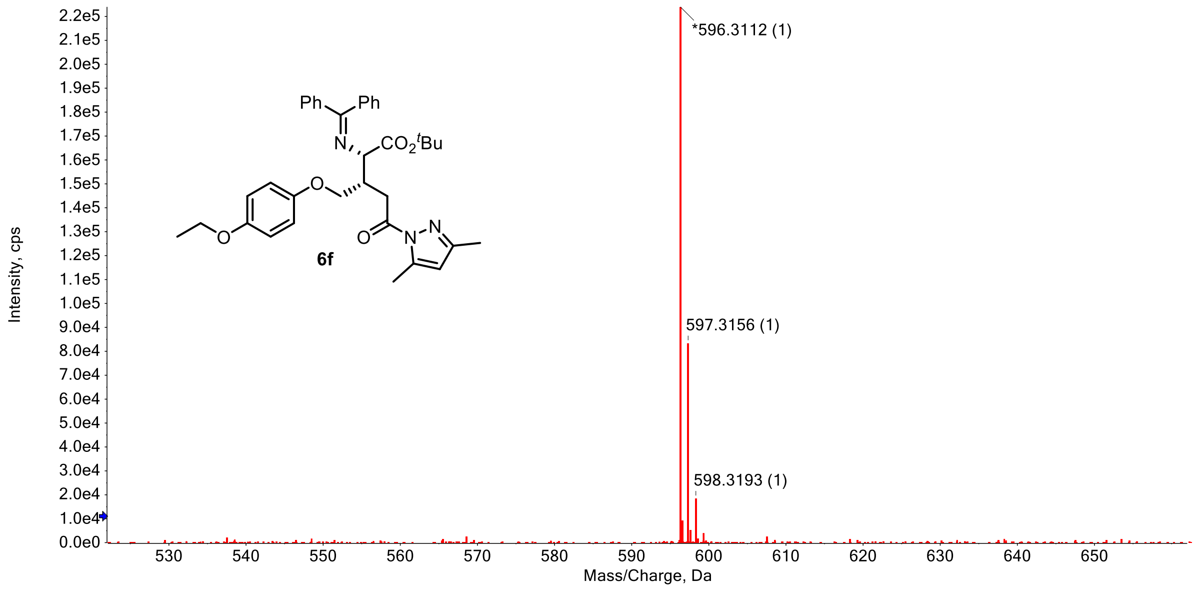

Spectrum from WPA-2024.wiff2 (sample 50) - 374, +TOF MS (50 - 2000) from 0.487 min

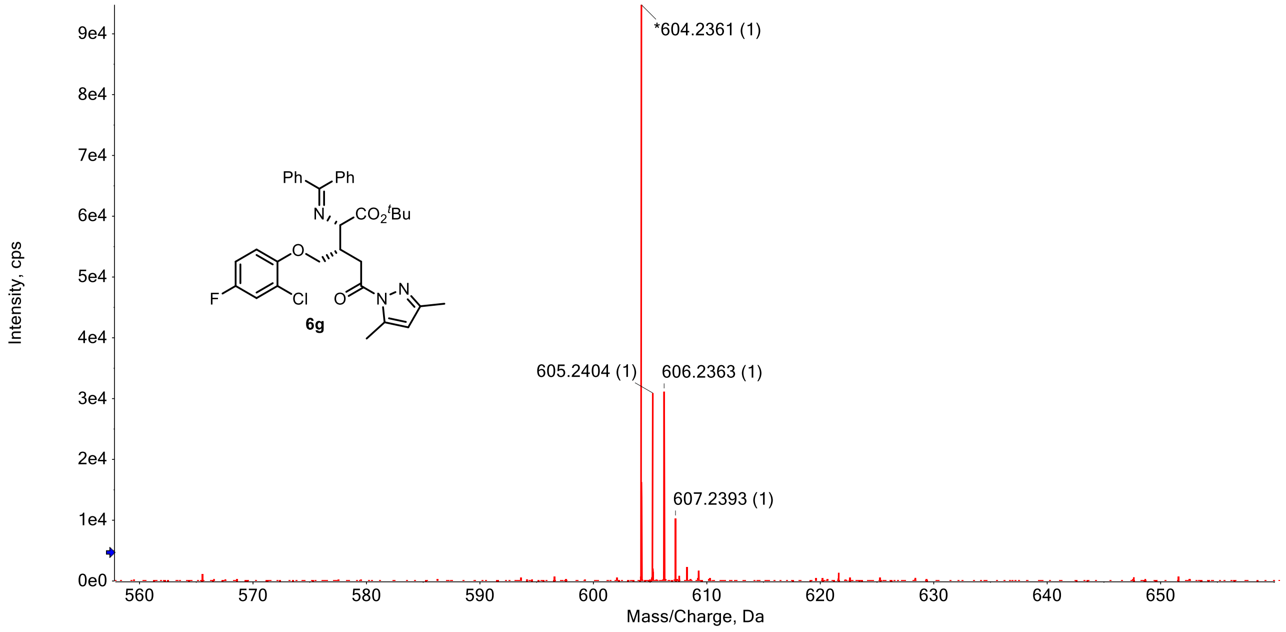

Spectrum from WPA-2024.wiff2 (sample 14) - 363, +TOF MS (50 - 2000) from 0.366 min

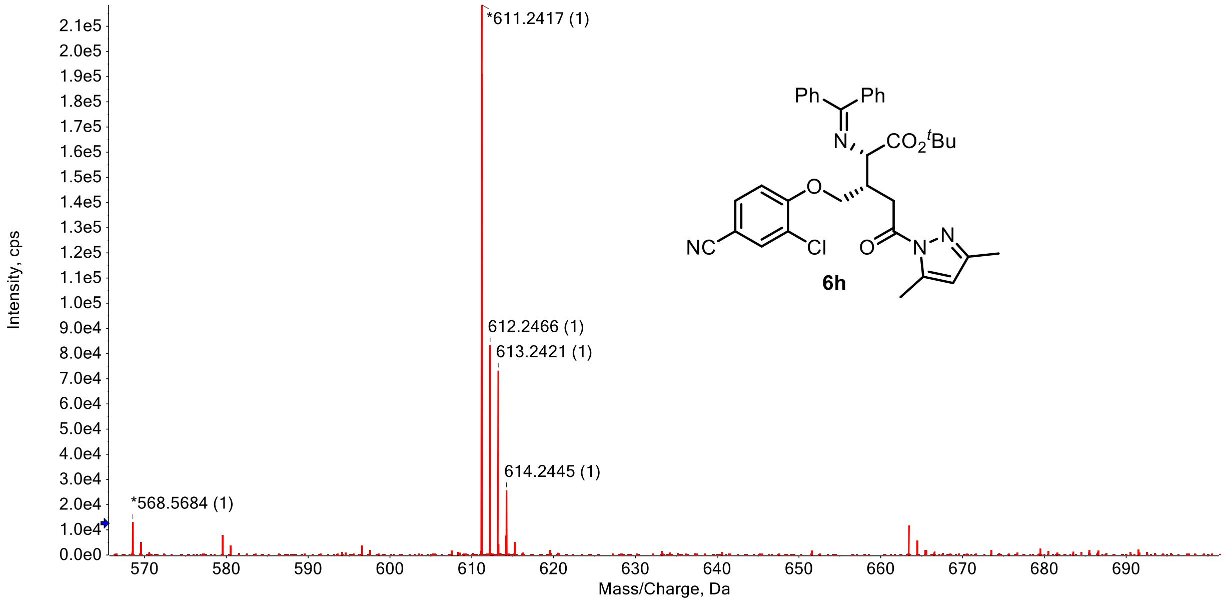

Spectrum from WPA-2024.wiff2 (sample 48) - 369, +TOF MS (50 - 2000) from 0.519 min

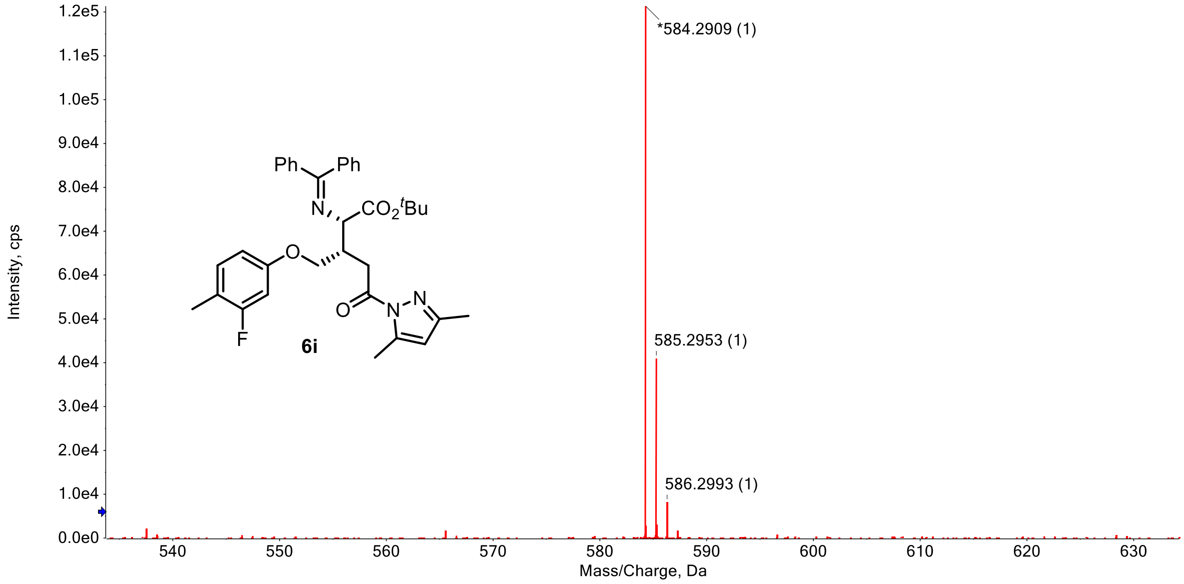

Spectrum from WPA-2024.wiff2 (sample 43) - 361, +TOF MS (50 - 2000) from 0.737 min

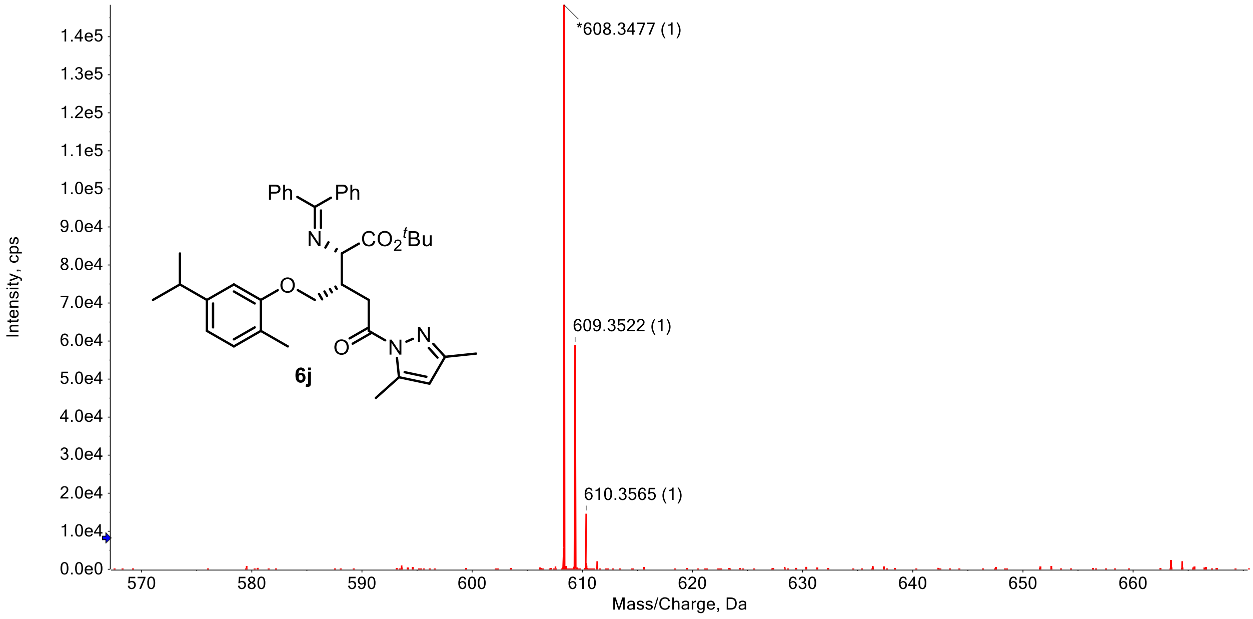

Spectrum from WPA-2024.wiff2 (sample 4) - 353, +TOF MS (50 - 2000) from 0.315 min

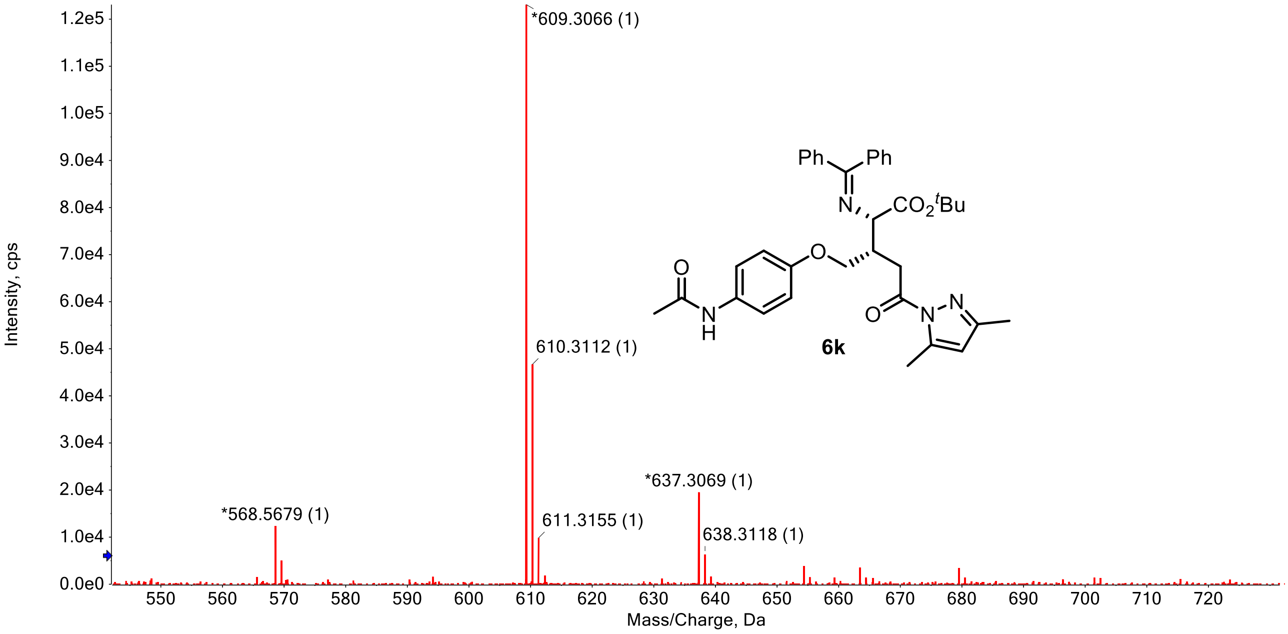

Spectrum from WPA-2024.wiff2 (sample 49) - 372, +TOF MS (50 - 2000) from 0.482 min

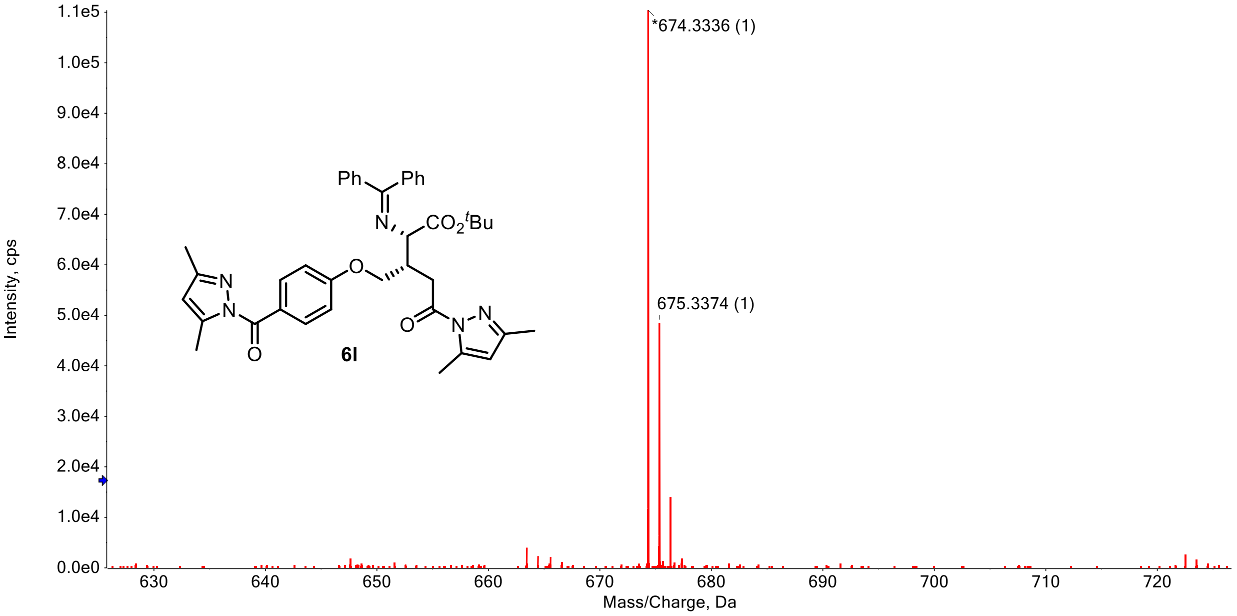

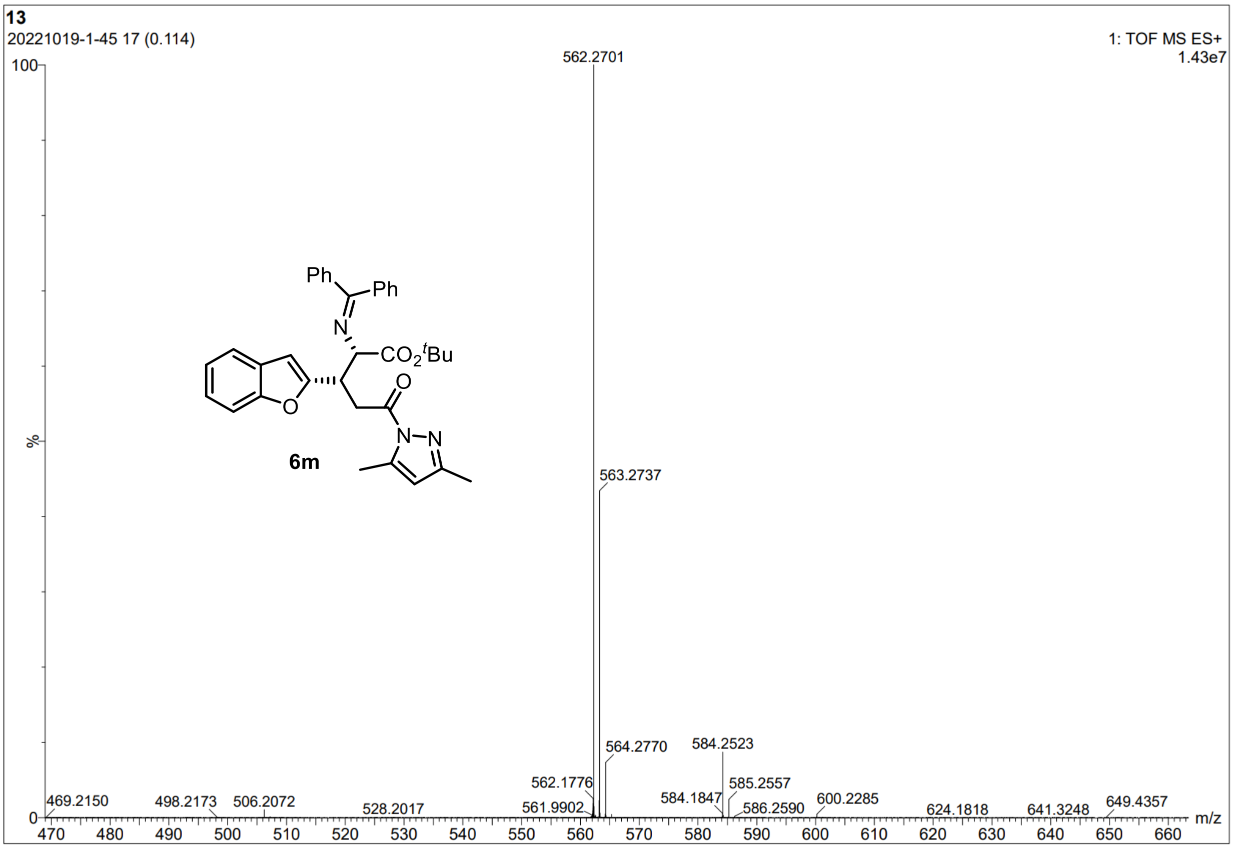
13
20221019-1-45 17 (0.114)
1: TOF MS ES+
1.43e7
562.2701
563.2737
564.2770
584.2523
562.1776
585.2557
584.1847
561.9902
586.2590
600.2285
469.2150
498.2173
506.2072
528.2017
624.1818
641.3248
649.4357
m/z
%
6m

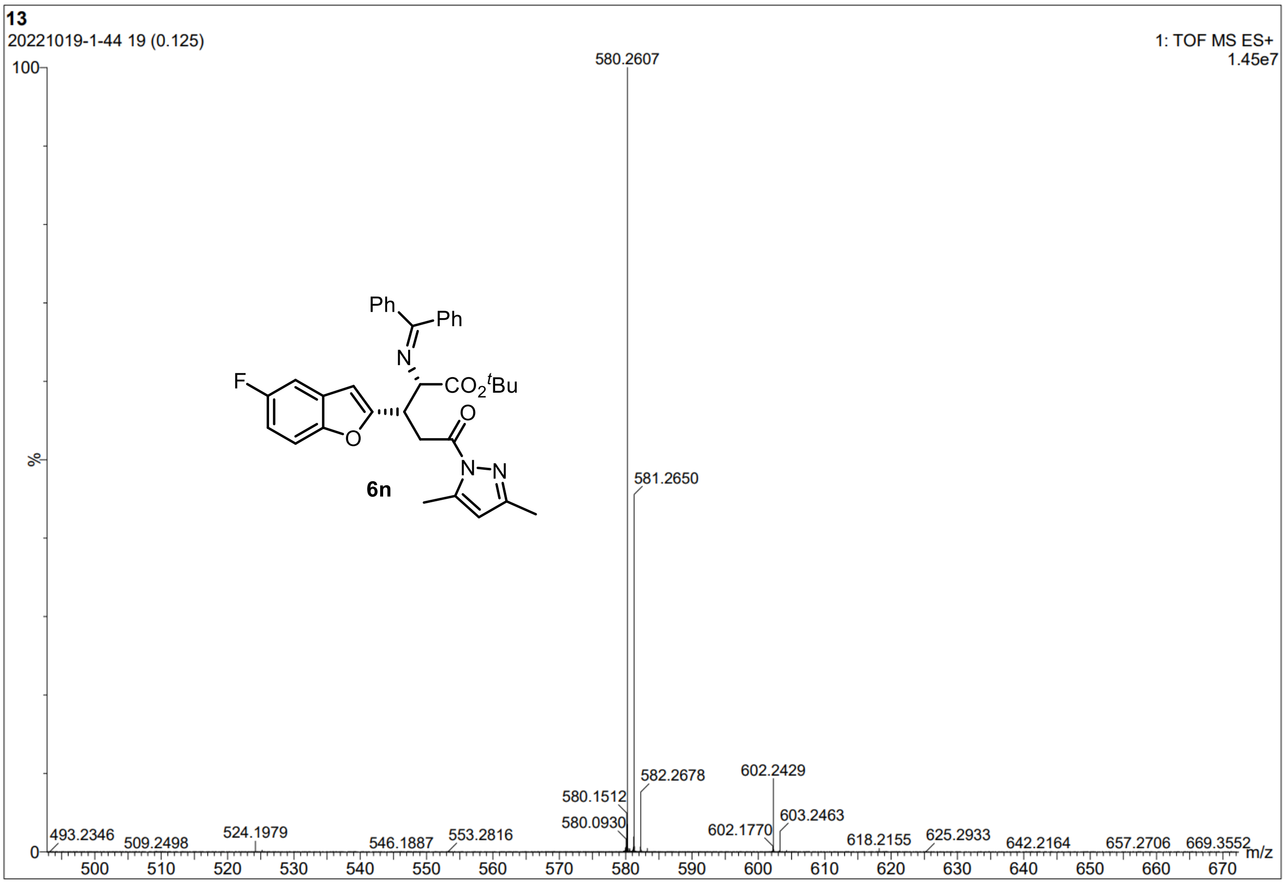
13
20221019-1-44 19 (0.125)
1: TOF MS ES+
1.45e7
580.2607
581.2650
582.2678
602.2429
580.1512
580.0930
603.2463
602.1770
493.2346
509.2498
524.1979
546.1887
553.2816
618.2155
625.2933
642.2164
657.2706
669.3552
m/z
%
6n

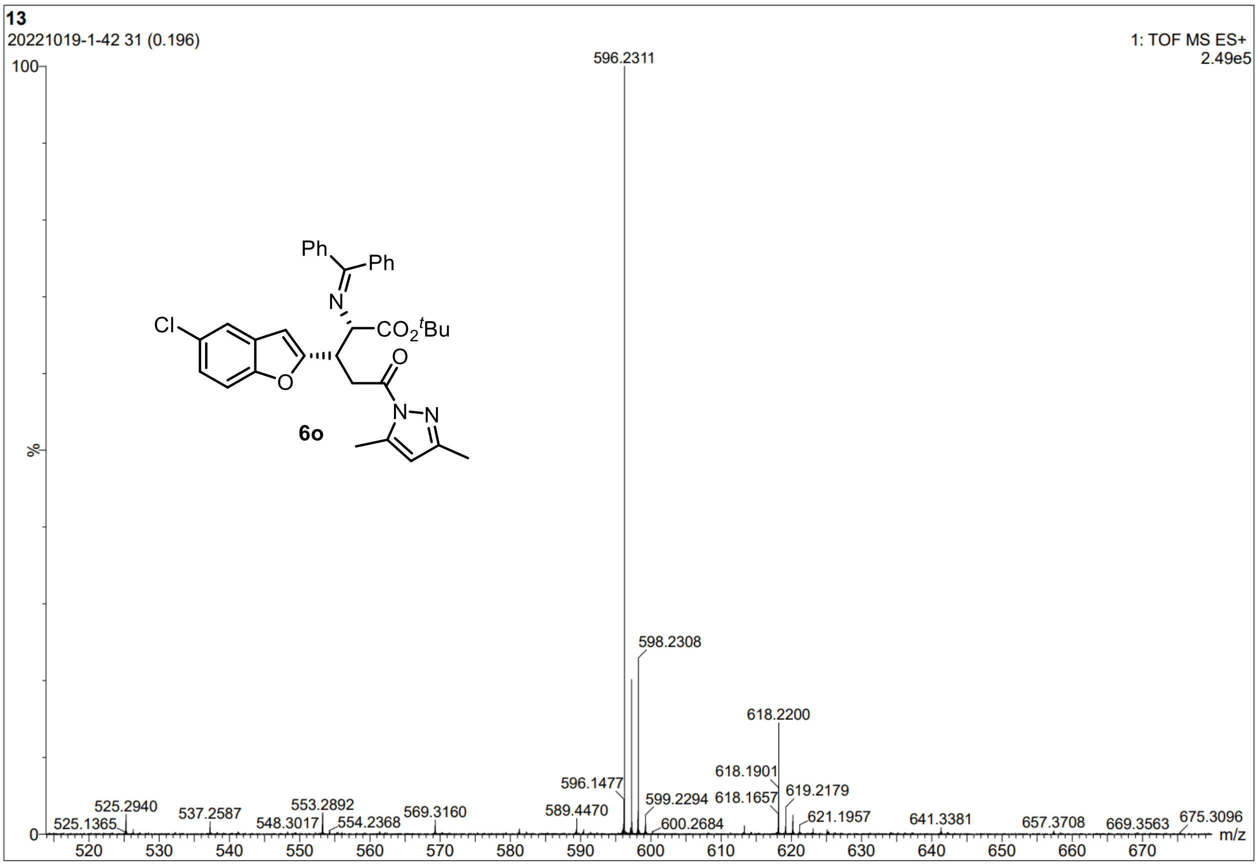
13
20221019-1-42 31 (0.196)
1: TOF MS ES+
2.49e5
596.2311
598.2308
618.2200
596.1477
618.1901
599.2294
618.1657
619.2179
621.1957
600.2684
589.4470
525.2940
525.1365
537.2587
553.2892
548.3017
554.2368
569.3160
641.3381
657.3708
669.3563
675.3096
m/z
%
6o

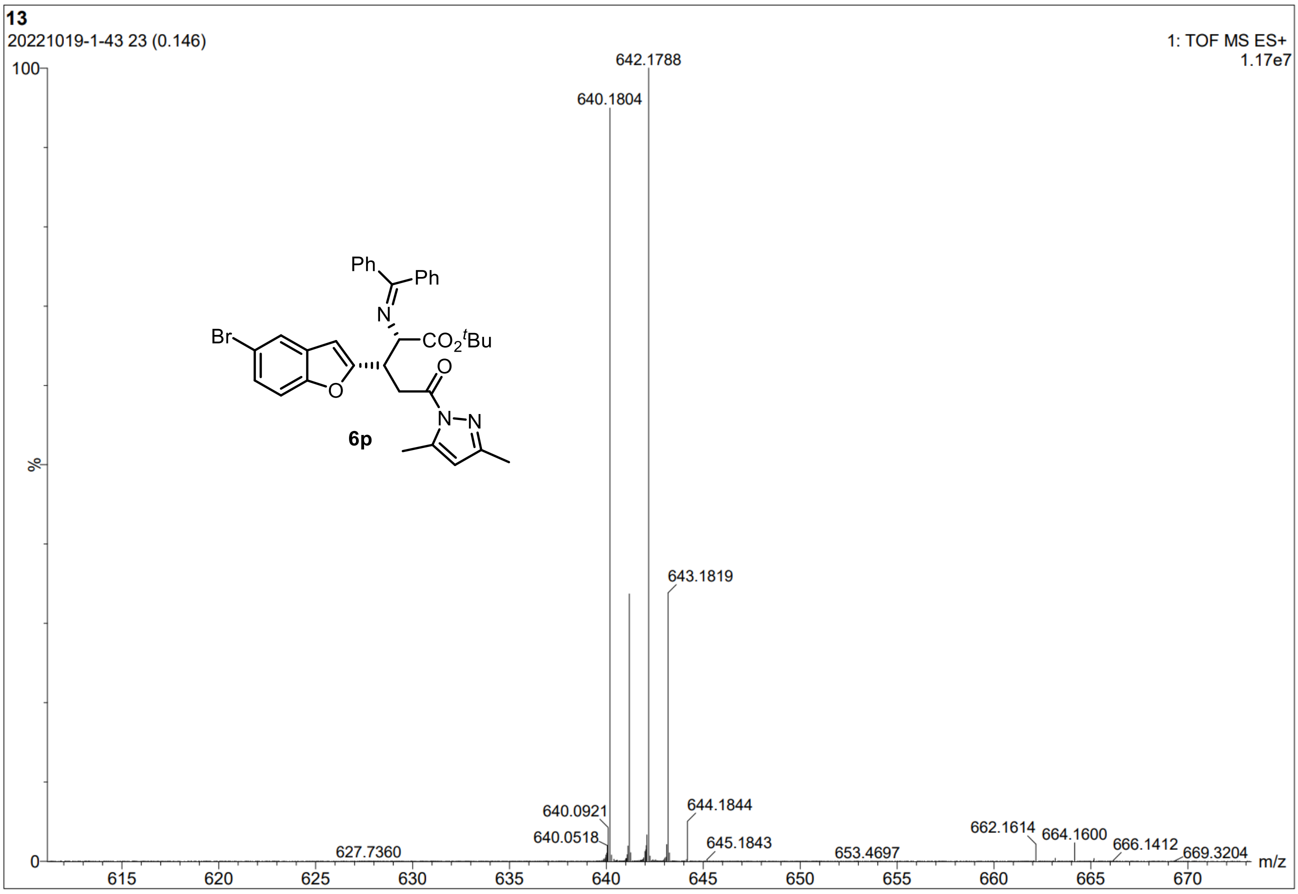
13
20221019-1-43 23 (0.146)
1: TOF MS ES+
1.17e7
642.1788
640.1804
643.1819
640.0921
640.0518
644.1844
645.1843
627.7360
653.4697
662.1614
664.1600
666.1412
669.3204
m/z
%
6p

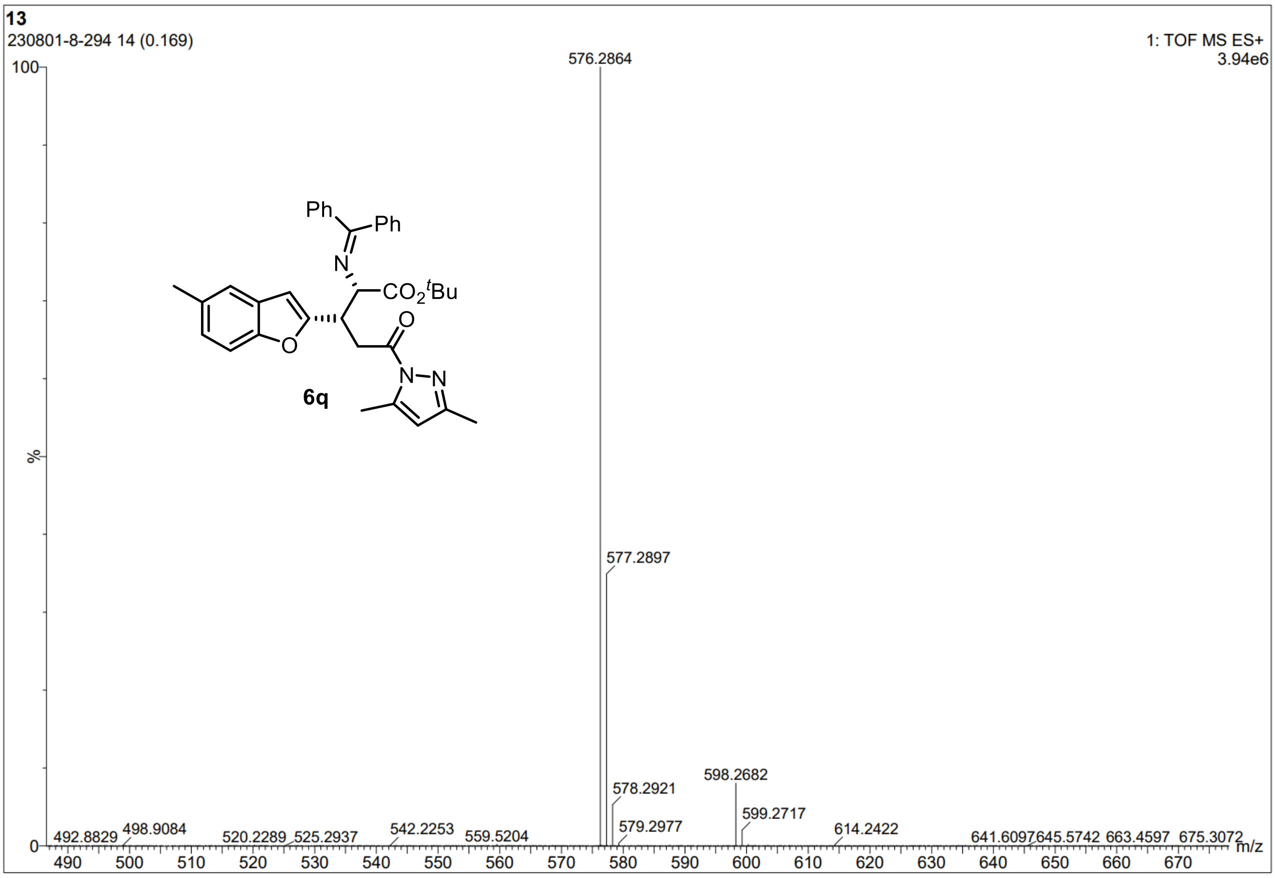
13
230801-8-294 14 (0.169)
1: TOF MS ES+
3.94e6
576.2864
577.2897
578.2921
579.2977
598.2682
599.2717
614.2422
492.8829
498.9084
520.2289
525.2937
542.2253
559.5204
641.6097
645.5742
663.4597
675.3072
m/z
%
6q

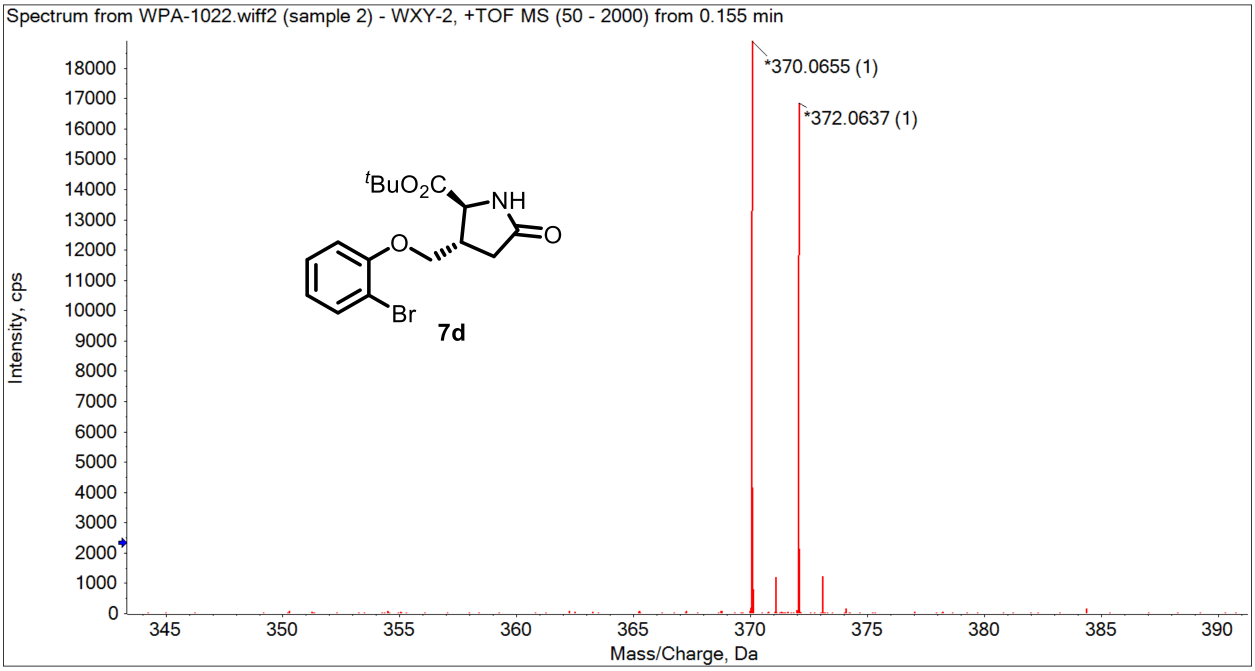
Spectrum from WPA-1022.wiff2 (sample 2) - WXY-2, +TOF MS (50 - 2000) from 0.155 min
*370.0655 (1)
*372.0637 (1)
Intensity, cps
Mass/Charge, Da
7d

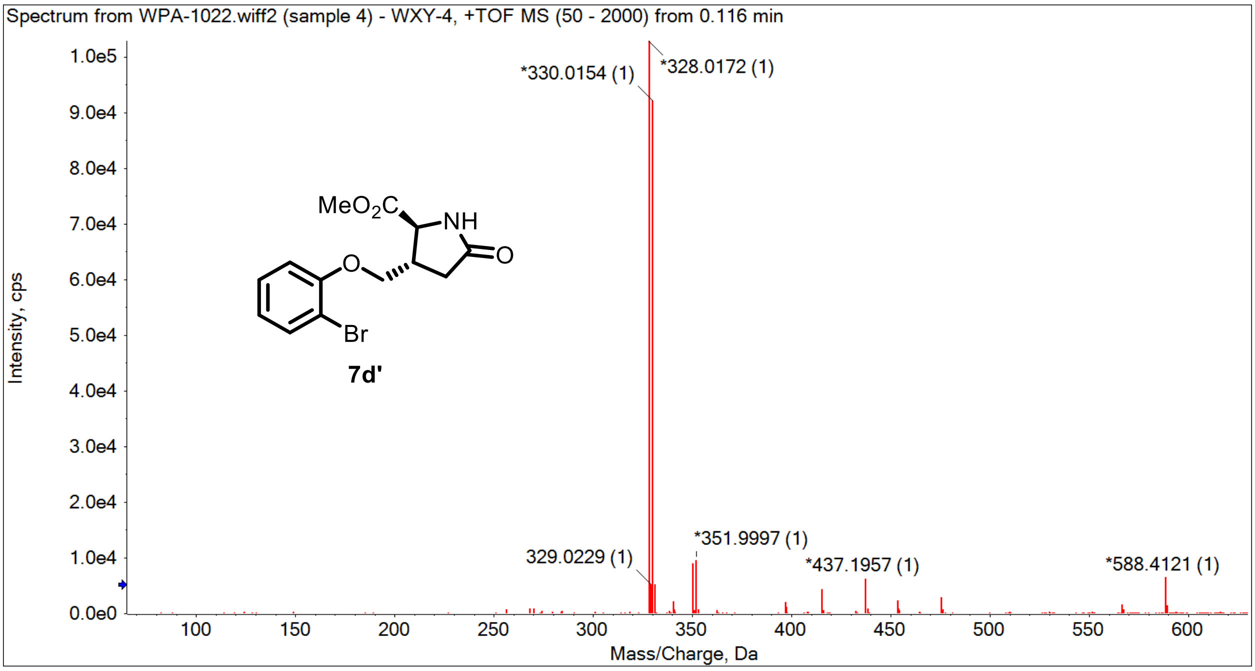

Spectrum from WPA-1022.wiff2 (sample 4) - WXY-4, +TOF MS (50 - 2000) from 0.116 min
*330.0154 (1)
*328.0172 (1)
329.0229 (1)
*351.9997 (1)
*437.1957 (1)
*588.4121 (1)
Intensity, cps
Mass/Charge, Da
MeO2C
NH
O
Br
7d'

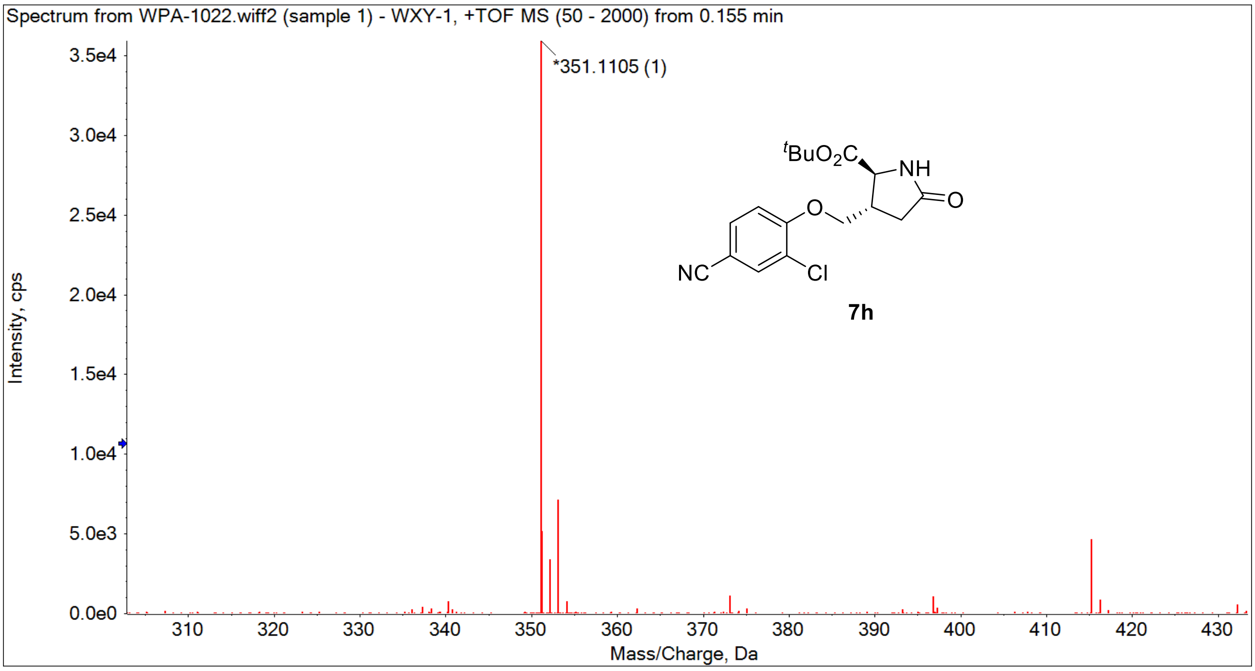

Spectrum from WPA-1022.wiff2 (sample 1) - WXY-1, +TOF MS (50 - 2000) from 0.155 min
*351.1105 (1)
Intensity, cps
Mass/Charge, Da
tBuO2C
NH
O
NC
Cl
7h

6. HPLC Charts

6a: Chiralpak OD-H, Hexane/2-Propanol = 90:10, flow rate 0.8 mL/min, 254 nm, 98% *ee*

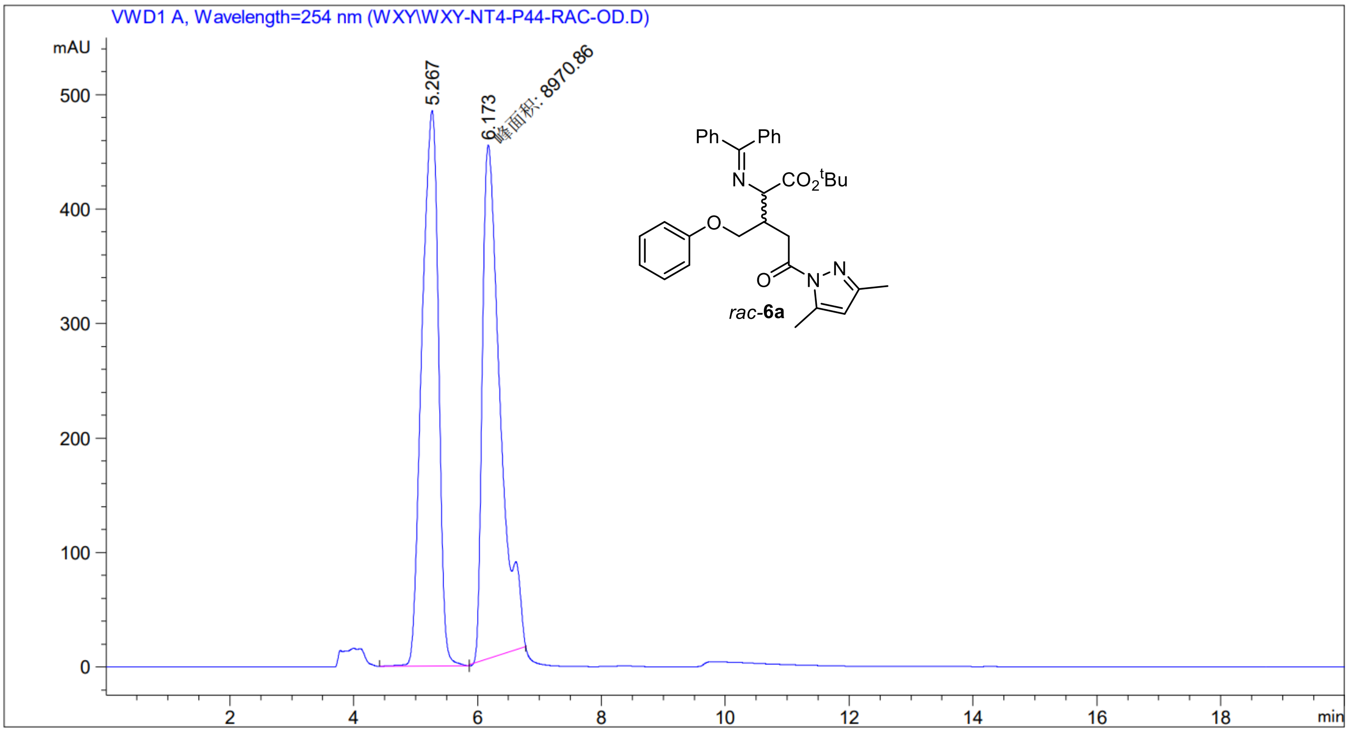

峰 #	保留时间 [min]	类型	峰宽 [min]	峰面积 [mAU*s]	峰高 [mAU]	峰面积 %
1	5.267	BB	0.3114	8998.98340	485.20590	50.0783
2	6.173	MM	0.3333	8970.85547	448.53177	49.9217

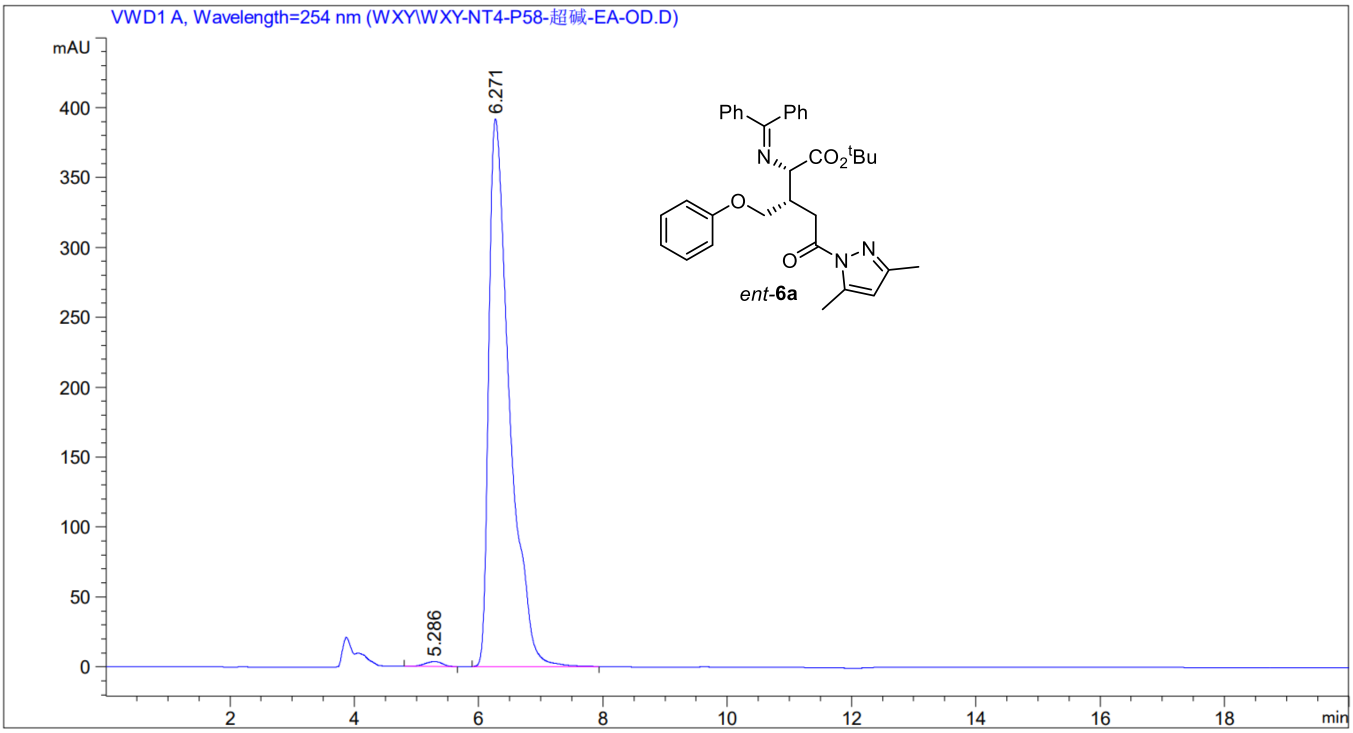

峰 #	保留时间 [min]	类型	峰宽 [min]	峰面积 [mAU*s]	峰高 [mAU]	峰面积 %
1	5.286	BB	0.3096	66.95730	3.44667	0.7338
2	6.271	VB R	0.3396	9058.12012	391.70947	99.2662

6b: Chiralpak OD-H, Hexane/2-Propanol = 90:10, flow rate 0.8 mL/min, 254 nm, 98% *ee*

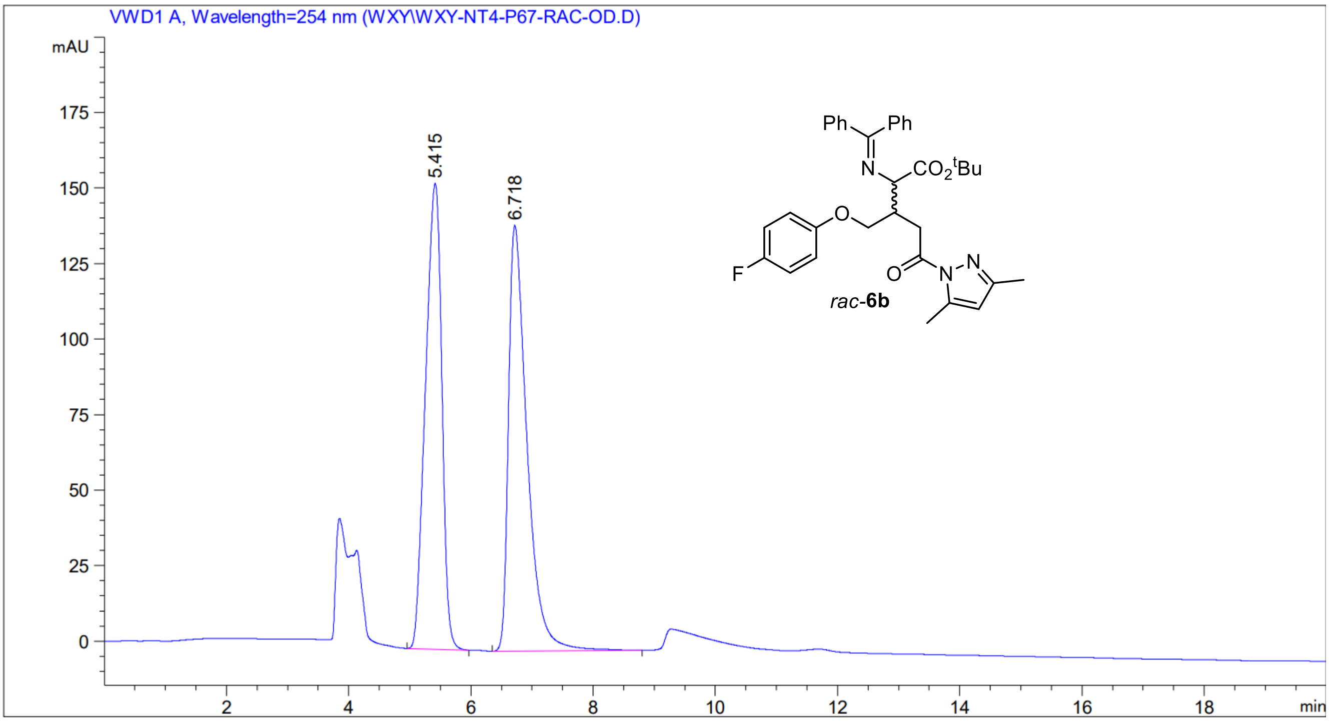

峰 #	保留时间 [min]	类型	峰宽 [min]	峰面积 [mAU*s]	峰高 [mAU]	峰面积 %
1	5.415	BB	0.2931	2857.42822	154.23409	49.0064
2	6.718	BB	0.3160	2973.30078	141.00313	50.9936

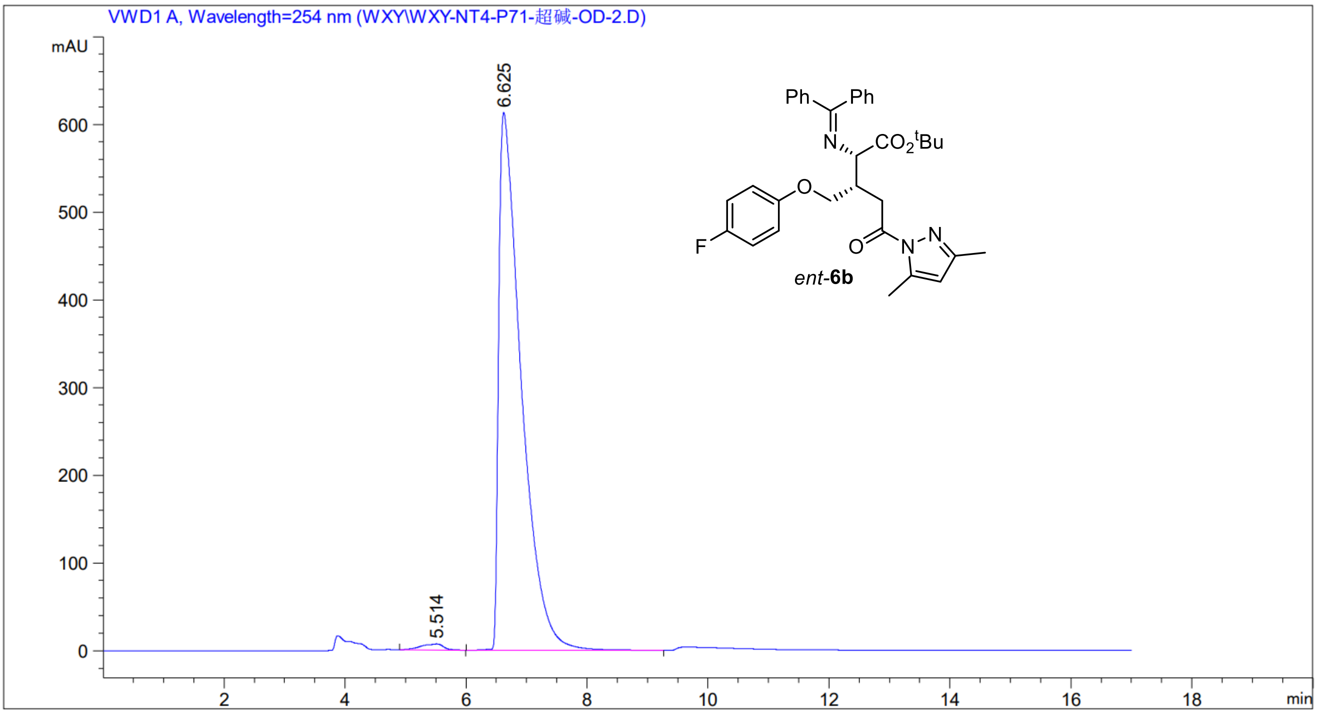

峰 #	保留时间 [min]	类型	峰宽 [min]	峰面积 [mAU*s]	峰高 [mAU]	峰面积 %
1	5.514	VB	0.3655	191.26997	7.09503	1.1939
2	6.625	BB	0.3943	1.58288e4	612.89136	98.8061

6c: Chiralpak OD-H, Hexane/2-Propanol = 90:10, flow rate 0.8 mL/min, 254 nm, 98% *ee*

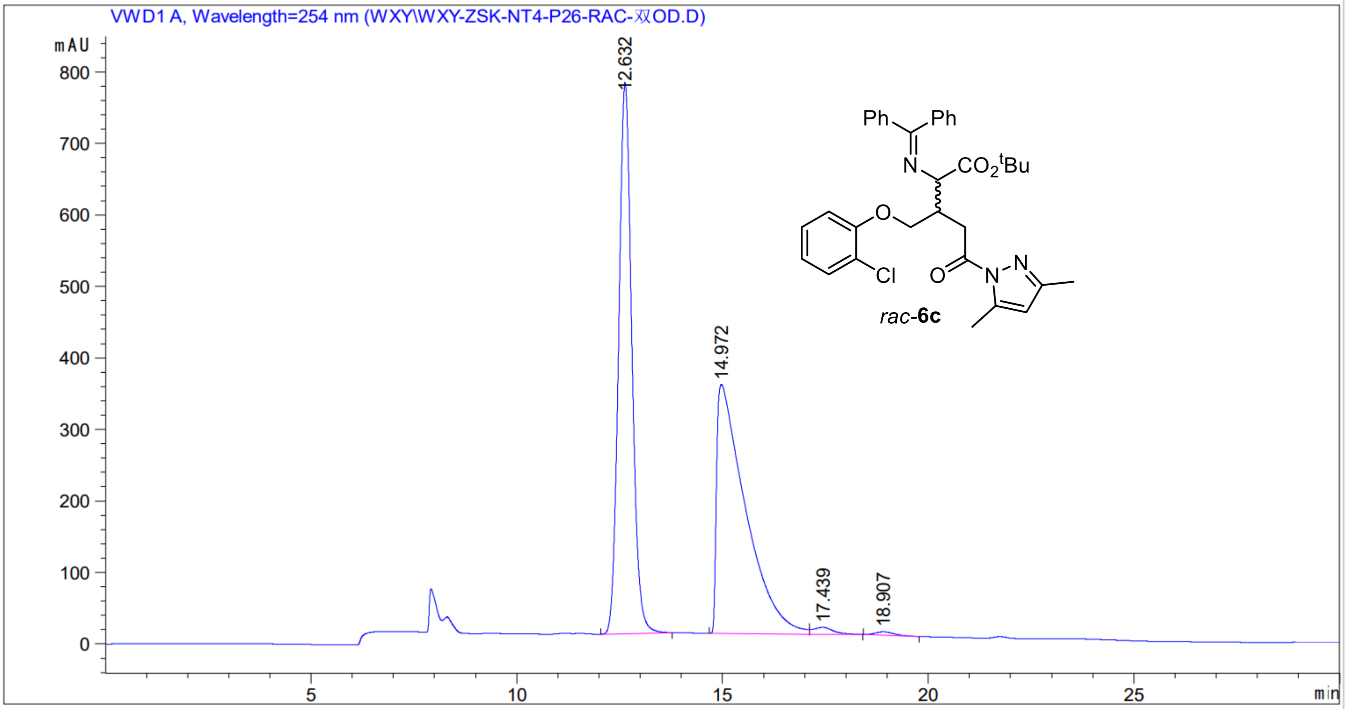

峰 #	保留时间 [min]	类型	峰宽 [min]	峰面积 [mAU*s]	峰高 [mAU]	峰面积 %
1	12.632	BB	0.3395	1.71533e4	771.12024	49.5547
2	14.972	BV	0.6838	1.69842e4	348.37381	49.0661
3	17.439	VB	0.4941	329.99570	9.62245	0.9533
4	18.907	BB	0.4714	147.39612	4.79813	0.4258

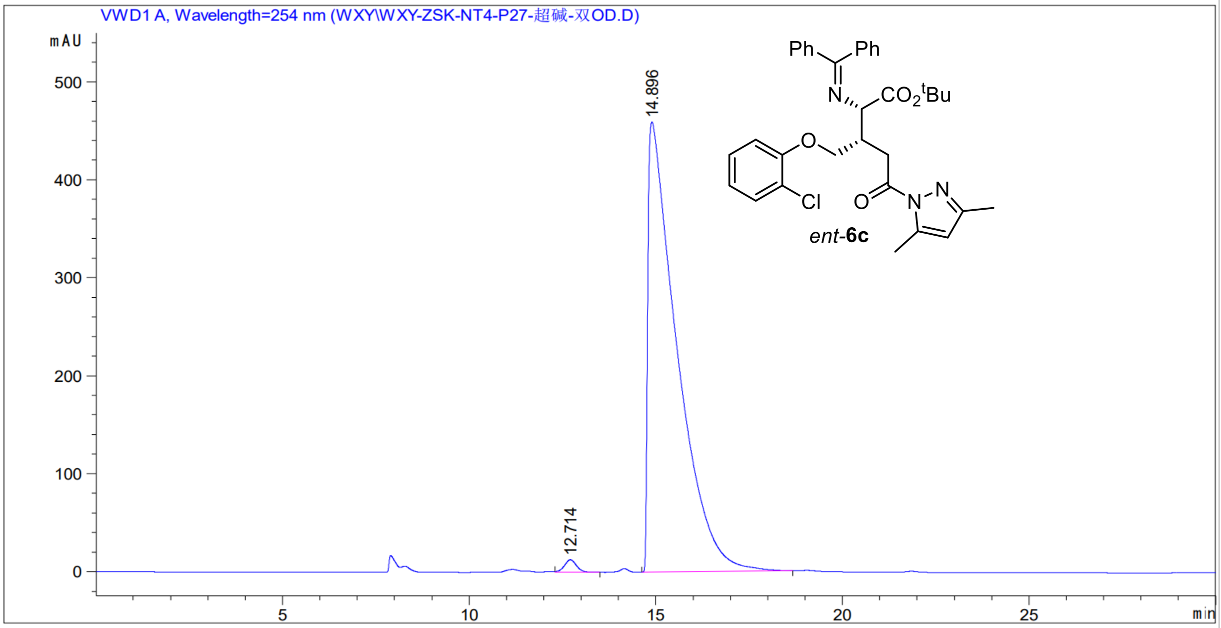

峰 #	保留时间 [min]	类型	峰宽 [min]	峰面积 [mAU*s]	峰高 [mAU]	峰面积 %
1	12.714	VB	0.3389	287.53424	12.80841	1.1464
2	14.896	BB	0.7527	2.47933e4	459.38339	98.8536

6d: Chiralpak OD-H, Hexane/2-Propanol = 90:10, flow rate 0.8 mL/min, 254 nm, 95% *ee*

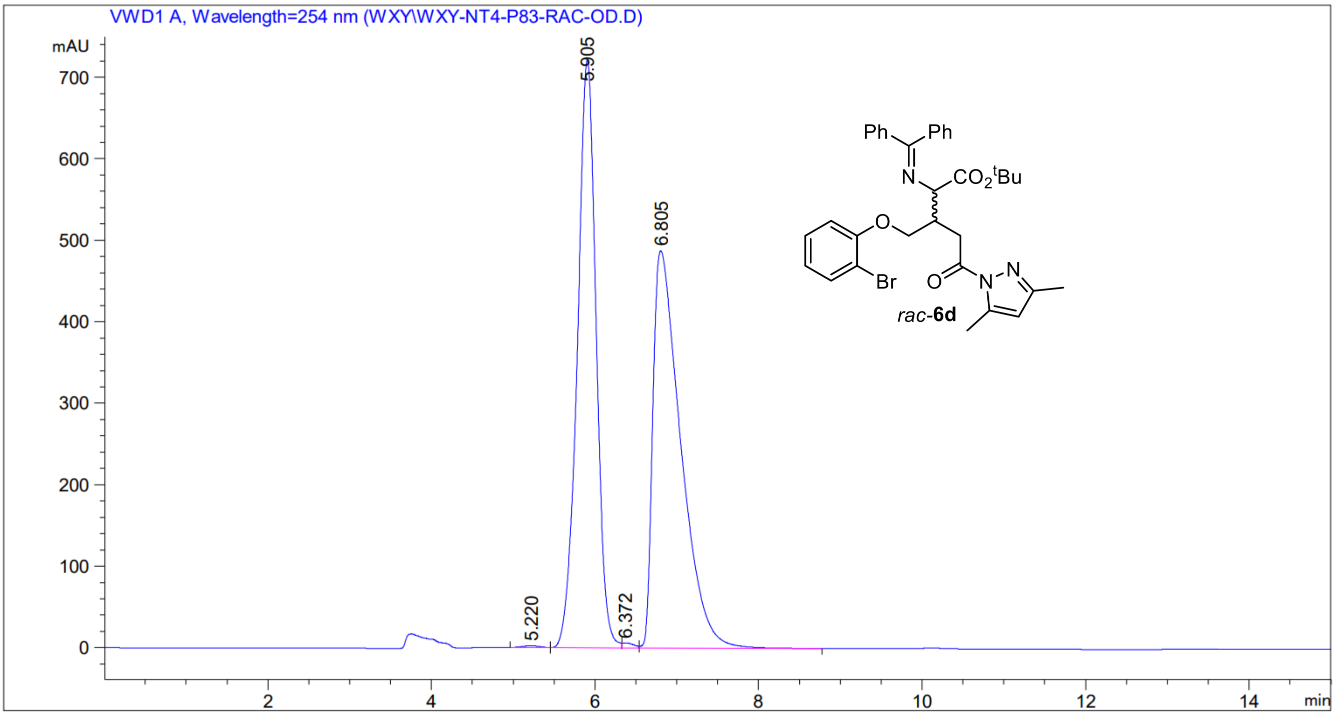

峰 #	保留时间 [min]	类型	峰宽 [min]	峰面积 [mAU*s]	峰高 [mAU]	峰面积 %
1	5.220	VB	0.2515	35.89716	2.25813	0.1554
2	5.905	BV	0.2426	1.14727e4	721.25623	49.6693
3	6.372	VV	0.1468	58.59090	6.11673	0.2537
4	6.805	VB	0.3561	1.15310e4	487.31595	49.9216

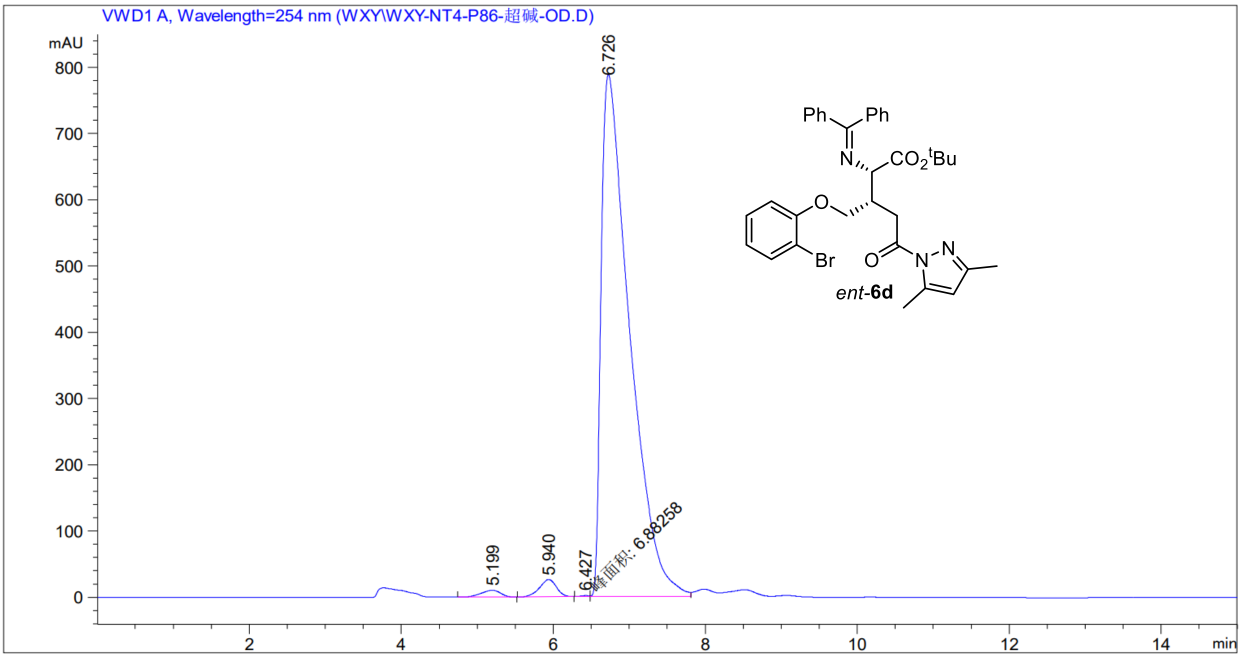

峰 #	保留时间 [min]	类型	峰宽 [min]	峰面积 [mAU*s]	峰高 [mAU]	峰面积 %
1	5.199	BB	0.2820	185.38196	10.34685	0.8790
2	5.940	BB	0.2388	407.62784	26.15991	1.9329
3	6.427	MM	0.1102	6.88258	1.04071	0.0326
4	6.726	VV	0.3812	2.04892e4	788.42419	97.1554

6d': Chiralpak OD-H, Hexane/2-Propanol = 90:10, flow rate 0.8 mL/min, 254 nm, 93.5% *ee*

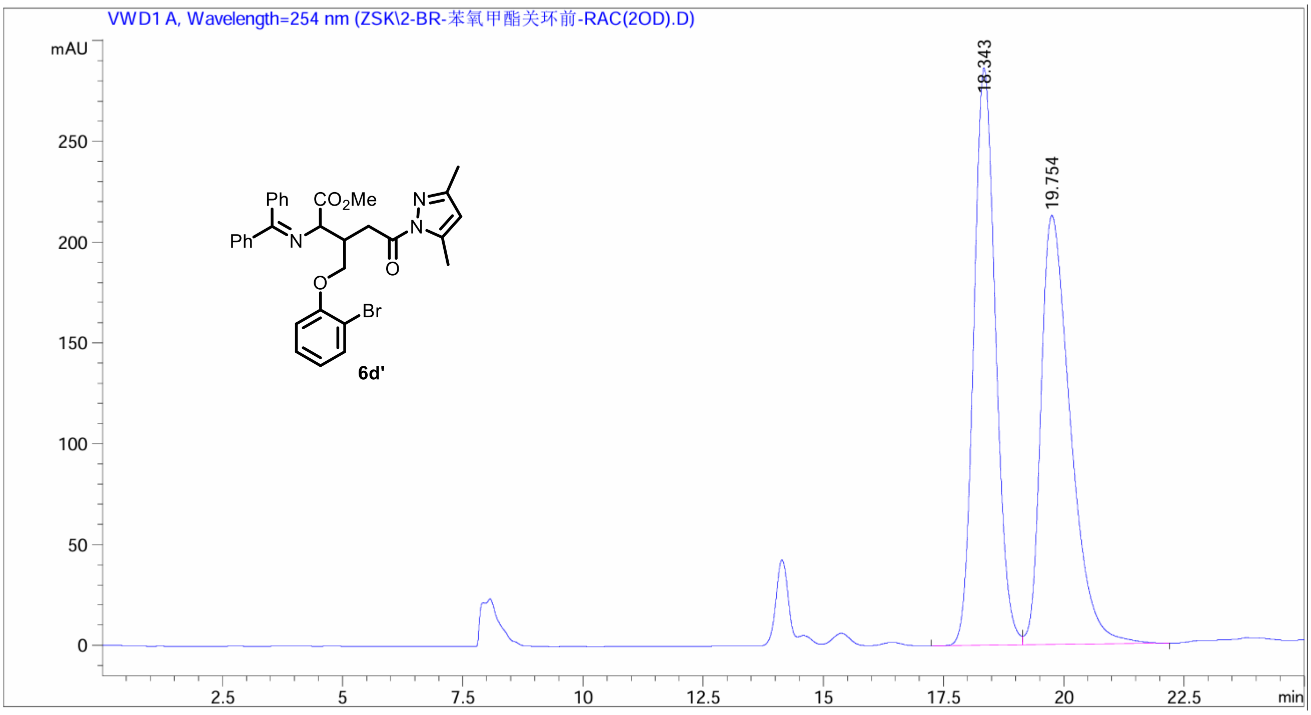

峰 #	保留时间 [min]	类型	峰宽 [min]	峰面积 [mAU*s]	峰高 [mAU]	峰面积 %
1	18.343	BV	0.4914	9040.09863	286.31525	49.8499
2	19.754	VB	0.6536	9094.53418	212.79874	50.1501

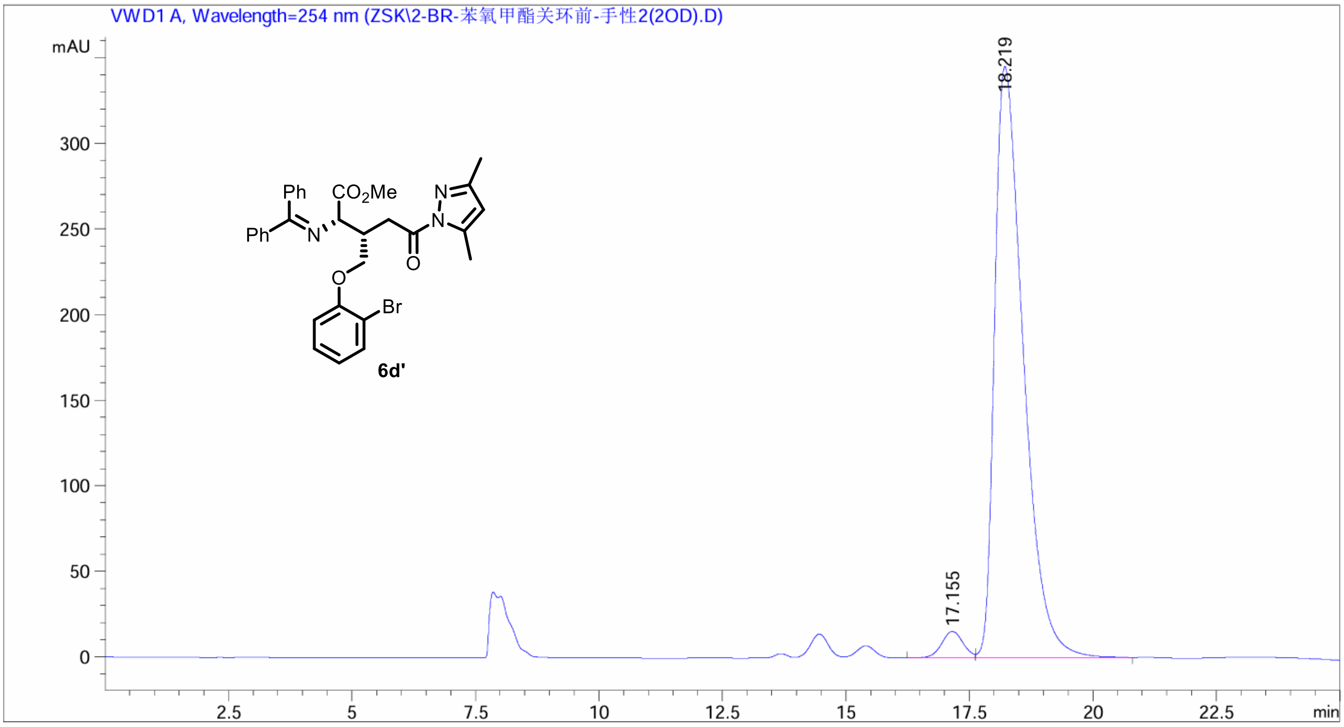

峰 #	保留时间 [min]	类型	峰宽 [min]	峰面积 [mAU*s]	峰高 [mAU]	峰面积 %
1	17.155	BV	0.4688	468.46130	15.49130	3.2505
2	18.219	VB	0.6199	1.39436e4	345.42664	96.7495

6e: Chiralpak OD-H, Hexane/2-Propanol = 90:10, flow rate 0.8 mL/min, 254 nm, 96% *ee*

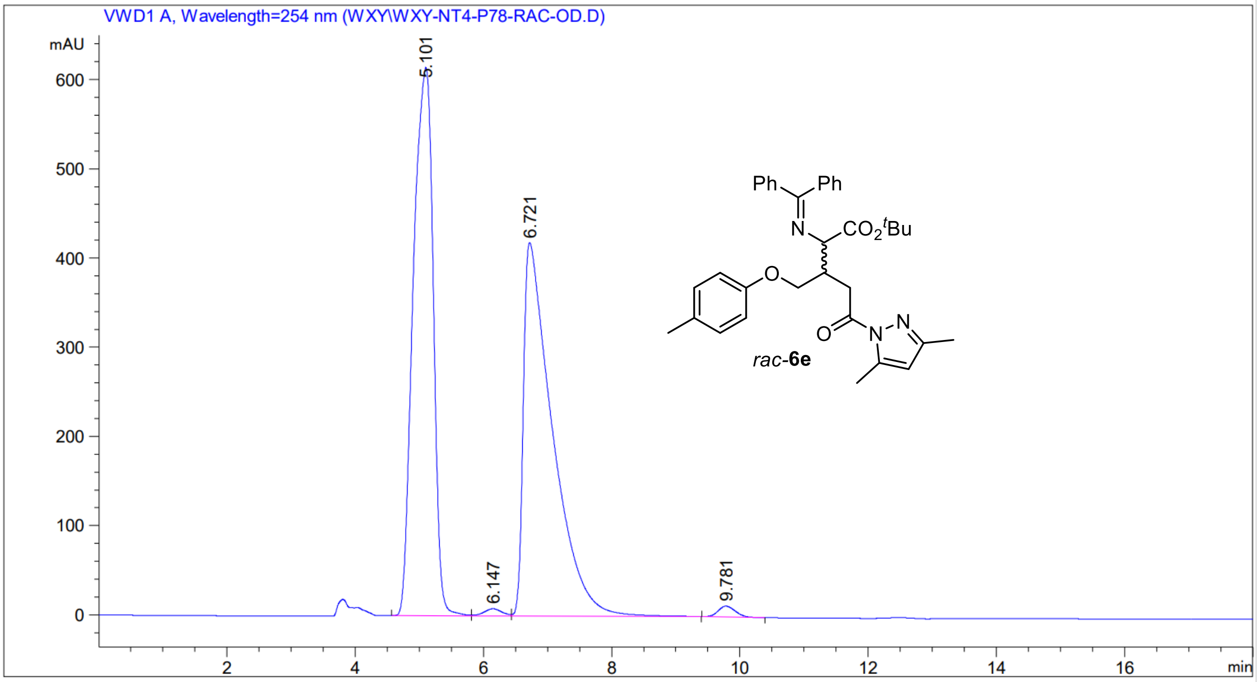

峰 #	保留时间 [min]	类型	峰宽 [min]	峰面积 [mAU*s]	峰高 [mAU]	峰面积 %
1	5.101	BV	0.3595	1.32455e4	614.36096	49.4506
2	6.147	VV	0.2876	152.34052	7.98296	0.5687
3	6.721	VB	0.4487	1.31604e4	418.70468	49.1330
4	9.781	BB	0.2924	227.05817	12.18659	0.8477

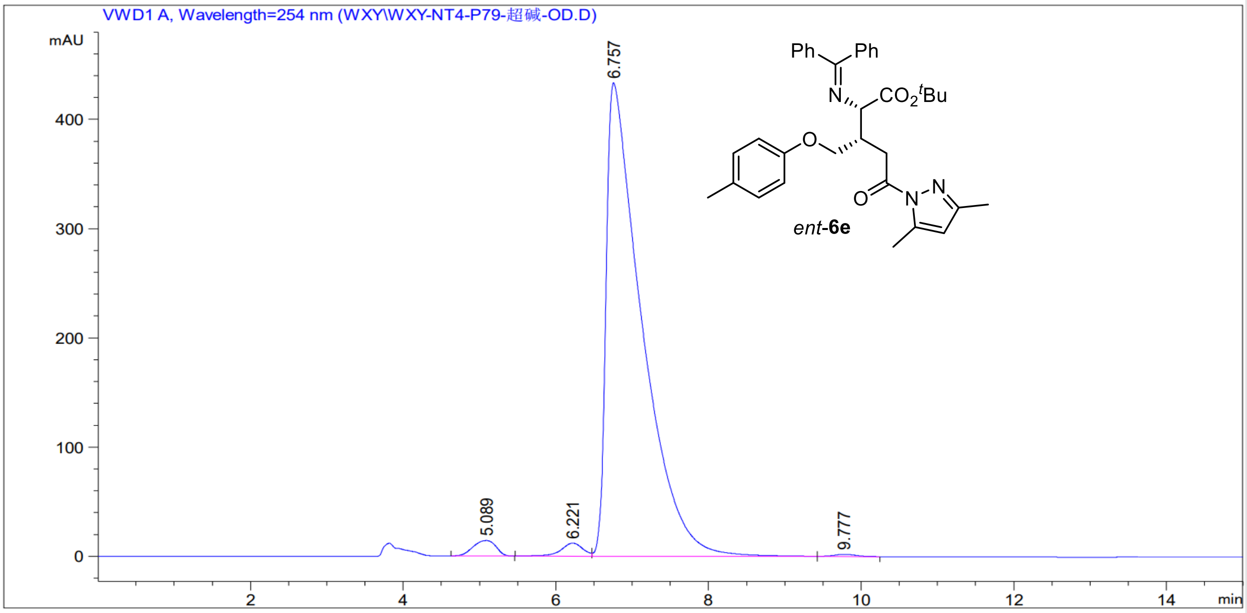

峰 #	保留时间 [min]	类型	峰宽 [min]	峰面积 [mAU*s]	峰高 [mAU]	峰面积 %
1	5.089	BB	0.3402	293.04715	14.17403	2.0103
2	6.221	BV	0.2992	238.88649	12.00224	1.6388
3	6.757	VB	0.4582	1.40078e4	433.30350	96.0946
4	9.777	BB	0.2913	37.35451	1.98687	0.2563

6f: Chiralpak OD-H, Hexane/2-Propanol = 90:10, flow rate 0.8 mL/min, 254 nm, 95% *ee*

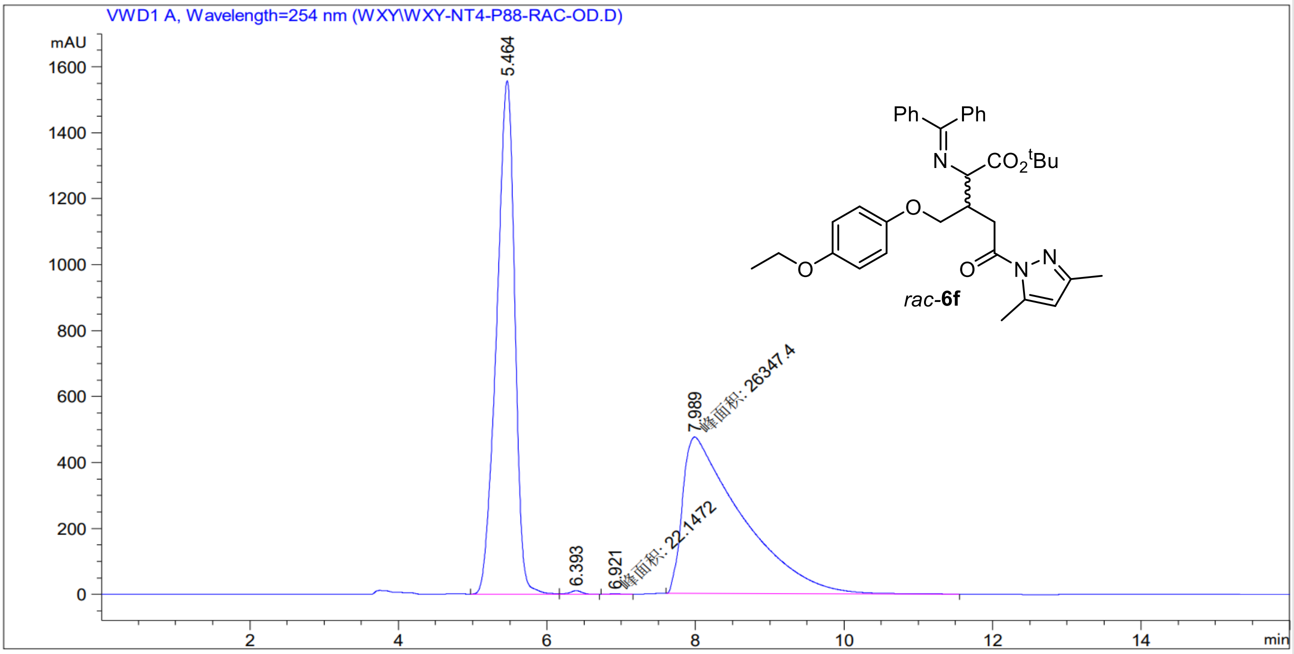

峰 #	保留时间 [min]	类型	峰宽 [min]	峰面积 [mAU*s]	峰高 [mAU]	峰面积 %
1	5.464	VV	0.2591	2.64080e4	1555.43823	49.9210
2	6.393	VB	0.1716	122.09744	10.82084	0.2308
3	6.921	MM	0.2464	22.14723	1.49783	0.0419
4	7.989	MM	0.9258	2.63474e4	474.32773	49.8063

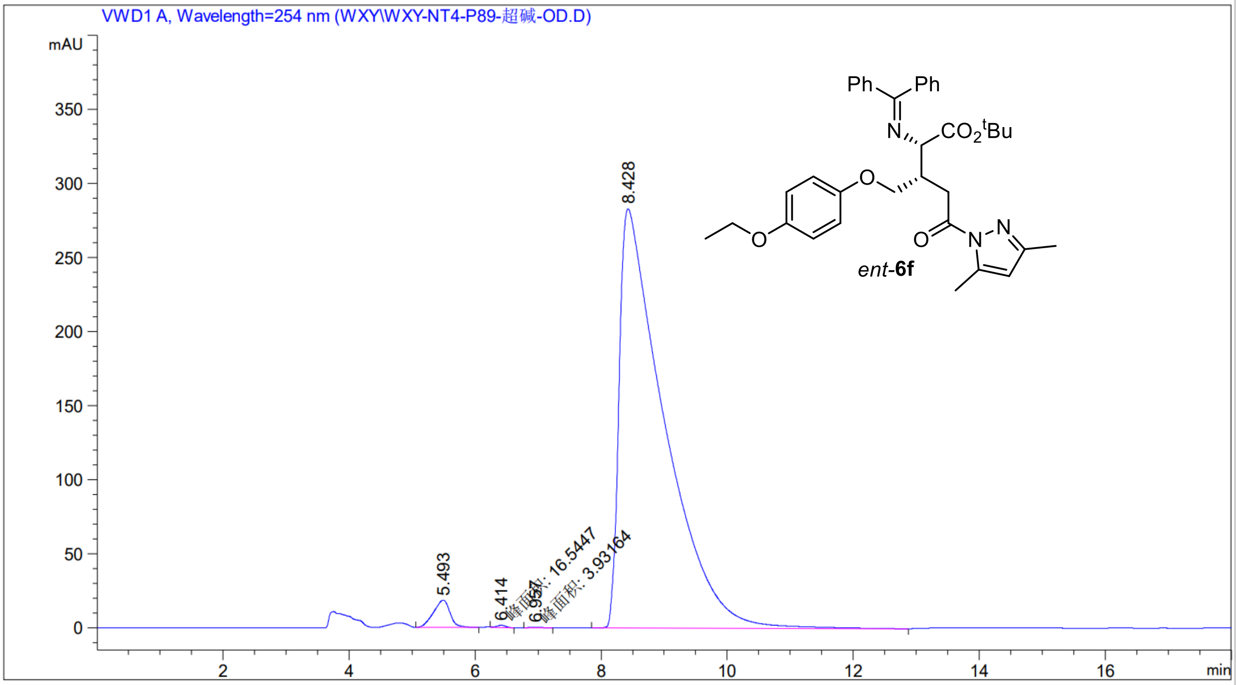

峰 #	保留时间 [min]	类型	峰宽 [min]	峰面积 [mAU*s]	峰高 [mAU]	峰面积 %
1	5.493	BB	0.2758	334.50278	18.33683	2.3213
2	6.414	MM	0.1735	16.54473	1.58939	0.1148
3	6.957	MM	0.1604	3.93164	4.08634e-1	0.0273
4	8.428	BB	0.6896	1.40550e4	282.90964	97.5366

6g: Chiralpak OD-H, Hexane/2-Propanol = 90:10, flow rate 0.8 mL/min, 254 nm, 97% *ee*

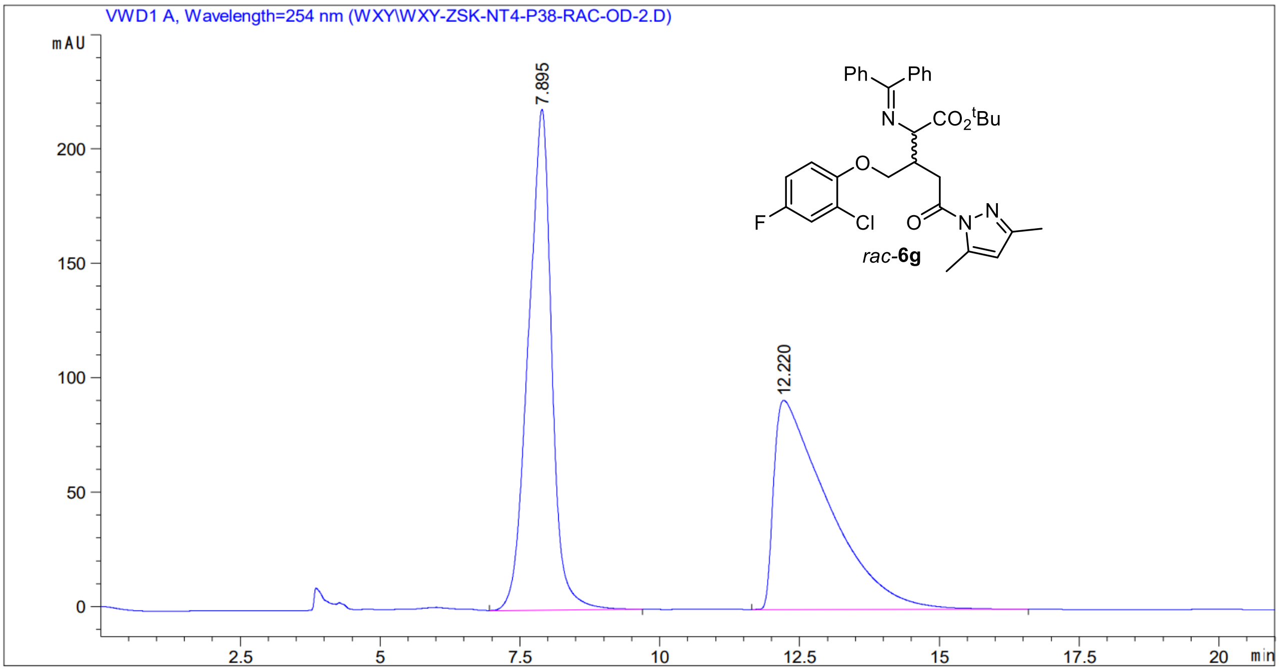

峰 #	保留时间 [min]	类型	峰宽 [min]	峰面积 [mAU*s]	峰高 [mAU]	峰面积 %
1	7.895	BB	0.4148	6373.45361	218.85530	50.6583
2	12.220	BB	0.9603	6207.81201	91.40847	49.3417

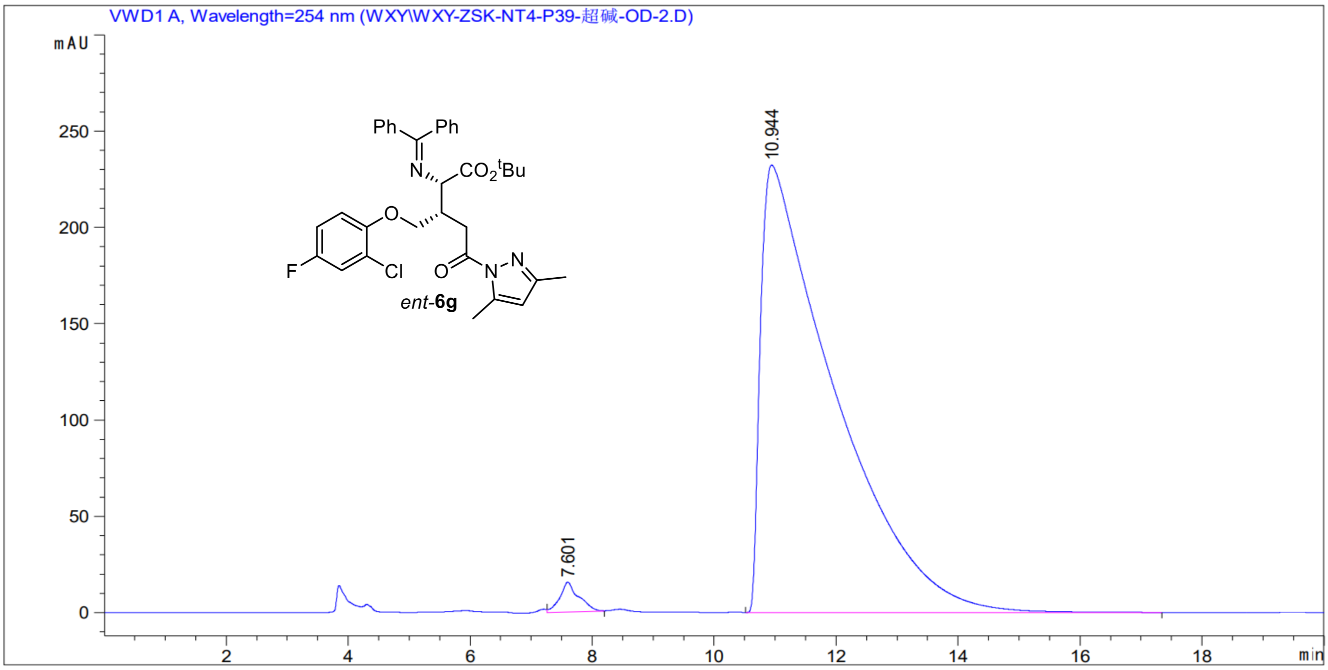

峰 #	保留时间 [min]	类型	峰宽 [min]	峰面积 [mAU*s]	峰高 [mAU]	峰面积 %
1	7.601	VB	0.2884	328.78769	15.44394	1.6537
2	10.944	BB	1.1282	1.95537e4	232.25250	98.3463

6h: Chiralpak OD-H, Hexane/2-Propanol = 90:10, flow rate 0.8 mL/min, 254 nm, 97% *ee*

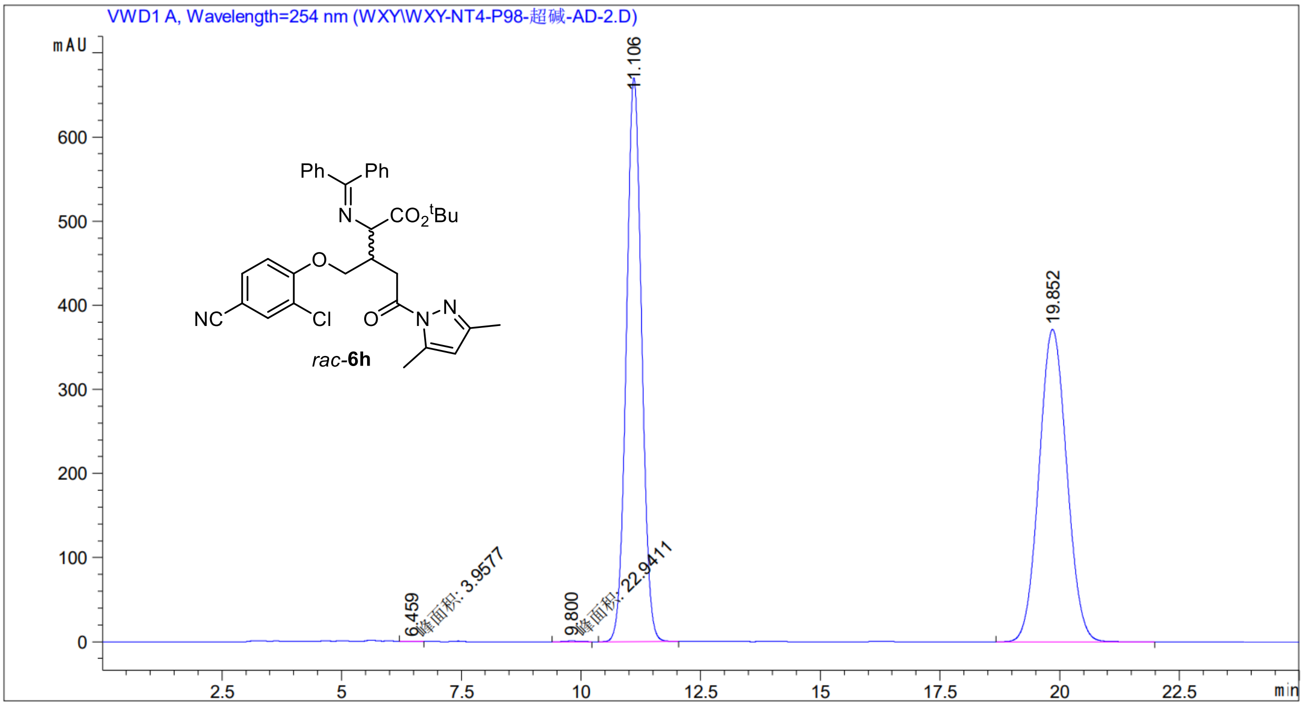

峰 #	保留时间 [min]	类型	峰宽 [min]	峰面积 [mAU*s]	峰高 [mAU]	峰面积 %
1	6.459	MM	0.2389	3.95770	2.76146e-1	0.0132
2	9.800	MM	0.3883	22.94106	9.84555e-1	0.0767
3	11.106	BB	0.3453	1.48993e4	670.25659	49.8457
4	19.852	BB	0.6291	1.49647e4	371.35327	50.0643

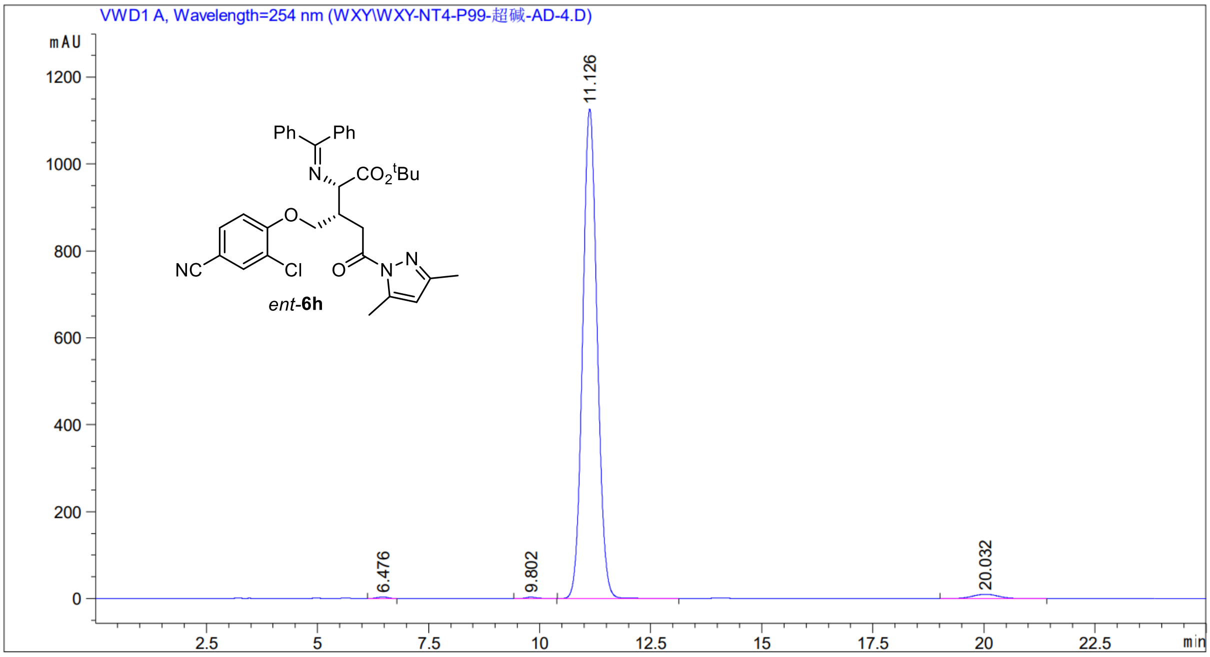

峰 #	保留时间 [min]	类型	峰宽 [min]	峰面积 [mAU*s]	峰高 [mAU]	峰面积 %
1	6.476	BB	0.2655	57.07695	3.49470	0.2191
2	9.802	BB	0.2496	59.40896	3.54274	0.2281
3	11.126	BB	0.3522	2.55224e4	1127.07324	97.9909
4	20.032	BB	0.6247	406.81039	10.04040	1.5619

6i: Chiralpak OD-H, Hexane/2-Propanol = 93:7, flow rate 1.0 mL/min, 254 nm, 98% *ee*

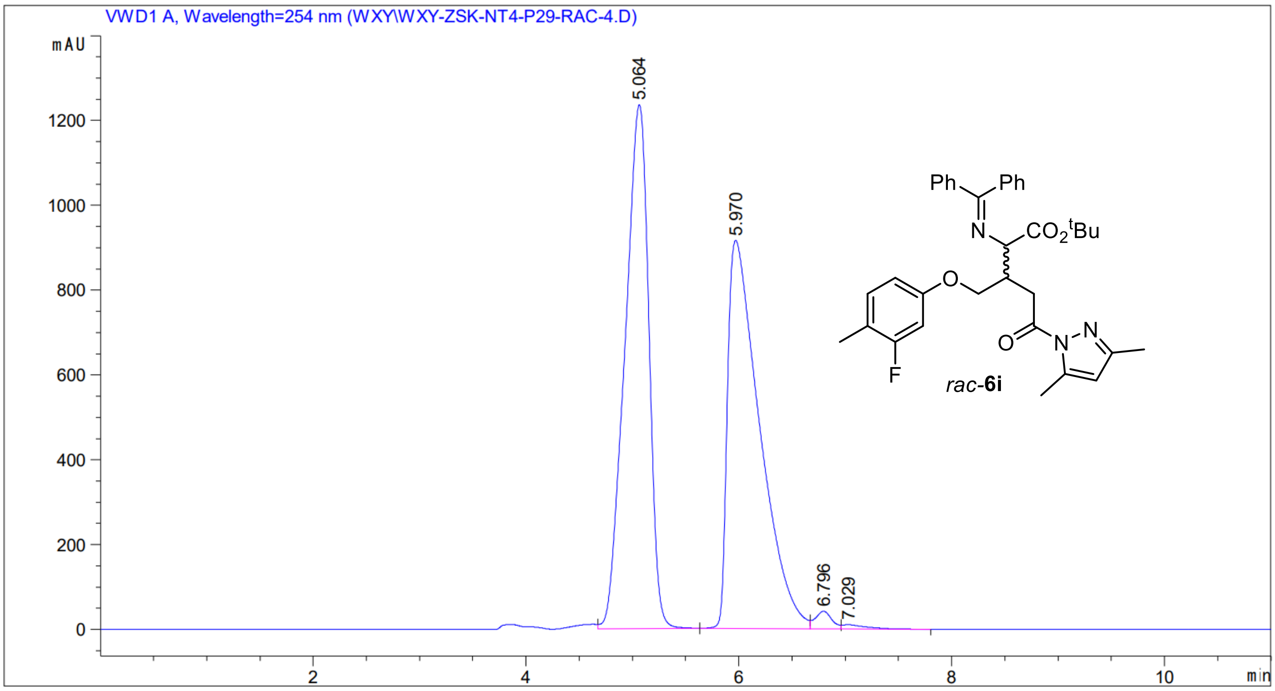

峰 #	保留时间 [min]	类型	峰宽 [min]	峰面积 [mAU*s]	峰高 [mAU]	峰面积 %
1	5.064	VB	0.2366	1.93390e4	1235.35608	49.4970
2	5.970	BV	0.3108	1.91259e4	915.10071	48.9517
3	6.796	VV	0.1592	447.43439	41.69141	1.1452
4	7.029	VB	0.2155	158.65451	10.03292	0.4061

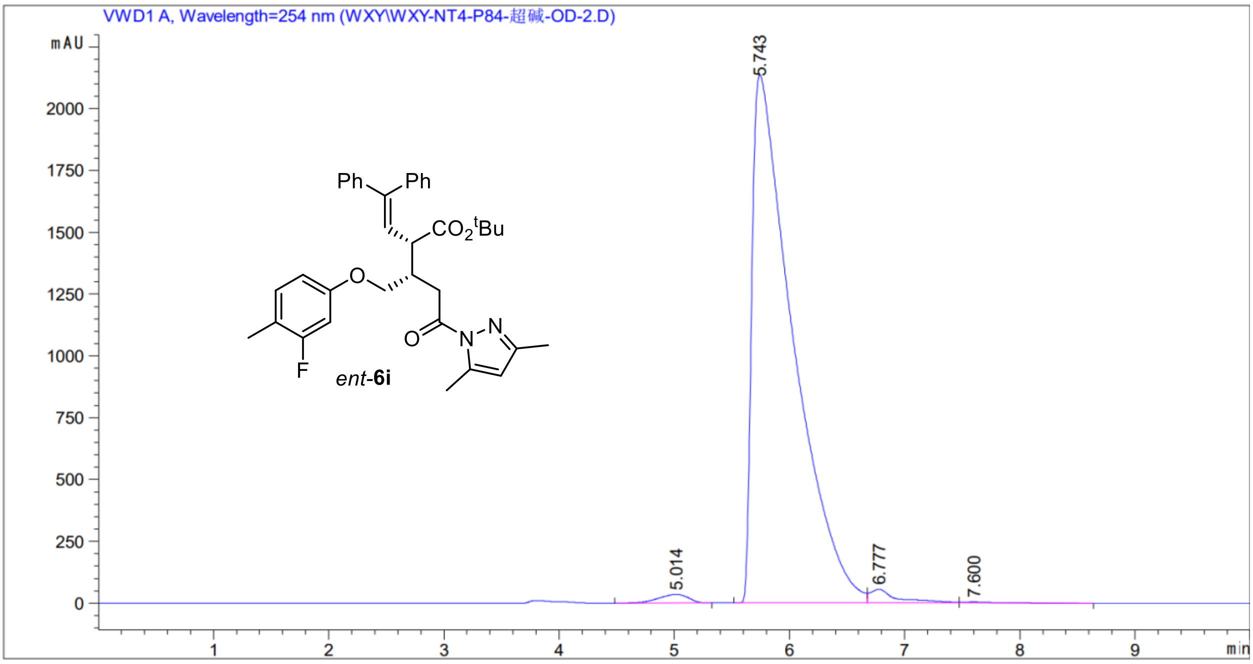

峰 #	保留时间 [min]	类型	峰宽 [min]	峰面积 [mAU*s]	峰高 [mAU]	峰面积 %
1	5.014	BB	0.2919	624.81549	34.72789	1.1525
2	5.743	BV	0.3562	5.25749e4	2135.64355	96.9731
3	6.777	VV	0.2310	936.22681	55.45214	1.7268
4	7.600	VB	0.2660	80.04018	4.38316	0.1476

6j: Chiralpak OD-H, Hexane/2-Propanol = 97:3, flow rate 0.8 mL/min, 254 nm, 98% *ee*

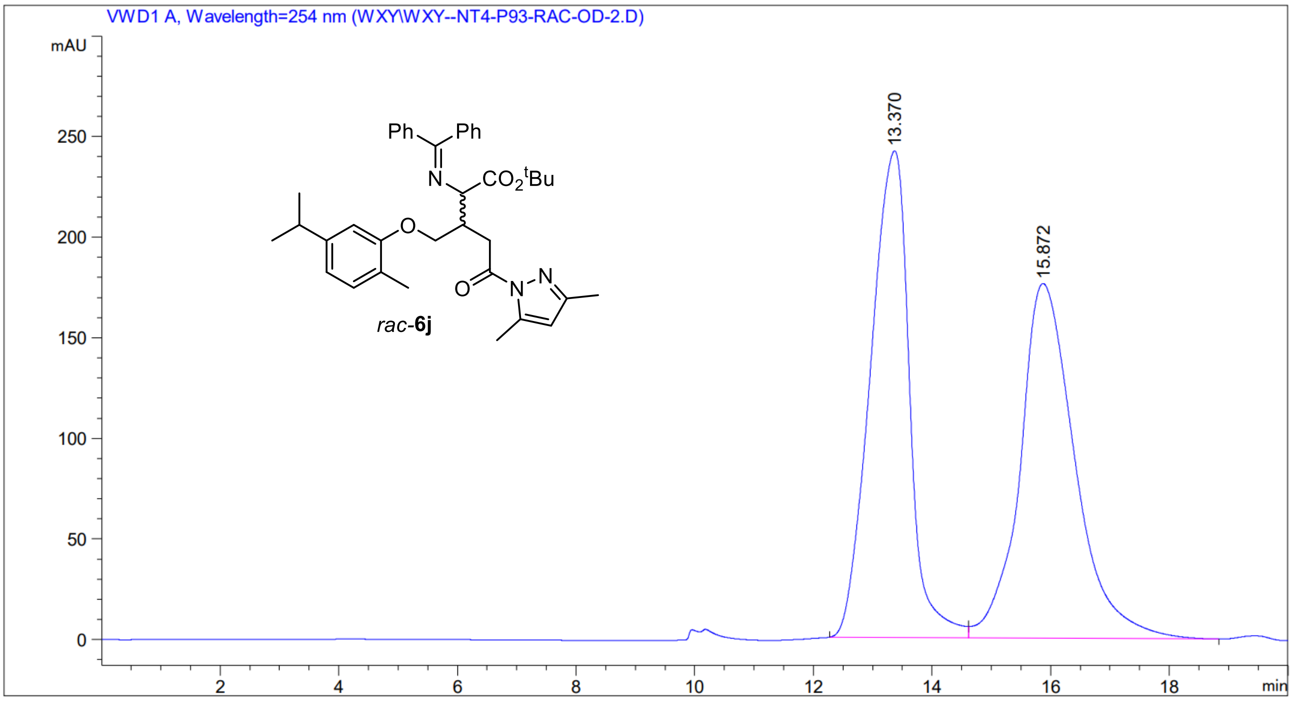

峰 #	保留时间 [min]	类型	峰宽 [min]	峰面积 [mAU*s]	峰高 [mAU]	峰面积 %
1	13.370	BV	0.6690	1.10710e4	241.88066	48.5853
2	15.872	VB	0.9782	1.13490e4	176.25839	49.8056
3	20.529	BB	0.3527	366.66870	15.80240	1.6091

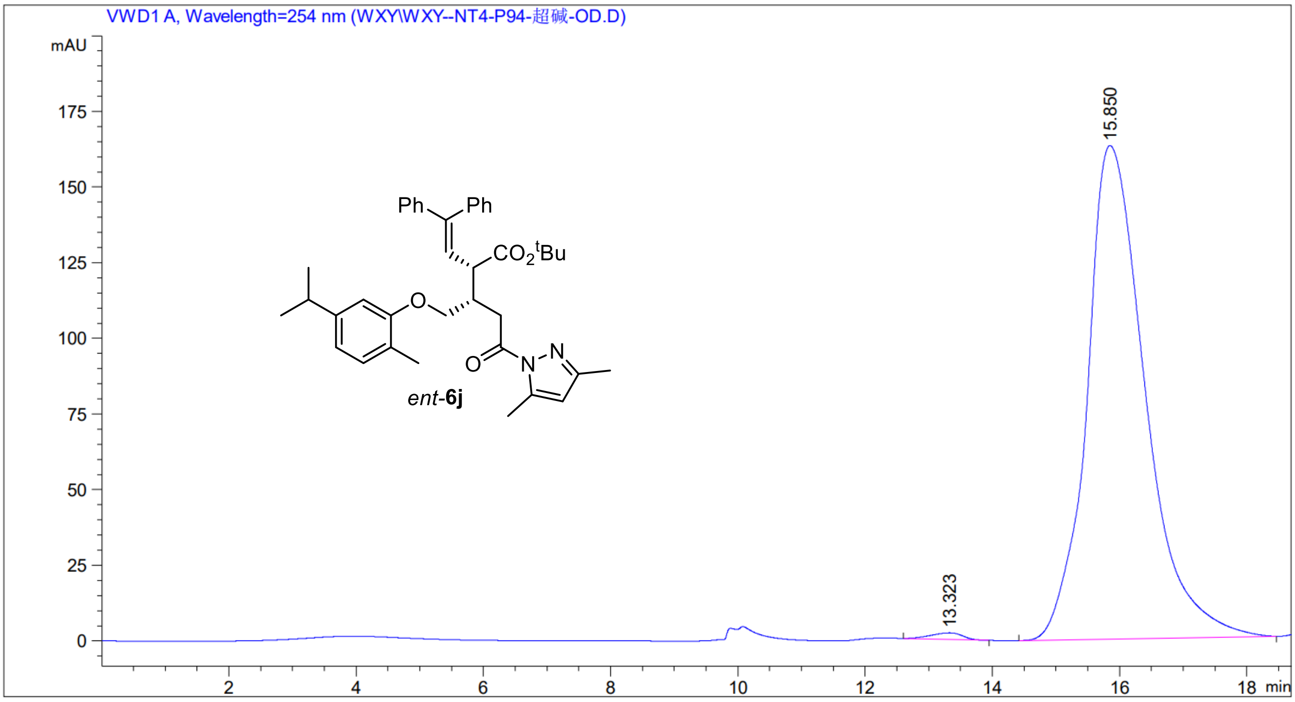

峰 #	保留时间 [min]	类型	峰宽 [min]	峰面积 [mAU*s]	峰高 [mAU]	峰面积 %
1	13.323	BB	0.5116	74.61986	2.17845	0.7025
2	15.850	BB	0.9591	1.03313e4	163.08000	97.2632
3	20.389	VB	0.3448	216.08324	9.41418	2.0343

6k: Chiralpak OD-H, Hexane/2-Propanol = 80:20, flow rate 0.9 mL/min, 254 nm, 86% *ee*

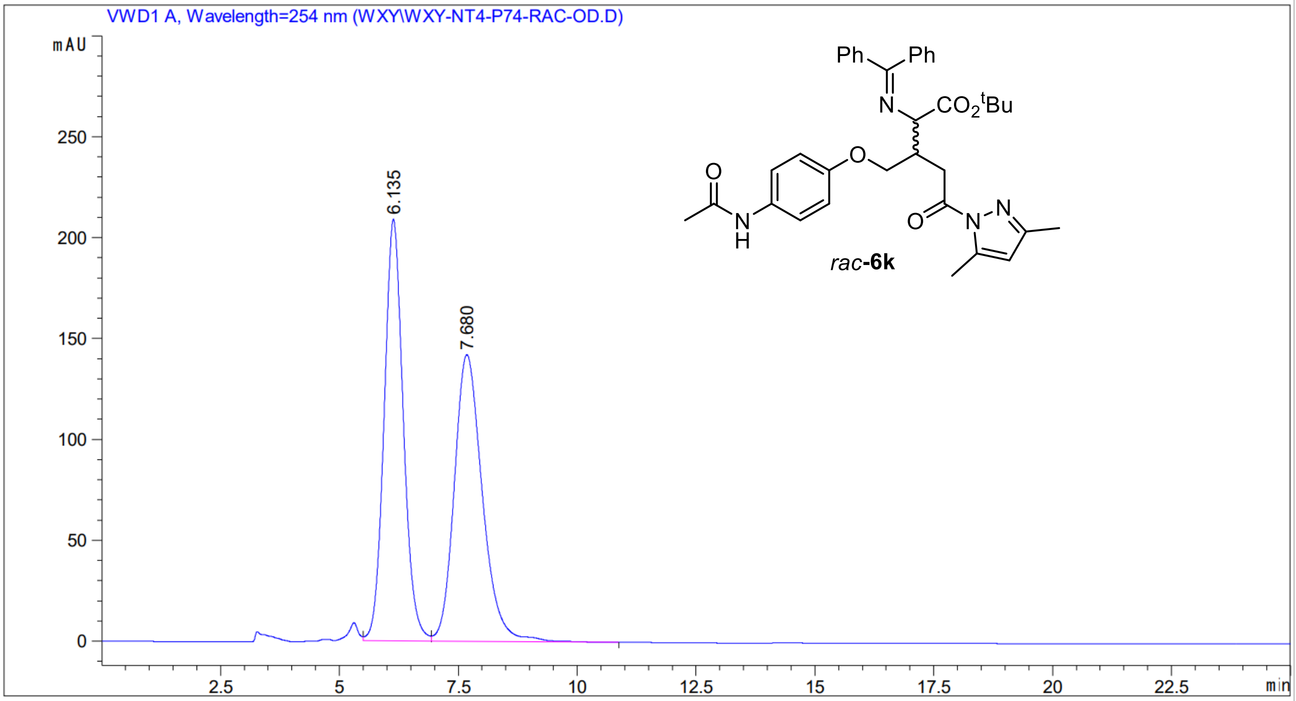

峰 #	保留时间 [min]	类型	峰宽 [min]	峰面积 [mAU*s]	峰高 [mAU]	峰面积 %
1	6.135	VV	0.4291	5858.65186	208.94930	50.0136
2	7.680	VB	0.6298	5855.47119	142.07472	49.9864

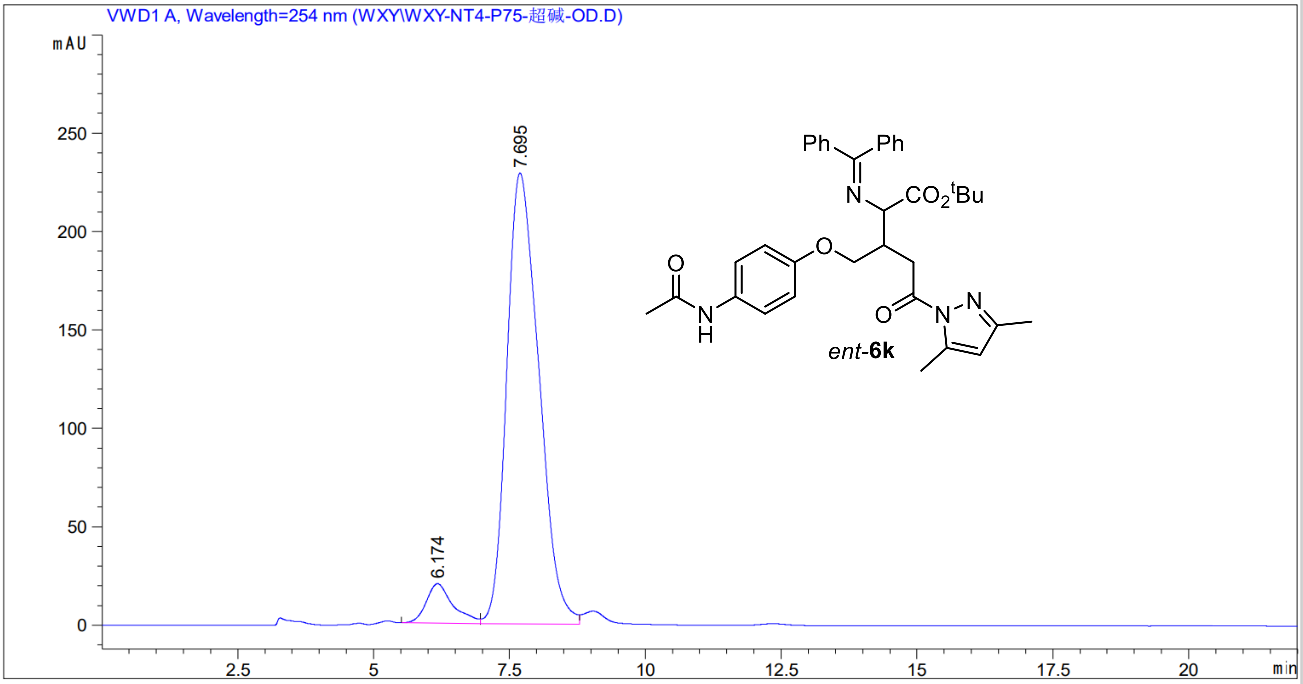

峰 #	保留时间 [min]	类型	峰宽 [min]	峰面积 [mAU*s]	峰高 [mAU]	峰面积 %
1	6.174	BV	0.5042	687.91565	20.05425	6.8167
2	7.695	VV	0.6770	9403.65820	229.04305	93.1833

6l: Chiralpak OD-H, Hexane/2-Propanol = 90:10, flow rate 0.8 mL/min, 254 nm, 98% *ee*

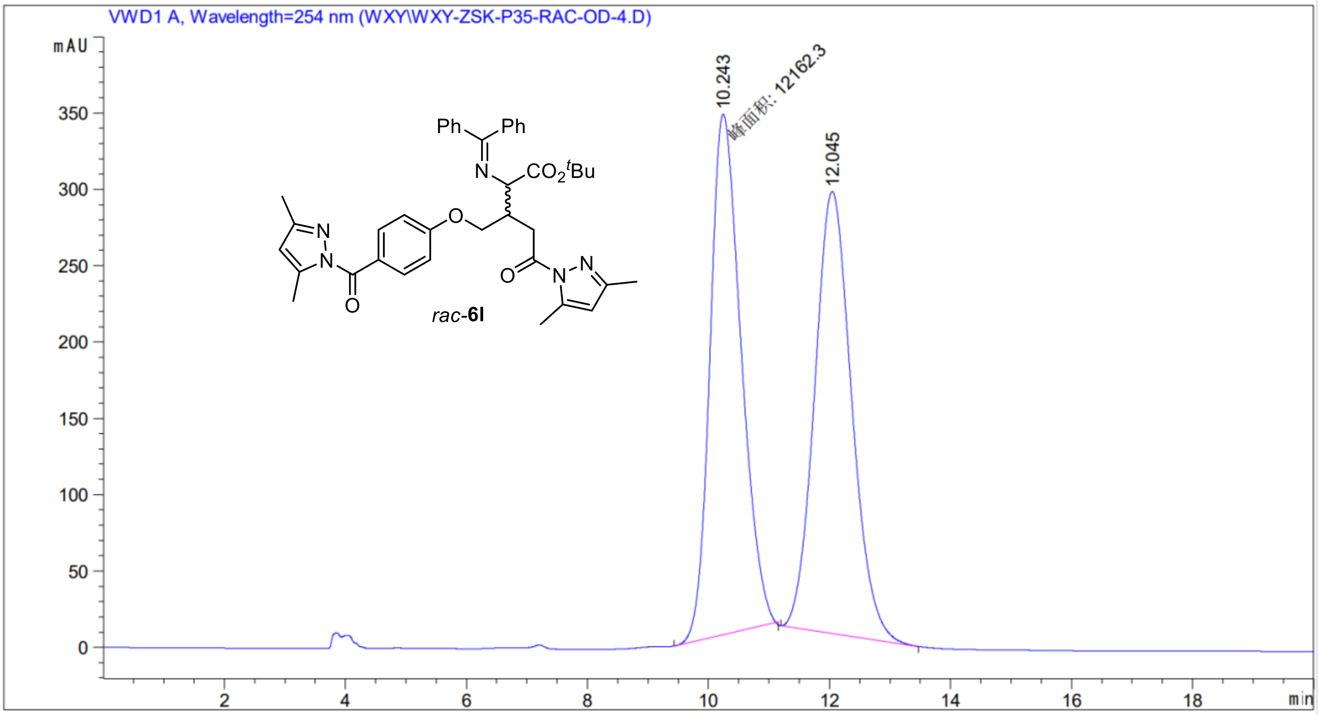

峰 #	保留时间 [min]	类型	峰宽 [min]	峰面积 [mAU*s]	峰高 [mAU]	峰面积 %
1	10.243	MM	0.5945	1.21623e4	340.97479	49.7419
2	12.045	BB	0.6530	1.22885e4	289.64008	50.2581

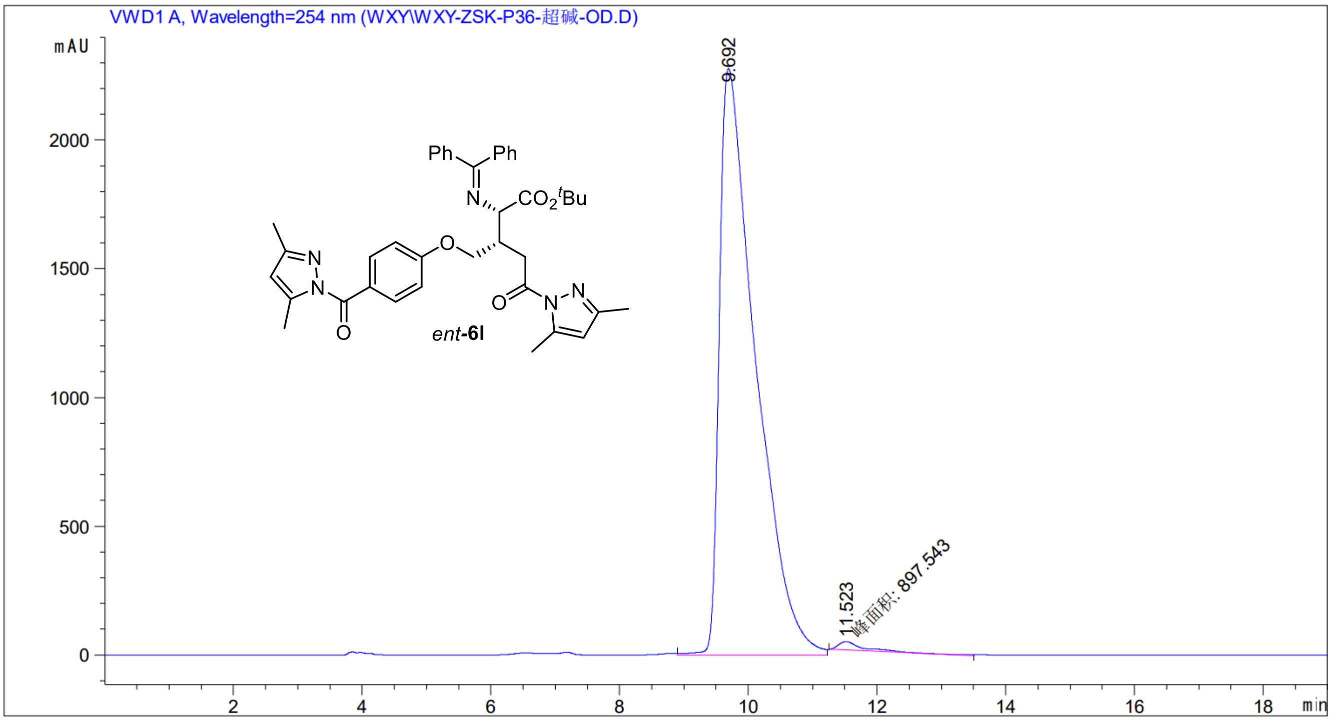

峰 #	保留时间 [min]	类型	峰宽 [min]	峰面积 [mAU*s]	峰高 [mAU]	峰面积 %
1	9.692	VV	0.5601	8.86681e4	2278.07129	98.9979
2	11.523	MM	0.4619	897.54272	32.38932	1.0021

6m: Chiralpak AD-H, Hexane/2-Propanol = 90:10, flow rate 1.0 mL/min, 254 nm, 93% *ee*

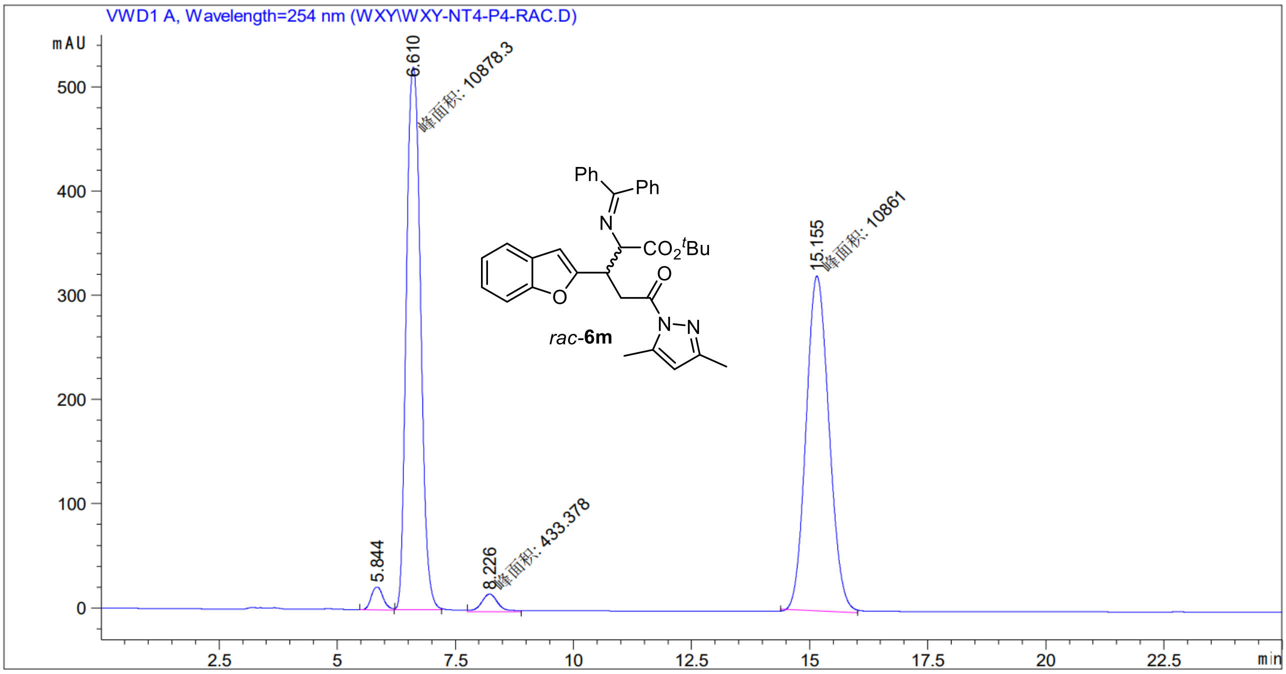

峰 #	保留时间 [min]	类型	峰宽 [min]	峰面积 [mAU*s]	峰高 [mAU]	峰面积 %
1	5.844	BV	0.2761	384.23334	21.96539	1.7034
2	6.610	MM	0.3482	1.08783e4	520.62128	48.2261
3	8.226	MM	0.4212	433.37790	17.14760	1.9213
4	15.155	MM	0.5634	1.08610e4	321.28571	48.1493

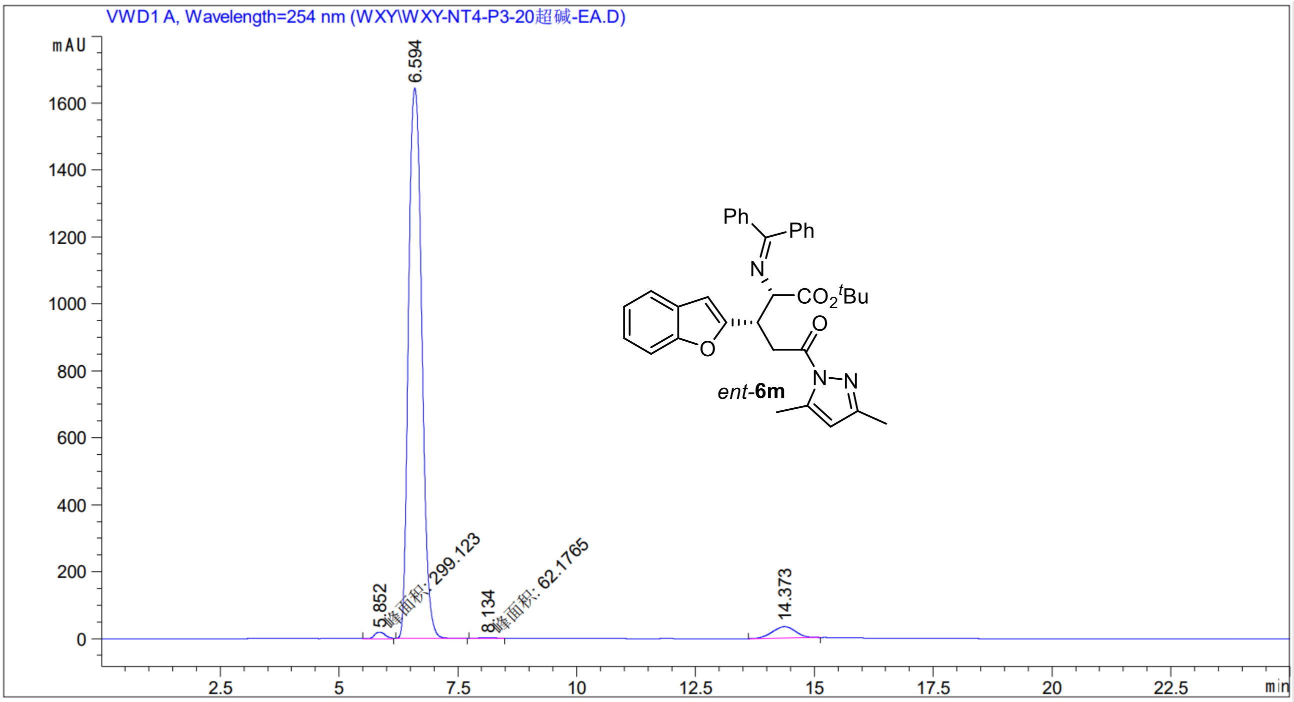

峰 #	保留时间 [min]	类型	峰宽 [min]	峰面积 [mAU*s]	峰高 [mAU]	峰面积 %
1	5.852	MM	0.2686	299.12277	18.55995	0.8931
2	6.594	VB	0.3138	3.19790e4	1644.93250	95.4859
3	8.134	MM	0.3696	62.17649	2.80348	0.1857
4	14.373	BB	0.5298	1150.50146	34.16873	3.4353

6n: Chiralpak AD-H, Hexane/2-Propanol = 90:10, flow rate 1.0 mL/min, 254, 97% *ee*

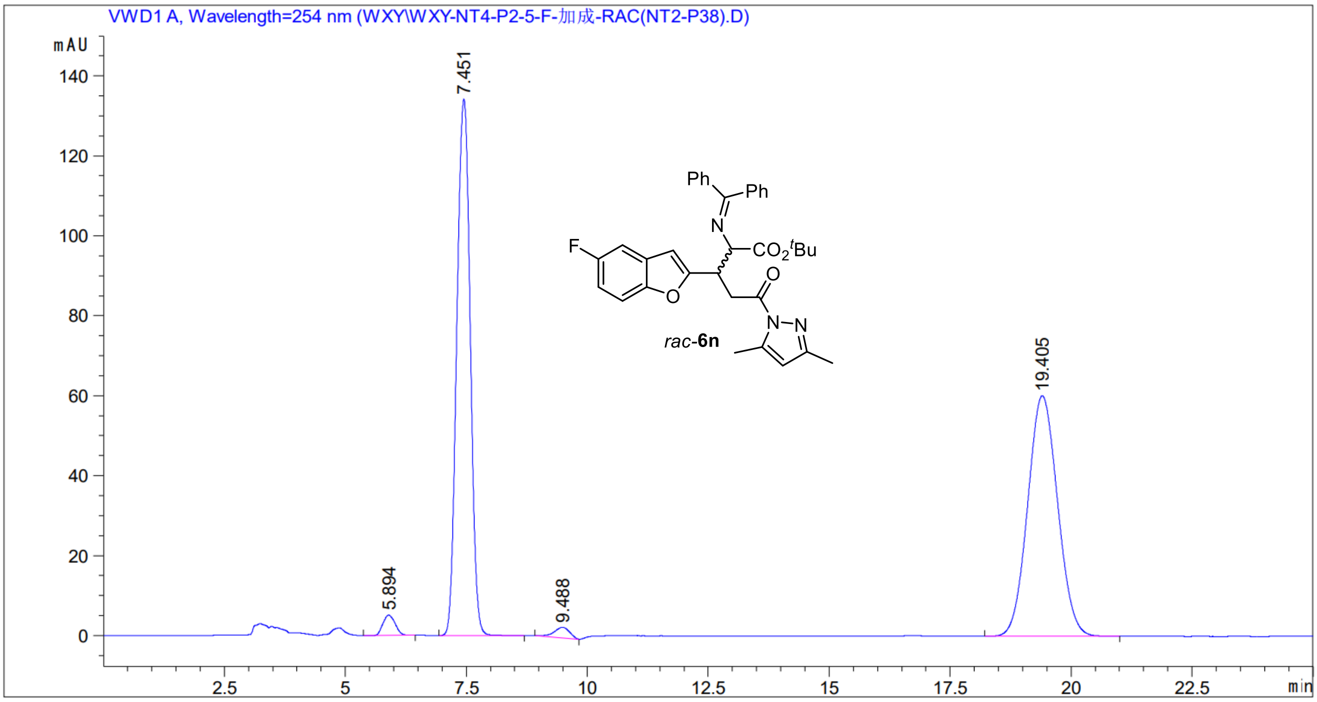

峰 #	保留时间 [min]	类型	峰宽 [min]	峰面积 [mAU*s]	峰高 [mAU]	峰面积 %
1	5.894	BB	0.2888	90.59968	5.11068	1.7046
2	7.451	BB	0.3107	2583.14551	134.14005	48.5999
3	9.488	BB	0.3449	58.08899	2.62747	1.0929
4	19.405	BB	0.6699	2583.29126	60.16844	48.6026

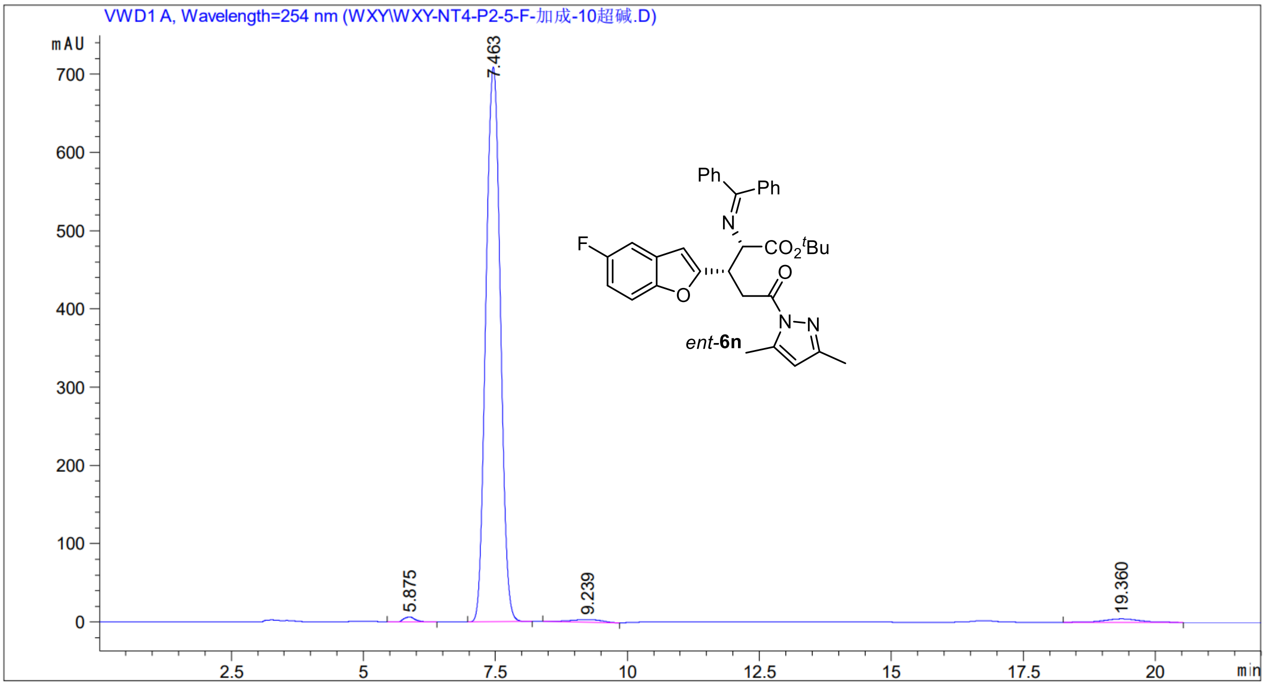

峰 #	保留时间 [min]	类型	峰宽 [min]	峰面积 [mAU*s]	峰高 [mAU]	峰面积 %
1	5.875	BB	0.2467	97.69559	6.30933	0.6956
2	7.463	BB	0.3102	1.36213e4	708.95465	96.9810
3	9.239	BB	0.6418	150.01370	3.53694	1.0681
4	19.360	BB	0.6346	176.31496	4.14180	1.2553

6o: Chiralpak AD-H, Hexane/2-Propanol = 90:10, flow rate 1.0 mL/min, 254 nm, 98% *ee*

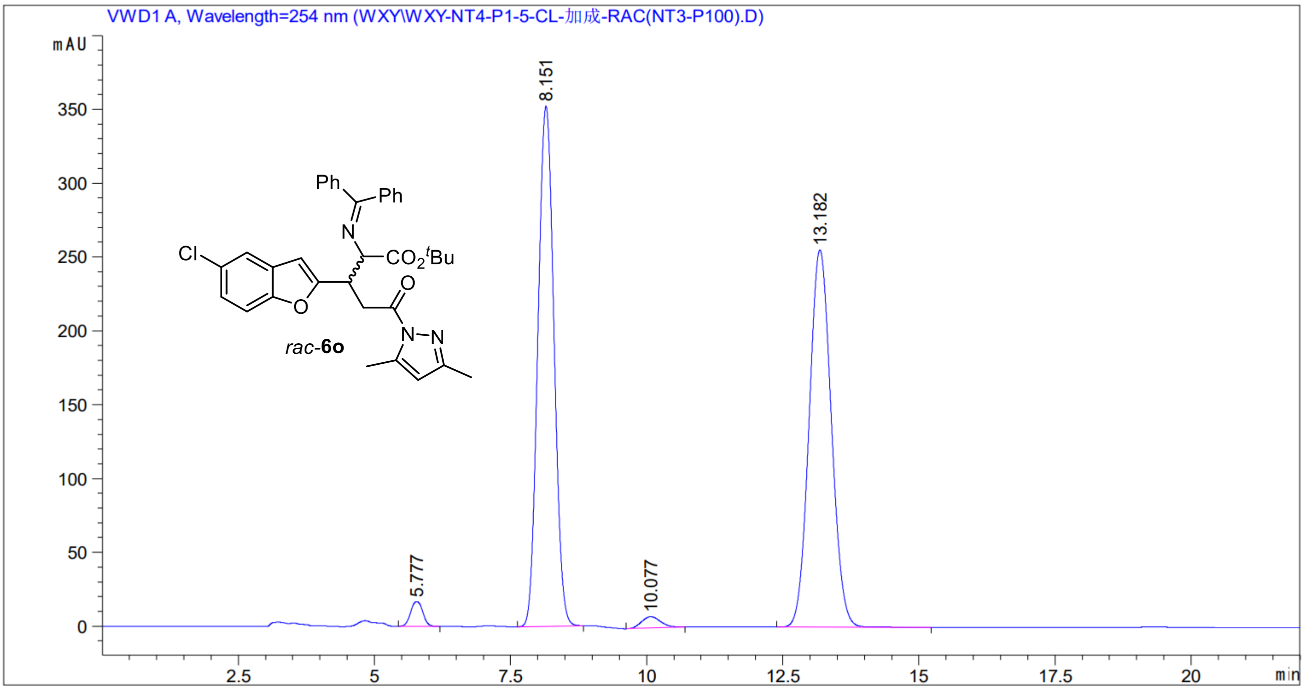

峰 #	保留时间 [min]	类型	峰宽 [min]	峰面积 [mAU*s]	峰高 [mAU]	峰面积 %
1	5.777	BB	0.2469	258.18765	16.74746	1.7415
2	8.151	BB	0.3241	7187.85352	352.15710	48.4814
3	10.077	BB	0.3851	186.07520	7.64001	1.2551
4	13.182	BB	0.4405	7193.88721	255.49200	48.5221

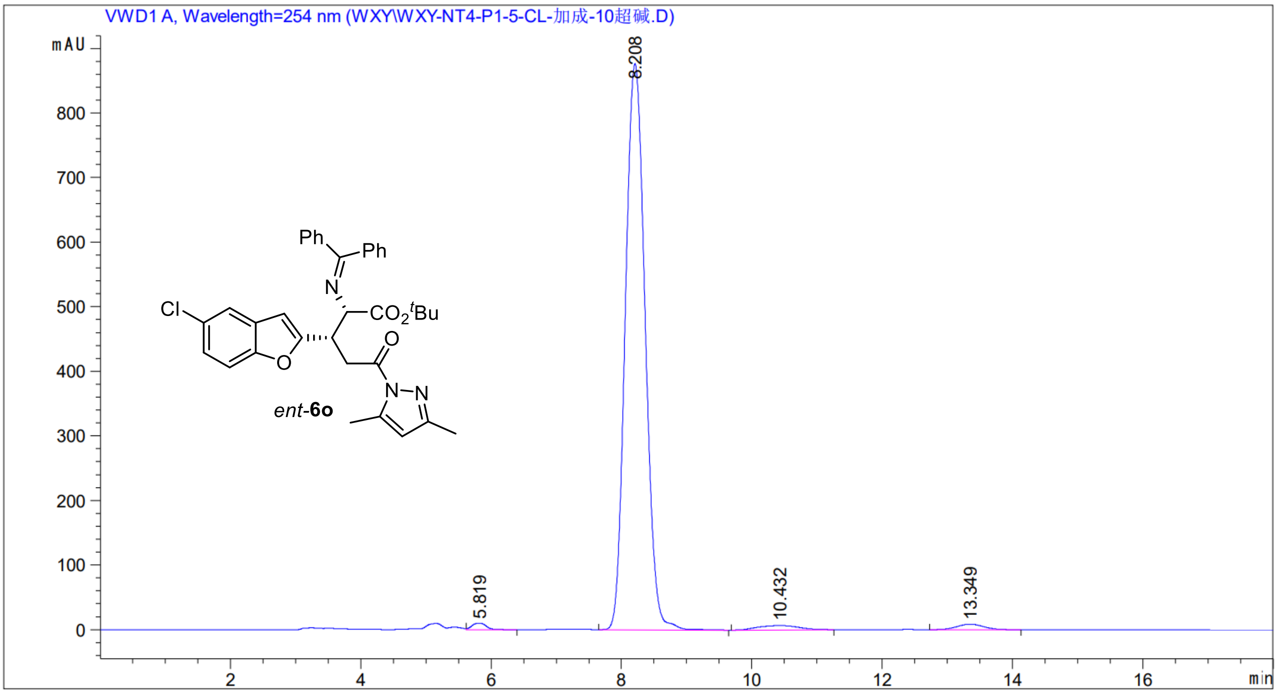

峰 #	保留时间 [min]	类型	峰宽 [min]	峰面积 [mAU*s]	峰高 [mAU]	峰面积 %
1	5.819	VB	0.2396	147.56123	9.97637	0.7729
2	8.208	BB	0.3342	1.84205e4	876.48389	96.4862
3	10.432	BB	0.7181	286.16037	6.58313	1.4989
4	13.349	BB	0.4407	237.11420	8.38959	1.2420

6p: Chiralpak AD-H, Hexane/2-Propanol = 90:10, flow rate 1.0 mL/min, 254 nm, 98% *ee*

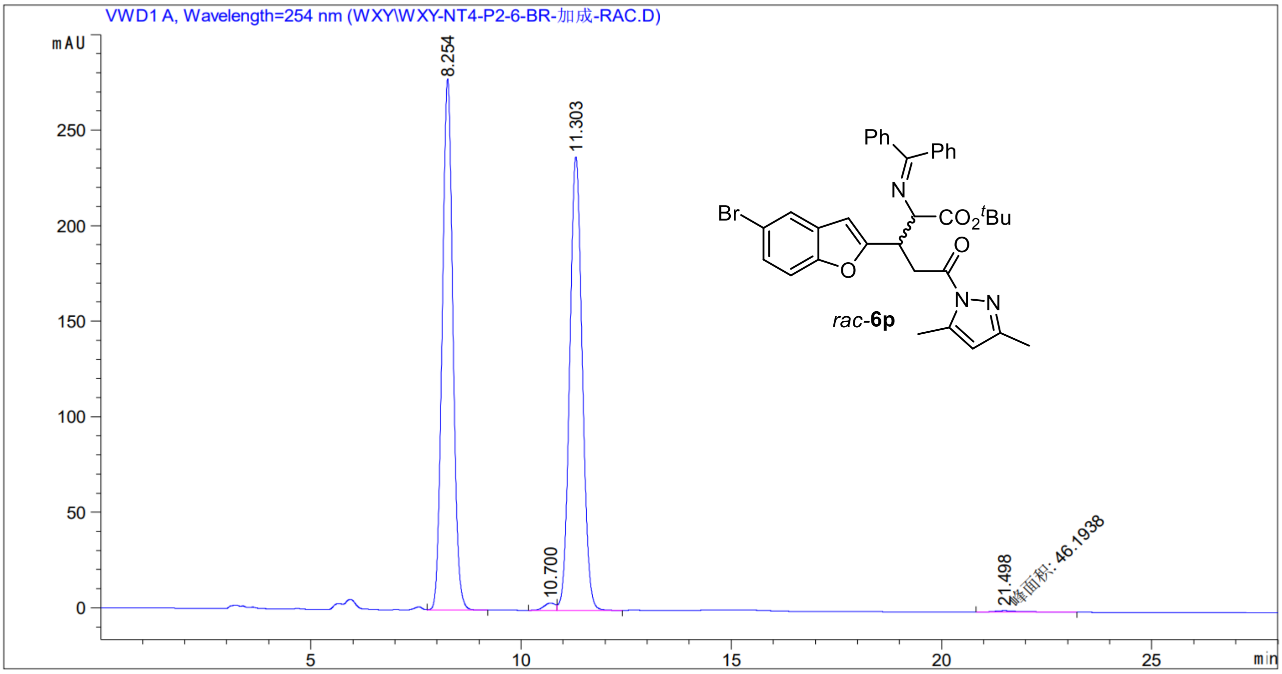

峰 #	保留时间 [min]	类型	峰宽 [min]	峰面积 [mAU*s]	峰高 [mAU]	峰面积 %
1	8.254	VB	0.2739	4906.62744	277.95935	49.3384
2	10.700	BV	0.2841	70.27888	3.79381	0.7067
3	11.303	VB	0.3220	4921.73682	237.24454	49.4904
4	21.498	MM	0.9241	46.19381	8.33150e-1	0.4645

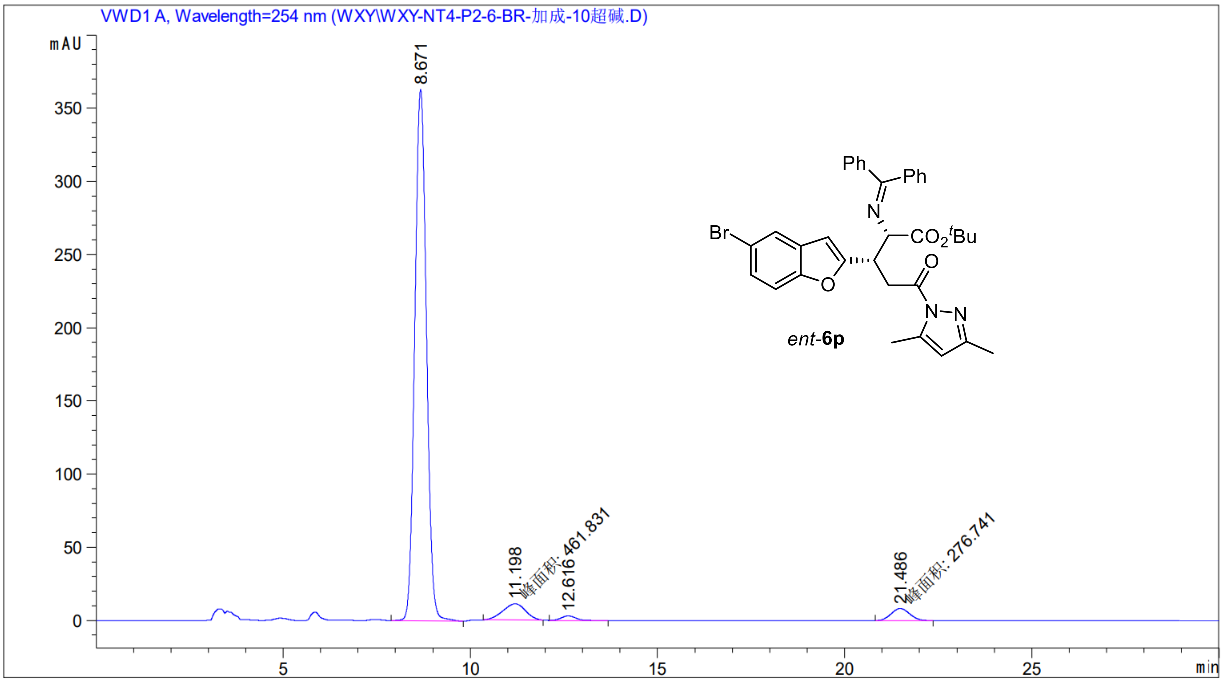

峰 #	保留时间 [min]	类型	峰宽 [min]	峰面积 [mAU*s]	峰高 [mAU]	峰面积 %
1	8.671	BB	0.3401	7845.74463	363.04828	90.5111
2	11.198	MM	0.6975	461.83093	11.03563	5.3278
3	12.616	BB	0.4262	83.95399	3.07766	0.9685
4	21.486	MM	0.5639	276.74081	8.17941	3.1926

6q: Chiralpak AD-H, Hexane/2-Propanol = 90:10, flow rate 1.0 mL/min, 254 nm, 94% *ee*

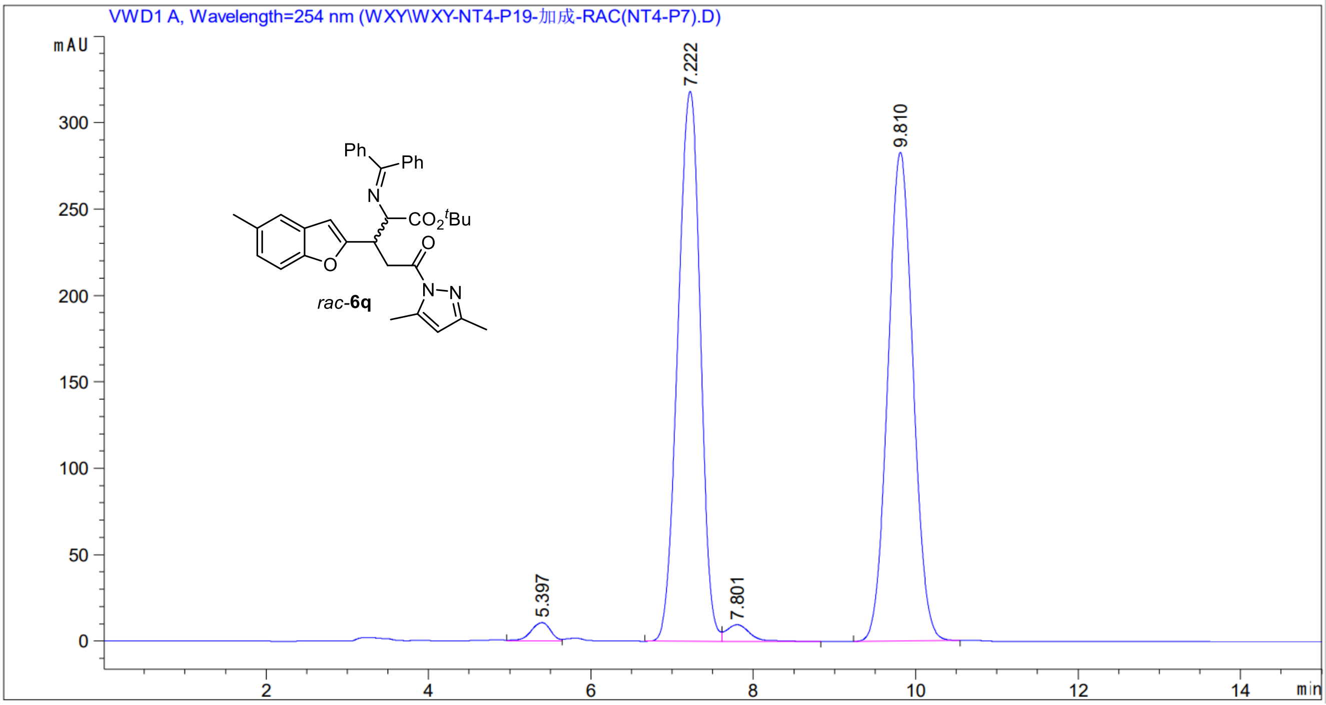

峰 #	保留时间 [min]	类型	峰宽 [min]	峰面积 [mAU*s]	峰高 [mAU]	峰面积 %
1	5.397	VV	0.2686	181.79300	10.67787	1.4438
2	7.222	BV	0.3057	6128.77197	318.17572	48.6749
3	7.801	VB	0.2980	186.53636	9.54449	1.4815
4	9.810	BB	0.3365	6094.13330	282.73517	48.3998

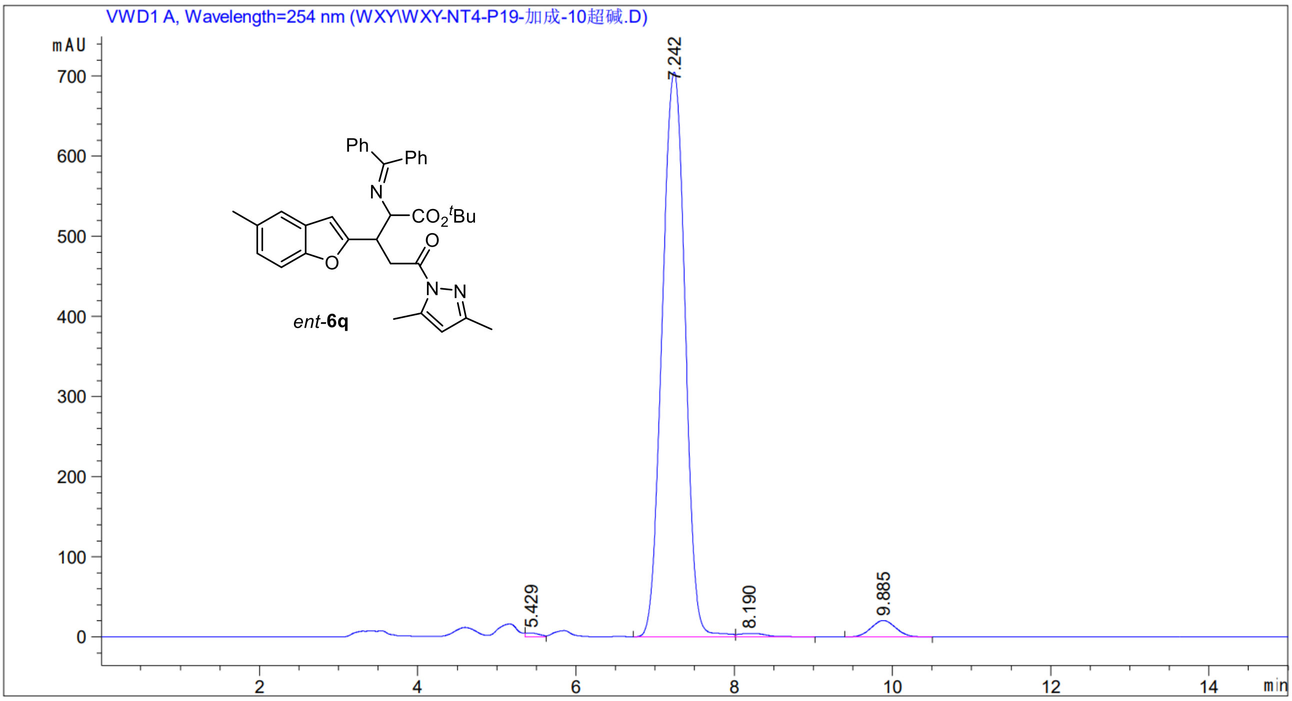

峰 #	保留时间 [min]	类型	峰宽 [min]	峰面积 [mAU*s]	峰高 [mAU]	峰面积 %
1	5.429	VV	0.1838	54.19453	4.49022	0.3671
2	7.242	BV	0.3203	1.41632e4	705.16302	95.9489
3	8.190	VB	0.3686	102.97324	4.28197	0.6976
4	9.885	BB	0.3361	440.82260	20.47915	2.9864

7d: Chiralpak AD-H, Hexane/2-Propanol = 80:20, flow rate 1.0 mL/min, 230 nm, 97% *ee*

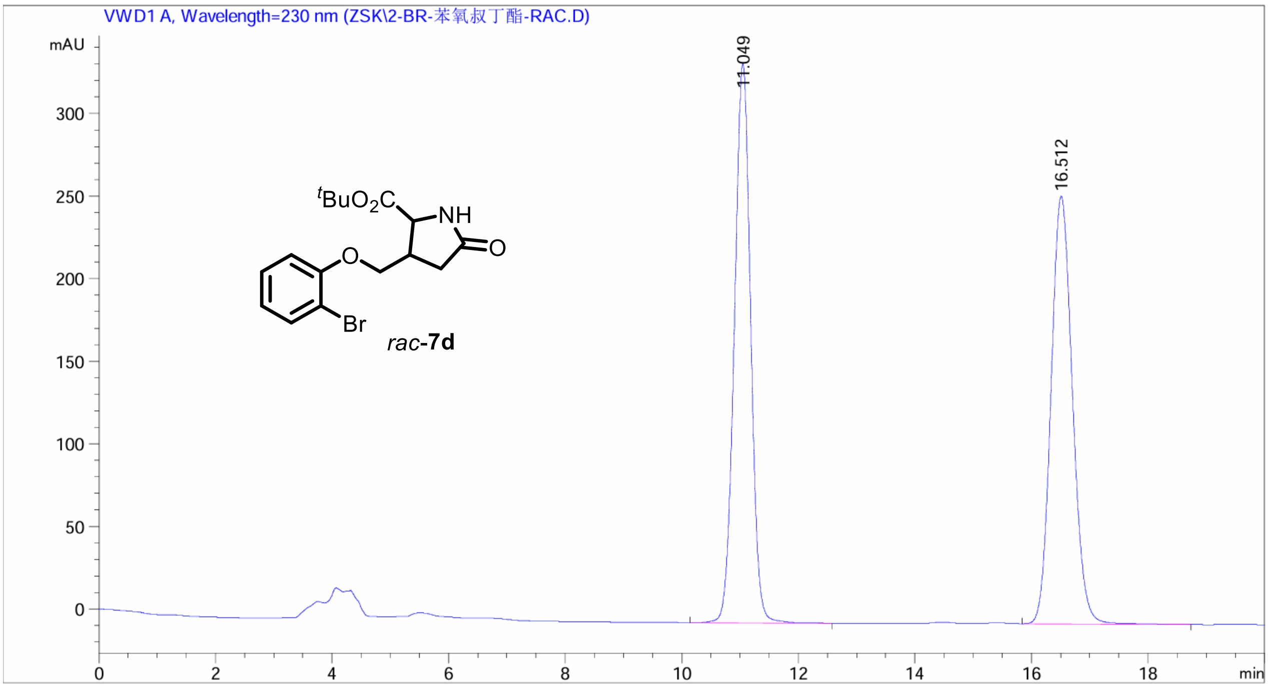

峰 #	保留时间 [min]	类型	峰宽 [min]	峰面积 [mAU*s]	峰高 [mAU]	峰面积 %
1	11.049	BB	0.2987	6553.97754	338.77023	49.8286
2	16.512	BB	0.3973	6599.07764	258.95917	50.1714

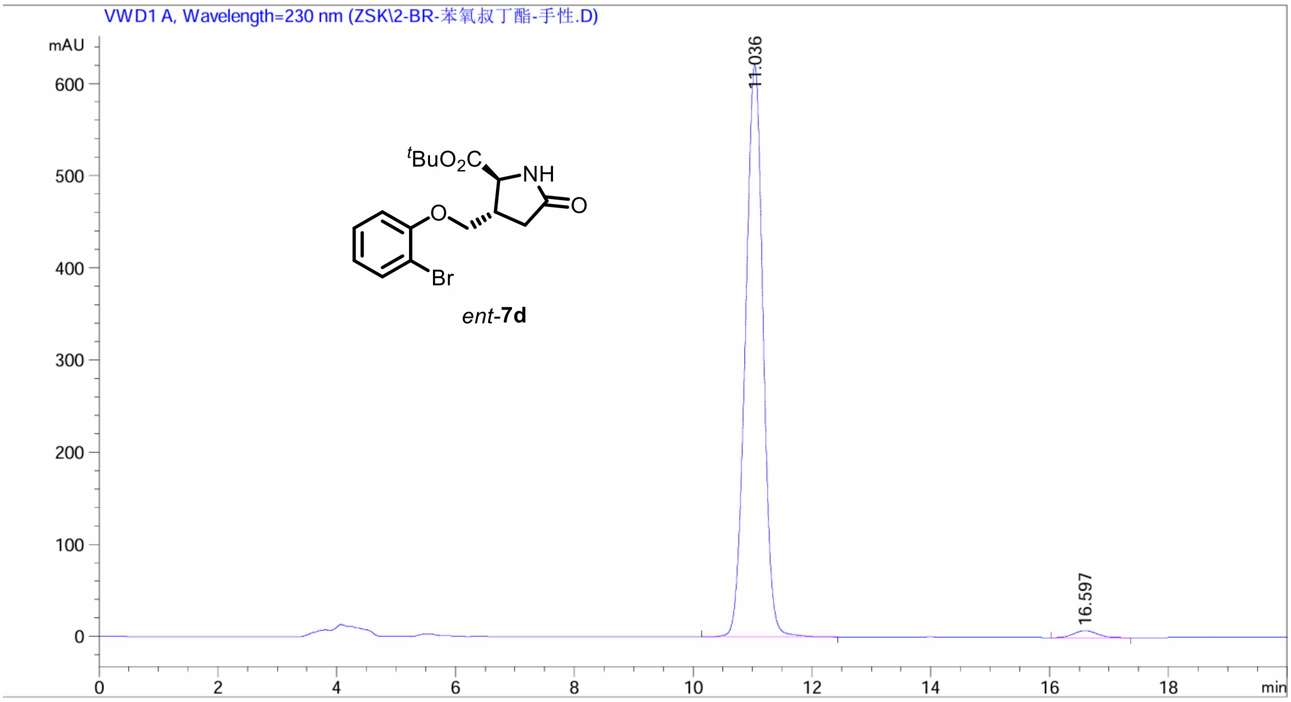

峰 #	保留时间 [min]	类型	峰宽 [min]	峰面积 [mAU*s]	峰高 [mAU]	峰面积 %
1	11.036	BB	0.3192	1.27943e4	621.37842	98.4199
2	16.597	BB	0.4198	205.40405	7.58874	1.5801

7d': Chiralpak AD-H, Hexane/2-Propanol = 80:20, flow rate 1.0 mL/min, 230 nm, 93% *ee*

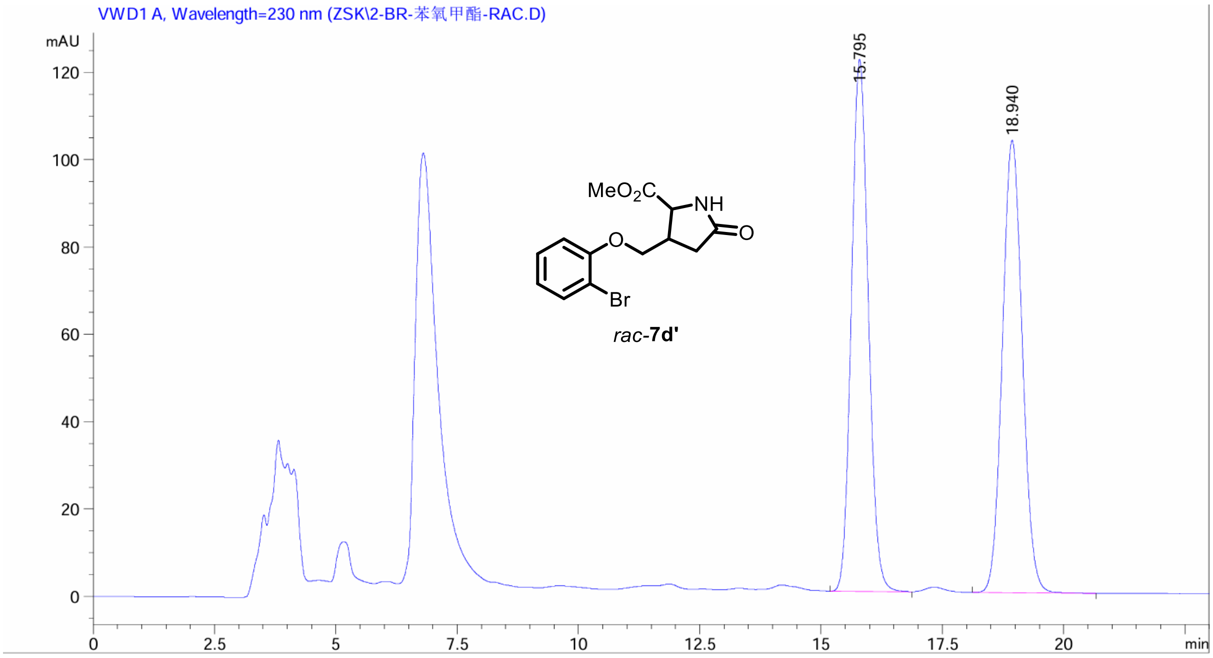

峰 #	保留时间 [min]	类型	峰宽 [min]	峰面积 [mAU*s]	峰高 [mAU]	峰面积 %
1	15.795	BB	0.3765	2950.72119	121.84306	49.8892
2	18.940	BB	0.4455	2963.83276	103.69021	50.1108

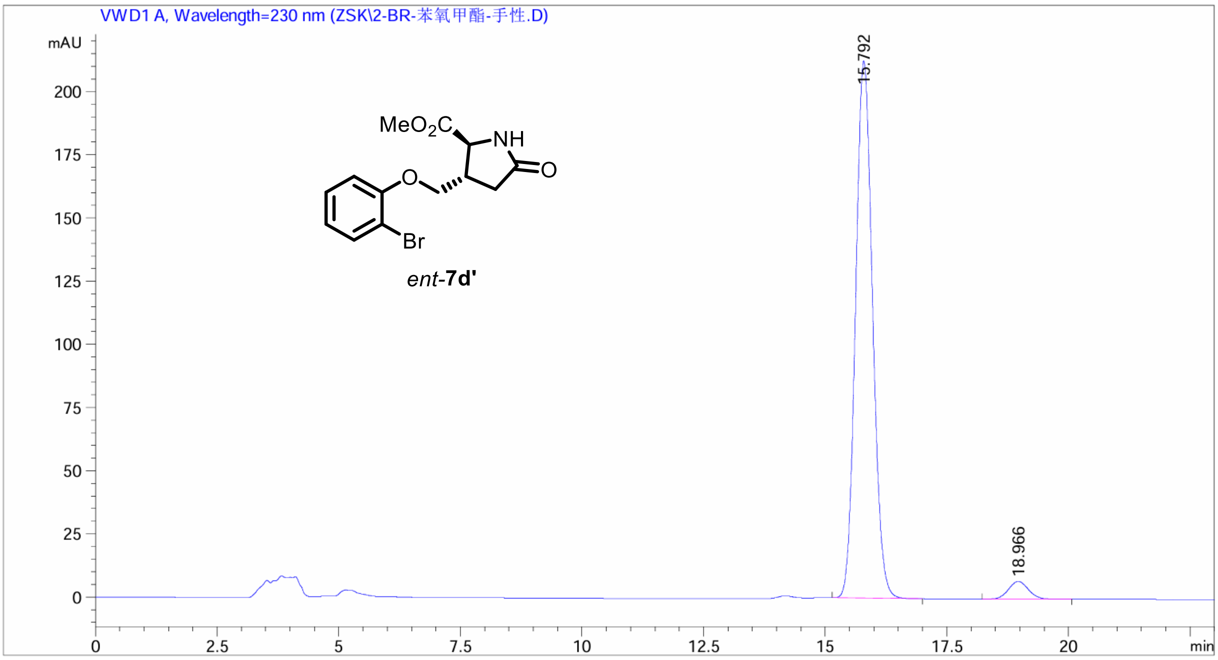

峰 #	保留时间 [min]	类型	峰宽 [min]	峰面积 [mAU*s]	峰高 [mAU]	峰面积 %
1	15.792	BB	0.3780	5155.19385	212.53491	96.2389
2	18.966	BB	0.4432	201.46959	7.03337	3.7611

7h: Chiralpak AD-H, Hexane/2-Propanol = 80:20, flow rate 1.0 mL/min, 230 nm, 98% *ee*

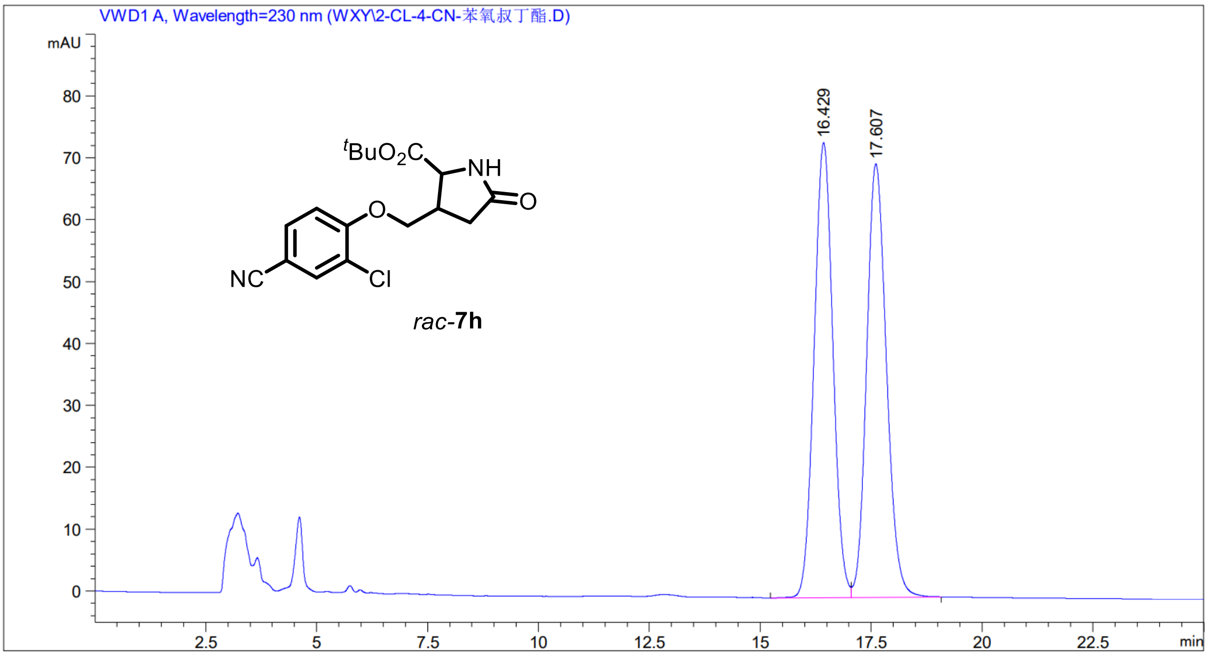

峰 #	保留时间 [min]	类型	峰宽 [min]	峰面积 [mAU*s]	峰高 [mAU]	峰面积 %
1	16.429	BV	0.4582	2162.98218	73.53853	49.8817
2	17.607	VB	0.4810	2173.23975	70.04466	50.1183

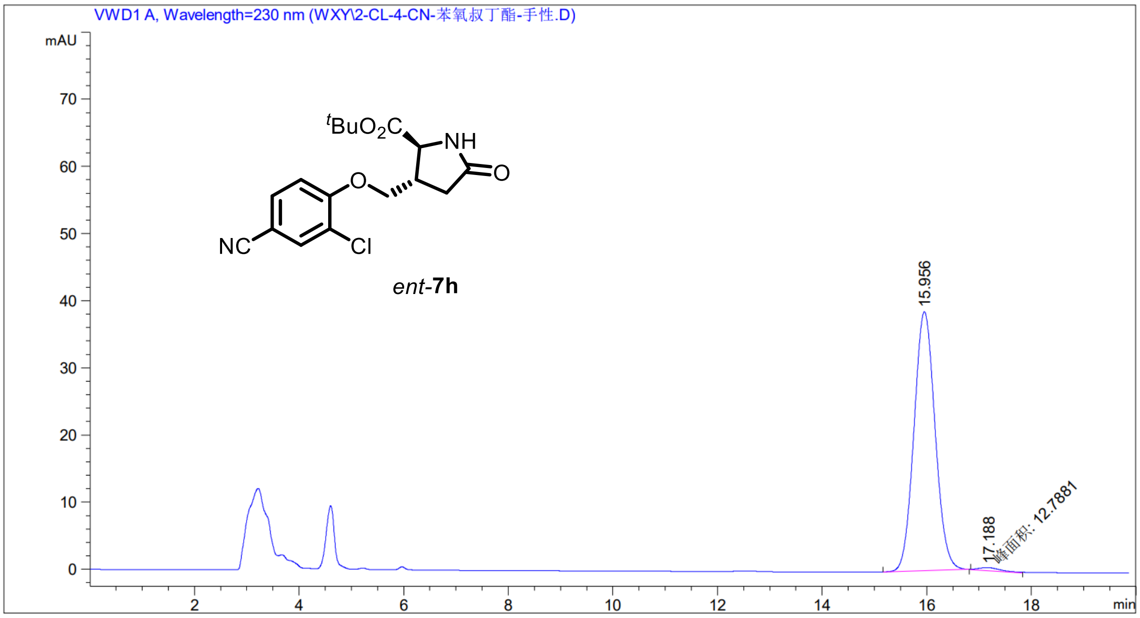

峰 #	保留时间 [min]	类型	峰宽 [min]	峰面积 [mAU*s]	峰高 [mAU]	峰面积 %
1	15.956	BB	0.4367	1080.14148	38.57282	98.8299
2	17.188	MM	0.4883	12.78814	4.36492e-1	1.1701
